# SIRT2 regulates extracellular vesicle-mediated liver–bone communication

**DOI:** 10.1038/s42255-023-00803-0

**Published:** 2023-05-15

**Authors:** Longshuai Lin, Zengya Guo, Enjun He, Xidai Long, Difei Wang, Yingting Zhang, Weihong Guo, Qian Wei, Wei He, Wanying Wu, Jingchi Li, Lulu Wo, Dengli Hong, Junke Zheng, Ming He, Qinghua Zhao

**Affiliations:** 1 Department of Pathophysiology, Key Laboratory of Cell Differentiation and Apoptosis of Ministry of Education, Shanghai Frontiers Science Center of Cellular Homeostasis and Human Diseases, Shanghai Jiao Tong University School of Medicine, Shanghai, China; 2 Department of Orthopedics, Shanghai General Hospital, Shanghai Jiao Tong University School of Medicine, Shanghai, China; 3 Department of Pathology, The Affiliated Hospital of Youjiang Medical University for Nationalities, Baise, China; 4 Department of General Surgery, Shanghai General Hospital, Shanghai Jiao Tong University School of Medicine, Shanghai, China

**Keywords:** Osteoporosis, Bone, Metabolism, Ageing

## Abstract

The interplay between liver and bone metabolism remains largely uncharacterized. Here, we uncover a mechanism of liver-bone crosstalk regulated by hepatocyte SIRT2. We demonstrate that hepatocyte SIRT2 expression is increased in aged mice and elderly humans. Liver-specific SIRT2 deficiency inhibits osteoclastogenesis and alleviates bone loss in mouse models of osteoporosis. We identify leucine-rich α-2-glycoprotein 1 (LRG1) as a functional cargo in hepatocyte-derived small extracellular vesicles (sEVs). In SIRT2-deficient hepatocytes, LRG1 levels in sEVs are upregulated, leading to increased transfer of LRG1 to bone-marrow-derived monocytes (BMDMs), and in turn, to inhibition of osteoclast differentiation via reduced nuclear translocation of NF-κB p65. Treatment with sEVs carrying high levels of LRG1 inhibits osteoclast differentiation in human BMDMs and in mice with osteoporosis, resulting in attenuated bone loss in mice. Furthermore, the plasma level of sEVs carrying LRG1 is positively correlated with bone mineral density in humans. Thus, drugs targeting hepatocyte-osteoclast communication may constitute a promising therapeutic strategy for primary osteoporosis.

## Abstract (web summary)

Lin et al. show that small extracellular vesicles derived from SIRT2-deficient hepatocytes inhibit osteoclast differentiation from mouse and human bone-marrow-derived monocytes and alleviate bone loss in mouse models of osteoporosis.

## Main

Osteoporosis is a common systemic skeletal disease characterized by altered bone metabolism, decreased bone mass, micro-architectural deterioration and increased fragility fracture risk^1,2^. Bone metabolism is characterized by an intimate cooperation of bone cells, including osteoblasts, osteoclasts and osteocytes to maintain bone tissue quantity and the integrity of bone structure^2^. The disruption of the exquisite balance between bone resorption driven by osteoclasts and bone formation mediated by osteoblasts underlies the pathogenesis of osteoporosis^3^. Increasing age, especially in postmenopausal females, is closely associated with the condition^2^. Osteoclasts are multi-nucleated bone-resorbing cells that differentiate from the precursor cells, bone-marrow-derived monocytes (BMDMs), in the presence of two indispensable cytokines: macrophage colony-stimulating factor (M-CSF) and receptor activator of nuclear factor (NF)-κB ligand (RANKL)^4^. Among them, NF-κB, consisting of several subunits such as p50, p52 and p65, is an important downstream transcription factor of the RANKL–RANK signaling pathway. Moreover, RANKL-induced NF-κB p65 activation and nuclear translocation are important for the initial induction of NF of activated T cells cytoplasmic 1 (NFATc1), which is a master regulator of osteoclast differentiation and induces the expression of downstream osteoclast-specific genes, such as those coding for tartrate-resistant acid phosphatase (TRAP), cathepsin K (CTSK), dendritic cell-specific transmembrane protein (DC-STAMP) and osteoclast-associated receptor (OSCAR)^5–7^. Therefore, preventing osteoclast activation, especially negatively regulating NF-κB-NFATc1, is one way to treat osteoporosis in clinic.

Osteoporosis is a systemic bone disease. Besides the intimate cooperation of bone cells, bone metabolism is regulated by the complex communication between bone cells and other organs, providing new insights for osteoporosis therapy^8,9^. Liver is a dynamic organ in many physiological and pathological processes, including the regulation of systemic glucose, lipid and vitamin D (VitD) metabolism^10,11^. Almost all patients with chronic liver disease (CLD) show altered bone metabolism and almost 75% of patients with CLD sooner or later suffer from severe osteoporosis, suggesting that the liver plays a pivotal role in regulating bone remodeling^12^; however, little has been reported about the role of the liver in primary osteoporosis, especially the most frequent senile osteoporosis and postmenopausal osteoporosis. Though there are some proteins secreted by the liver that involve bone metabolism^13^, alterations of VitD metabolism is the most studied liver–bone communication contributor to primary osteoporosis, as VitD is hydroxylated by VitD 25-hydroxylase (CYP2R1) and sterol 27-hydroxylase (CYP27A1) in the liver^12^; however, VitD supplementation alone is not sufficient to prevent or delay loss of bone mineral density (BMD) in patients with osteoporosis. These studies suggest that the other unknown liver–bone communications are crucial and required for the pathogenesis and development of primary osteoporosis.

sEVs may be produced by diverse cells and function as important cell–cell messengers^14^. Hepatocytes are sEV-releasing and/or sEV-targeted cells. Moreover, hepatocyte-derived sEVs are released under either physiological or pathological conditions, including aging and liver diseases, and exert a wide range of effects on target cells by transmitting hepatocyte-associated protein cargo as well as messenger RNA, micro RNA and lipids^15^. In bone, sEVs are involved in the communication between bone cells for bone remodeling, with a predominantly paracrine effect^16^. Recent studies have detected that osteoblast-derived sEVs could fuse with osteoclasts to promote osteoclastogenesis and boost the clearance of damaged tissue during bone remodeling^17^; however, the pathophysiological effects of hepatocyte-derived sEVs in bone remodeling, especially osteoblastogenesis and osteoclastogenesis, have so far not been described.

Sirtuin 2 (SIRT2) is a nicotinamide adenine dinucleotide (NAD^+^)-dependent protein deacetylase and the only sirtuin mainly located in the cytoplasm and abundantly expressed in the liver^18^. Accumulating studies have found that SIRT2 plays an important role in the regulation of life activities such as aging, metabolism, apoptosis, cell differentiation, cell cycle, inflammation and tumorigenesis; however, SIRT2 plays controversial and multiple roles by deacetylating different substrates in diverse liver diseases, including alcoholic liver disease (ALD), nonalcoholic fatty liver disease (NAFLD), liver fibrosis and hepatic ischemia-reperfusion (I/R) injury^18–20^. Our previous work has demonstrated that SIRT2 in macrophages prevents aging-associated inflammation and maintains hepatic insulin sensitivity during physiological aging through deacetylation of NLRP3 (ref. ^21^); however, the contribution of SIRT2 in hepatocytes to bone homeostasis and osteoporosis is unknown.

Here, we uncovered hepatocyte–osteoclast communication regulated by SIRT2 with therapeutic potential in osteoporosis. We verified that hepatocyte SIRT2 expression increased with aging both in mice and humans. The liver-specific SIRT2 deficiency (*SIRT2*-KO^hep^) abolishes bone loss and osteoporosis in aged mice and an ovariectomy (OVX)-induced postmenopausal osteoporosis mouse model. We elucidated the mechanism that *SIRT2*-KO^hep^-upregulated leucine-rich α-2-glycoprotein 1 (LRG1) in hepatocyte-derived sEVs (sEV-LRG1) transfers to BMDMs via blood and then suppresses osteoclastogenesis through inhibiting NF-κB p65 activation. Moreover, we show that AGK2, a specific inhibitor for SIRT2 and osteoclast-targeted sEV-LRG1 treatment could repress the differentiation of osteoclasts from both OVX mice and human primary mononuclear cells. In addition, using BMDM-specific *SIRT2* knockout mice, we identified that the hepatocyte SIRT2-regulated liver–bone axis, not BMDM-intrinsic SIRT2, is the predominant regulator of osteoclastogenesis and osteoporosis. Moreover, LRG1-sEV treatment was superior to denosumab in a rebound effect of human osteoclastogenesis. The clinical data further verified that plasma sEV-LRG1 expression was strongly and positively correlated with BMD and negatively related with bone resorption markers in patients.

## Results

### Hepatocyte-specific SIRT2 deficiency prevents aging-associated bone loss by suppressing osteoclastogenesis in mice

**Fig. 1:**
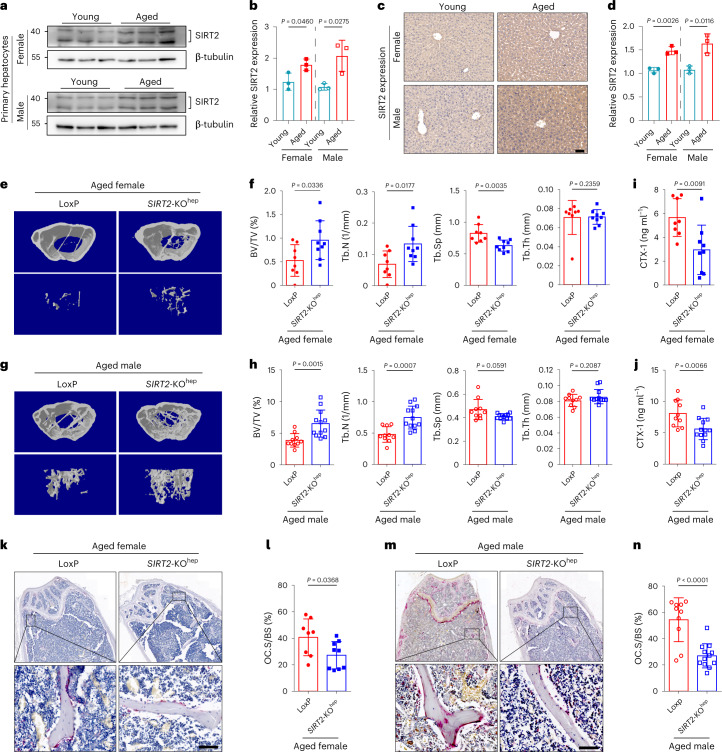
Hepatocyte-specific *SIRT2* knockout prevents age-related bone loss with less-active osteoclastogenesis. **a**, Western blot analysis of SIRT2 protein expression in primary hepatocytes of aged female and male mice; *n* = 3 mice, two technical replicates of three biological replicates for each group and results from one experiment are shown. **b**, Western blot density analyzed by ImageJ and quantification analysis was shown (young mice, 3 months of age, *n* = 3 and aged mice, 18 months of age, *n* = 3,). **c**, Represented IHC images of SIRT2 protein expression in liver tissues from young and aged mice (scale bar, 50 µm); *n* = 3 mice, one technical replicate of three biological replicates for each group. **d**, SIRT2 protein expression intensity analyzed by ImageJ and quantification analysis was shown (young mice, 3 months of age, *n* = 3 and aged mice, 18 months of age, *n* = 3,). **e**,**f**, Represented images of 3D restoration and quantification of trabecular BV/TV, Tb.N, Tb.Sp and Tb.Th of distal femurs of aged female LoxP and *SIRT2*-KO^hep^ mice (18 months of age), as measured by μ-CT (*n* = 8, LoxP mice and *n* = 9, *SIRT2*-KO^hep^ mice). **g**,**h**, Represented images of 3D restoration and quantification of trabecular BV/TV, Tb.N, Tb.Sp and Tb.Th of distal femurs of aged male LoxP and *SIRT2*-KO^hep^ mice (18 months of age), as measured by μ-CT (*n* = 10, LoxP mice and *n* = 12, *SIRT2*-KO^hep^ mice). **i**,**j**, Plasma CTX-1 in aged female and male LoxP and *SIRT2*-KO^hep^ mice was detected by ELISA. **k**,**m**, TRAP staining on paraffin-embedded femur sections in aged female and male LoxP and *SIRT2*-KO^hep^ mice (scale bar, 100 µm). **l**,**n**, Quantification of osteoclast surface/bone surface ratios (Oc.S/BS) is shown on the right. (**e**,**f**,**i**,**k**,**l** shows one technical replicate of eight (LoxP mice) or nine (*SIRT2*-KO^hep^ mice) biological replicates for each group. **g**,**h**,**j**,**m**,**n**, one technical replicate of 10 (LoxP mice) or 12 (*SIRT2*-KO^hep^ mice) biological replicates for each group). Data are presented as mean ± s.d., with biologically individual data points shown. *P* values were determined by unpaired two-tailed Mann–Whitney *U*-test (Tb.Th group of **f** and Tb.Sp group of **h**), unpaired two-tailed Student’s *t*-test with Welch’s correction (BV/TV group of **h**) or unpaired two-tailed Student’s *t*-test (others). Source data

**Extended Data Fig. 1:**
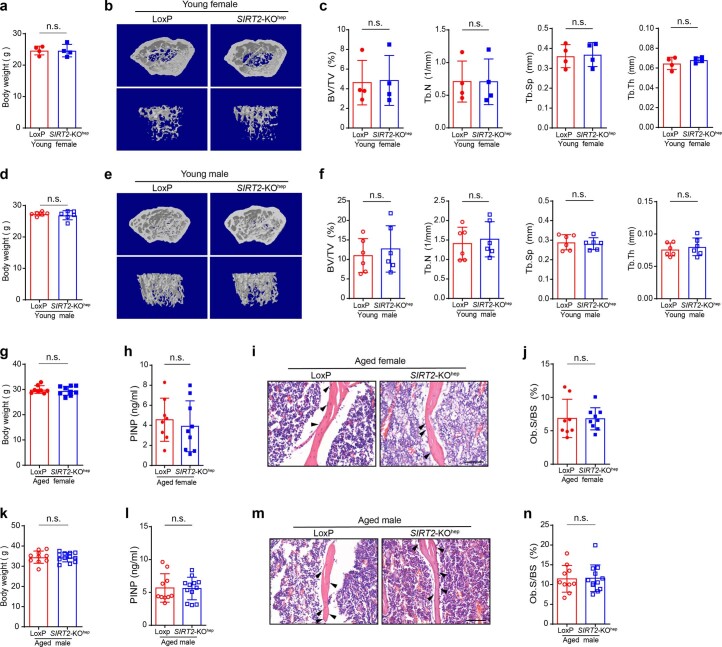
Young *SIRT2*-KO^hep^ mice have normal body weight, bone mass and aged *SIRT2*-KO^hep^ mice have normal body weight and osteoblastogenesis. Young LoxP and *SIRT2-*KO^hep^ mice in both sexes were compared (3 months of age). **a**, Body weight of the young female LoxP (n = 4) and *SIRT2-*KO^hep^ (n = 4) mice. **b,c**, Represented images of 3D restoration and quantification of trabecular BV/TV, Tb.N., Tb.Sp. and Tb.Th. of distal femur of the young female LoxP (n = 4) and *SIRT2-*KO^hep^ (n = 4) mice, as measured by micro-CT.(**a-c**. one technical replicate of 4 biological replicates for each group). **d**, Body weight of the young male LoxP (n = 6) and *SIRT2-*KO^hep^ (n = 6) mice. **e,f**, Represented images of 3D restoration and quantification of trabecular BV/TV, Tb.N., Tb.Sp. and Tb.Th. of distal femur of the young male LoxP (n = 6) and *SIRT2-*KO^hep^ (n = 6) mice, as measured by micro-CT.(**d-f**, one technical replicate of 6 biological replicates for each group). Aged LoxP and *SIRT2-*KO^hep^ mice in both sexes were compared (18 months of age). **g**, Body weight of the aged female LoxP (n = 8) and *SIRT2-*KO^hep^ (n = 9) mice. **h**, PINP in aged female LoxP (n = 8) and *SIRT2-*KO^hep^ (n = 9) mice were detected by ELISA. **k**, Body weight of the aged male LoxP (n = 10) and *SIRT2-*KO^hep^ (n = 12) mice. **l**, PINP in aged male LoxP (n = 10) and *SIRT2-*KO^hep^ (n = 12) mice. **i, m**, H&E staining on paraffin-embedded femur sections in aged female and male LoxP and *SIRT2-*KO^hep^ mice (scale bar, 100 µm; black arrowheads point to osteoblasts). **j, n**, Quantification of osteoblast surface/bone surface ratios (Ob.S/BS) are shown on the right (j, aged female LoxP mice, n = 8 and aged female *SIRT2-*KO^hep^mice,n = 9;n, aged male LoxP mice, n = 10 and aged male *SIRT2-*KO^hep^mice, n = 12). (**g-j**. one technical replicate of 8 (LoxP mice) or 9 (*SIRT2*-KO^hep^ mice) biological replicates for each group. **k-n**. one technical replicate of 10 (LoxP mice) or 12 (*SIRT2*-KO^hep^ mice) biological replicates for each group). Data are presented as mean ± SD. with biologically individual data points shown. *P* values are determined by unpaired two-tailed Student’s *t*-test (**a, c, d, f, g, h, j, k, l, n**). n.s., not significant. Source data

To investigate whether hepatic SIRT2 is potentially involved in aging-associated bone loss, hepatocyte SIRT2 expression in aged mice with osteoporosis was examined. Compared to young mice, SIRT2 protein expression was obviously increased in hepatocytes of both aged female and male mice with osteoporosis (Fig. 1a–d). To further identify the pathophysiological role of hepatocyte SIRT2 in bone homeostasis in vivo, we generated liver-specific SIRT2-knockout mice using a floxed SIRT2 mouse strain and an Alb-Cre line as previously described^20^. Compared to young *SIRT2*^flox/flox^Alb-Cre^−^ (LoxP) control littermates (3 months old), young *SIRT2*^flox/flox^Alb-Cre^+^ (*SIRT2*-KO^hep^) mice were phenotypically unremarkable, including healthy body weight and bone mass (Extended Data Fig. 1a–f). With aging, micro-computed tomography (μ-CT) analysis of distal femurs showed obvious bone loss and osteoporosis in both aged female and male mice (18 months old) (Fig. 1e,g). The aged *SIRT2*-KO^hep^ mice showed similar body weight as aged LoxP mice (Extended Data Fig. 1g,k). Notably, compared to aged LoxP mice, the aged *SIRT2*-KO^hep^ mice in both sexes exhibited markedly increased bone mass (Fig. 1e,g), shown by increased bone volume/tissue volume ratio (BV/TV), trabecular number (Tb.N) and decreased trabecular separation (Tb.Sp) (Fig. 1f,h). Trabecular thickness (Tb.Th) was not different between groups (Fig. 1f,h). These results suggest that genetic deletion of SIRT2 in hepatocyte significantly slows down bone loss and prevents senile osteoporosis in mice.

To examine whether SIRT2 deficiency in hepatocytes disrupted the dynamic balance between osteoclasts and osteoblasts, we measured the markers for bone resorption and bone formation. Compared to aged LoxP mice, there was an obviously decreased level of serum C-terminal telopeptide for type 1 collagen (CTX-1) in aged *SIRT2*-KO^hep^ mice, suggesting that *SIRT2*-KO^hep^ abolishes bone resorption and osteoclast activity (Fig. 1i,j). Moreover, lower number of TRAP-positive osteoclasts shown by TRAP staining on paraffin-embedded bone sections was observed on the surface of trabecular bone in female (Fig. 1k,l) and male (Fig. 1m,n) *SIRT2*-KO^hep^ aged mice compared to the LoxP group, which is consistent with the CTX-1 serological evidence. Meanwhile, we noted a similar level of serum procollagen type 1 N-propeptide (P1NP) (Extended Data Fig. 1h,l) and number of osteoblasts (Extended Data Fig. 1i,j,m,n) between aged groups, which suggests similar bone formation and osteoblast activity. In sum, these results provide evidence that *SIRT2*-KO^hep^ prevents aging-associated osteoporosis by suppressing osteoclastogenesis.

### SIRT2^−/−^ hepatocyte-derived sEVs abolish osteoclastogenesis

**Fig. 2:**
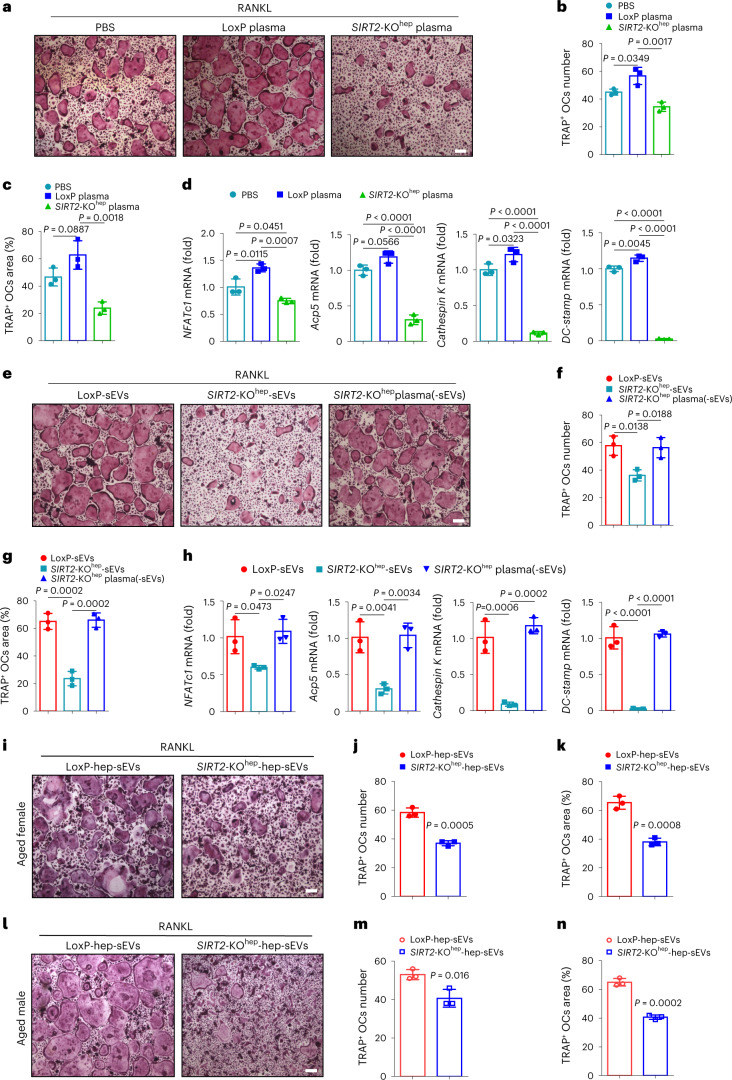
Aged *SIRT2*^−/−^ hepatocyte-derived sEVs inhibit osteoclastogenesis. BMDMs were isolated and cultured with murine M-CSF and RANKL stimulation for 7 d to generate osteoclasts, combined with the corresponding treatments. **a**, Representative TRAP staining images of osteoclasts administered with the plasma (100 µl) of aged LoxP or *SIRT2*-KO^hep^ male mice (LoxP plasma or *SIRT2*-KO^hep^ plasma) (scale bar, 200 µm). **b**,**c**, Number and area of multi-nucleated TRAP^+^ cells with indicated treatment were measured. OC, osteoclast. **d**, Osteoclast-specific genes *NFATc1*, *Acp5*, *cathespin K* and *DC-STAMP* mRNA levels in LoxP-plasma- or *SIRT2*-KO^hep^-plasma-treated osteoclasts were measured by real-time PCR; *n* = 3 biologically independent experiments (**a**–**d**). **e**, Representative TRAP staining images of osteoclasts treated with sEVs (derived from 100 µl plasma) derived from LoxP plasma or *SIRT2*-KO^hep^ plasma (LoxP-sEVs or *SIRT2*-KO^hep^-sEVs), as well as with *SIRT2*-KO^hep^-plasma-depleted sEVs (*SIRT2*-KO^hep^-plasma(-sEVs), 100 µl) (scale bar, 200 µm). **f**,**g**, Number and area of multi-nucleated TRAP^+^ cells with indicated treatment were measured. **h**, The mRNA expression of osteoclast-specific genes in the corresponding treated osteoclasts was measured by real-time PCR; *n* = 3 biologically independent experiments (**e**–**h**). **i**,**l**, Representative TRAP staining images of osteoclasts treated with sEVs (4 µg ml^−1^) derived from the medium of the primary hepatocytes of aged female (**i**) and male (**l**) LoxP mice or *SIRT2*-KO^hep^ mice (LoxP-hep-sEVs or *SIRT2*-KO^hep^-hep-sEVs) (scale bar, 200 µm). **j**,**k**,**m**,**n**, Number and area of multi-nucleated TRAP^+^ cells of female (**j**,**k**) and male (**m**,**n**) mice with indicated treatment were measured, respectively; *n* = 3 biologically independent experiments (**i**–**n**). Data are presented as mean ± s.d., with biologically individual data points shown. *P* values were determined by one-way analysis of variance (ANOVA) followed by Tukey’s test (**b**–**d**,**f**–**h**) and an unpaired two-tailed Student’s *t*-test (**j**,**k**,**m**,**n**). Source data

**Extended Data Fig. 2:**
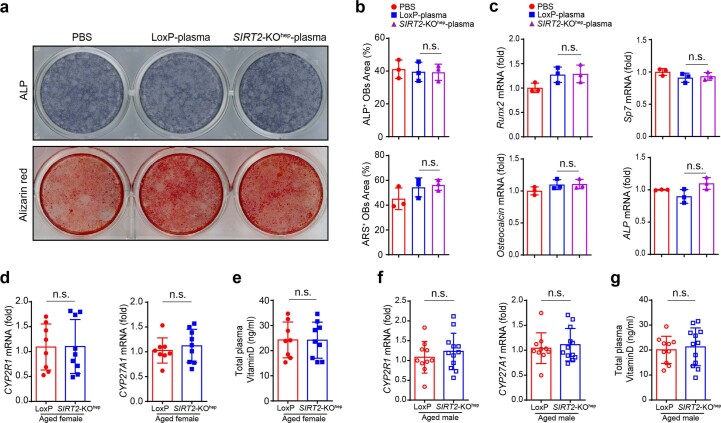
Liver-specific SIRT2 deficiency has no effects on osteogenic differentiation and Vitamin D synthesis. **a**, Alkaline phosphatase (ALP) and alizarin red (ARS) staining after osteogenesis from bone marrow-derived mesenchymal cells (BM-MSCs) treated with LoxP-plasma and *SIRT2-*KO^hep^-plasma for 7 days and 14 days respectively. **b**, Area of ALP staining and ARS staining positive osteoblasts was analyzed by ImageJ and quantification analysis is shown. **c**, The expression of osteogenic genes Runx2, Sp7, osteocalcin and ALP in osteoblasts as measured by real-time PCR. n = 3 biologically independent experiments. **d**, *CYP2R1* and *CYP27A1* mRNA level in livers of aged female LoxP and *SIRT2-*KO^hep^ mice (18 months of age) were analyzed by real-time PCR (aged female LoxP mice, n = 8 and aged female *SIRT2-*KO^hep^ mice, n = 9). **e**, Total plasma concentration of Vitamin D of aged female LoxP and *SIRT2-*KO^hep^ mice were detected by ELISA. **f**, *CYP2R1* and *CYP27A1* mRNA level in livers of aged male LoxP and *SIRT2-*KO^hep^ mice (18 months of age) were analyzed by real-time PCR (aged male LoxP mice, n = 10 and aged male *SIRT2-*KO^hep^ mice, n = 12). **g**, Total plasma concentration of Vitamin D of aged male LoxP and *SIRT2-*KO^hep^ mice were detected by ELISA. (**d,e**. one technical replicate of 8 (LoxP mice) or 9 (*SIRT2*-KO^hep^ mice) biological replicates for each group. **f, g**. one technical replicate of 10 (LoxP mice) or 12 (*SIRT2*-KO^hep^ mice) biological replicates for each group). Data are presented as mean ± SD. with biologically individual data points shown. *P* values were determined by one-way ANOVA followed by Tukey’s test (**b, c**). *P* values are determined by unpaired two-tailed Student’s *t*-test (**d-g**). n.s., not significant. Source data

To investigate the molecular mechanisms underlying liver–bone communication by SIRT2 in senile osteoporosis, we first verified the effect of plasma from two aged mouse groups on osteoblast and osteoclast differentiation. For osteoblast differentiation, bone-marrow-derived mesenchymal stem cells (BM-MSCs) from C57BL/6 mice were isolated and cultured with the plasma of aged LoxP or *SIRT2*-KO^hep^ male mice (LoxP plasma or *SIRT2*-KO^hep^ plasma) combined with murine osteogenic differentiation medium to induce osteogenic differentiation. After induction, alkaline phosphatase (ALP) staining and Alizarin red staining (ARS) showed that *SIRT2*-KO^hep^ plasma had a similar effect on the potential of osteogenic differentiation as LoxP plasma, as evidenced by the similar ALP- and ARS-positive osteoblast area (Extended Data Fig. 2a,b). Consistently, the expression of osteogenesis-specific genes *Runx2*, *ALP*, *SP7* and *osteocalcin* was not changed in BM-MSCs treated with *SIRT2*-KO^hep^ plasma and LoxP plasma (Extended Data Fig. 2c), suggesting that hepatocyte SIRT2 had no effect on osteogenic differentiation. Meanwhile, to determine whether the *SIRT2*-KO^hep^-enhanced bone mass is due to decreased bone resorption, we isolated BMDMs and induced osteoclast differentiation. BMDMs were cultured with LoxP plasma or *SIRT2*-KO^hep^ plasma combined with murine M-CSF and RANKL stimulation for 7 d to generate osteoclasts, subsequently followed by TRAP staining. While LoxP plasma has potential pro-osteoclastic activity, *SIRT2*-KO^hep^ plasma obviously suppressed RANKL-induced osteoclastogenesis, as characterized by lower TRAP-positive osteoclast numbers with smaller volume (Fig. 2a–c). Moreover, *SIRT2*-KO^hep^ plasma treatment also inhibited RANKL-induced expression of NFATc1, Acp5, cathepsin K and DC-STAMP, any of which is a critical marker of osteoclastogenesis (Fig. 2d). Altogether, plasma mediates the inhibitory effects of *SIRT2*-KO^hep^ on osteoclastogenesis.

**Extended Data Fig. 3:**
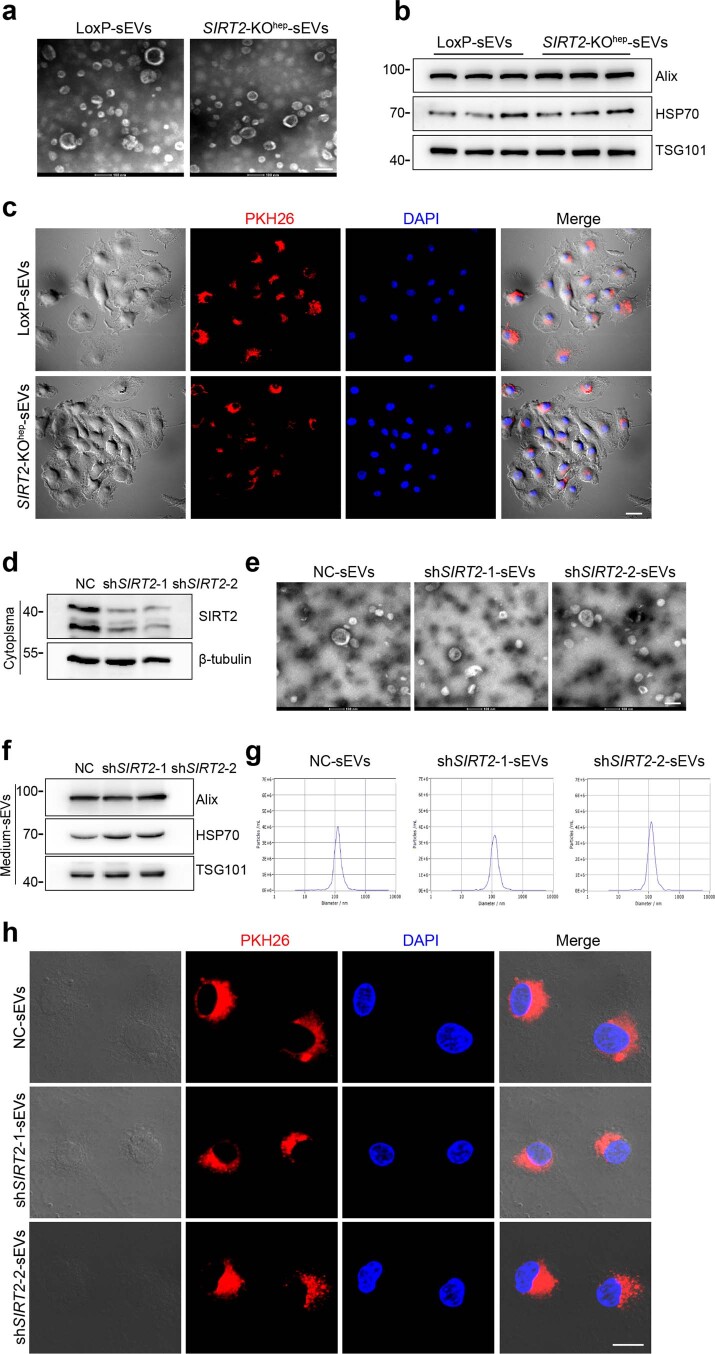
Identification of murine plasma-derived and AML12 hepatocytes supernatant-derived sEVs. **a**, Electron microscopy images of sEVs isolated from plasma of aged LoxP and *SIRT2-*KO^hep^ mice (18 months) (scale bar, 100 nm). n = 2 mice. **b**, Western blot analysis of sEVs protein markers TSG101, HSP70, ALIX in plasma-derived sEVs. n = 3 mice. **c**, Representative immunofluorescence images show the internalization of PKH26-labeled plasma-derived sEVs (4 μg/ml) (red) in BMDMs (scale bar, 20 µm). n = 2 biologically independent experiments. **d**, Western blot analysis of SIRT2 expression in the cytoplasm of SIRT2-knockdown AML12 cells. **e**, Electron microscopy images of sEVs isolated from AML12 hepatocytes supernatant-derived sEVs (scale bar, 100 nm). **f**, Western blot analysis of sEVs protein markers TSG101, HSP70, ALIX in the sEVs derived from the supernatant control (NC) and SIRT2-knockdown (SIRT2 shRNA) AML12 cells. **g**, Nanoparticle tracking analysis (NTA) of the sEVs derived from the supernatant NC and SIRT2 shRNA AML12 cells and isolated by ultracentrifugation. **h**, Representative immunofluorescence images show the internalization of PKH26-labeled sEVs (4 μg/ml) (red) in BMDMs (scale bar, 20 µm). (d-h, n = 1 biologically independent experiment). Source data

Next, to explore which components in *SIRT2*-KO^hep^ plasma suppressed osteoclastogenesis, we first examined the VitD metabolism in the two aged groups; however, there was no difference in the concentration of plasma total VitD and the expression of hepatic CYP2R1 and CYP27A1 between the aged LoxP and *SIRT2*-KO^hep^ group in both sexes (Extended Data Fig. 2d–g), suggesting that the suppression of osteoclastogenesis by *SIRT2*-KO^hep^ plasma is independent of VitD synthesis. To further investigate whether sEVs are required for the protection of *SIRT2*-KO^hep^ against osteoclastogenesis, we isolated sEVs from LoxP plasma or *SIRT2*-KO^hep^ plasma (LoxP-sEVs or *SIRT2*-KO^hep^-sEVs) and labeled them with PKH26 (a fluorescent lipophilic dye) before co-culturing with BMDMs. Transmission electron microscopy analysis and western blot confirmed the purity and characteristics of the isolated sEVs (Extended Data Fig. 3a,b). No significant differences in particle shapes and numbers were observed between LoxP-sEVs and *SIRT2*-KO^hep^-sEVs. After 10 h of co-culture, sEVs may be internalized and mainly found in the cytoplasm in BMDMs (Extended Data Fig. 3c). Notably, the results showed a lower number and smaller volume of TRAP-positive osteoclasts from BMDMs treated with *SIRT2*-KO^hep^-sEVs compared to LoxP-sEVs (Fig. 2e–g); however, the *SIRT2*-KO^hep^ plasma had no inhibitive effects on osteoclastogenesis after removing sEVs (Fig. 2e–g). Consistently, real-time PCR analysis also confirmed that osteoclast-specific genes were downregulated by *SIRT2*-KO^hep^-sEVs (Fig. 2h).

**Extended Data Fig. 4:**
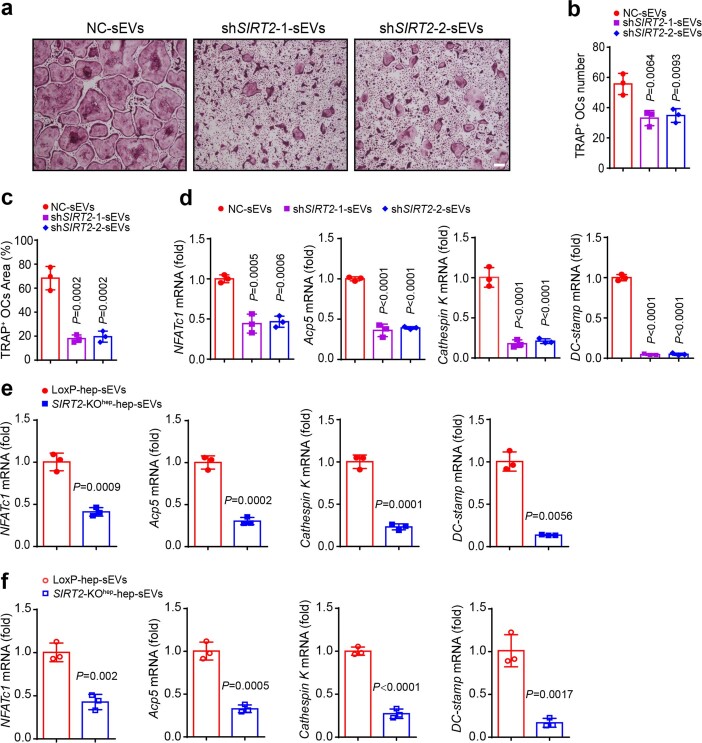
*SIRT2*-knockdown AML12 hepatocytes and aged *SIRT2*^-/-^ hepatocyte-derived sEVs inhibit osteoclastogenesis. **a**, Representative TRAP staining images of osteoclasts treated with the sEVs (4 μg/ml) derived from control AML12 hepatocytes (NC-sEVs) or *SIRT2*-knockdown AML12 hepatocytes (sh*SIRT2*-1-sEVs or sh*SIRT2*-2-sEVs) (scale bar, 200 µm). **b,c**, Number and area of multi-nucleated TRAP^+^ cells with indicated treatment were measured. **d**, The mRNA expression of osteoclast-specific genes in the corresponding treated osteoclasts was measured by real-time PCR. (a-d, n = 3 biologically independent experiments). **e,f**, The osteoclast-specific genes *NFATc1, Acp5, Cathespin K* and *DC-stamp* mRNA expression in osteoclasts treated with aged female(**e**) or male (**f**) LoxP-hep-sEVs or *SIRT2*-KO^hep^-hep-sEVs measured by real-time PCR. n = 3 biologically independent experiments. Data are presented as mean ± SD. with biologically individual data points shown. *P* values are determined by one-way ANOVA followed by Tukey’s test (**b-d**), unpaired two-tailed Student’s *t*-test with Welch’s correction (*DC-stamp* group of **e**) and unpaired two-tailed Student’s*t*-test (**e**, others**; f**). Source data

To further verify whether sEVs directly derived from hepatocytes, not indirectly from other types of cells in liver, regulate osteoclast differentiation, we created a *SIRT2*-knockdown cell line in AML12 murine hepatocytes and isolated primary hepatocytes from aged LoxP and *SIRT2*-KO^hep^ mice. The effect of their sEVs on osteoclastogenesis was assessed. The shape, size, proteins and internalization of sEVs derived from the supernatant of AML12 hepatocytes were similar with those of plasma-derived sEVs (Extended Data Fig. 3d–h). Furthermore, sEVs derived from the medium of *SIRT2*-knockdown AML12 hepatocytes (sh*SIRT2*-sEVs) markedly reduced osteoclast numbers, size and expression of osteoclast-specific genes compared to sEVs derived from control AML12 hepatocytes (NC-sEVs) treatment (Extended Data Fig. 4a–d). Moreover, medium-derived sEVs of the primary hepatocytes of aged *SIRT2*-KO^hep^ (*SIRT2*-KO^hep^-hep-sEVs) markedly suppressed osteoclastogenesis compared to sEVs from primary hepatocytes of aged LoxP mice (LoxP-hep-sEVs) (Fig. 2i–n and Extended Data Fig. 4e,f). In sum, these data provide compelling evidence that SIRT2^−**/**−^ hepatocytes inhibit osteoclastogenesis through an sEV pathway, suggesting the potential involvement of sEVs in inter-organ crosstalk between the liver and bone in osteoporosis.

### SIRT2^−**/**−^ hepatocyte-derived sEVs contain higher level of LRG1 protein via increasing acetylation of H4K16

**Fig. 3:**
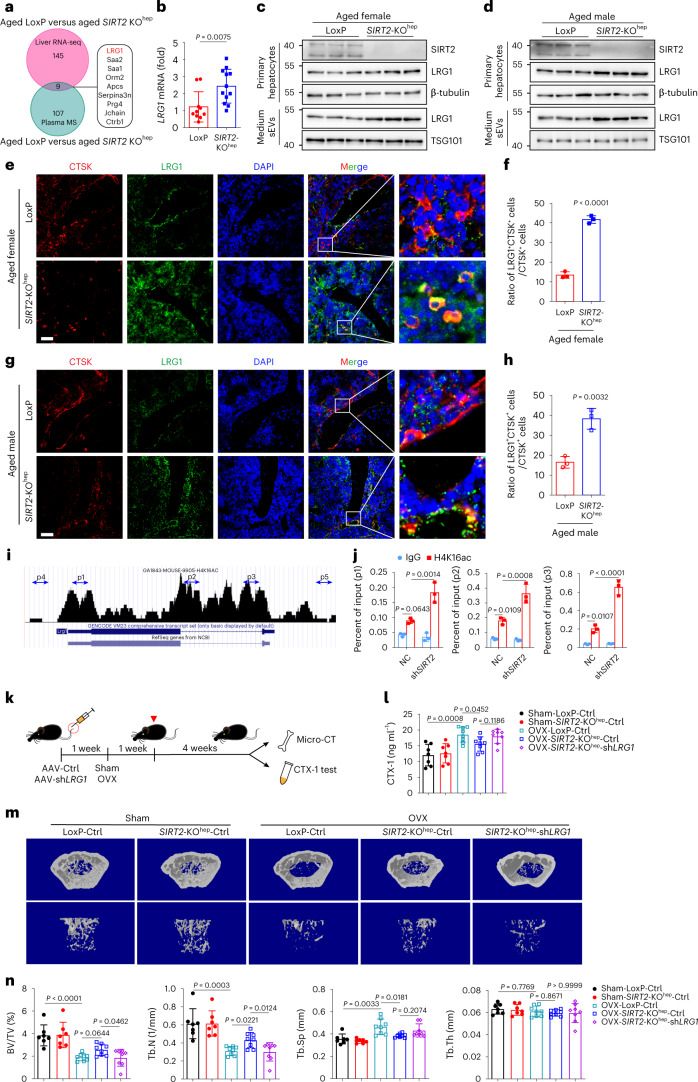
*SIRT2*-KO^hep^ prevents OVX-induced bone loss through upregulating LRG1 expression in hepatocytes. **a**, Venn diagram showing the overlap numbers of *SIRT2*-KO^hep^-regulated plasma proteins by mass spectra (MS) and *SIRT2*-KO^hep^-regulated hepatic mRNAs by RNA-seq in aged mice (18 months of age). **b**, *LRG1* mRNA expression in the livers of aged LoxP and *SIRT2*-KO^hep^ mice measured by real-time PCR. One technical replicate of 10 (LoxP mice) or 12 (*SIRT2*-KO^hep^ mice) biological replicates for each group. **c**,**d**, Western blot analysis of LRG1 protein expression in the primary hepatocytes and supernatant-derived sEVs of aged female (**c**) and male (**d**) LoxP and *SIRT2*-KO^hep^ mice; *n* = 3 mice, one technical replicate of three biological replicates for each group. **e**,**g**, Represented in situ immunofluorescence images of murine femurs in aged female (**e**) and male (**g**) LoxP and *SIRT2*-KO^hep^ mice (18 months of age) (scale bar, 50 µm). DAPI, 4,6-diamidino-2-phenylindole. **f**,**h**, Quantitation of ratio of LRG1 and CTSK double-positive area to CTSK-positive area on bone sections of the aged female (**f**) or male (**h**) LoxP (*n* = 3) and *SIRT2-*KO^hep^ (*n* = 3) mice, was measured by ImageJ. (**e**–**h**, one technical replicate of three biological replicates for each group). **i**, Schematic view of enrichment of H4K16ac on LRG1 promoter region from ChIP-seq data from Cistrome DB Toolkit. **j**, ChIP analysis showing enrichment of H4K16ac at the LRG1 proximal promoter region in NC and shSIRT2-AML12 hepatocytes using the primers p1, p2 and p3; *n* = 3 biologically independent experiments. **k**, The experimental procedure for hepatocyte-specific *LRG1* knockdown by AAV8 virus. LoxP and *SIRT2-*KO^hep^ mice (12 weeks of age) were given tail injections of 2 × 10^11^ viral particles of either AAV8-sh*LRG1* or Ctrl vector 7 d before OVX to maximize the viral expression and knockdown efficiency. The real-time observation of gene expression was performed by BLI 14 d after viral injection. Mice were killed 5 weeks after OVX for CTX-1 and bone mass test. **l**, Plasma CTX-1 was detected by ELISA. **m**,**n**, Represented images of 3D restoration and quantification of trabecular BV/TV, Tb.N, Tb.Sp and Tb.Th of distal femurs of the indicated group mice, as measured by μ-CT (*n* = 7, Sham-LoxP-Ctrl mice; *n* = 7, Sham-*SIRT2*-KO^hep^-Ctrl mice; *n* = 8, OVX-LoxP-Ctrl mice; *n* = 8, OVX-*SIRT2*-KO^hep^-Ctrl mice and *n* = 8, OVX-*SIRT2*-KO^hep^*-*sh*LRG1* mice). (**l**–**n**, one technical replicate of seven (Sham-LoxP-Ctrl mice); seven (Sham-*SIRT2*-KO^hep^-Ctrl mice); eight (OVX-LoxP-Ctrl mice); eight (OVX-*SIRT2*-KO^hep^-Ctrl mice) and eight (OVX-*SIRT2*-KO^hep^*-*sh*LRG1* mice) biological replicates for each group). Data are presented as mean ± s.d., with biologically individual data points shown. *P* values were determined by unpaired two-tailed Student’s *t*-test (**b**,**f**,**h**), one-way ANOVA followed by Tukey’s test (**l**,**n**) and two-way ANOVA followed by Tukey’s test (**j**). Source data

**Extended Data Fig. 5:**
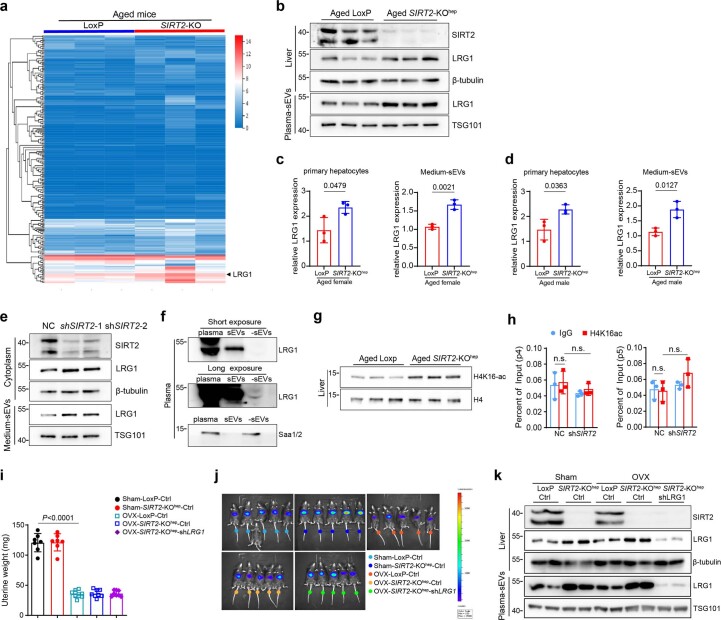
*SIRT2*-KO^hep^ prevents against OVX-induced bone loss through upregulating LRG1 expression in hepatocytes and sEVs. **a**, Heatmap of differentially expressed genes identified by RNA-seq using liver tissues from aged LoxP (n = 3) and *SIRT2*-KO^hep^ mice (n = 3). **b**, Western blot analysis of LRG1 protein expression in the livers and plasma sEVs of aged LoxP and *SIRT2*-KO^hep^ mice. n = 3 mice.one technical replicate of 3 biological replicates for each group. **c, d**, Western blot density analyzed by ImageJ and quantification analysis was shown. n = 3 mice, one technical replicate of 3 biological replicates for each group. **e**, Western blot analysis of LRG1 protein expression in the cytoplasm and supernatant-derived sEVs of NC and shSIRT2-AML12 hepatocytes. **f**, Western blot analysis of respective LRG1, Saa1/2 protein level in plasma, plasma sEVs and plasma removed sEVs (-sEVs). **g**, Western blot analysis of H4K16ac protein levels in the liver of aged LoxP and *SIRT2*-KO^hep^ mice. n = 3 mice.one technical replicate of 3 biological replicates for each group. **h**,ChIP analysis showing the enrichment of H4K16ac at the LRG1 proximal promoter region in NC and shSIRT2-AML12 hepatocytes using the primers p4 and p5, which are located at the distant upstream or downstream sites of LRG1 promoter and used as negative control. n = 3 biologically independent experiments. **i**, Uterine weight in each group after OVX or sham operation (Sham-LoxP-Ctrl mice: n = 7; Sham-*SIRT2*-KO^hep^-Ctrl mice: n = 7; OVX-LoxP-Ctrl mice: n = 8; OVX-*SIRT2*-KO^hep^-Ctrl mice: n = 8; and OVX-*SIRT2*-KO^hep^*-*sh*LRG1* mice: n = 8). (one technical replicate of 7 (Sham-LoxP-Ctrl mice); 7 (Sham-*SIRT2*-KO^hep^-Ctrl mice); 8 (OVX-LoxP-Ctrl mice); 8 (OVX-*SIRT2*-KO^hep^-Ctrl mice) and 8 (OVX-*SIRT2*-KO^hep^*-*sh*LRG1* mice) biological replicates for each group). **j**, The real-time observation of AAV8 liver-specific expression in each group was performed by BLI at 14th day after viral injection. **k**, Western blot analysis of LRG1 protein levels in the liver and plasma sEVs of each group. one technical replicate of 2 biological replicates for each group. Data are presented as mean ± SD. with biologically individual data points shown. *P* values are determined by unpaired two-tailed Student’s *t*-test (**c, d, i**), two-way ANOVA followed by Tukey’s test (**h**). n.s., not significant. Source data

Given that sEVs can transmit molecular cargos into recipient cells, we presumed that SIRT2^−**/**−^ hepatocyte-sEVs might deliver certain proteins to BMDMs to inhibit their differentiation to osteoclasts. To address this hypothesis, we undertook a global comparison of the plasma proteins of aged LoxP and *SIRT2*-KO^hep^ mice by mass spectrometry, together with mRNA expression profiling in the liver by RNA sequencing (RNA-seq) analysis (Fig. 3a and Extended Data Fig. 5a). Among the nine shared regulated proteins, LRG1 expression in the liver and plasma was the most significantly increased in *SIRT2*-KO^hep^ mice (Fig. 3a). Real-time PCR and western blot analysis confirmed this observation (Fig. 3b and Extended Data Fig. 5b). LRG1, a secreted glycoprotein, is a highly conserved member of the leucine-rich repeat family of proteins, many of which are involved in protein–protein interactions and signaling^22^. Western blot analyses showed the vast majority of LRG1 protein in plasma was located in sEVs (Extended Data Fig. 5f). Furthermore, the level of LRG1 protein in the *SIRT2*-KO^hep^-sEVs was higher than that in LoxP-sEVs (Extended Data Fig. 5b). Consistent with in vivo results, *SIRT2* knockout obviously enhanced LRG1 protein expression both in the cytoplasm and medium-derived sEVs of primary hepatocytes (Fig. 3c,d and Extended Data Fig. 5c,d) and AML12 hepatocytes (Extended Data Fig. 5e). Further, in situ immunofluorescence analysis of murine femurs showed that the number of LRG1-expressing osteoclast progenitors (CTSK^+^ cells) in aged *SIRT2*-KO^hep^ mice was significantly higher than that of aged LoxP group (Fig. 3e,g). Moreover, LRG1 protein level was significantly increased in the osteoclast progenitors of aged *SIRT2*-KO^hep^ mice (Fig. 3e–h). All of these results suggested that hepatic SIRT2 might regulate LRG1 protein levels in osteoclast progenitors via sEV transfer.

It has been previously reported that SIRT2 has a strong preference for acetylation of histone 4 lysine 16 (H4K16ac) in its deacetylation activity^23^. H4K16ac activates gene transcription by influencing both chromatin structure and interplay with nonhistone proteins^23^. To explore whether SIRT2 regulates LRG1 transcription via deacetylating H4K16ac, we analyzed the previously reported chromatin immunoprecipitation (ChIP)-seq data^24^ and predicted three regions in the LRG1 promoter containing high acetylation levels on H4K16 (Fig. 3i). Three primers (p1, p2 and p3) encompassing all the regions were designed and the following ChIP result revealed significant higher enrichment of H4K16ac in the three regions in sh*SIRT2-*AML12 hepatocytes compared to control hepatocytes (NCs) (Fig. 3j and Extended Data Fig. 5g), whereas no obvious changes were detected in the distant upstream or downstream sites (Extended Data Fig. 5h). The results indicated that SIRT2 inhibits sEV-LRG1 protein expression through deacetylation of H4K16 in hepatocytes.

### *SIRT2*-KO^hep^ prevents OVX-induced bone loss by upregulating hepatic LRG1 expression

**Extended Data Fig. 6:**
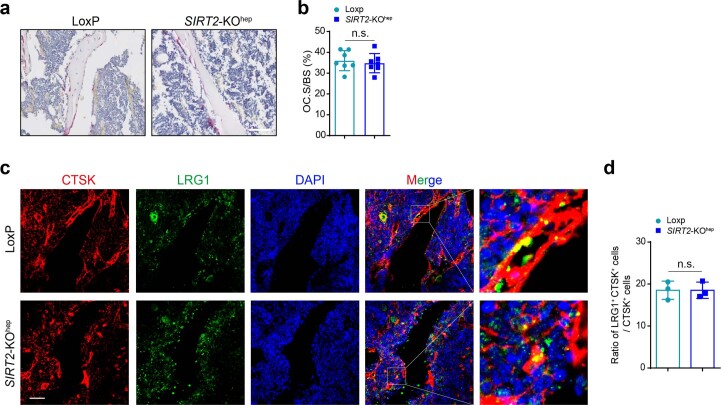
*SIRT2*-KO^hep^ has no effects on number of osteoclasts and LRG1 protein level in the osteoclast progenitors in young mice. **a, b**, TRAP staining on paraffin-embedded femur sections in young LoxP (n = 7) and *SIRT2-*KO^hep^ (n = 7) mice (3 months of age) (scale bar, 100 µm) and quantification of Oc.S/BS is shown on the right. One technical replicate of 7 biological replicates for each group. **c,d**, Represented in situ immunofluorescence images of murine femurs in young mice (scale bar, 50 µm)and quantitation of ratio of LRG1 and CTSK double positive area to CTSK positive area on bone sections of the young LoxP (n = 3) and *SIRT2-*KO^hep^ (n = 3) mice. One technical replicate of 3 biological replicates for each group. Data are presented as mean ± SD. with biologically individual data points shown. *P* values were determined by unpaired two-tailed Student’s *t-test* (**b, d**). Source data

As estrogen deficiency increases osteoclast formation, we performed ovariectomy (OVX) in female mice to simulate the estrogen loss in postmenopausal women (Fig. 3k and Extended Data Fig. 5i). Though OVX has no effect on hepatic SIRT2 and LRG1 expression, hepatic SIRT2 deficiency upregulates LRG1 expression both in hepatocytes and sEVs in OVX models (Extended Data Fig. 5k). To find out whether upregulation of hepatic LRG1 is the underlying mechanism for the protective effect of *SIRT2*-KO^hep^ in osteoclastogenesis, LoxP and *SIRT2*-KO^hep^ mice were given injections of either a recombinant adeno-associated viral vector serotype 8 (AAV8) expressing sh*LRG1* under the control of the hepatocyte-specific thyroxin-binding globulin (TBG) promoter (AAV8-sh*LRG1*) or control vector AAV8-NC (Ctrl) (Extended Data Fig. 5j). The AAV8 vector contained a luciferase reporter gene for real-time observation of gene expression by bioluminescence imaging (BLI). The mice were treated with 2 × 10^11^ viral particles via tail vein injection 7 d before OVX to maximize viral expression and knockdown efficiency (Extended Data Fig. 5j). In accordance with BLI results (Extended Data Fig. 5j), western blot analysis confirmed the knockdown of LRG1 both in hepatocyte and plasma sEVs in the AAV8-sh*LRG1* group (Extended Data Fig. 5k). As revealed by μ-CT and plasma CTX-1, hepatocyte-specific SIRT2 deficiency prevented OVX-induced osteoclastogenesis, bone loss (lower BV/TV) and poorly organized trabecular architecture (lower Tb.N and higher Tb.Sp) (Fig. 3l–n). Moreover, the knockdown of hepatocyte LRG1 completely reversed the osteoclastogenesis and bone phenotype in the *SIRT2*-KO^hep^-OVX mice group (Fig. 3l–n). The number of osteoclasts (Extended Data Fig. 6a,b) and LRG1-expressing osteoclast progenitors (Extended Data Fig. 6c,d) in young *SIRT2*-KO^hep^ mice was similar to that of young LoxP mice, although young *SIRT2*-KO^hep^ mice had higher LRG1 expression in the liver and plasma (Extended Data Fig. 5k). These results indicate that the alleviated bone loss and osteoporosis in *SIRT2*-KO^hep^-OVX mice is, to a large extent, a consequence of upregulating LRG1 expression in hepatocytes.

### LRG1 is the cargo of hepatocyte-derived sEVs to mediate the protection of *SIRT2*-KO^hep^ against osteoclastogenesis and bone loss

**Fig. 4:**
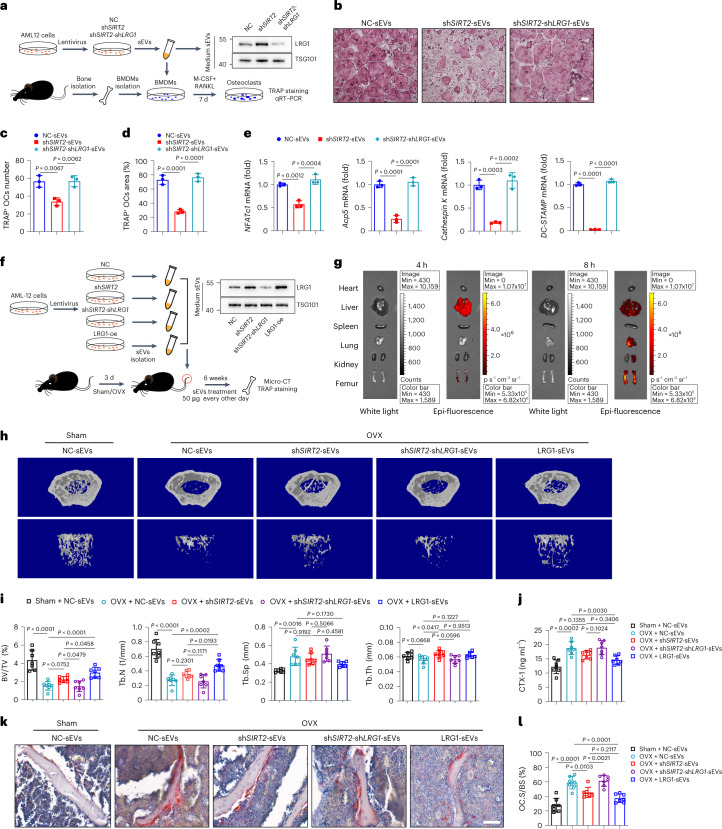
Hepatocyte-derived sEV-LRG1 mediates the protection of *SIRT2*-KO^hep^ against osteoclastogenesis and bone loss. **a**, Schema of BMDM treatment with sEVs. The sEVs were purified from the supernatant of sh*SIRT2*-AML12 cells infected with Ctrl or sh*LRG1* lentiviral vectors. sEV-LRG1 protein expression was analyzed by western blot. The isolated primary BMDMs were co-cultured with each set of transduced sEVs (4 µg ml^−1^) and murine M-CSF/RANKL stimulation for 7 d to generate osteoclasts and followed TRAP staining and real-time PCR test. **b**, TRAP staining of osteoclasts treated with NC-sEVs or sh*SIRT2*-sEVs or sh*SIRT2*-sh*LRG1*-sEVs (scale bar, 200 µm). **c**,**d**, Number and area of multi-nucleated TRAP^+^ cells with indicated treatment were measured. **e**, The mRNA expression of osteoclast-specific genes in the corresponding treated osteoclasts was measured by real-time PCR. (**b**–**e**, *n* = 3 biologically independent experiments). **f**, The experimental procedure for sEVs treatment in vivo. C57BL/6J mice were consecutively intravenously injected with the NC-sEVs, sh*SIRT2*-sEVs, sh*SIRT2*-sh*LRG1-*sEVs and LRG1-sEVs (50 µg per mouse, every other day) 3 d after OVX. Micro-CT and TRAP staining were performed 6 weeks after the first injection. **g**, Representative biophotonic images of the tissue distribution of fluorescence signal in mice at 4 and 8 h after intravenous injection of PKH26-labeled sEVs isolated from the supernatant of AML12 cells. **h**,**i**, Represented images of 3D restoration (**h**) and quantification of trabecular BV/TV, Tb.N, Tb.Sp and Tb.Th of distal femurs of the indicated group mice (**i**), as measured by μ-CT (*n* = 7, sham + NC-sEVs; *n* = 7, OVX + NC-sEVs; *n* = 7, OVX + sh*SIRT2*-sEVs; *n* = 7, OVX + sh*SIRT2*-sh*LRG1*-sEVs and *n* = 7, OVX + LRG1-sEVs). **j**, Plasma CTX-1 in each group was detected by ELISA. **k**, TRAP staining on paraffin-embedded femur sections in each group after corresponding sEV treatment (scale bar, 100 µm). **l**, Quantification of Oc.S/BS is shown on the right; (**h**–**l**, one technical replicate of seven biological replicates for each group). Data are presented as mean ± s.d., with biologically individual data points shown. *P* values were determined by one-way ANOVA followed by Tukey’s test (**c**–**e**,**i**,**j**,**l**). Source data

**Extended Data Fig. 7:**
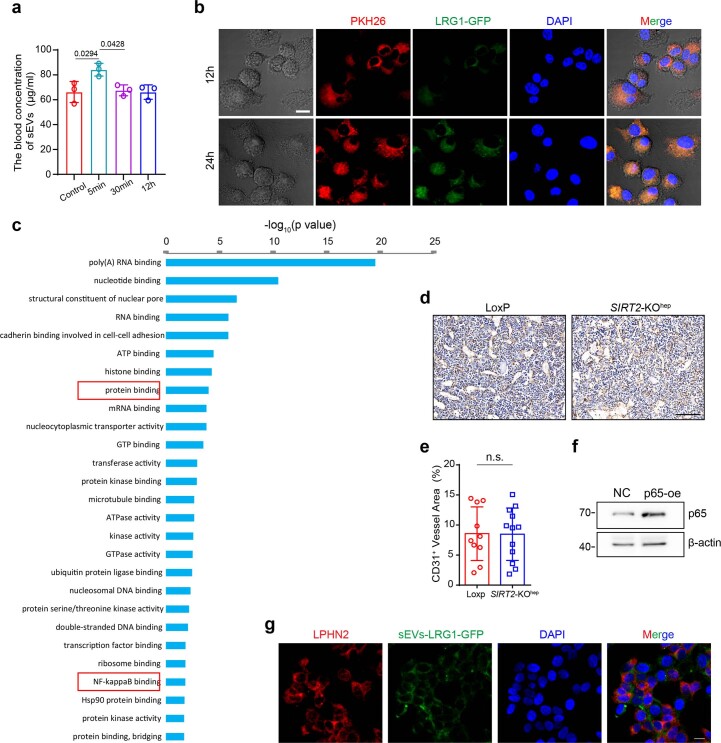
Hepatocyte-derived sEV-LRG1 suppresses osteoclast differentiation by inhibiting NF-κB p65 nuclear translocation, independently of angiogenesis. **a**, The blood concentration of sEVs at different time points after tail vein injection of sEVs. n = 3 mice. One technical replicate of 3 biological replicates. **b**, Immunofluorescence analysis of the distribution of sEV-LRG1-GFP (green) in RANKL-induced BMDMs at 12 h and 24 h after supplementing sEVs labeled with PKH26 (red) in supernatant (scale bar, 20 µm). **c**, Enrichment of signaling pathway of sEV-LRG1 binding proteins in DAVID Bioinformatics database. **d**, IHC detection of CD31 in the paraffin-embedded bone section of distal femur of aged LoxP and *SIRT2*-KO^hep^ mice (scale bar, 100μm). **e**, Quantification of CD31 positive vessels area (aged LoxP mice: n = 10 and aged *SIRT2*-KO^hep^ mice: n = 12). One technical replicate of 10 (LoxP mice) or 12 (*SIRT2*-KO^hep^ mice) biological replicates for each group. **f**, Western blot analysis of p65 protein levels in the RAW 264.7 cells overexpressed p65. n = 3 biologically independent experiments. **g**, HEK293T cells transfected with LPHN2 plasmid were treated with LRG1-sEVs (4 μg/ml) and then immunofluorescence colocalization analysis of sEV-LRG1 and LPNH2 was shown (scale bar, 20 µm). n = 2 biologically independent experiments. Data are presented as mean ± SD. with biologically individual data points shown. *P* values are determined by one-way ANOVA followed by Tukey’s test (**a**) and unpaired two-tailed Student’s *t*-test (**e**). n.s., not significant. Source data

We continued to verify the connection between LRG1 in sEVs (sEV-LRG1) and the protective role of *SIRT2*-KO^hep^ in osteoporosis. The sEVs were purified from the supernatant of sh*SIRT2*-AML12 cells infected with an empty vector (Ctrl) or sh*LRG1* lentiviral vectors. Then, BMDMs were co-cultured with each set of transduced sEVs and murine M-CSF/RANKL stimulation for 7 d to generate osteoclasts (Fig. 4a). Compared to *SIRT2*-knockdown AML12 cell-derived sEVs (sh*SIRT2*-sEVs), *SIRT2* and *LRG1* double-knockdown AML12 cell-derived sEVs (sh*SIRT2*-sh*LRG1*-sEVs) resulted in enhanced osteoclastogenesis, as indicated by the greater number and size of TRAP-positive osteoclasts from BMDMs (Fig. 4b–d) and the increased expression of osteoclast-specific genes (Fig. 4e). These data suggest that SIRT2-regulated sEV-LRG1 is directly linked to osteoclastogenesis. To further verify the mechanism and the therapeutic potential of sEV-LRG1 in osteoporosis in vivo, we consecutively intravenously injected the mice with control-sEVs (NC-sEVs), sh*SIRT2*-sEVs and sh*SIRT2*-sh*LRG1-*sEVs (50 µg per mouse, every other day) 3 d after OVX (Fig. 4f). At first, we measured the blood concentration of sEVs at different time points after tail vein injection of sEVs. The results showed that 5 min after 50 µg sEV injection, the blood sEV concentration was increased by about 27% (66 µg ml^−1^ to 84 µg ml^−1^); however, the concentration returned to baseline level a short time after injection (Extended Data Fig. 7a). Biophotonic imaging detected the intraosseous fluorescence signal in mice administrated with PKH26-labeled sEVs at either 4 or 8 h after administration (Fig. 4g) as previously described^25^. Six weeks after the first injection, μ-CT and TRAP staining showed that sh*SIRT2*-sEVs significantly abolished OVX-induced bone loss, with poorly organized trabecular architecture (Fig. 4h,i) and osteoclastogenesis (Fig. 4j–l); however, sh*SIRT2*-sh*LRG1*-sEVs reversed the osteoclastogenesis and bone phenotype in sh*SIRT2*-sEVs-treated OVX mice (Fig. 4h–l), indicating that sEV-LRG1 is required for the protection of sh*SIRT2*-sEVs against bone loss in vivo. Thereafter, we investigated the therapeutic potential of sEV-LRG1 in osteoporosis. Impressively, the present results showed substantially higher trabecular bone mass and better trabecular architecture as well as fewer TRAP-positive osteoclasts and lower osteoclast activities in the OVX mice treated with sEVs derived from *LRG1*-overexpressed AML12 cells (LRG1-sEVs) when compared to those in NC-sEV-treated OVX mice (Fig. 4h–l). Of note, the therapeutic effect of LRG1-sEVs was even better than sh*SIRT2*-sEVs. Together, these data demonstrate that LRG1 is the bona fide functional cargo of hepatocyte-derived sEVs and mediated the therapeutic effect of *SIRT2*-KO^hep^ on osteoclastogenesis and bone loss.

### Hepatocyte-derived sEV-LRG1 suppresses osteoclast differentiation by inhibiting RANKL-induced NF-κB p65 nuclear translocation

**Fig. 5:**
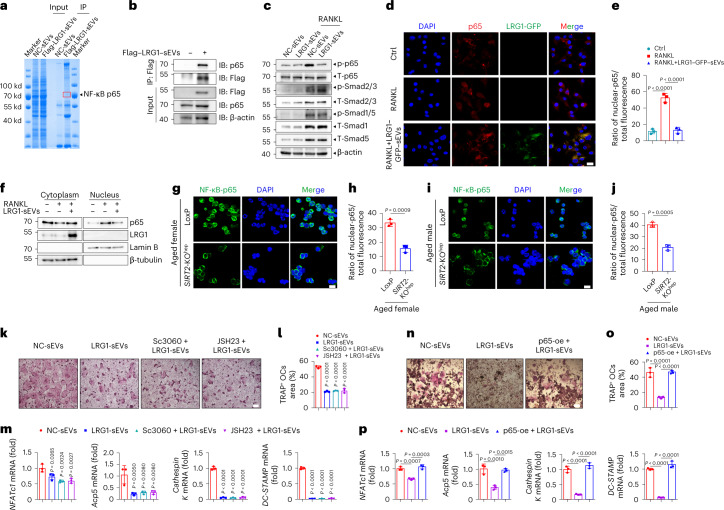
Hepatocyte-derived sEV-LRG1 inhibits osteoclastogenesis by repressing RANKL-induced NF-κB p65 nuclear translocation. **a**, Western blot (IB) of the hepatocyte-derived sEV-LRG1 binding proteins identified by IP assays in BMDMs, followed by LC–MS. **b**, Endogenous sEV-LRG1-NF-κB p65 interaction was analyzed by the amount of NF-κB p65 co-immunoprecipitated with sEVs–Flag–LRG1 (4 µg ml^−1^) in primary BMDMs. Representative from two independent biological experiments. **c**, Western blot analysis of phosphorylation of p65 and the activities of TGF-β signaling in osteoclasts treated with LRG1-sEVs (4 µg ml^−1^). Representative result from two independent biological experiments. **d**,**e**, Immunofluorescence analysis of p65 (red) location in RANKL-induced BMDMs treated with LRG1–GFP–sEVs (4 µg ml^−1^) (green) (scale bar, 20 µm) and quantitation of ratio of nuclear p65 to total p65 was measured by ImageJ. Representative result from three independent biological experiments. **f**, BMDMs were treated with RANKL or LRG1-sEVs (4 µg ml^−1^) for 24 h followed by nucleocytoplasmic separation analysis with western blot. Lamin B and β-tubulin served as internal controls for the nucleus and cytoplasm, respectively. Representative results from two independent biological experiments. **g**,**i**, Represented immunofluorescence images of primary BMDMs isolated from aged female (**g**) or male (**i**) LoxP (*n* = 3, 18 months of age) and *SIRT2-*KO^hep^ mice (*n* = 3, 18 months of age) (scale bar, 20 µm). **h**,**j**, Quantitation of ratio of nuclear p65 to total p65 in BMDMs of female (**h**) or male (**j**) LoxP and *SIRT2-*KO^hep^ mice was measured by ImageJ. (**g**–**j**, one technical replicate of three biological replicates for each group). **k**, TRAP staining of osteoclasts treated with LRG1-sEVs (4 µg ml^−1^) and the inhibitors of p65 nuclear translocation, Sc-3060 (10 µM) and JSH-23 (6 µM) (scale bar, 200 µm). **l**, Quantitation of the area of multi-nucleated TRAP^+^ cells with indicated treatment. **m**, The mRNA expression of osteoclast-specific genes in each group osteoclast was measured by real-time PCR. (**k**–**m**, *n* = 3 biologically independent experiments). **n**, TRAP staining of RAW 264.7 cells overexpressing p65 and treated with LRG1-sEVs (4 µg ml^−1^) (scale bar, 200 µm). **o**, Quantitation of the area of multi-nucleated TRAP^+^ cells with indicated treatment. **p**, The mRNA expression of osteoclast-specific genes in each indicated osteoclast group was measured by real-time PCR. (**n**–**p**, *n* = 3 biologically independent experiments). Data are presented as mean ± s.d., with biologically individual data points shown. *P* values were determined by unpaired two-tailed Student’s *t*-test (**h**,**j**) and one-way ANOVA followed by Tukey’s test (**e**,**l**,**m**,**o**,**p**). Source data

To understand the mechanism underlying the inhibitive effect of hepatocyte-derived sEV-LRG1 on osteoclastogenesis, we isolated sEVs from the medium of AML12 hepatocytes transduced with LRG1–GFP fusion protein and then observed the sEV-LRG1–GFP internalization. Immunostaining analysis showed that after either 12 h or 24 h of co-culture, most of the hepatocyte-derived sEVs were internalized and LRG1–GFP, co-localized with PKH26, was evenly distributed in the cytoplasm of BMDMs (Extended Data Fig. 7b). Given that hepatocyte-derived sEVs can transmit molecular cargos into recipient cells and LRG1 mediates protein–protein interactions as reported previously, the screening of proteins interacting with hepatocyte-derived sEV-LRG1 was performed in BMDMs. We treated BMDMs with the sEVs derived from the AML12 cells overexpressed Flag–LRG1 (Flag–LRG1-sEVs), followed by affinity purification using an anti-Flag antibody and the bound proteins were analyzed by liquid chromatography with tandem mass spectrometry (LC–MS/MS). MS analysis revealed that NF-κB p65 was the only predicted pro-osteoclastic factor among the proteins interacting with sEV-LRG1 (Fig. 5a and Extended Data Fig. 7c). Further, both immunoprecipitation (IP) assay and immunofluorescence staining validated the endogenous interaction of sEV-LRG1 with p65 in the cytoplasm of primary BMDMs (Fig. 5b,d). Moreover, LRG1-sEVs obviously abolished RANKL-induced p65 phosphorylation (Fig. 5c) and nuclear translocation (Fig. 5d,e). More notably, both immunofluorescence (Fig. 5d,e) and nucleocytoplasmic separation (Fig. 5f) results verified that sEV-LRG1 markedly reduced RANKL-induced p65 nuclear translocation. Further, compared to aged LoxP mice, the nuclear colocalization of p65 was significantly decreased in the primary BMDMs isolated from aged *SIRT2*-KO^hep^ mice (Fig. 5g–j), suggesting an inhibitive effect of the hepatic SIRT2–sEV-LRG1 axis on p65 nucleus translation in vivo. Next, we wanted to determine whether the nuclear translocation of p65 is necessary for the suppression of sEV-LRG1 in osteoclast differentiation. Neither Sc-3060 nor JSH-23, the inhibitors of p65 nuclear translocation, can further inhibit RANKL-induced NFATc1 signaling activation in the osteoclasts administrated with LRG1-sEVs (Fig. 5k,m), as well as the number and size of osteoclasts (Fig. 5l). In contrast, p65 overexpression totally reversed the sEV-LRG1-induced inhibition of osteoclastogenesis and NFATc1 signaling in RANKL-treated RAW 264.7 cells (Fig. 5n–p and Extended Data Fig. 7f).

LRG1 has been previously reported to promote angiogenesis by modulating endothelial transforming growth factor (TGF)-β signaling^22^. The immunohistochemistry (IHC) data showed a similar amount of blood vessels in the distal femurs of aged *SIRT2-*KO^hep^ mice compared to that of aged LoxP mice (Extended Data Fig. 7d,e). Meanwhile, MS results showed no component of the TGF-β receptor complex binding to sEV-LRG1, and western blot revealed that LRG1-sEVs had no effect on RANKL-induced TGF-β signaling activation in primary BMDMs, including Smad1/5 and Smad2/3 signaling (Fig. 5c). These data exclude the possibility that the inhibition of osteoclastogenesis by sEV-LRG1 was due to promoting TGF-β signaling and angiogenesis. In addition, a recent report showed that LRG1 promoted both angiogenic and neurotrophic processes in mouse tissue explants under hyperglycemic conditions by interacting with the adhesion GPCR latrophilin-2 (LPHN2), a new TGF-β-independent receptor of LRG1 (ref. ^26^). To investigate whether LPHN2 is involved in the transferring and signaling of sEV-LRG1, the sEV binding experiment was performed in 293T cells as previously described^27^. The results revealed no coexistence of immunoreactivities for hepatocyte-derived sEV-LRG1–GFP and LPNH2 (Extended Data Fig. 7g), suggesting that sEV-LRG1 transferred and played roles independently of binding LPNH2. In sum, these data support the mechanism that hepatocyte-derived sEV-LRG1 suppresses osteoclast differentiation through directly binding cytoplasmic NF-κB p65, not through regulating the NF-κB-NFATc1 and LPNH2 signal pathway.

### Pharmacological inhibition of SIRT2 attenuates bone loss and osteoporosis

**Fig. 6:**
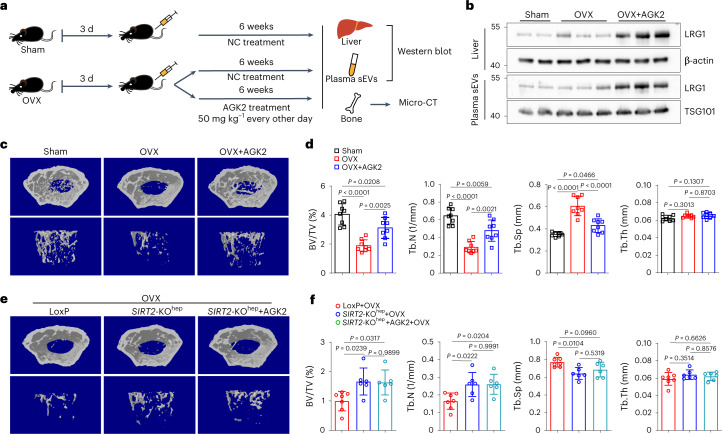
SIRT2 inhibitor AGK2 significantly suppresses OVX-induced bone loss in vivo. **a**, The experimental procedure for AGK2 treatment on OVX mouse model. **b**, Western blot analysis of LRG1 protein expression in the livers and plasma sEVs of OVX C57BL/6J mice treated with AGK2. (Two technical replicates of two (sham mice), three (OVX mice) and three (OVX + AGK2 mice) biological replicates for each group). **c**,**d**, Represented images of 3D restoration and quantification of trabecular BV/TV, Tb.N, Tb.Sp and Tb.Th of distal femurs of OVX C57BL/6J mice after 6 weeks of intraperitoneal injection of AGK2 (50 mg kg^−1^, every other day), as measured by μ-CT (*n* = 8, sham mice; *n* = 8, OVX mice; *n* = 8, OVX + AGK2 mice). (**c**,**d**, one technical replicate of eight biological replicates for each group). **e**,**f**, Micro-CT analysis of 3D restoration and quantification of trabecular BV/TV, Tb.N, Tb.Sp and Tb.Th of distal femurs of OVX-*SIRT2*-KO^hep^ mice after 6 weeks of treatment of AGK2 (*n* = 7, LoxP + OVX mice; *n* = 6, *SIRT2*-KO^hep^ + OVX mice and *n* = 6, *SIRT2*-KO^hep^ + OVX + AGK2 mice). (**e**,**f**. one technical replicate of seven (LoxP + OVX mice), six (*SIRT2*-KO^hep^ + OVX mice) and six (*SIRT2*-KO^hep^ + OVX + AGK2 mice) biological replicates for each group). Data are presented as mean ± s.d., with biologically individual data points shown. *P* values were determined by one-way ANOVA followed by Tukey’s test (**d**,**f**). Source data

**Extended Data Fig. 8:**
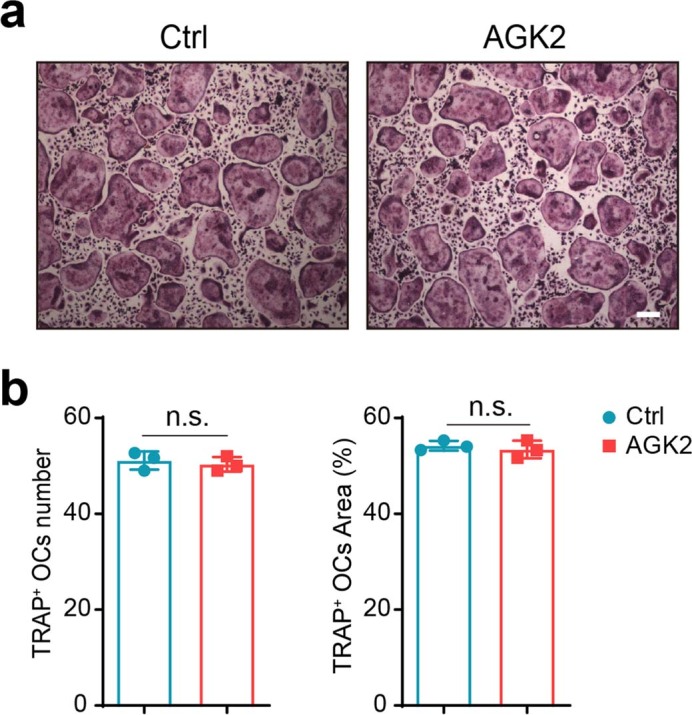
AGK2 has no effect on osteoclast differentiation. **a**, Representative TRAP staining images of BMDMs treated with RANKL and AGK2 (10 µM) or DMSO (scale bar, 200 µm). **b**, Number and area of multi-nucleated TRAP^+^ cells with indicated treatment were measured. n = 3 biologically independent experiments. Data are presented as mean ± SD. with biologically individual data points shown. *P* values are determined by unpaired two-tailed Student’s *t*-test (**b**). n.s., not significant. Source data

Given the protective role of *SIRT2*-KO^hep^ in both aging-associated and OVX-induced osteoporosis, we evaluated whether AGK2, a specific SIRT2 inhibitor, can be repositioned for prevention or treatment of osteoporosis. As expected, 6-week intraperitoneal injections of AGK2 (50 mg kg^−1^, every other day, started 3 d after OVX) markedly upregulated LRG1 protein levels both in the livers and plasma sEVs of OVX C57BL/6 mice (Fig. 6a,b). Notably, AGK2 treatment significantly increased bone mass and improved trabecular architecture in OVX mice (Fig. 6c,d). The intraperitoneal injection of AGK2 may be widely distributed throughout the body. Therefore, we asked whether intraperitoneal AGK2 treatment worked mainly through inhibiting SIRT2 in hepatocytes and not in other types of cells. To do so, the effect of AGK2 in osteoclasts was first verified and the result showed that AGK2 had no inhibitory effect on osteoclast differentiation (Extended Data Fig. 8). Then, we evaluated the efficiency of AGK2 in *SIRT2*-KO^hep^ mice. Notably, there was no difference in bone mass and trabecular architecture between vehicle-treated and AGK2-treated OVX-*SIRT2*-KO^hep^ mice (Fig. 6e,f), suggesting that hepatocyte SIRT2 is the major therapeutic target of AGK2. Thus, pharmacological inhibition of hepatocyte SIRT2 is a promising approach that should be effective for the prevention and treatment of osteoporosis.

**Extended Data Fig. 9:**
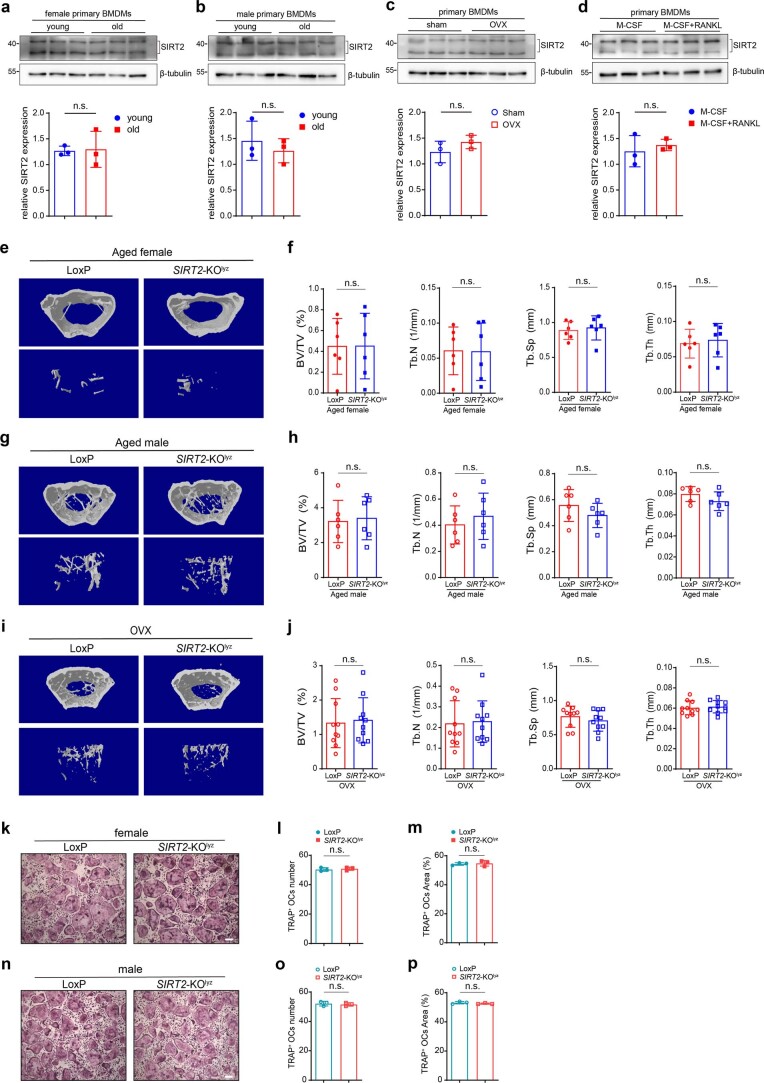
BMDM-specific *SIRT2* knockout has no effect on inhibiting osteoclastogenesis and slowing down bone loss in aged and OVX mice. **a,b**, Western blot analysis of SIRT2 protein expression in the primary BMDMs derived from female (**a**)or male (**b**) young (n = 3, 3 months of age) and aged (n = 3, 18 months of age) mice.**c**, Western blot analysis of SIRT2 protein expression in the primary BMDMs derived from sham (n = 3) and OVX (n = 3) mice (12 weeks of age). (a-c, one technical replicate of 3 biological replicates for each group). **d**, Western blot analysis of SIRT2 protein expression in the primary BMDMs treated with RANKL. n = 3 biologically independent experiments. (a-d, Western blot density analyzed by ImageJ and quantification analysis was shown). **e-h**, Represented images of 3D restoration and quantification of trabecular BV/TV, Tb.N., Tb.Sp. and Tb.Th. of distal femur of the aged female (**e,f**) or male (**g,h**) LoxP (both n = 6) and *SIRT2-*KO^lyz^ (both n = 6) mice, as measured by micro-CT. One technical replicate of 6 biological replicates for each group. **i,j**, Represented images of 3D restoration and quantification of trabecular BV/TV, Tb.N., Tb.Sp. and Tb.Th. of distal femur of the OVX female LoxP (n = 10) and *SIRT2-*KO^hep^ (n = 10) mice (12 weeks of age), as measured by micro-CT. One technical replicate of 10 biological replicates for each group. **k,n**, BMDMs were isolated from LoxP and *SIRT2-*KO^lyz^ mice (8 weeks of age) and cultured with murine M-CSF and RANKL stimulation for 7 days to generate osteoclasts. Representative TRAP staining images of osteoclasts (scale bar, 200 µm). **l,m,o,p**, Number and area of multi-nucleated TRAP^+^ cells of female (**l-m**) or male (**o-p**) mice. n = 3 biologically independent experiments. Data are presented as mean ± SD. with biologically individual data points shown. *P* values are determined by unpaired two-tailed Student’s *t*-test (**a-d,f, h, j, l, m, o, p**). n.s., not significant. Source data

Furthermore, we observed whether SIRT2 in BMDMs plays a role in osteoporosis. There was no change of SIRT2 protein expression in primary BMDMs from both aged and OVX mouse models (Extended Data Fig. 9a–c). Moreover, RANKL had no effect on SIRT2 expression in murine BMDMs (Extended Data Fig. 9d). We also generated BMDM-specific SIRT2 knockout mice (*SIRT2*^flox/flox^Lyz2-Cre^+^, *SIRT2*-KO^lyz^) by using Cre driven by Lyz2 (Lysosome C-2) promoters^28^. The results showed that in both aged- and OVX-induced osteoporosis mouse models, BMDM-specific SIRT2 deficiency had no effects on bone loss and osteoporosis (Extended Data Fig. 9e–j). Moreover, the isolated BMDMs from *SIRT2*-KO^lyz^ mice showed the similar RANKL-induced osteoclastogenesis with BMDMs of LoxP littermates (Extended Data Fig. 9k–p). All the above results indicate that the hepatocyte SIRT2-regulated liver–bone axis, not BMDM-intrinsic SIRT2, is the predominant regulator of osteoclastogenesis and osteoporosis.

### Hepatocyte-derived sEV-LRG1 inhibits human osteoclast differentiation

**Fig. 7:**
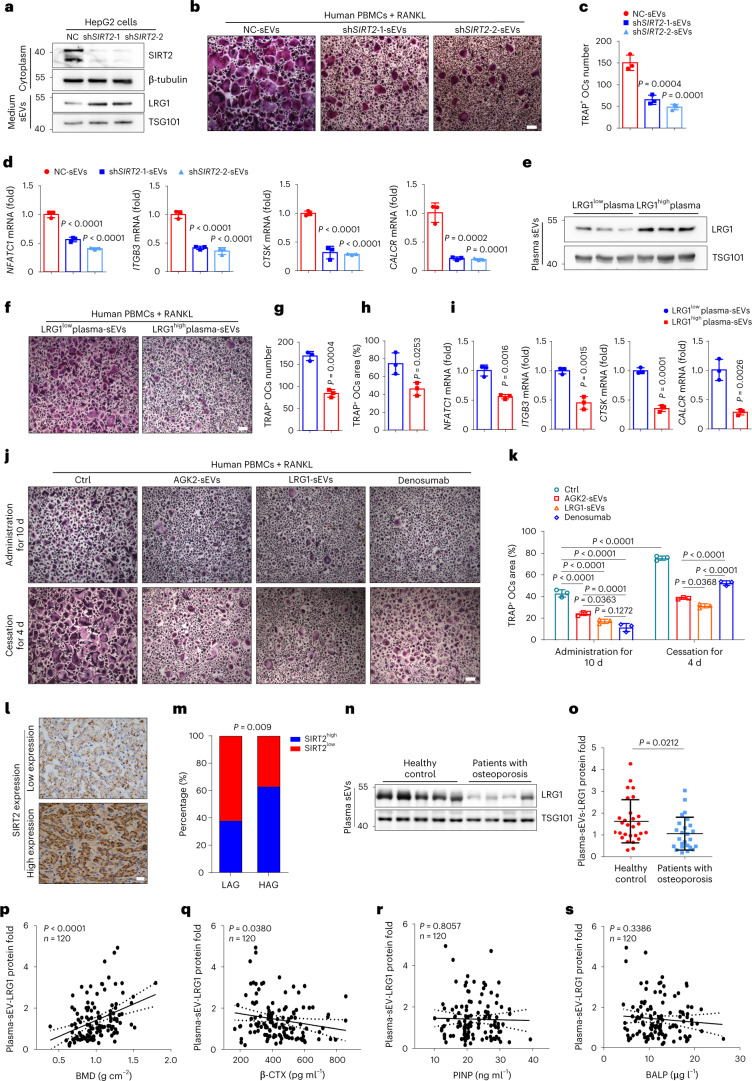
Hepatocyte-derived sh*SIRT2*-sEVs or human LRG1^high^ plasma sEVs inhibit human osteoclast differentiation and plasma sEV-LRG1 inversely correlates with bone resorption in patients. **a**, Western blot analysis of LRG1 protein expression in the cytoplasm and sEVs derived from *SIRT2*-knockdown (sh*SIRT2*) HepG2 human hepatocytes. **b**, Representative TRAP staining images of human PBMCs cultured with RANKL and sEVs (10 µg ml^−1^) derived from the supernatant of control and shSIRT2-HepG2 cells (NC-sEVs, sh*SIRT2*-1-sEVs, sh*SIRT2*-2-sEVs) (scale bars, 200 µm). **c**, Number of multi-nucleated TRAP^+^ cells with indicated treatment was measured. **d**, The mRNA expression of osteoclast-specific genes in the corresponding treated osteoclasts was measured by real-time PCR. (**a**–**d**, *n* = 3 biologically independent experiments). **e**, Western blot analysis of LRG1 protein expression in sEVs derived from LRG1-high- or low-expression plasma. **f**, Representative TRAP staining images of human PBMCs cultured with RANKL and sEVs (20 µg ml^−1^) derived from three LRG1-high-expression human plasma or three LRG1-low-expression human plasma (LRG1^low^ plasma sEVs, LRG1^high^ plasma sEVs) (scale bars, 200 µm). **g**,**h**, Number and area of multi-nucleated TRAP^+^ cells. **i**,The mRNA expression of osteoclast-specific genes measured by real-time PCR. (**e**–**i**, *n* = 3 biologically independent experiments). **j**, The inhibitory effect of sEVs and denosumab on osteoclast differentiation and the rebound effect after cessation of treatments. Representative TRAP staining images of PBMCs cultured with RANKL and sEV-LRG1 (10 µg ml^−1^) or denosumab (500 ng ml^−1^) (scale bars, 200 µm). The inhibitory experiments were performed by the administration of RANKL and sEVs or the commercial denosumab. After 10 d of treatment, half of PBMCs were stained by TRAP (top). At the same time, for the other half of PBMCs, both sEVs and denosumab administration were stopped, but PBMCs continued to be treated with RANKL for 4 d (bottom). **k**, Area of multi-nucleated TRAP^+^ cells with indicated treatment was measured. (**j**–**k**, *n* = 3 biologically independent experiments). **l**,**m**, The presented IHC images of SIRT2 expression levels in human liver tissues and association between SIRT2 expression levels and different ages are shown. LAG, low age groups (age <51 years, *n* = 54); HAG, high age groups (age >51 years, *n* = 60). **n**, Representative western blot analysis of protein expression of plasma sEV-LRG1 from female healthy control (BMD T score of lumbar spine is >−1, *n* = 28) and patients with osteoporosis (BMD T score of lumbar spine <−2.5, *n* = 25). **o**, Plots of protein expression of plasma sEV-LRG1 in female healthy control (*n* = 28) and osteoporotic patient group (*n* = 25). Association between human plasma sEV-LRG1 expression and BMD (**p**), bone resorption marker β-CTX (**q**) and bone formation markers PINP (**r**) and BALP (**s**) in 120 human participants of both sexes (females, *n* = 84 and males, *n* = 36). Data are presented as mean ± s.d., with biologically individual data points shown. *P* values were determined by one-way ANOVA followed by Tukey’s test (**c**,**d**), unpaired two-tailed Student’s *t*-test (**g**–**i**), unpaired two-tailed Mann–Whitney *U*-test (**o**), two-way ANOVA followed by Tukey’s test (**k**), two-tailed Spearman’s correlation test (**m**) and linear correlation and regression analyses (**p**–**s**). Source data

**Extended Data Fig. 10:**
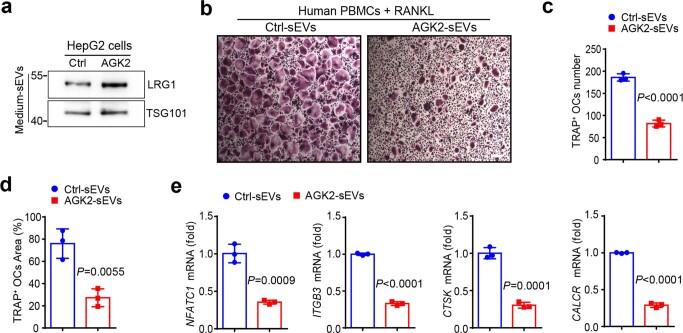
Hepatocyte-derived AGK2-sEVs inhibit human osteoclast differentiation. **a**, Western blot analysis of LRG1 protein expression in the sEVs derived from HepG2 cells treated with AGK2 (5 µM). **b**, Representative TRAP staining images of human PBMCs cultured with RANKL and sEVs(10 μg/ml) derived from the supernatant of HepG2 cells treated with control or AGK2 (Ctrl-sEVs, AGK2-sEVs) (scale bars, 200 µm). **c,d**, Number and area of multi-nucleated TRAP^+^ cells. **e**, The mRNA expression of osteoclast-specific genes measured by real-time PCR. n = 3 biologically independent experiments. Data are presented as mean ± SD. with biologically individual data points shown. *P* values are determined by unpaired two-tailed Student’s *t*-test (**c**-**e**). Source data

In our final analyses, we investigated whether SIRT2 inhibition and sEV-LRG1 could also suppress human osteoclast differentiation. For this purpose, we isolated primary human peripheral blood mononuclear cells (PBMCs) and induced osteoclast differentiation according to the previous report^29^. PBMCs were cultured with the sEVs purified from the supernatant of human hepatocyte HepG2 cells stably transfected with sh*SIRT2* (sh*SIRT2*-HepG2-sEVs) or control (NC-HepG2-sEVs) plasmids. Western blot confirmed the knockdown of SIRT2 in sh*SIRT2*-hepatocytes and the upregulation of LRG1 protein in sh*SIRT2*-HepG2-sEVs (Fig. 7a). Similar to the finding in mice, sh*SIRT2*-HepG2-sEVs treatment obviously inhibited osteoclasts differentiated from PBMCs (Fig. 7b–d). Similarly, supplementing with human sEVs derived from HepG2 cells treated with AGK2 (AGK2-HepG2-sEVs) markedly abolished RANKL-induced differentiation of human PBMCs (Extended Data Fig. 10). Further, sEVs were isolated from the patients’ plasma. The sEV-LRG1 was measured by western blot and was presented as the ratio of LRG1 grayscale value to TSG101 grayscale value. To make the results of different western blots comparable, the expression of sEV-LRG1 from a patient with healthy BMD served as a control. The present results showed that sEVs with LRG1 high expression (LRG1^high^ plasma sEVs) resulted in less number and size of TRAP-positive osteoclasts differentiated from PBMCs compared to the treatment of sEVs derived from LRG1-low-expressed human plasma (LRG1^low^ plasma sEVs) (Fig. 7e–h), as well as the decreased expression of osteoclast-specific genes (Fig. 7i). In sum, hepatocyte-derived LRG1-rich sEVs significantly suppress RANKL-induced human osteoclast differentiation. Further, we compared the inhibitory effect of sEVs and RANKL inhibitor denosumab on osteoclast differentiation. The isolated human PBMCs were treated with RANKL and denosumab or the sEVs purified from the supernatant of human hepatocyte HepG2 cells treated with AGK2 (AGK2-sEVs) or stably transfected with LRG1 (LRG1-sEVs). After 10 d of treatment, both sEVs and denosumab showed obvious inhibitory effects on osteoclast differentiation. Moreover, the inhibitive efficiency of LRG1-sEVs was comparable to that of denosumab (Fig. 7j,k). As referred to in previous reports, denosumab discontinuation is associated with rebound increase in bone resorption and subsequent loss in bone mass^30,31^. We also found that there is an obvious rebound effect on the human osteoclast differentiation 4 d after cessation of denosumab. Remarkably, both sEV treatments showed longer inhibitive effects and slower rebound increase in osteoclastogenesis after discontinuation of sEVs (Fig. 7j,k). This indicates that the hepatocyte-derived high-expressing LRG1-sEVs treatment was superior to denosumab in rebound effect after stopping medication.

### Plasma sEV-LRG1 positively correlates with BMD and inversely correlates with bone resorption marker in patients

To gain insights into human disease, we first analyzed SIRT2 protein expression in human liver samples by IHC staining. Hepatic tissues presented different degrees of immunoreactive scores (IRSs) of SIRT2 (Fig. 7l). And the association between SIRT2 expression (*n* = 114) and different ages was analyzed. For the 60 specimens from the high age group (HAG; age >51 years), 63% of liver tissues showed higher SIRT2 expression (IRS > 10, SIRT2^high^), whereas 38% showed higher SIRT2 expression in the low age group (LAG; *n* = 54, age <51 years) (Fig. 7m), suggesting that hepatic SIRT2 protein expression was positively correlated with age. Furthermore, we analyzed data from 120 patients with osteoporosis or healthy bone mass for associations between bone-related parameters and plasma sEV-LRG1 levels. The patients presented different protein levels of plasma sEV-LRG1 and BMD, the gold standard in the diagnosis of osteoporosis is assessment using dual X-ray absorptiometry. Notably, compared to the female control group (healthy bone density), there was significantly decreased protein levels of plasma sEV-LRG1 in the female patients with osteoporosis by western blot (Fig. 7n,o). Moreover, plasma sEV-LRG1 protein expression was strongly and positively correlated with BMD and inversely related to the clinical bone resorption marker β-CTX (Fig. 7p,q). In comparison, there was no association between plasma sEV-LRG1 level and the clinical bone formation marker PINP and bone-specific ALP (BALP) (Fig. 7r,s). Collectively, our results suggest that plasma sEV-LRG1 suppresses clinical bone resorption.

## Discussion

The present study describes a highly significant function of hepatocyte–osteoclast communication, where upregulation of hepatocyte SIRT2 is involved in aging-associated NF-κB p65 activation and osteoclastogenesis through sEV-LRG1. Our results show that hepatocyte-specific *SIRT2* deficiency (*SIRT2*-KO^hep^) markedly abolishes bone loss in aged and postmenopausal osteoporosis mouse models. Mechanistically, we revealed that *SIRT2*-KO^hep^-upregulated hepatic LRG1 transfers to BMDMs through hepatocyte-derived sEVs, resulting in inhibition of NF-κB p65-NFATc1 activation and osteoclastogenesis. Our findings greatly extend our current understanding of the pathophysiological role of the liver in bone metabolism and suggest that the liver–bone communication by SIRT2-sEV-LRG1 may function to restore bone homeostasis in older people or postmenopausal women. Notably, treatment with AGK2 or LRG1-sEVs conferred a therapeutic benefit in osteoporosis, including in animal models and human primary cell cultures, corroborating targeting hepatocyte SIRT2 or sEV-LRG1 as a promising therapeutic modality in primary osteoporosis.

SIRT2 has been implicated in the aging process and liver diseases. Our group recently found that hepatic SIRT2 prevents ALD through deacetylating CCAAT/enhancer-binding protein (C/EBP)-β^20^. In addition, we also reported that SIRT2 in macrophages prevents and reverses aging-associated inflammation and insulin resistance through deacetylation of NLRP3 (ref. ^21^). In the present study, we mainly focused on the function of hepatic SIRT2 in osteoporosis, therefore the liver-specific knockout mice and AAV8 viral expression system with hepatocyte-specific TBG promoter were used to exclude the influence of SIRT2 in other organs. In a previous report, Jing et al. reported that SIRT2 knockout prevents bone loss in 36-week-old rats. In vitro, RANKL increased SIRT2 mRNA expression in rat BMDMs and AGK2 inhibited rat osteoclast differentiation^32^. As global SIRT2 knockout rats were used, the study did not allow for consideration of potential interaction among different organs and there is the possibility that hepatocyte SIRT2 deficiency contributed to the protective effects on bone loss. Therefore, it is important to dissect which SIRT2-regulated mechanism (liver–bone axis or bone-intrinsic) is predominant. First, SIRT2 protein expression in primary BMDMs from both aged and OVX mouse models was examined and no change was observed (Extended Data Fig. 9a–c). Moreover, RANKL had no effect on SIRT2 expression in murine BMDMs (Extended Data Fig. 9d); however, SIRT2 protein levels in the hepatocytes of aged mice and older patients were significantly higher than that of young mice and patients (Fig. 1a–c and Fig. 7l,m). To investigate whether SIRT2 in BMDMs plays a role in osteoporosis, we generated BMDM-specific SIRT2 knockout mice (*SIRT2*^flox/flox^Lyz2-Cre^+^, *SIRT2*-KO^lyz^) by using Cre driven by Lyz2 (Lysosome C-2) promoters^28^. Aged- and OVX-induced osteoporosis mouse models were constructed. The μ-CT analysis showed that aged *SIRT2*-KO^lyz^ mice (18 months old) of both sexes exhibited similar bone loss and osteoporosis compared to aged LoxP mice (Extended Data Fig. 9e–h). Moreover, the bone loss in OVX-*SIRT2*-KO^lyz^ and OVX-LoxP mice was comparable (Extended Data Fig. 9i,j). Meanwhile, isolated BMDMs from *SIRT2*-KO^lyz^ mice showed similar RANKL-induced osteoclastogenesis as seen with BMDMs of LoxP littermates (Extended Data Fig. 9k–p). These results indicate that genetic deletion of SIRT2 in BMDMs has no effect on inhibiting osteoclastogenesis and slowing down bone loss during aging in mice. In addition, the TRAP result showed that AGK2 had no direct inhibitory effect on osteoclast differentiation in vitro (Extended Data Fig. 8), while AGK2 obviously attenuated bone loss in vivo (Fig. 6c,d) and AGK2-sEVs suppressed osteoclastogenesis (Fig. 6 and Extended Data Fig. 10). Notably, AGK2 treatment cannot further improve the ameliorated bone mass and trabecular architecture in OVX-*SIRT2*-KO^hep^ mice (Fig. 6e,f). These results indicate that AGK2 prevents osteoporosis mainly through targeting hepatocyte SIRT2. Taken together, these data suggest that the hepatocyte SIRT2-regulated liver–bone axis, not BMDM-intrinsic SIRT2, is the predominant regulator of osteoclastogenesis and osteoporosis. Some current data differ from that of the Jing et al. study, including the effects of AGK2 on osteoclastogenesis in vitro, but the decreased bone loss in our aged *SIRT2*-KO^hep^ mice was further validated in global *SIRT2* knockout rats. BMDMs from different rodents (C57BL/6 mice for our studies versus Sprague Dawley rats for Jing et al.^32^) may have contributed to the inconsistent results between the two studies.

Moreover, hepatic SIRT2 exerts its effect on osteoclast differentiation mainly through upregulating the sEV cargo LRG1 protein level, not affecting sEV biogenesis, maturation and secretion. There are many hepatic non-sEV-related hormonal and signaling effectors involved in bone metabolism besides sEVs. We have ruled out the previously reported non-sEV-related hormonal and signaling effectors in the SIRT2-regulated liver–bone axis. First, we tested VitD metabolism and found there was no difference in the concentration of plasma total VitD and the expression of hepatic CYP2R1 and CYP27A1 between the aged LoxP and *SIRT2*-KO^hep^ group (Extended Data Fig. 2d–g). Second, the hepatic effectors that mediated the liver–bone axis were mainly transferred to bone cells via blood. We carefully checked the original MS data and found that there was no difference in the concentrations of the hormonal or signaling effectors involved in the liver–bone axis previously reported between the two groups, including fibroblast growth factor 21 (FGF21), insulin-like growth factor binding protein 1 (IGFBP1)^13,33^, lecithin-cholesterol acyltransferase (LCAT)^34^, transforming growth factor (TGF)-β^12,35^ and insulin-like growth factor (IGF)-1 (refs. ^36–38^). Though MS data showed the higher level of fibronectin in *SIRT2*-KO^hep^ mice, the result does not match our phenotype according to the previous report that fibronectin inhibits osteoblast function^39,40^. Furthermore, *SIRT2*-KO^hep^ plasma had inhibitive effects on osteoclastogenesis, but the inhibitive effects were abolished after removing sEVs (Fig. 2e–h). All these results suggested that SIRT2^−/−^ hepatocyte-derived sEVs, not non-sEV-related hormonal and signaling effectors, abolish osteoclastogenesis. Therefore, because Saa1 and Saa2 in Fig. 3a had a very low proportion in plasma sEVs (Extended Data Fig. 5f), the possibility that hepatic SIRT2 modulates osteoclast differentiation through them was also excluded.

This study provides evidence that hepatocyte-derived sEVs directly transfer anti-resorption factors from the liver to intraosseous osteoclasts. Moreover, LRG1 in hepatocyte-derived sEVs was originally discovered as an osteoclast cell fate determinant. LRG1 is expressed abundantly in hepatocytes, but lowly in osteoclasts. Our results show that the majority of secreted LRG1 protein in aged plasma was located in sEVs (Extended Data Fig. 5f), explaining why the *SIRT2*-KO^hep^ plasma has no inhibitive effects on osteoclastogenesis after depletion of sEVs (Fig. 2e–h). LRG1 is previously reported to promote angiogenesis by tipping the balance of TGF-β1 signaling toward the ALK1–Smad1/5/8 pathway in endothelial cells (ECs), which is dependent on the presence of the TGF-β1 type III receptor, endoglin^22^. In addition, recent literature described that miR-497-downregulated LRG1 promotes osteoblast viability and collagen synthesis via activating the TGF-β1–Smad signaling pathway^41^. In this study, we investigated whether the inhibitive role of sEV-LRG1 in osteoclastogenesis was also due to regulating TGF-β signaling. The MS and western blot results showed no interaction between sEV-LRG1 and endoglin. Moreover, there was no activation of TGF-β1 signaling after LRG1-sEVs treatment in osteoclasts (Fig. 5c), suggesting that hepatocyte-derived sEV-LRG1 exerts its function in osteoclasts through a mechanism independently of the TGF-β1 signaling pathway.

We also noticed that young *SIRT2*-KO^hep^ mice had higher LRG1 expression in the liver and plasma (Extended Data Fig. 5k), but the osteoclast number (Extended Data Fig. 6a,b) and bone mass (Extended Data Fig. 1) of young *SIRT2*-KO^hep^ mice were comparable to that of young LoxP mice. To explore this, we performed in situ immunofluorescence analysis of murine femurs (Extended Data Fig. 6c,d). The number of LRG1-expressing osteoclast progenitors in young *SIRT2*-KO^hep^ mice was similar to that of young LoxP mice, which may explain why there was no difference in osteoclast number and the bone mass between the two young groups. For the difference of the change of LRG1 expression in plasma and in osteoclast progenitors, presumably it is because the increased hepatocyte-derived plasma sEV-LRG1 might not proportionally transfer into osteoclast progenitors.

The immunofluorescence showed no binding of LRG1 on cell membrane and strong signal in cytoplasm. Furthermore, hepatocyte-derived sEV-LRG1 directly binds to p65 and inhibits p65 nuclear translocation after uptake by BMDMs. It has been widely reported that phosphorylation of p65 results the nuclear translocation^42–46^, whether sEV-LRG1 directly binds to the phosphorylation site of p65 or whether sEV-LRG1 affects the binding of other phosphorylases in addition to p65 needs further study. Therefore, we revealed a direct inter-organ regulatory mode between histone acetylation in hepatocytes and phosphorylation of transcription factor in osteoclasts via sEV protein cargo transfer. While the intracellular NF-κB p65-NFATc1 signal is over-activated in osteoclasts, LRG1 protein transferred into osteoclasts by the extracellular hepatocyte-derived sEVs acts as a brake on pro-osteoclastic activity to maintain bone homeostasis (Fig. 5). A recent report showed that LRG1 can induce phosphorylation of NF-κB p65 in human umbilical vein ECs (HUVECs) by interacting with LPHN2 (ref. ^26^); however, the sEV binding experiment showed no binding of sEV-LRG1 and LPNH2 (Extended Data Fig. 7g). Meanwhile, the reported regulation of LPHN2 on p65 mainly contributed to angiogenesis, but IHC data revealed a similar amount of blood vessels in the distal femurs of aged *SIRT2-*KO^hep^ mice compared to that of aged LoxP mice (Extended Data Fig. 7d,e). These results suggested that sEV-LRG1 transferred and played roles independently of binding LPNH2, partly explaining the different regulating effects on p65 activation between LRG1 in ECs and sEV-LRG1 in osteoclasts.

Osteoporosis is one of the major health problems worldwide and its incidence is growing with the aging population^47,48^. Moreover, accumulating studies reported that osteoporosis is a frequently observed complication in patients with CLD, particularly liver cirrhosis and cholestatic liver diseases^12,49^. The elusive mechanisms and poor outcomes are getting more attention. Therefore, identification of therapeutic targets on liver–bone communications is an urgent clinical need in primary osteoporosis. Epigenetics studies have provided new understanding in the mechanism of treatment and pathophysiology of bone remodeling occurring in osteoporosis. SIRT1 and SIRT6 have been implicated in bone metabolism^50,51^. A randomized, double-blind, placebo-controlled trial investigated the effects of resveratrol, an agonist of SIRT1, on BMD in obese individuals. Results indicated a significant dose-dependent increase of bone ALP and BMD, but to date, no data have been reported for women with osteoporosis^52^. In the present study, the specific inhibitor of SIRT2, AGK2 was verified as a promising therapeutic agent for osteoporosis in OVX mice. In the past few years, extracellular vesicle-based engineered delivery systems for precision nanomedicine have attracted wide interest across areas of molecular cell biology, pharmaceutical sciences and nanoengineering^53^. Here, we also identified LRG1-rich sEVs as an effective therapy for treatment of osteoporosis in mice. Moreover, sEVs derived from either human LRG1^high^ plasma or human hepatocytes with SIRT2 inhibition significantly suppress RANKL-induced osteoclastogenesis.

Targeting of the NF-κB pathway is a well-known current treatment for osteoporosis. Through comparison with denosumab, we verified the benefit of targeting hepatocyte SIRT2 and sEV-LRG1, the new upstream regulators of NF-κB signaling. On the one hand, both hepatocyte-derived AGK2-sEVs and LRG1-sEVs significantly inhibited osteoclastogenesis, especially the inhibitive efficiency of LRG1-sEVs was comparable to that of denosumab. On the other hand, both high-expressing LRG1-sEV treatments were confirmed to be superior to denosumab in rebound effect after stopping medication, as evidenced by longer inhibitive effects and slower rebound increase in osteoclastogenesis after cessation of sEVs (Fig. 7j,k). Though the in vitro results provide compelling evidence, further in vivo research is needed, such as using human RANKL knock-in mice.

The positive correlation between plasma sEV-LRG1 expression and BMD in clinical samples further strengthens the therapy potential of LRG1^high^-sEVs. Meanwhile, we analyzed the liver proteomics data of the hepatic osteodystrophy patients in the recent report^34^. There was the trend that patients with cirrhosis with decreased expression of hepatic LRG1 have lower bone mass, though the difference was not statistically significant. Here we mainly focused on primary osteoporosis without liver diseases and the correlation between hepatic LRG1 expression and primary osteoporosis still needs more clinical samples and further study. Therefore, our data provide definitive evidence that targeting hepatic SIRT2 or sEV-LRG1 is a powerful strategy for primary osteoporosis therapy.

Some limitations need to be mentioned here. We noted that though the increased sEV concentration (84 μg ml^−1^) is higher than that seen in vivo, the concentration returned to baseline level in a short time (Extended Data Fig. 7a) and the hepatic sEVs were retained by multiple organs (Extended Data Fig. 7a and Fig. 4g). The result suggests that hepatic sEVs may reach bones with a rapid fluctuation in plasma concentration. Next, we will investigate and modify hepatic sEVs to make them more efficient to transport to osteoclasts, and lower-dose sEVs may play a therapeutic role in osteoporosis. In addition, for some in vitro results, the biological replicates in a few experiments were small and we will improve this in future research.

**Fig. 8:**
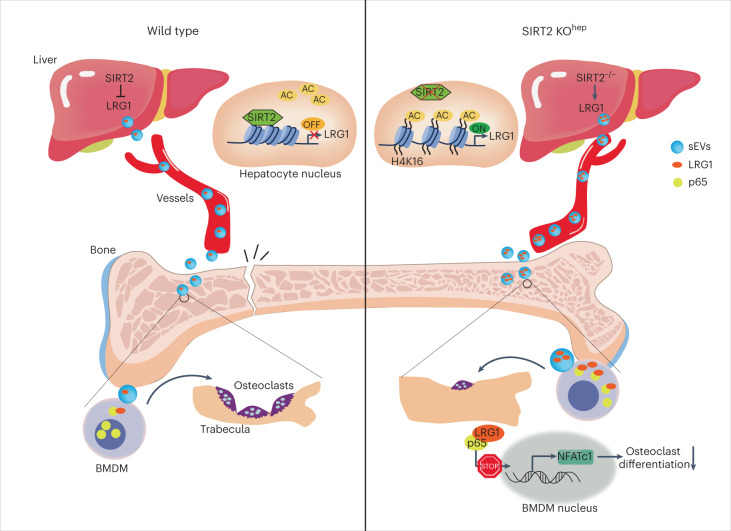
A working model of the hepatic SIRT2-regulated liver–bone communication. The upregulation of hepatocyte SIRT2 involved in aging-associated NF-κB p65 activation and osteoclastogenesis through sEV-LRG1. Upregulated LRG1 protein in SIRT2^−/−^ hepatocytes transferred into osteoclasts through sEVs acts as a brake on pro-osteoclastic activity to maintain aged and postmenopausal bone homeostasis. The inter-organ action of the SIRT2–sEV-LRG1–NF-κB–NFATc1 axis may be a promising therapeutic target in primary osteoporosis.

In summary, our findings therefore unveiled a working model of liver–bone communication, depicted in Fig. 8, to illustrate that hepatocyte SIRT2 regulates pro-osteoclastic signaling of NF-κB p65 in osteoclasts through the sEV-LRG1 pathway. The inter-organ action of SIRT2–sEV-LRG1–NF-κB–NFATc1 axis may also be essential to maintaining bone homeostasis and a promising therapeutic target in primary osteoporosis. Targeting hepatocyte SIRT2 or sEV-LRG1 may serve as a potential therapeutic strategy.

## Methods

### Animals

Hepatocyte-specific SIRT2 knockout mice (*SIRT2*-KO^hep^) were produced by crossing SIRT2^flox/flox^ mice obtained from Johan Auwerx Laboratory (Switzerland) and Alb-Cre mice purchased from The Jackson Laboratory (US) in a C57BL/6 background. The BMDM-specific SIRT2 knockout mice (*SIRT2*^flox/flox^Lyz2-Cre^+^, *SIRT2*-KO^lyz^) were generated by using Cre driven by Lyz2 (lysosome C-2) promoters.C57BL/6 female mice were purchased from the Shanghai SLAC Laboratory Animal Co. All mice were housed at a specific-pathogen-free environment in the Animal Laboratory Unit of the Shanghai Jiao Tong University School of Medicine (SJTU-SM) and all mice were provided with sterile food and water. The animal experiments were performed in accordance with the approved guidelines by the Institutional Animal Care and Use Committee at SJTU-SM.

### Micro-CT analysis

Quantitative tomography of distal femoral metaphysis was performed using an X-ray μ-CT (Skyscan1076, Bruker micro-CT). Regions of interest were defined from 0 image slice to 200 image slices, where the growth plate slice was defined as 0 image slice. The standardized region of femurs were scanned at 9-μm resolutions. Trabecular bone mass and microarchitecture were defined, including BV/TV, Tb.N, Tb.Sp and Tb.Th.

### ELISA

The blood collected from mice was centrifuged for 30 min at 2,000*g* and the plasma was collected and stored at −80 °C for subsequent assays. Type 1 collagen amino-terminal propeptide (P1NP), type 1 collagen C breakdown products (CTX-1) (Elabscience) and total VitD (J&L biological) concentrations in the plasma were all performed according to the manufacturer’s instructions.

### Histology and IHC staining

After routine 4% formaldehyde fixation, decalcification with 10% EDTA (pH 7.4) for 1 week and paraffin-embedded (FFPE) specimen processing, bone sections (5–7 µm) were stained with hematoxylin and eosin and TRAP staining for histological evaluation of osteoblasts and osteoclasts in mice. Osteoclasts and osteoblasts surface were assessed relatively to the total bone surface as Oc.S/BS and Ob.S/BS. The expression levels of CD31^+^ vessels in the bone tissue slides of aged mice were tested using IHC according to a standard procedure. The corresponding antibodies (CD31 goat pAb, 1:200 dilution, ServiceBio, GB13063) and HRP-conjugated rabbit anti-goat IgG (H+L) (1:200 dilution, ServiceBio, GB23204). The quality control for IHC was administered with controls. Quantification of CD31^+^ vessels/bone surface ratio was measured. After routine FFPE specimen processing, liver tissue was sliced (3–4 µm). IHC was used to detect the expression level of SIRT2 protein in liver tissue sections of patients and mice. The corresponding antibodies are SIRT2 rabbit polyclonal antibody (1:250 dilution for patients and 1:200 dilution for mice, Sigma, S8447), HRP-conjugated goat anti-rabbit IgG (H+L) (1:200 dilution, ServiceBio, GB23303). Quality control for IHC was administered with controls. The evaluation of IHC staining was completed by two independent pathologists according to the immune response score (IRS) system as previously reported^20^. In this study, SIRT2 expression levels were divided into two categories, low (IRS ≤ 10) and high (IRS > 10).

### Cell culture

Mouse hepatic cell lines (AML12) were obtained from the American Type Culture Collection (ATCC). They were cultured in DMEM/F12 with 10% fetal bovine serum (FBS) (Sigma), 40 ng ml^−1^ dexamethasone (Sigma), 0.45% Liquid Media Supplement (ITS, Sigma) and incubated in a humidified atmosphere at 37 °C and 5% CO_2_. Human hepatic cell lines (HepG2) were obtained from ATCC and cultured in DMEM with 10% FBS (Sigma) and incubated in a humidified atmosphere at 37 °C and 5% CO_2_. HEK293T cells were obtained from ATCC and maintained in DMEM supplemented with 10% FBS (Sigma). RAW 264.7 cells were obtained from ATCC and maintained in DMEM supplemented with 10% FBS (Sigma). Mouse primary BM-MSCs were isolated from femur and tibia bones from 2–4-week-old C57BL/6 mice. Femur and tibia bones were obtained, rinsed while stripping muscle tissue and soft tissue was removed before transfer to the culture medium. Bone tissue was shredded with forceps and the shredded bone was rinsed with a 1-ml syringe to obtain bone-marrow cells, which were inoculated and cultured. The culture medium was changed after 2 d and passaged for culture. The cells at passage six were used for cell function experiments. Mouse primary BMDMs were isolated from C57BL/6 femur and tibia bones from 6–8-week-old mice. Briefly, bone-marrow cells were flushed out from long bones with ɑ-MEM medium (HyClone). Cell suspensions were filtered through a 100-µm cell strainer (FALCON) and cultured in α-MEM supplemented with 10% FBS (Sigma), 1% penicillin/streptomycin (Invitrogen) and 1% GlutaMAX Supplement (Thermo Fisher). After 24 h, the supernatant was collected and cell precipitation was obtained by centrifugation, then BMDMs were attached in α-MEM medium supplemented with 10% FBS, 1% GlutaMAX Supplement and 30 ng ml^−1^ murine M-CSF (Peprotech). BMDMs were collected at day 2 after M-CSF stimulus. For osteogenic induction, BM-MSCs were seeded in 12-well plates and treated with osteogenic medium (Cyagen). The culture medium was replaced every other day. At 7–14 d later, osteogenic differentiation was assessed by ALP staining (Beyotime Biotechnology) and ARS (Cyagen) according to the manufacturer’s protocol. For osteoclast differentiation, collected BMDMs were scraped and seeded into 12-well plates at a concentration of 2 × 10^5^ cells per well for differentiation experiments. Cells were stimulated with 50 ng ml^−1^ receptor activator of RANKL (R&D, 462-TEC-010) and 30 ng ml^−1^ M-CSF (Peprotech, 315-02-50) for 7 d and the medium was replaced every 2 d. Osteoclasts were fixed and stained using the TRAP staining kit (Sigma-Aldrich, 387A-1KT).

### Primary hepatocyte isolation

The perfusion buffer (50 ml Hanks’ balanced salt solution (HBSS; no Ca2^+^/Mg^2+^, Gibco,14175095), with 500 µl 7.5% NaHCO_3_ and 71.17 µl 0.5 M EDTA (Thermo Fisher, AM9260G) and digestion buffer (30 ml HBSS, with Ca2^+^/Mg^2+^ (Gibco, 14025134), with 300 µl 7.5% NaHCO_3_, 60 µl 2.5 M CaCl_2_ and collagenase type 2 (Worthington, LS004176) until the color turns to light brown) were prepared and placed in a metal bath and heated to 37 °C. Mice were anesthetized and then the liver, heart and portal vein were exposed, a syringe needle was inserted into the right atrium to start perfusion, then the portal vein was cut. The perfusion speed was 1–2 ml min^−1^ for 5 min. The perfusion buffer was stopped and changed to collagenase solution and perfusion was continued at a speed of 1 ml min^−1^ for 5 min until the liver was soft. The whole liver was transferred to a 10-cm dish and the gallbladder was removed. About 300 µl collagenase solution was dropped on the liver and digested in a 37 °C incubator for 3 min. The liver was crushed with ophthalmic forceps and then digested in a 37 °C incubator for a further 3 min. The cells were resuspended in 15 ml DMEM, filtered using a 100-µm strainer into a 50-ml centrifuge tube, then centrifuged at room temperature and 50*g* for 3 min. The supernatant was discarded, the precipitation was resuspended and centrifuged again and then counted, inoculated in the culture dish (coated with 0.1% type I collagen solution for 15 min) and the supernatant replaced 2 h later.

### sEV isolation and identification

The mouse hepatic AML12 cell lines were cultured in normal medium until 60–70% confluency, then the medium was replaced with 10% sEV-depleted FBS and cultured in normal conditions for 2 d. For the mouse primary hepatocytes, the supernatant was replaced with 10% sEV-depleted FBS 2 h after inoculation. The sEVs in the supernatant of hepatocytes were isolated by the ultracentrifugation method^27,54^. In brief, the supernatant was collected and centrifuged at 300*g* for 10 min, 2,000*g* for 10 min, 10,000*g* for 30 min and then ultracentrifuged at 110,000*g* for 90 min (Beckman Ultra high-speed refrigerated centrifuge) and the sediment was resuspended with PBS and stored at −80 °C for further use. Plasma sEVs were isolated using Exo Quick extraction reagent (SBI, EXOQ5TM-1) according to the manufacturer’s instructions^27,54,55^. In brief, the plasma was incubated with thrombin to convert fibrinogen into fibrin, then centrifuged to remove fibrin, cells and cell debris. Then 25 µl Exo Quick extraction reagent was added to a 100-µl sample (4:1), incubated at 4 °C for 1 h and rotated at 1,500*g* for 30 min to separate sEVs. The separated sEVs either from supernatant or plasma were found in the precipitate. Then, following centrifugation, the sEV pellet was removed and resuspended in PBS for the corresponding experiments. The remaining plasma after removal of sEVs was defined as plasma(-sEVs). The sizes of sEVs were analyzed using the Electrophoresis & Brownian Motion Video Analysis Laser Scattering Microscopy. In addition, microscopic images of sEVs were observed by transmission electron microscopy. Protein markers of sEVs, including Alix, HSP70 and TSG101 were measured by western blot analysis.

### In vivo and in vitro sEV treatment

The concentration of the sEVs was detected by a bicinchoninic acid protein assay kit. For in vitro assays, 2 µg murine hepatocyte medium sEVs or 5 µg aged murine plasma-derived sEVs were administered to 1 × 10^5^ murine BMDMs every other day; 10 µg medium sEVs from human HepG2 cells or 20 µg plasma sEVs from patients were administered to 5 × 10^5^ human PBMCs every other day. For in vivo treatment, 50 µg medium sEVs of AML12 cells were injected intravenously into a mouse every other day. In the experiment for the effect of human plasma sEVs on PBMC differentiation, three patients with plasma sEV-LRG1^low^ and three patients with plasma sEV-LRG1^high^ were selected and verified by western blot analysis. Then the three LRG1^low^ plasma sEVs were mixed together as well as three LRG1^high^ plasma sEVs and then co-cultured with PBMCs to observe their effects on osteoclastogenesis.

### sEV labeling and tracking

Purified sEVs isolated from plasma or culture medium were collected and labeled with PKH26, a red fluorescent membrane dye (Sigma) according to the manufacturer’s instructions. Labeled sEVs were isolated with ExoQuick Reagent (SBI); briefly, labeled sEVs were incubated with ExoQuick Reagent (5:1 dilution, SBI, EXOTC50A-1) overnight and centrifuged at 1,500*g* for 30 min. Then sEVs were resuspended in PBS and added to the BMDMs for sEV uptake studies. After incubation for 10 hours at 37 °C, cells were observed by Laser scanning confocal microscope (Nikon). Labeled sEVs were injected intravenously into mice and the distribution was monitored by BLI.

### RNA interference

For construction of stable cell lines, the shRNAs cloned into the pGIPZ vector were obtained from the scientific research platform of SJTU-SM. HEK293T cells were co-transfected with the lentivirus vector described above and packaging vectors psPAX2and pMD2.G with Lipofectamine 2000 transfection reagent (Invitrogen) for producing lentivirus. The p65 plasmid was transfected into RAW 264.7 cells with Lipofectamine 3000 transfection reagent (Invitrogen) according to the manufacturer’s instruction. The complementary DNA target sequences of shRNAs and primer sequences used for cloning in this study are provided in Supplementary Table 1.

### Western blot analysis

Cells, sEVs or mouse tissues were lysed in SDS-lysis or RIPA buffer. The protein samples were loaded into SDS–PAGE gels and then transferred onto 0.45-µm or 0.22-µm nitrocellulose membranes (Axygen). Membranes were blocked with 5% skimmed milk at room temperature for 1 h and incubated with primary antibodies at 4 °C overnight, following by incubation with the HRP-conjugated secondary antibodies at room temperature for 1 h. Finally, the membranes were visualized with an Enhanced Chemiluminescence (ECL) Detection kit (Millipore) and by using Image Quant LAS 4000 Mini (GE Healthcare Bio-Sciences AB). The primary antibodies used in the experiments are provided in Supplementary Table 2.

### Quantitative RT–PCR

Total RNA was extracted from cells or mouse tissue by Trizol reagent (Invitrogen) and was reverse-transcribed into cDNA with AMV Reverse Transcriptase XL (Takara). Quantitative real-time PCR was performed using SYBR Green PCR Master Mix (Applied Biosystems) and on the ABI 7300 PCR system (Applied Biosystems). The primer sequences used are provided in Supplementary Table 3.

### RNA-seq and LC–MS/MS

RNA from liver tissues (20 mg) was extracted. Total RNA was processed with mRNA enrichment method or rRNA removal method. Oligo(dT) magnetic beads were used to enrich the mRNA with a poly A tail; a DNA probe was used to hybridize rRNA and RNase H was used to selectively digest the DNA/RNA hybrid chain and then digest the DNA probe with DNase I. After purification, the obtained RNA was fragmented with the interrupted buffer. Then, first-strand cDNA was generated using reverse transcription with random N6 primers, followed by two-strand cDNA synthesis to form double-stranded DNA. Afterwards, the synthetic double-stranded DNA ends were filled and the 5′ end was phosphorylated to form a sticky end protruding an ‘A’ at the 3′ end. This was then connected to a blister linker with a protruding ‘T’ at the 3′ end. The ligation product was amplified by PCR with specific primers. The PCR product was heat-denatured into single-strands and then the single-stranded DNA was circularized with a bridge primer to obtain a single-stranded circular DNA library. Sequencing was conducted using the DNBSEQ platform (BGI-Shenzhen).

Differentially expressed proteins in the plasma of aged LoxP and *SIRT2*-KO^hep^ mice were determined by mass spectrometry of the Basic Medicine Public Technology Platform of SJTU-SM.

### Chromatin immunoprecipitation

Enrichment of H4K16ac on LRG1 promoter region from H4K16ac ChIP-seq was predicted in the Cistrome Data Browser (http://cistrome.org/db) and visualized at UCSC Genome Browser (http://genome.ucsc.edu)^56,57^. ChIP analysis was performed using the Millipore ChIP Assay kit (Millipore) according to the manufacturer’s instructions. Briefly, ChIP was performed with 5 × 10^6^ cells per reaction. Cells were crosslinked with formaldehyde for 10 min at room temperature and then sonicated. Corresponding IgG was used as controls. The precipitated DNA was quantified by qPCR with reverse transcription. Primers sequences used for ChIP are provided in Supplementary Table 4.

### Adeno-associated virus 8-mediated gene expression

An AAV8 delivery system was used to specifically knock down murine LRG1 in mouse liver. The open reading frame encoding the *LRG1* gene, without a stop codon, was cloned into an AAV8 package vector pAAV-TBG-T2A-luciferase. The mice were injected with 2 × 10^11^ viral particles of AAV8 containing either the target gene or scrambled vector via the tail vein 7 d before OVX model construction. Target gene expression was monitored by BLI through intraperitoneal injection of d-luciferin (150 μg g^−1^ body weight).

### Ovariectomy mouse model

For studies in vivo, mice were randomly divided by weight. For the ovariectomy-induced bone loss model, sham-operation or ovariectomy were performed in 8–12-week-old female mice. After weeks of treatment, femurs were isolated for μ-CT or histology analysis. Blood was collected for the CTX-1 test.

### Nano-LC-ESI–MS/MS analysis

Nano-LC–MS/MS with electrospray ionization (ESI) (Basic Medicine Public Technology Platform of SJTU-SM) was used to identify interacting proteins. In brief, sEVs containing the FLAG–LRG1 protein (sEVs–FLAG–LRG1) were purified from culture medium of AML12 cells stably transfected with Flag-tagged LRG1 expression. After co-culture with sEVs–FLAG–LRG1 for 48 h, BMDMs were collected, lysed and briefly sonicated at 4 °C. The supernatants (whole-cell lysates) were collected and incubated with Protein A/G PLUS-agarose (Santa Cruz, sc-2003) at room temperature for 1 h and then mixed protein lysates were subjected to IP with anti-FLAG M2 beads (Sigma). IP samples were separated by SDS–PAGE and visualized with colloidal Coomassie blue. The target lane from gels was prepared for analysis by LC–MS/MS. The MS spectrum was acquired using an Orbitrap Fusion LUMOS mass spectrometer (Thermo Fisher Scientific) connected to an Easy-nLC 1200 via an Easy Spray (Thermo Fisher Scientific). The MS analysis was conducted using the DAVID Bioinformatics database.

### Immunoprecipitation

After co-culture with sEVs–FLAG-LRG1 for 48 h, cells were collected, lysed and briefly sonicated at 4 °C. The supernatants (whole-cell lysates) were collected and incubated with Protein A/G PLUS-Agarose (Santa Cruz, sc-2003) at room temperature for 1 h and then incubated with anti-FLAG M2 beads (Sigma) at 4 °C overnight. The precipitates were washed five times with IP buffer (50 mM Tris-HCl, pH 7.6, 150 mM NaCl, 1 mM EDTA, 1% NP-40, 1 mM phenylmethylsulfonyl fluoride and 1× protease inhibitor cocktail (Calbiochem)), boiled in sample buffer and subjected to western blot analysis.

### Nucleocytoplasmic separation

The nuclear and cytoplasmic extracts from cells were obtained using an NE-PER Nuclear Cytoplasmic Extraction Reagent kit (Thermo Fisher Scientific) according to the manufacturer’s instructions. In brief, cells were treated with RANKL and sEVs for 24 h and then cells were collected. The ice bath CERI was added to the cell precipitation. The cells were fully suspended by vortex, put into an ice bath for 10 min and then ice bath CER II was added. Cells were vortexed for 5 s, placed in an ice bath for 1 min, vortexed for 5 s, then centrifuged at 4 °C for 15,000*g* for 5 min. The supernatant was collected and put into the ice bath; this provided the cytoplasmic protein. The ice bath NER was added to the precipitation, vortexed for 15 s and placed in an ice bath for 10 min. This process required 4–5 cycles, then the sample was centrifuged at 4 °C for 15,000*g* for 5 min and the supernatant was collected; this provided the protein of the nucleus. The samples were subjected to western blot analysis.

### Immunofluorescence

For in situ immunofluorescence, murine femurs were fixed in 4% paraformaldehyde at 4 °C for 6 h and rinsed in PBS three times and then dehydrated with 20% sucrose and embedded in optimal cutting temperature compound (OCT). Femoral hemisection was prepared in a cryostat (Leica Microsystems) at −24 °C and rinsed in PBS five times to remove OCT. The hemisection femur was permeabilized in 0.5% Triton X-100 at room temperature for 30 min, rinsed in PBS three times and blocked in 5% bovine serum albumin (BSA) at room temperature for 1 h. The hemisection femur was incubated overnight at 4 °C with mouse monoclonal anti-CTSK antibody (1:100 dilution, Santa, sc-48353) or rabbit polyclonal anti-LRG1 antibody (1:50 dilution, ABclonal, A7850) in a 500-µl micro-centrifuge tube. Then, the hemisection femur was rinsed in PBS and incubated with Texas red goat anti-mouse IgG H+L antibody (1:1,000 dilution, Abcam, ab6787), Alexa Fluor 488 donkey anti-rabbit IgG (H+L) antibody (1:1,000 dilution, Thermo Fisher Scientific, A-21206) and 1:1,000 dilution DAPI (Sigma) overnight at 4 °C and examined under a Nikon Laser Confocal Scanning Microscope. Quantitation of the ratio of LRG1 and CTSK-double-positive areas to CTSK-positive areas on bone sections was measured by ImageJ (Media Cybernetics). For immunofluorescence to identify the interaction of p65 with LRG1, BMDMs were seeded on coverslips in 24-well plates overnight. After treatment with RANKL and GFP-labeled LRG1-sEVs for 48 h, cells were rinsed with PBS three times and fixed in 4% paraformaldehyde at room temperature for 10 min. Next, coverslips were rinsed in PBS three times and permeabilized in methanol at 4 °C for 10 min. Then, coverslips were rinsed in PBS three times and blocked in 1% BSA at room temperature for 1 h. Target protein p65 location was detected by incubating with primary antibodies (1:100 dilution, Cell Signaling Technology, 8242) overnight at 4 °C in a humid chamber. After washing three times, secondary antibodies (1:200 dilution, Thermo Fisher Scientific, Alexa Fluor 594, A-21207) were applied in a 1:200 dilution in staining buffer for 1 h at 37 °C in a humid chamber. After washing, coverslips were mounted with Vector shield with DAPI (Vector Laboratories) and analyzed on a Nikon Laser Confocal Scanning Microscope. Quantitation of ratio of nucleus-p65/total p65 was measured by ImageJ. For immunofluorescence of BMDMs from aged mice, BMDMs were fixed on the slide with a cytospin machine and the subsequent steps were similar to those described for cellular immunofluorescence. The primary antibody was rabbit polyclonal anti-p65 antibody (1:100 dilution, Cell Signaling Technology, 8242) and the secondary antibody was Alexa Fluor 488 donkey anti-rabbit IgG (H+L) antibody (1:200 dilution, Thermo Fisher Scientific, A-21206). Quantitation of ratio of nucleus-p65/total p65 was measured by ImageJ. For immunofluorescence to identify the interaction of LPHN2 with LRG1, HEK293T cells were seeded on coverslips in 24-well plates overnight, the LPHN2 plasmid was transfected into HEK293T cells and treated with GFP-labeled LRG1-sEVs for 24 h. The subsequent steps were similar to those described for cellular immunofluorescence. The primary antibody was rabbit polyclonal anti-LPHN2 antibody (1:100 dilution, Abcam, ab139498) and the secondary antibody was Alexa Fluor 594 donkey anti-rabbit IgG (H+L) antibody (1:200 dilution, Thermo Fisher Scientific, A-21207).

### Primary cultures of human peripheral blood mononuclear cells

Human PBMCs were isolated from healthy donors by Ficoll gradient centrifugation. Written informed consent was obtained from all donors. PBMCs were cultured in ɑ-MEM with 10% FBS (Sigma), 1% GlutaMAX Supplement (Thermo Fisher) and 30 ng ml^−1^ human CSF-1 (Sino Biological, 11792-H08Y) and incubated in a humidified atmosphere at 37 °C and 5% CO_2_. For osteoclastogenesis, 5 × 10^5^ PBMCs were seeded in a 12-well plate, after 48 h, cells were stimulated with 50 ng ml^−1^ human RANKL (R&D, 6449–TEC-010) and 30 ng ml^−1^ human CSF-1 for 10–14 d. Medium was changed every 2 d. Osteoclasts were fixed and stained using the TRAP staining kit (Sigma-Aldrich, 387A-1KT).

### Denosumab administration and cessation

Isolated human PBMCs were seeded on 12-well plates. Inhibition experiments were performed by the administration of RANKL and sEVs or the commercial denosumab (Amgen). The final concentration of denosumab was 500 ng ml^−1^ as previously reported^58^. Medium was changed every 2 d. After 10 d of treatment, half of PBMCs were stained by TRAP and the inhibitory effects of sEVs or denosumab were analyzed. At the same time, for the other half of PBMCs, both sEVs and denosumab administration was stopped, but PBMCs continued to be treated with RANKL for 4 d to compare the rebound effect after cessation of sEVs and denosumab.

### Patient liver tissues, blood samples and human bone mineral densities

A total of 114 margin non-tumor tissues were collected from surgically resected liver tissues with hepatocellular carcinoma from The Affiliated Hospitals of Youjiang Medical University for Nationalities between January 2018 and May 2022. Before samples were collected, we obtained Institutional Review Board approval and the written informed consent from the human participants. The amount of SIRT2 protein expression in these liver tissue samples were tested using IHC as previously described^20^. The study participants were divided into two groups, namely LAG (age <51 years, *n* = 54) and HAG (age >51 years, *n* = 60). The correlations between different expression levels and ages were analyzed by Spearman’s correlation test. Patient blood samples were collected from 120 patients aged 60–70 years between 2018 and 2021 at Shanghai General Hospital. The whole blood of the patients was extracted and placed in an anticoagulant tube at 4 °C. Plasmas were obtained with a refrigerated centrifuge (1,000*g* for 15 min) and were stored at −80 °C. The concentrations of β-CTX, PINP and BALP were analyzed in the Department of Laboratory Medicine in Shanghai General Hospital. Plasma sEVs were extracted by ultracentrifugation; centrifuged at 300*g* for 10 min, 2,000*g* for 10 min, 10,000*g* for 30 min and then centrifuged at 110,000*g* for 90 min (Beckman Ultra high-speed refrigerated centrifuge). The separated plasma sEVs were found in the precipitate and were resuspended by 1× SDS. Protein levels of plasma sEV-LRG1 of all 120 patients were detected by western blot and quantified by gray scanning. The sEV-LRG1 expression was presented as the ratio of LRG1 grayscale value to TSG101 grayscale value. The sEV samples of 120 patients were named nos. 1–120. To make the results of different western blots comparable, the no. 1 sample from a patient with healthy BMD was selected as a control. Each western blot analysis included no. 1 and the other 12 samples. The sEV-LRG1 expression of the 12 other samples in each analysis were calculated using no. 1 as a control. Next, the correlations of the expression of plasma sEV-LRG1 and BMD, β-CTX, PINP or BALP were analyzed. BMDs were measured by dual-energy X-ray absorptiometry. BMD was analyzed in three categories: healthy, osteopenia and osteoporosis, based on the World Health Organization T score classification (osteoporosis was defined as T score <−2.5, osteopenia was defined as T score between −1.0 and −2.5 and T score >−1.0 was considered as healthy). All participants were patients who needed spinal surgery and met the clinical indications for BMD measurement. And participants underwent standardized preoperative examinations and had no history of CLD, diabetes, tumor or other organ diseases. The study was approved by the Medical Ethic Committee of SJTU-SM and was conducted in accordance with ethical principles of the World Medical Association and Declaration of Helsinki. Informed written consent was given by all patients before this study. In the comparison of the protein level of plasma sEV-LRG1 between the healthy BMD group and patients with osteoporosis, only female patients (*n* = 53) meeting the diagnosis were included because there were no male patients diagnosed with osteoporosis in our collected data. In the correlation analysis between the expression of plasma sEV-LRG1 and BMD, β-CTX, PINP or BALP, due to the small number of male patient samples, both female and male patients were included for statistical analysis.

### Statistics and reproducibility

Statistical analyses were performed using Prism 9 (GraphPad Software) and data are presented as mean ± s.d. A Kolmogorov–Smirnov test, Anderson–Darling test, D’Agostino–Pearson omnibus test or Shapiro–Wilk test were used to test normality. The assumptions of homogeneity of error variances were tested using an *F*-test (*P* > 0.05). An unpaired two-tailed Student’s *t*-test was used to determine significance between two groups of normally distributed data. Welch’s correction was used for groups with unequal variances. An unpaired two-tailed Mann–Whitney *U*-test was used to determine significance between data without a normal distribution. For comparisons between multiple groups, an ordinary one-way or two-way ANOVA was used, followed by Tukey’s test. For comparison between multiple groups with two fixed factors, an ordinary two-way ANOVA was used, followed by Tukey’s multiple-comparisons test. The Spearman’s correlation test was used to evaluate the correlation between SIRT2 expression and aging. The correlations of plasma sEV-LRG1 level and BMD, β-CTX, PINP and BALP were analyzed using linear correlation and regression. Differences were considered significant when *P* < 0.05 or *P* > 0.05 with large differences of observed effects^59,60^. In vitro experiments were performed with two or three biological replicates. Biological replicates refer to the experiments conducted by the same treatment between samples of different biological individuals or different biological groups, whereas technical replicates refer to multiple identical experiments conducted on the same sample. All specific statistical details and the number of biological or technical replicates can be found in the figure captions and statistical data.

### Reporting summary

Further information on research design is available in the Nature Portfolio Reporting Summary linked to this article.

## Supplementary information

Supplementary Information Additional images of replicates.

Reporting Summary

Supplementary Tables Primers and antibodies.

## Data Availability

The Gene Expression Omnibus accession number for the RNA-seq data is GSE228204. The mass spectrometry proteomics data have been deposited to the ProteomeXchange Consortium (http://proteomecentral.proteomexchange.org) via the iProX partner repository^61^ with the dataset identifier PXD041145. Materials, reagents or other experimental data are available upon request. Source data are provided with this paper.

## Source data

Source Data Fig. 1 Statistical source data and unprocessed western blots.
Source Data Fig. 2 Statistical source data.
Source Data Fig. 3 Statistical source data and unprocessed western blots.
Source Data Fig. 4 Statistical source data and unprocessed western blots.
Source Data Fig. 5 Statistical source data and unprocessed western blots.
Source Data Fig. 6 Statistical source data and unprocessed western blots.
Source Data Fig. 7 Statistical source data and unprocessed western blots.
Source Data Extended Data Fig. 1 Statistical source data.
Source Data Extended Data Fig. 2 Statistical source data.
Source Data Extended Data Fig. 3 Unprocessed western blots.
Source Data Extended Data Fig. 4 Statistical source data.
Source Data Extended Data Fig. 5 Statistical source data and unprocessed western blots.
Source Data Extended Data Fig. 6 Statistical source data.
Source Data Extended Data Fig. 7 Statistical source data and unprocessed western blots.
Source Data Extended Data Fig. 8 Statistical source data.
Source Data Extended Data Fig. 9 Statistical source data and unprocessed western blots.
Source Data Extended Data Fig. 10 Statistical source data and unprocessed western blots.
