# Supplementary material for: SIRT2 regulates extracellular vesicle-mediated liver–bone communication
Source: Nat Metab. 2023 May 15;5(5):821–41. doi: 10.1038/s42255-023-00803-0 (PMC10229428; doi:10.1038/s42255-023-00803-0)

nature metabolism

Article

https://doi.org/10.1038/s42255-023-00803-0

SIRT2 regulates extracellular vesicle-mediated liver–bone communication

In the format provided by the authors and unedited

Figure.1a Western blot analysis of SIRT2 protein expression in primary hepatocytes of aged female and male mice

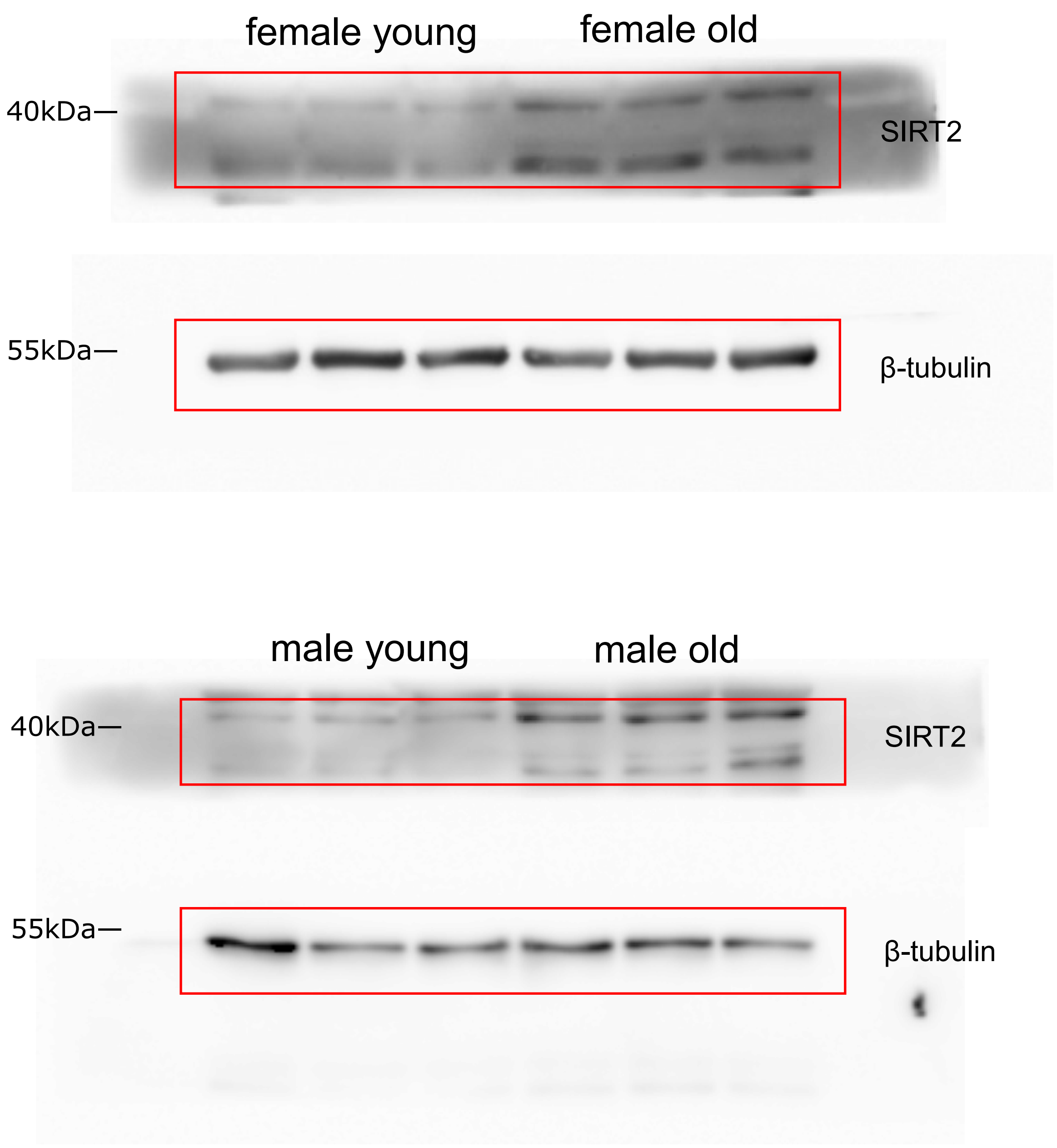

Figure.1c IHC images of SIRT2 protein expression in liver tissues from young and aged mice

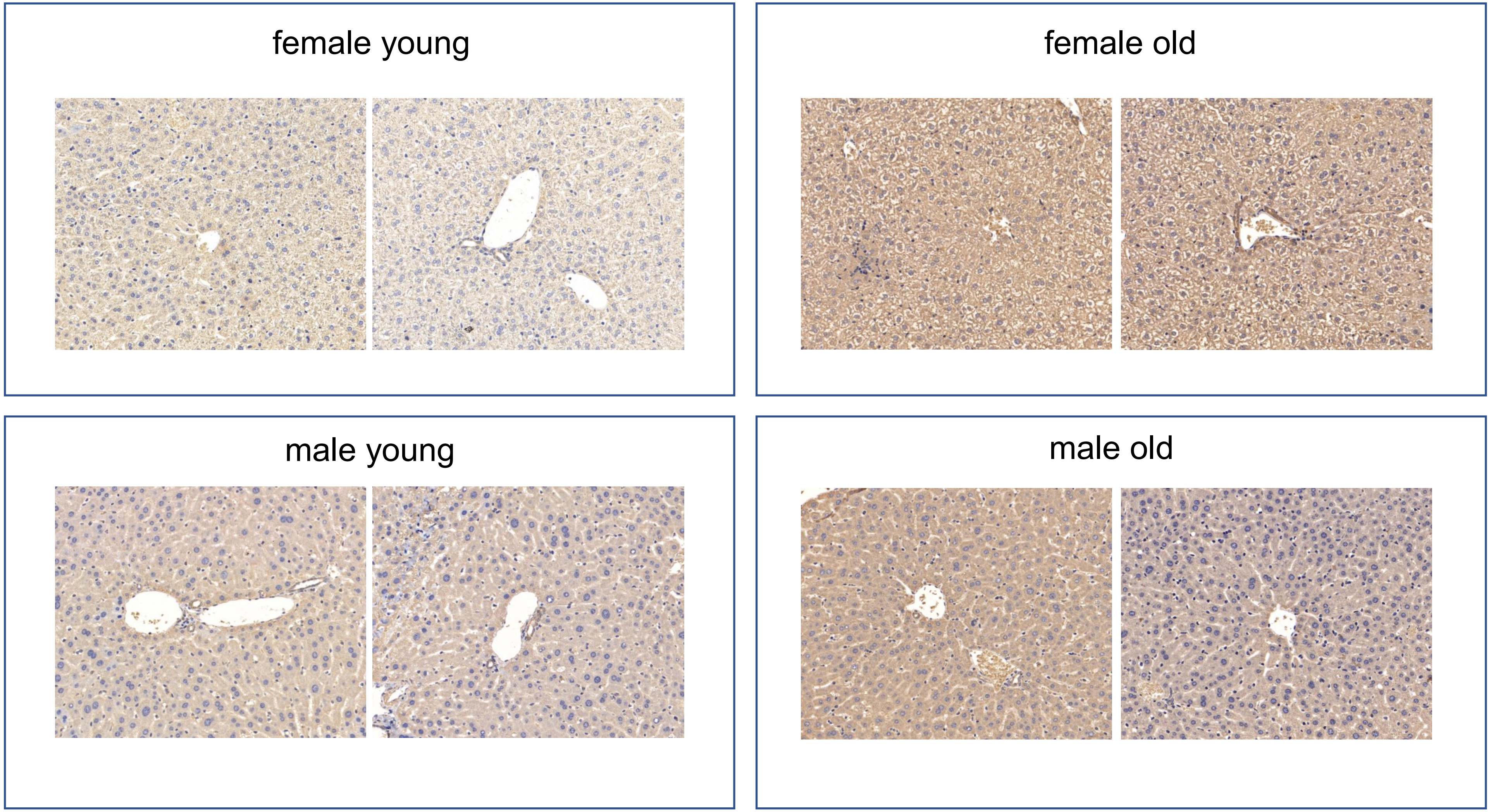

Figure.1e

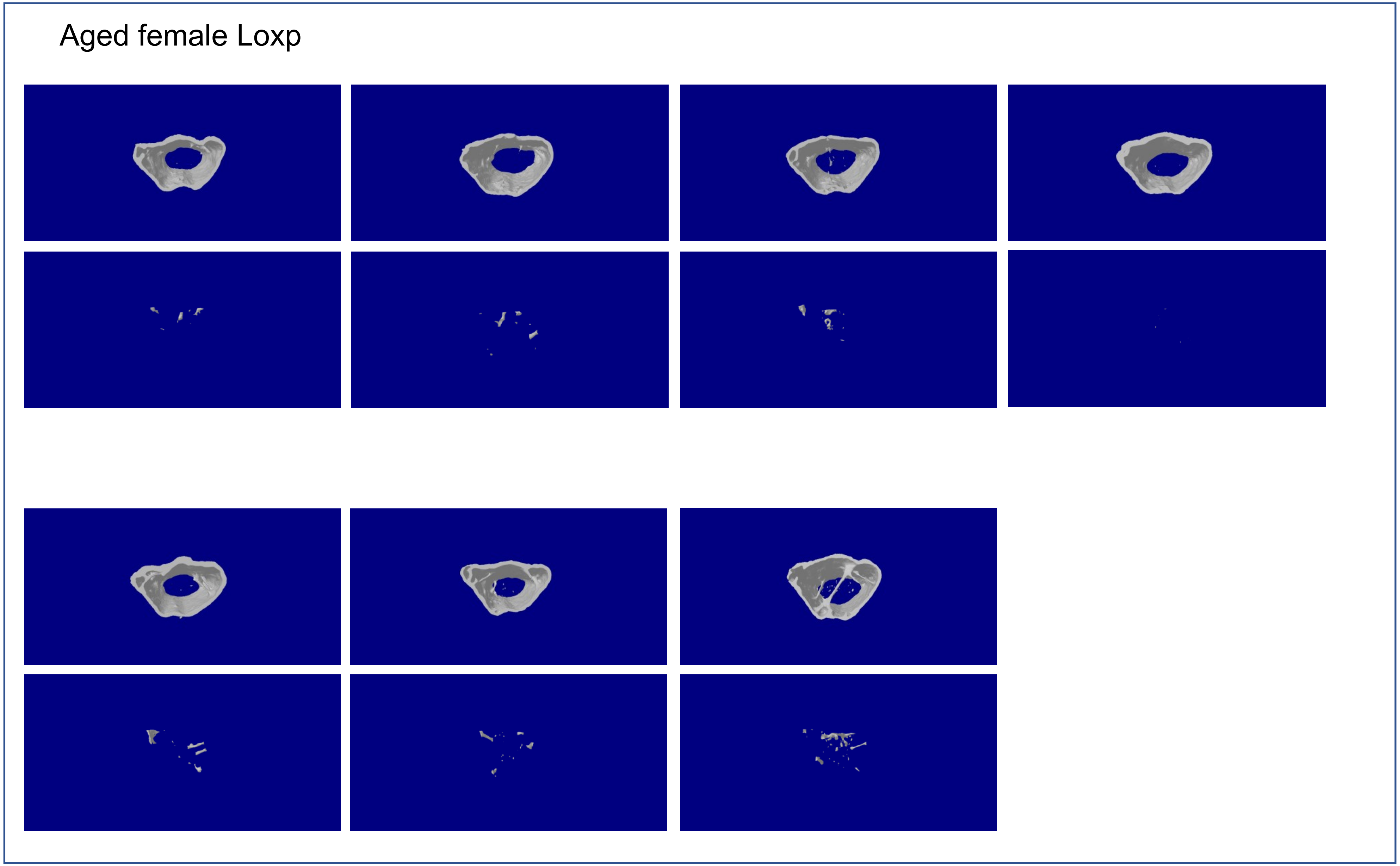

Figure.1e

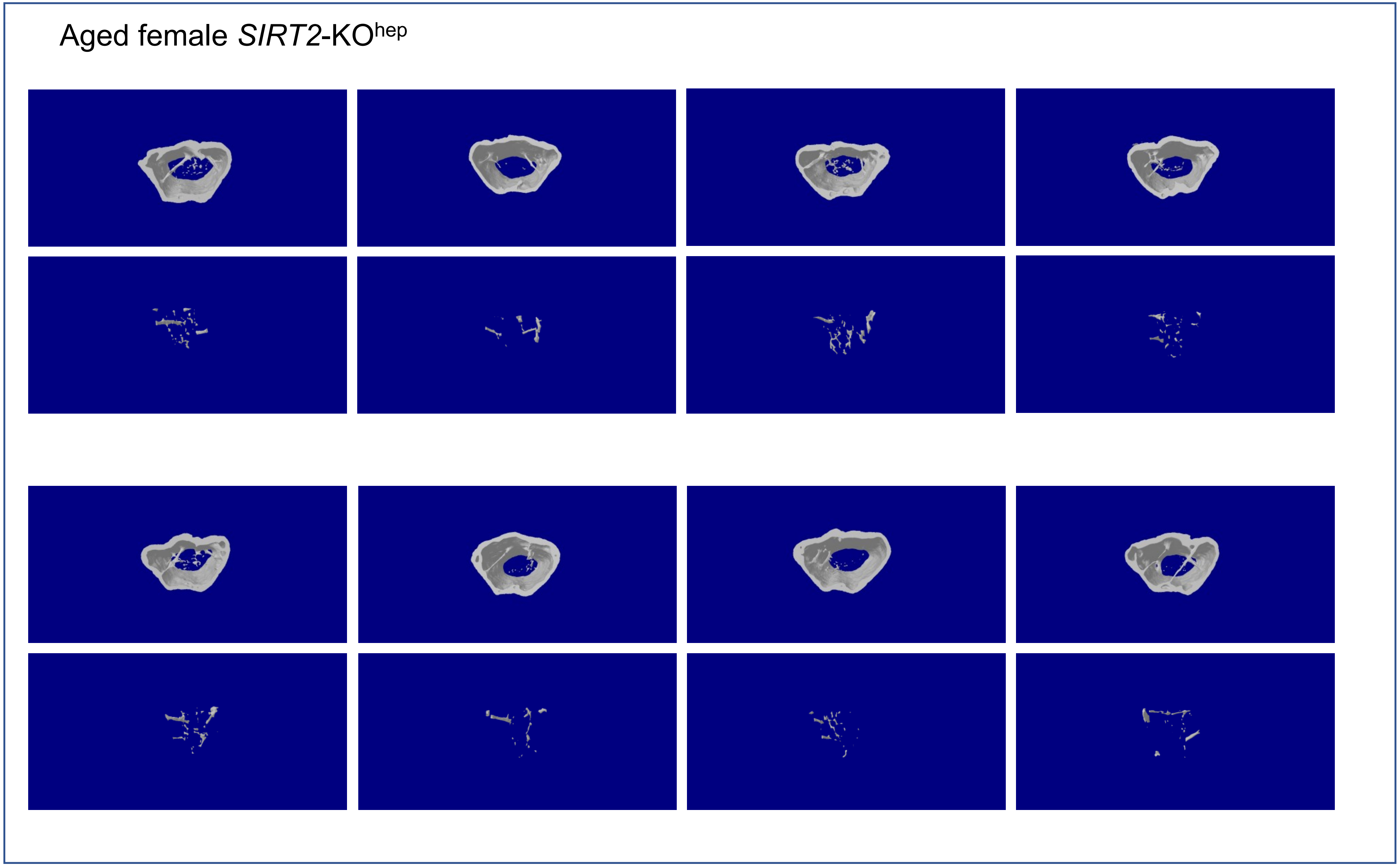

Figure.1g

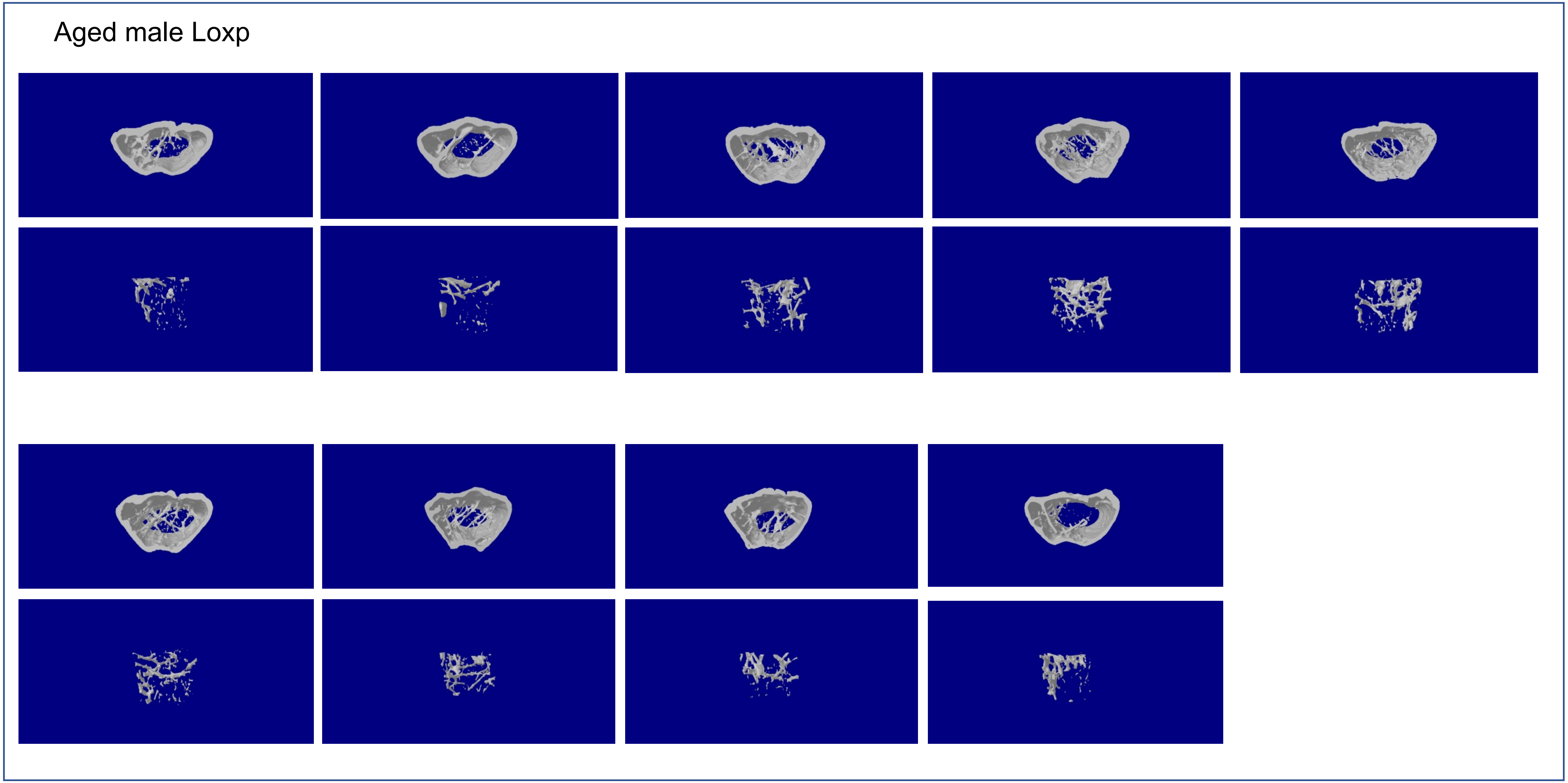

Figure.1g

Aged male *SIRT2*-KO^{hep}

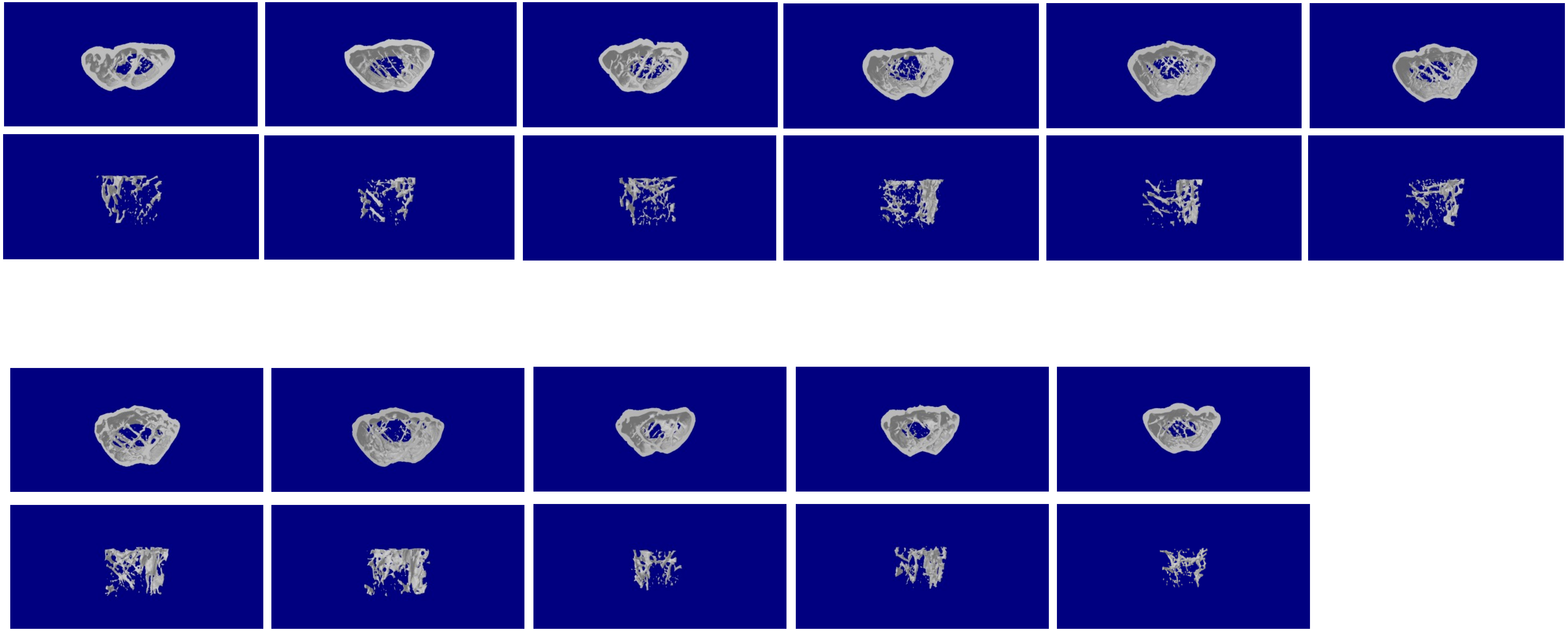

Figure.1k TRAP staining on paraffin-embedded femur sections in aged mice

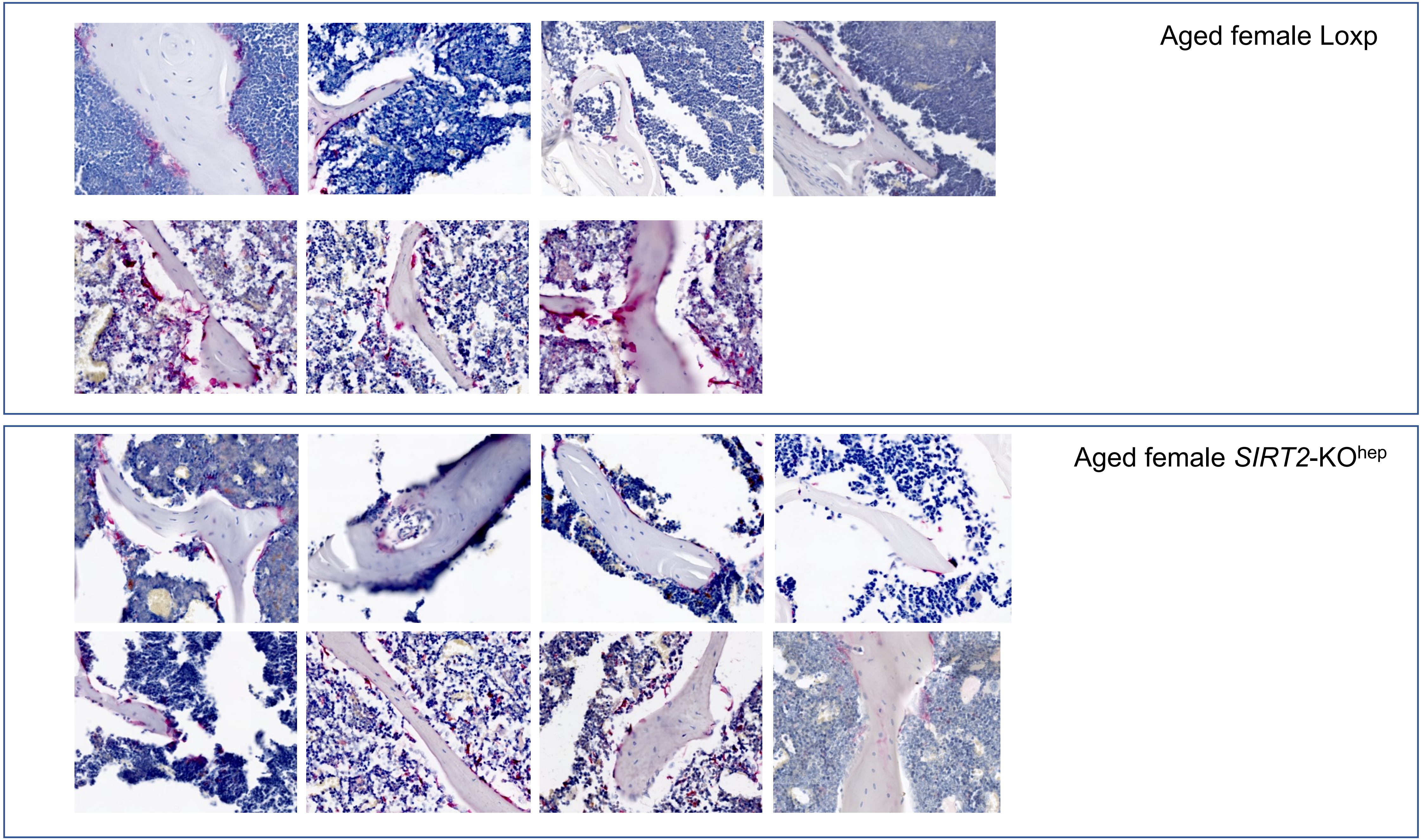

Figure.1m TRAP staining on paraffin-embedded femur sections in aged mice

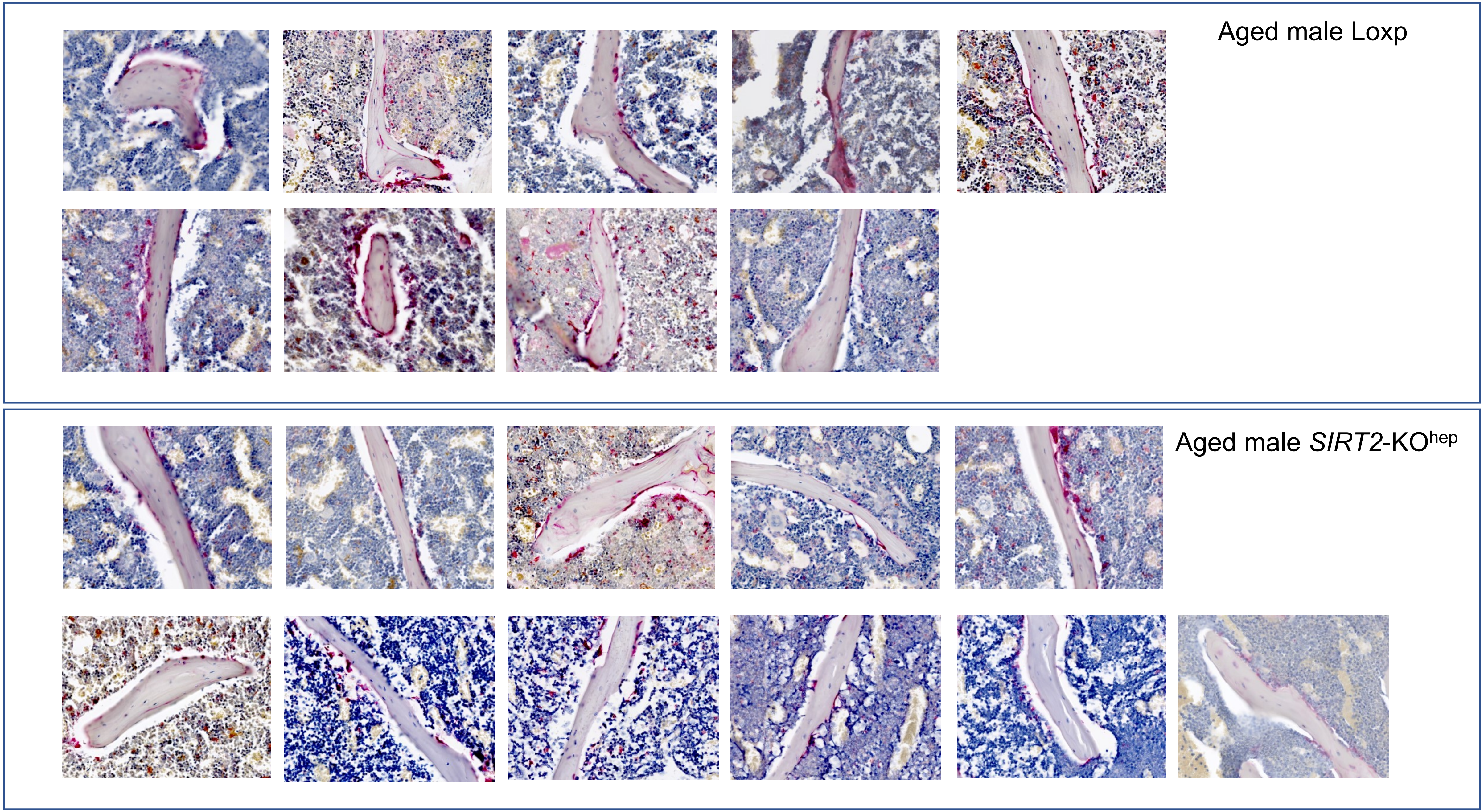

Figure.2a TRAP staining images of osteoclasts administered with the plasma of aged mice

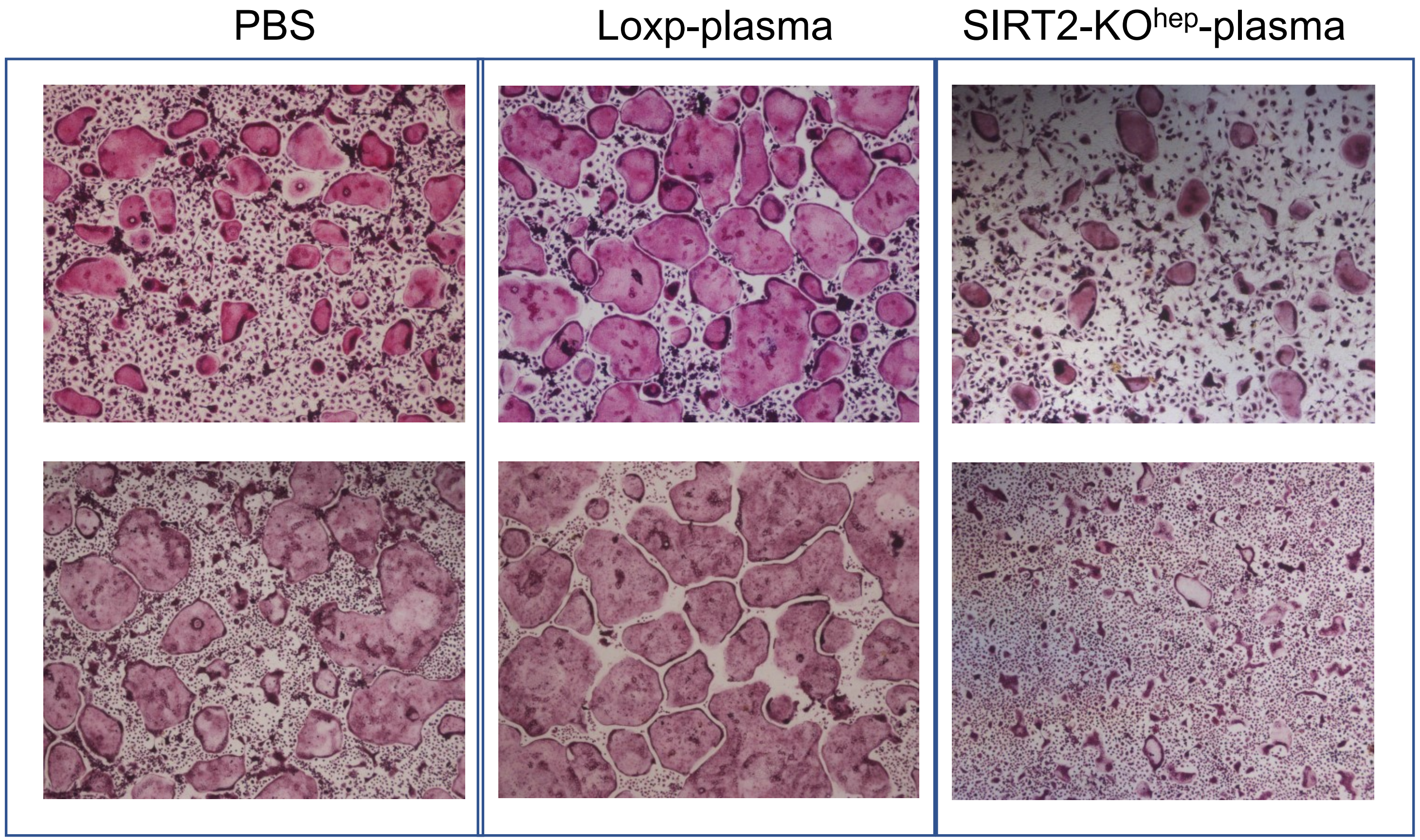

Figure.2e TRAP staining images of osteoclasts treated with the sEVs derived from LoxP or SIRT2-KOhep plasma

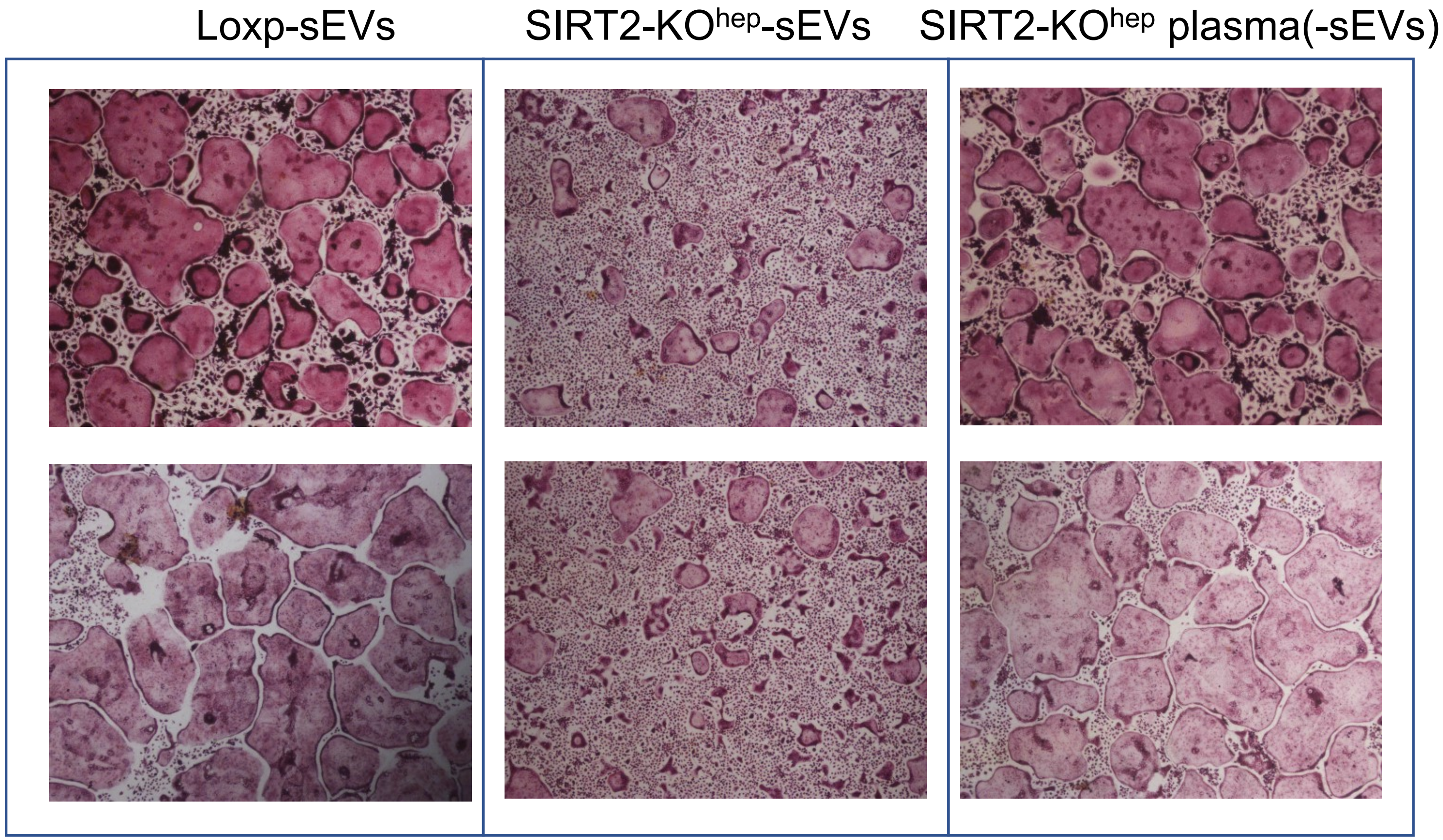

Figure.2i TRAP staining of osteoclasts treated with sEVs derived from the medium of the primary hepatocytes of aged female mice

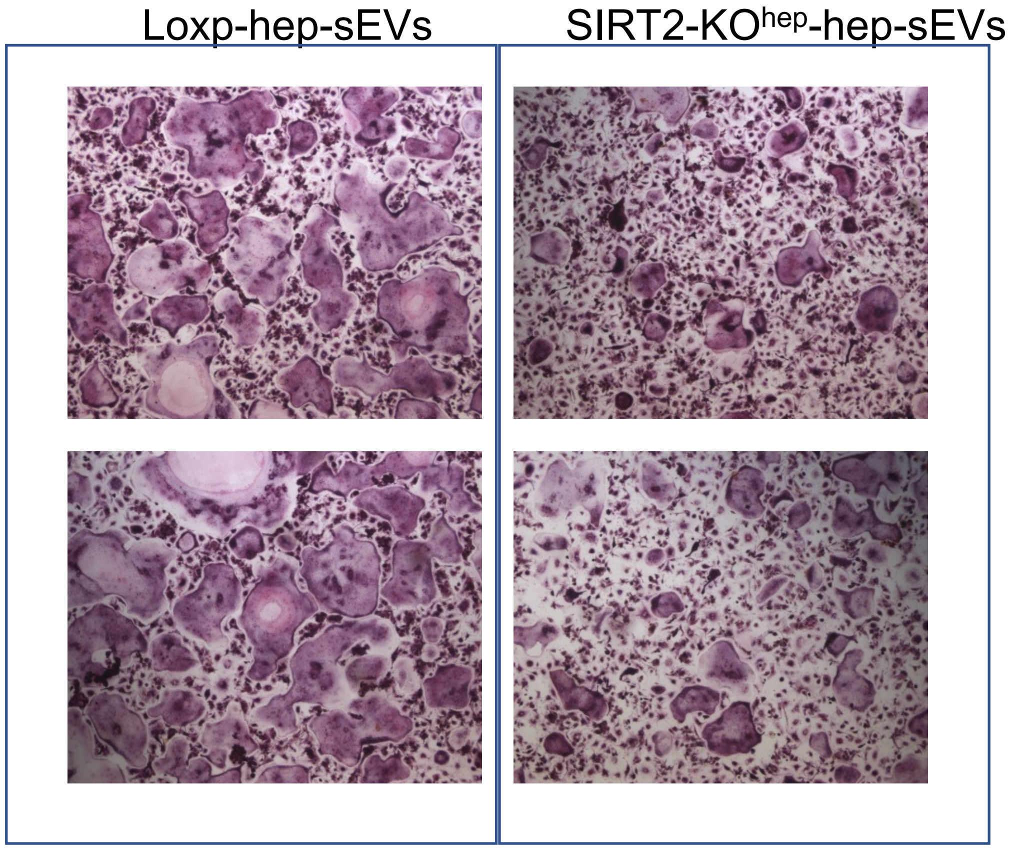

Figure.2I TRAP staining of osteoclasts treated with sEVs derived from the medium of the primary hepatocytes of aged male mice

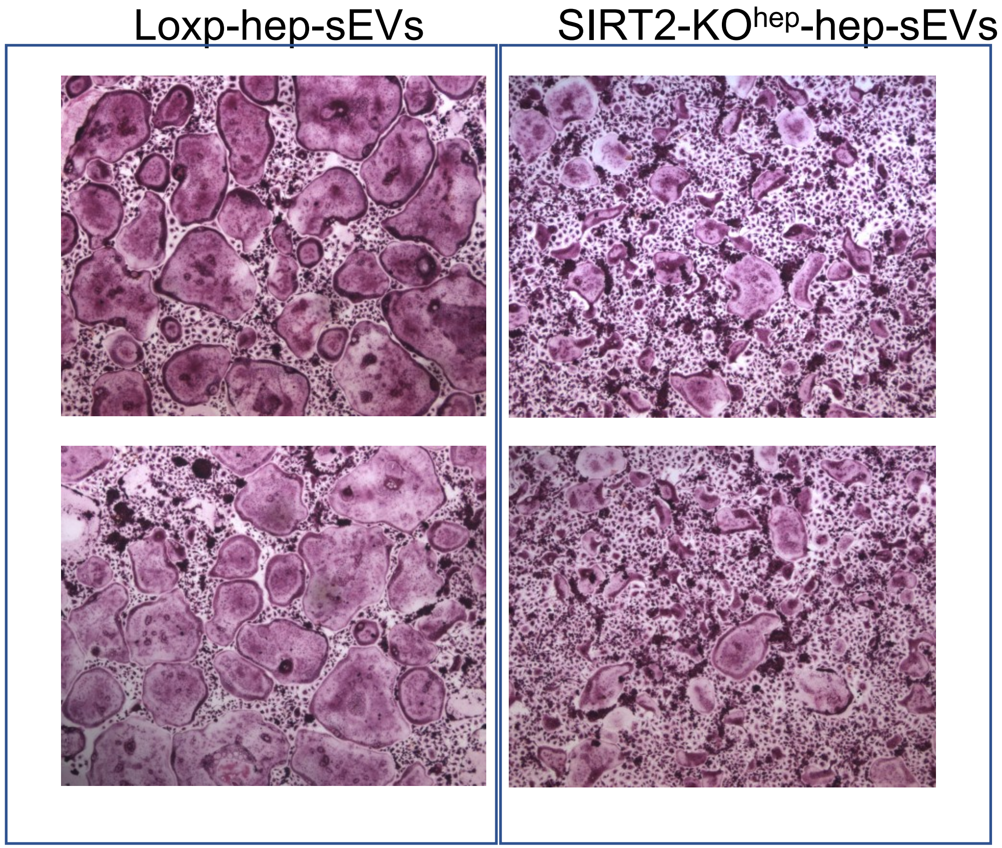

Figure.3e immunofluorescence images of murine femurs in aged mice

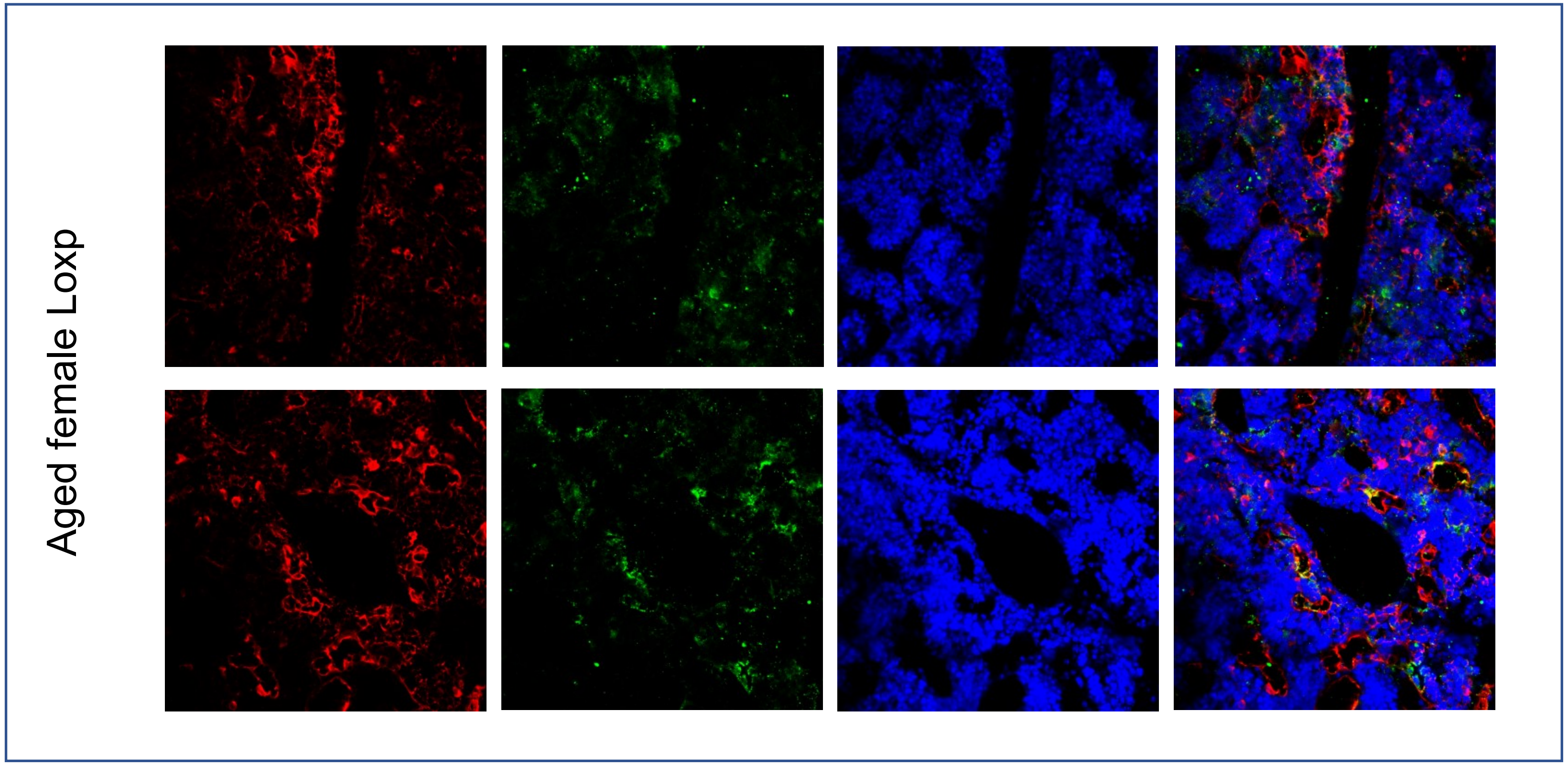

Figure.3e immunofluorescence images of murine femurs in aged mice

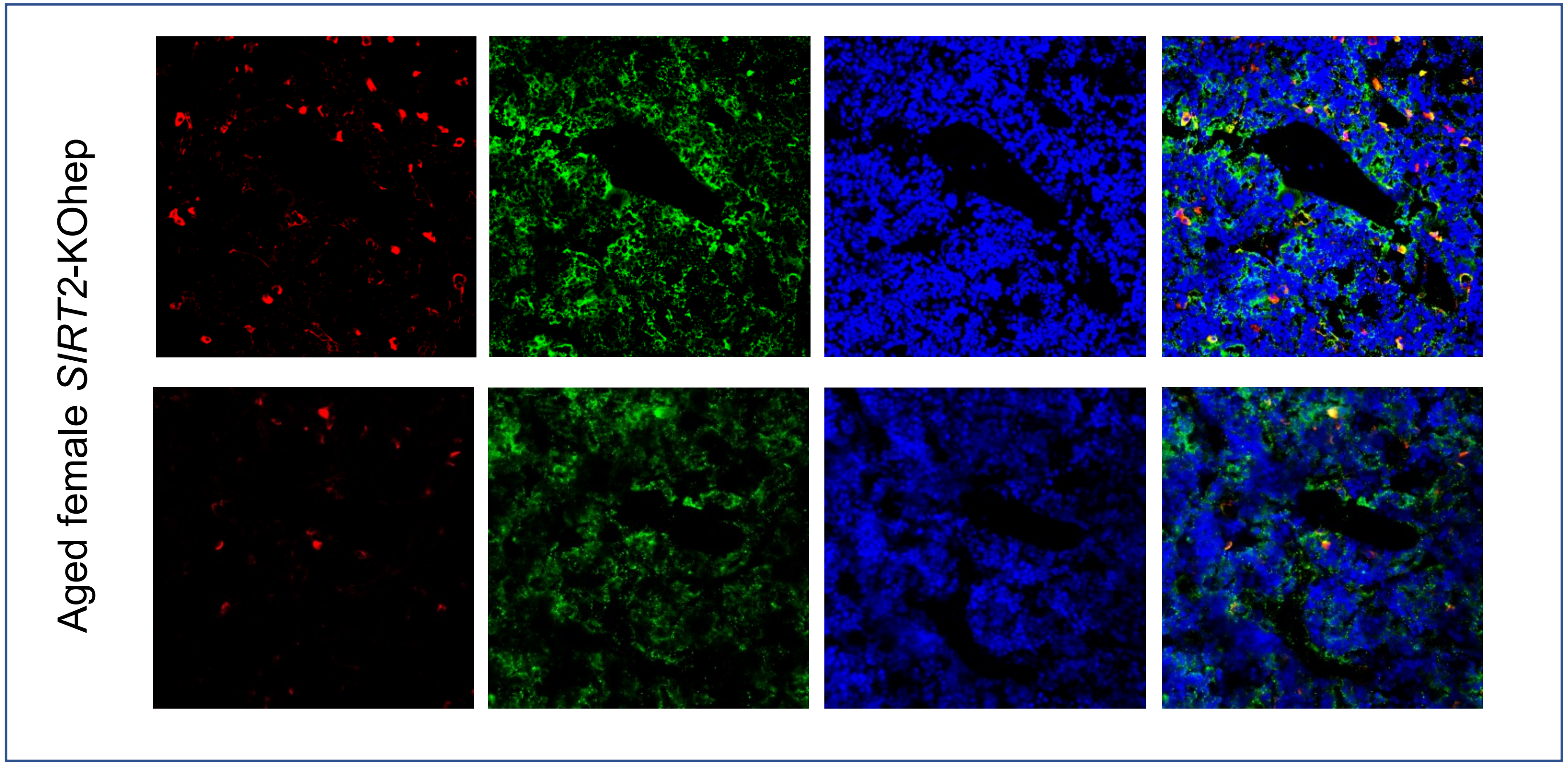

Figure.3g immunofluorescence images of murine femurs in aged mice

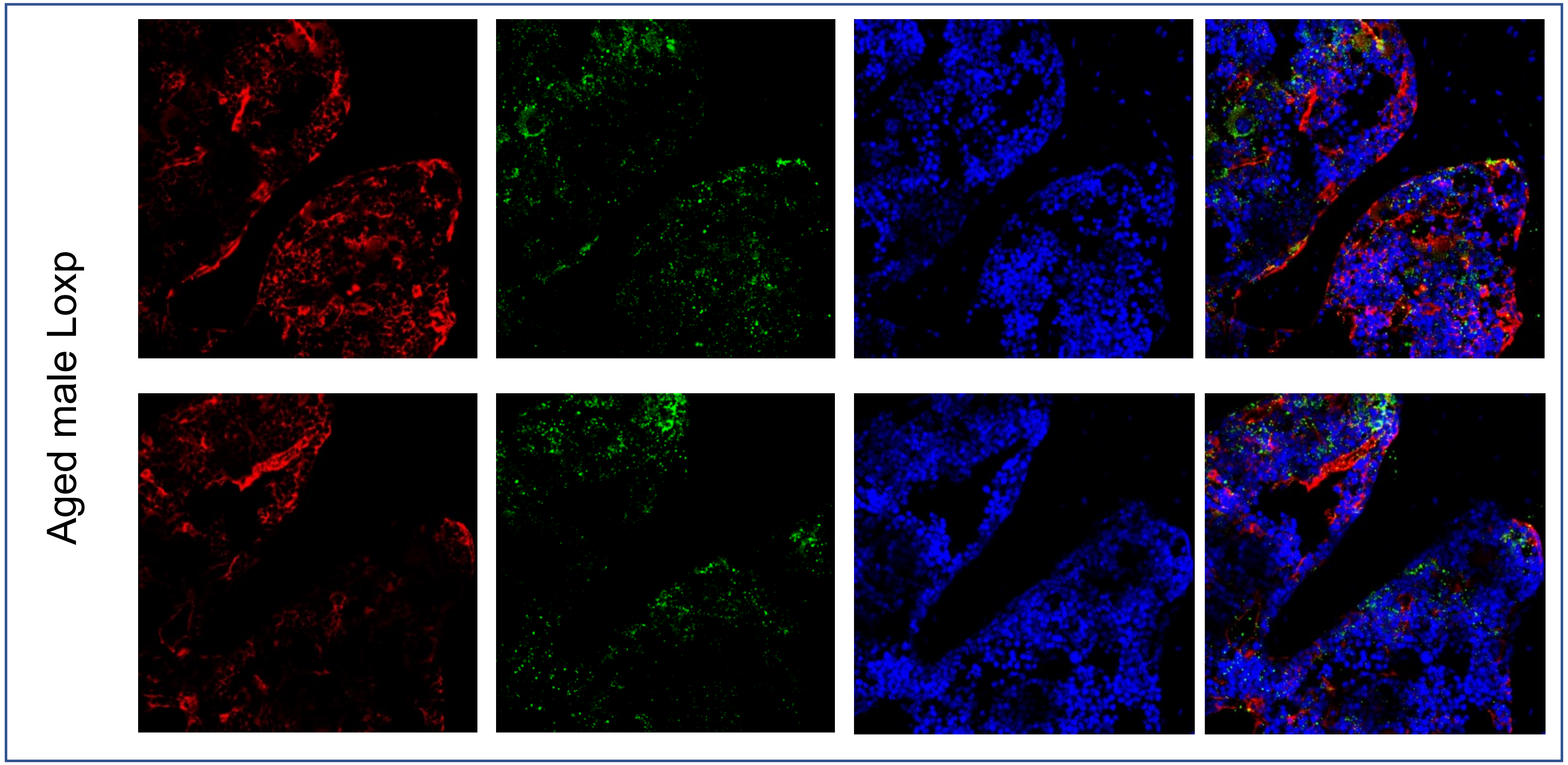

Figure.3g immunofluorescence images of murine femurs in aged mice

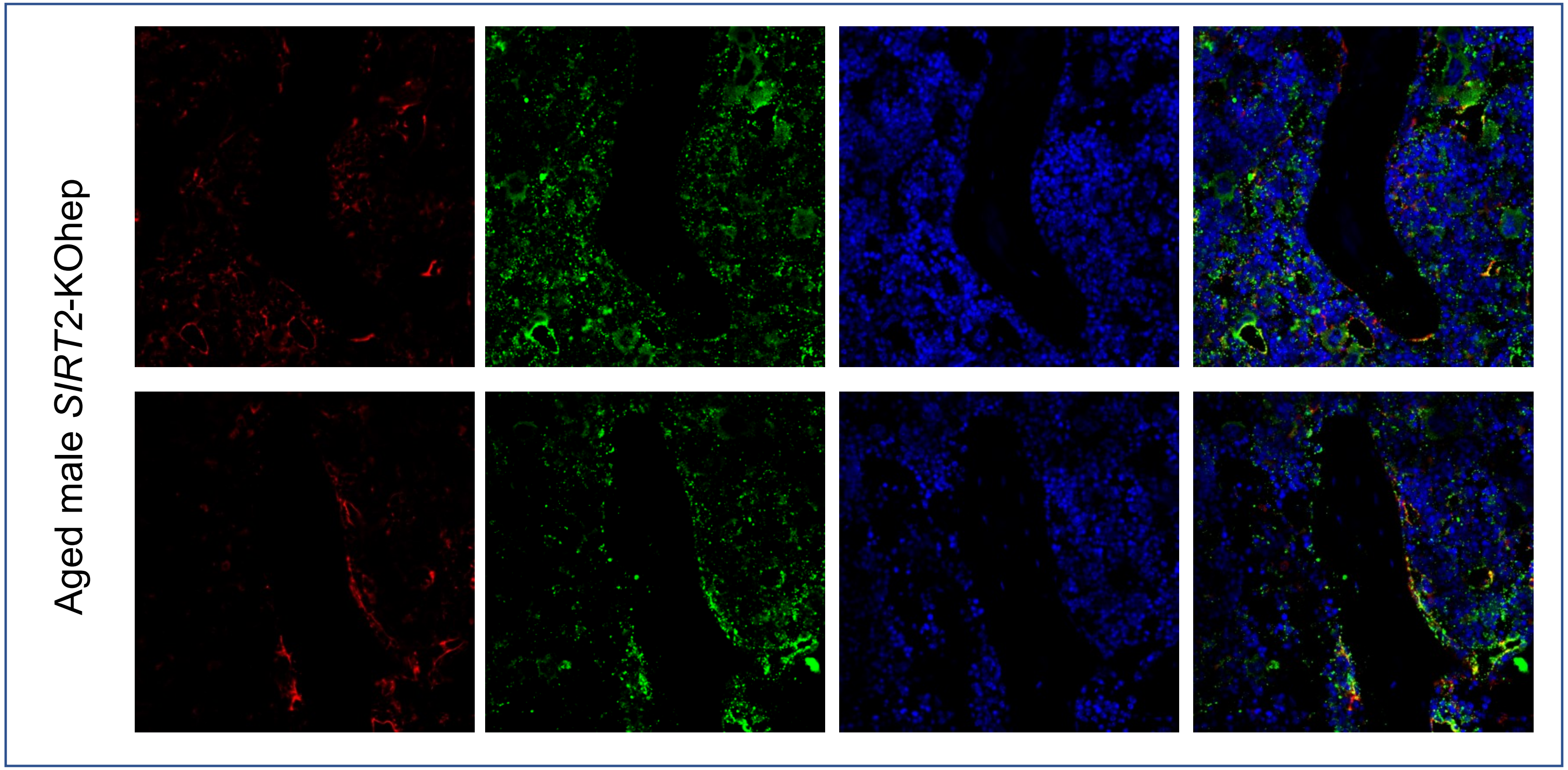

Figure.3m

sham Loxp-ctrl

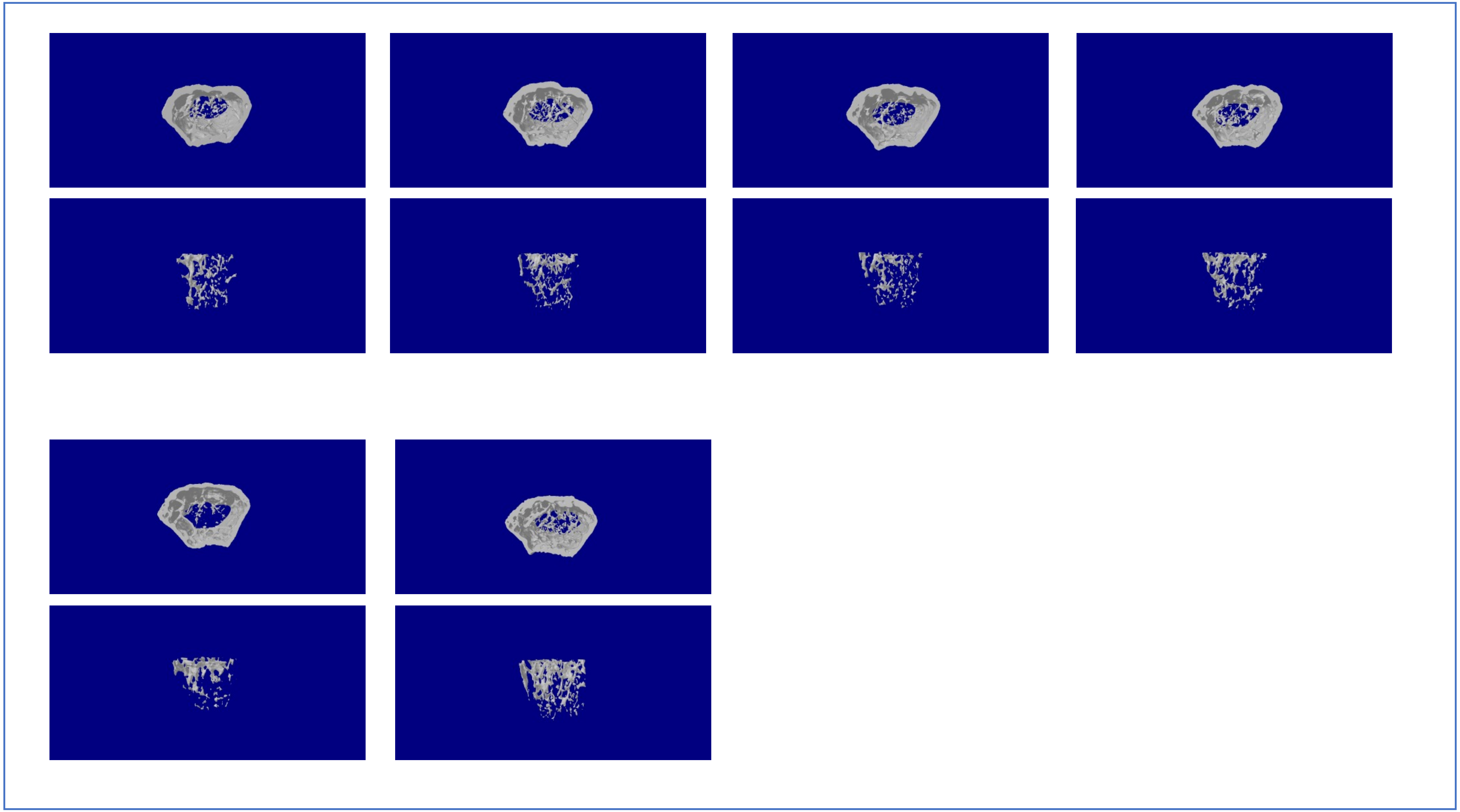

Figure.3m

sham SIRT2-KO^{hep}-ctrl

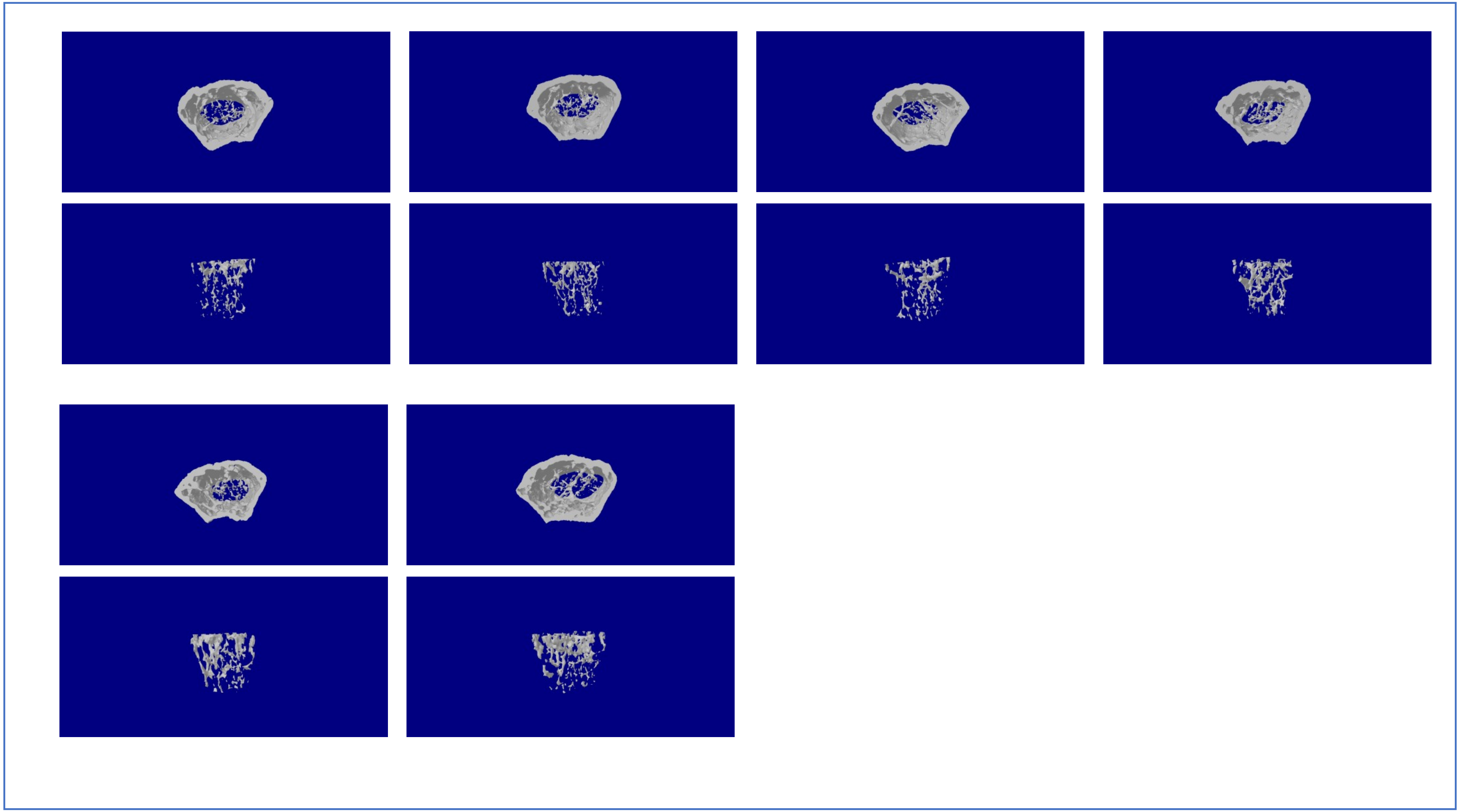

Figure.3m

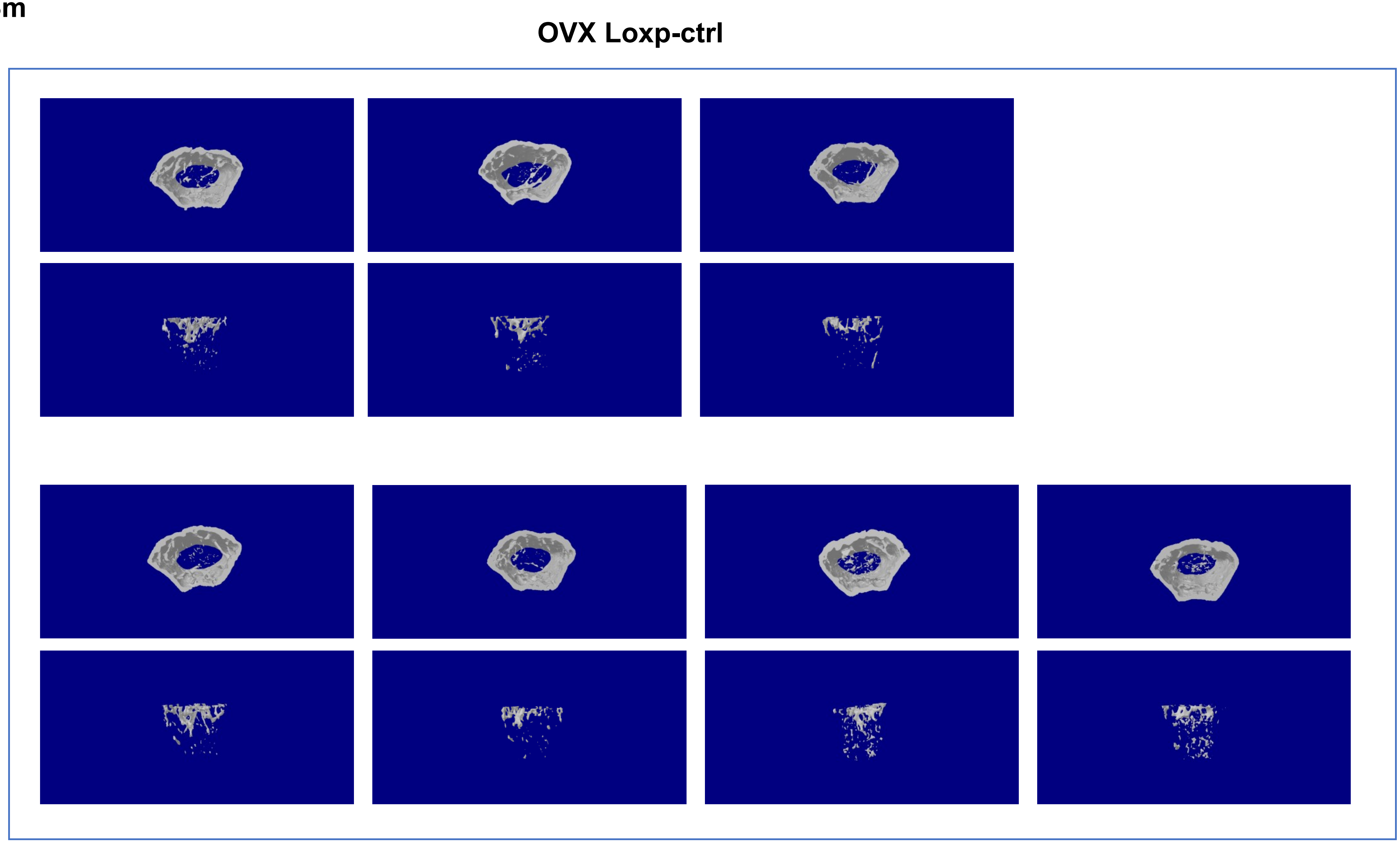

Figure.3m

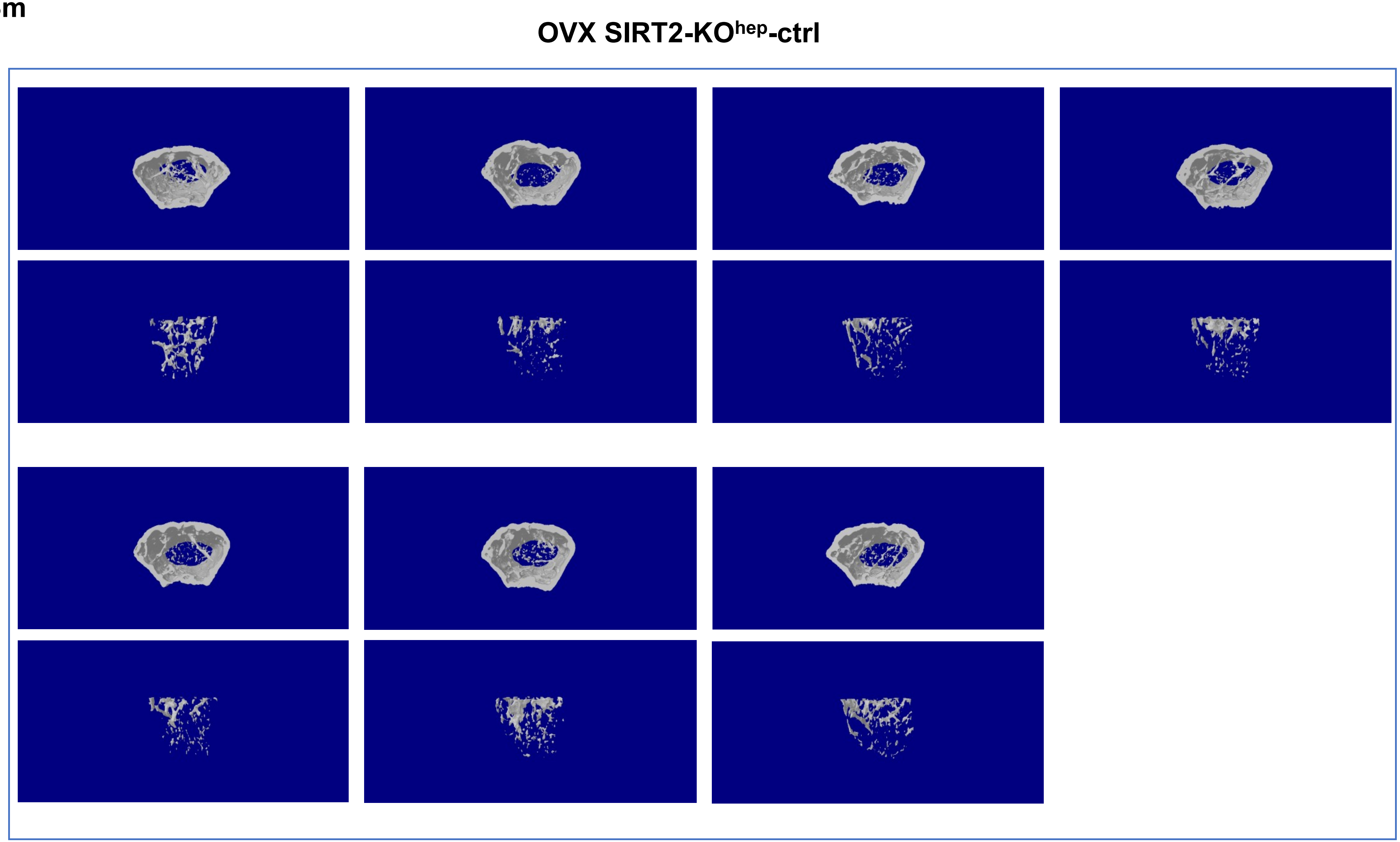

Figure.3m

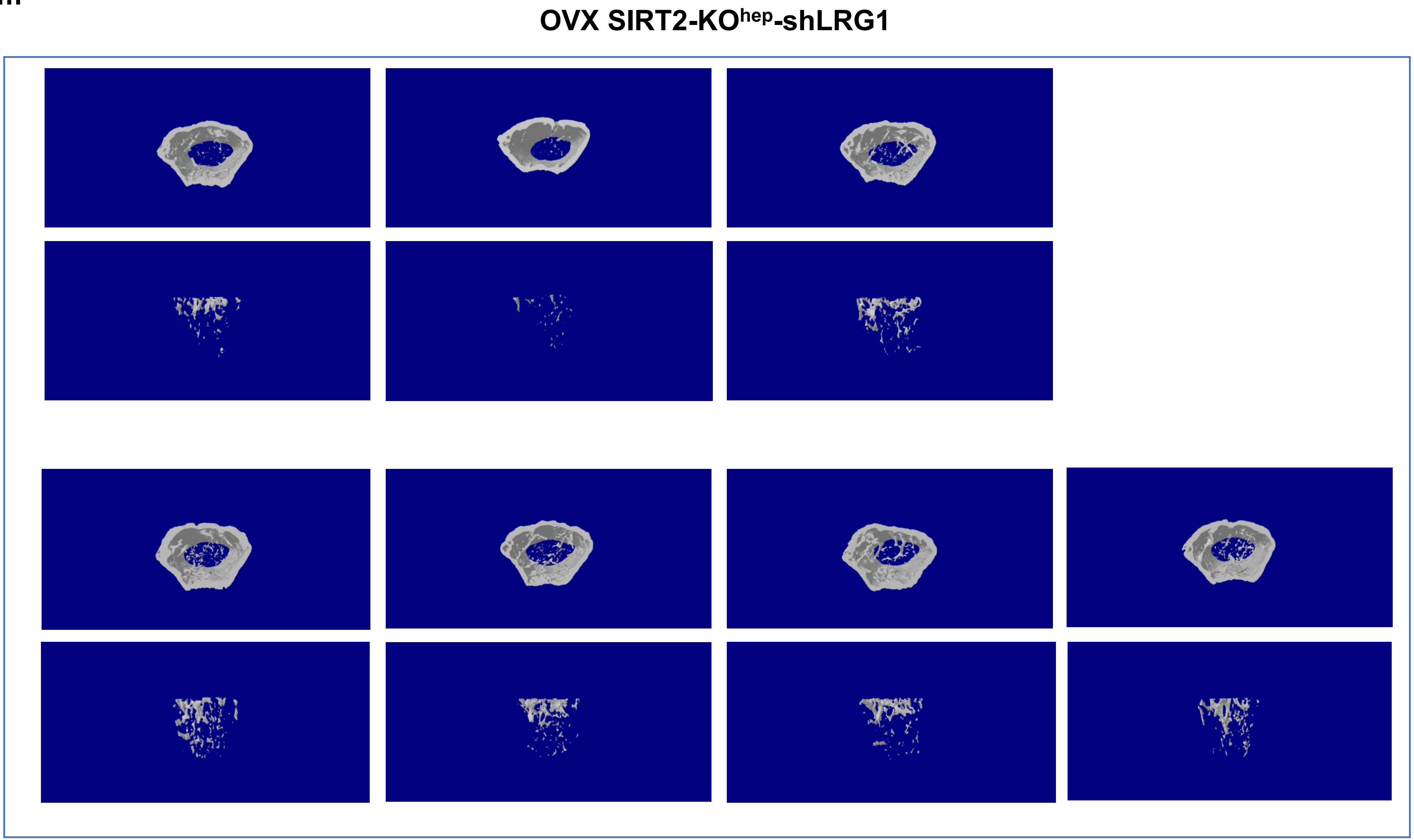

Figure.4b TRAP staining of osteoclasts treated with sEVs from AML-12 cells

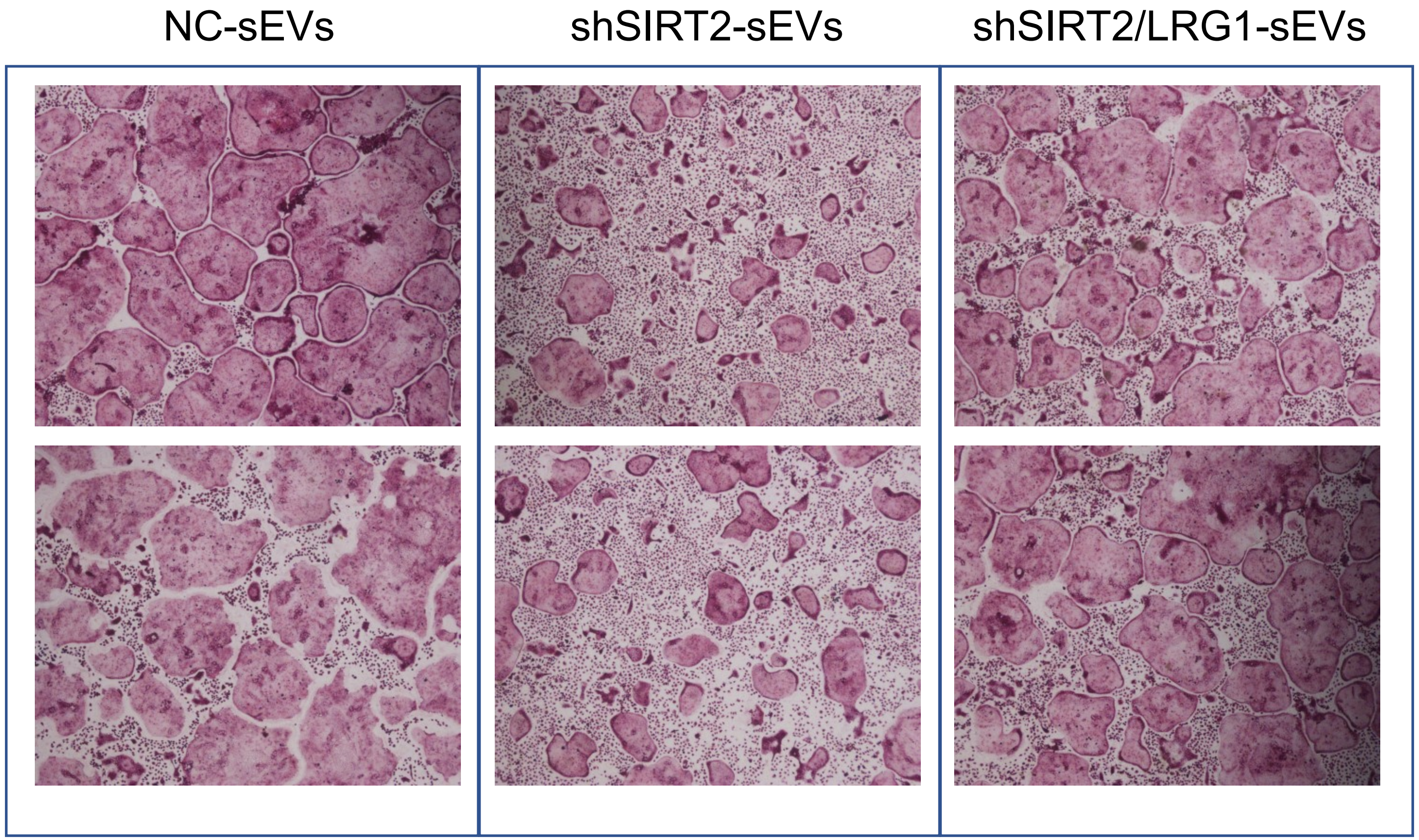

Figure.4h

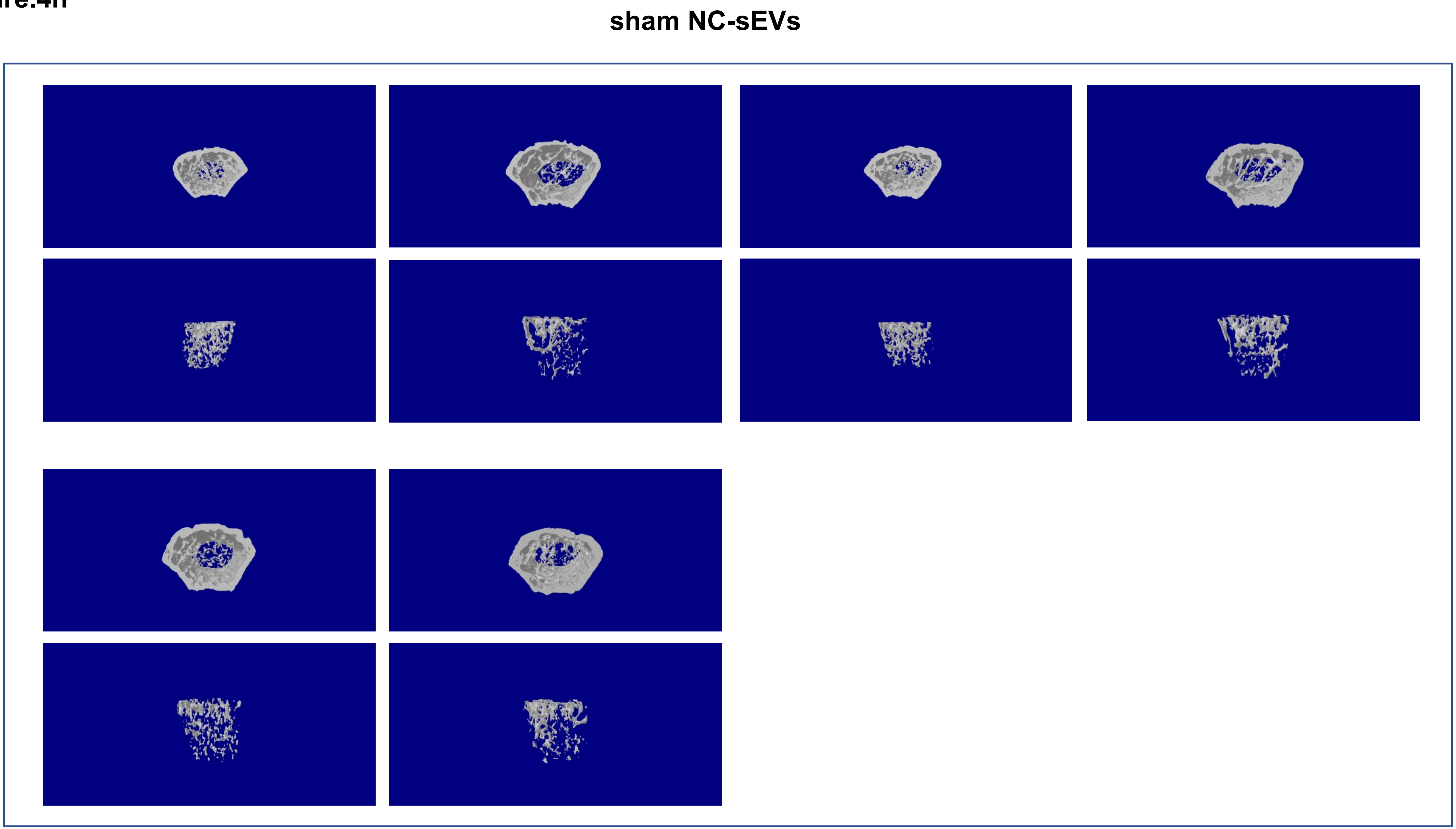

Figure.4h

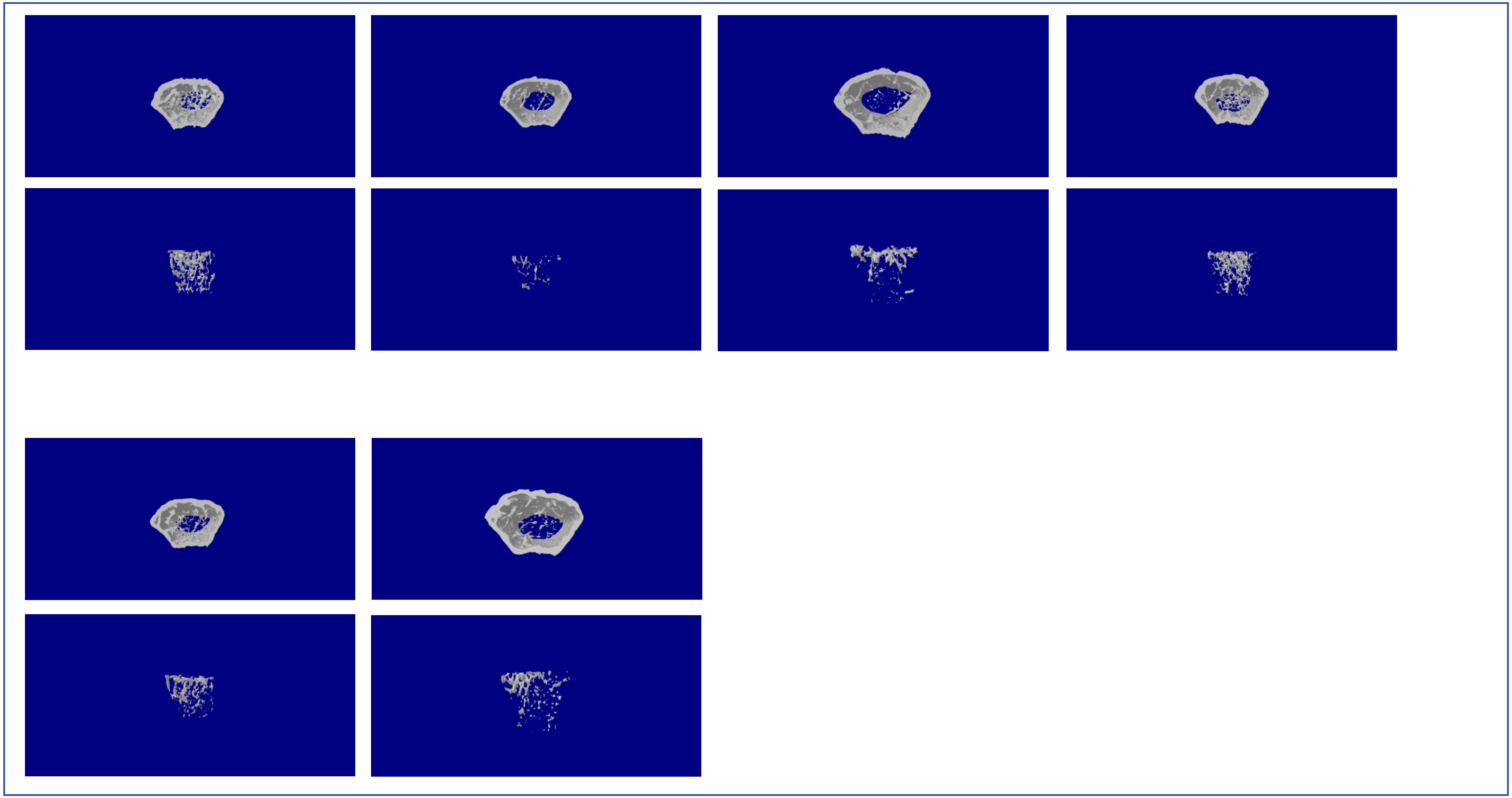

Figure.4h

OVX shSIRT2-sEVs

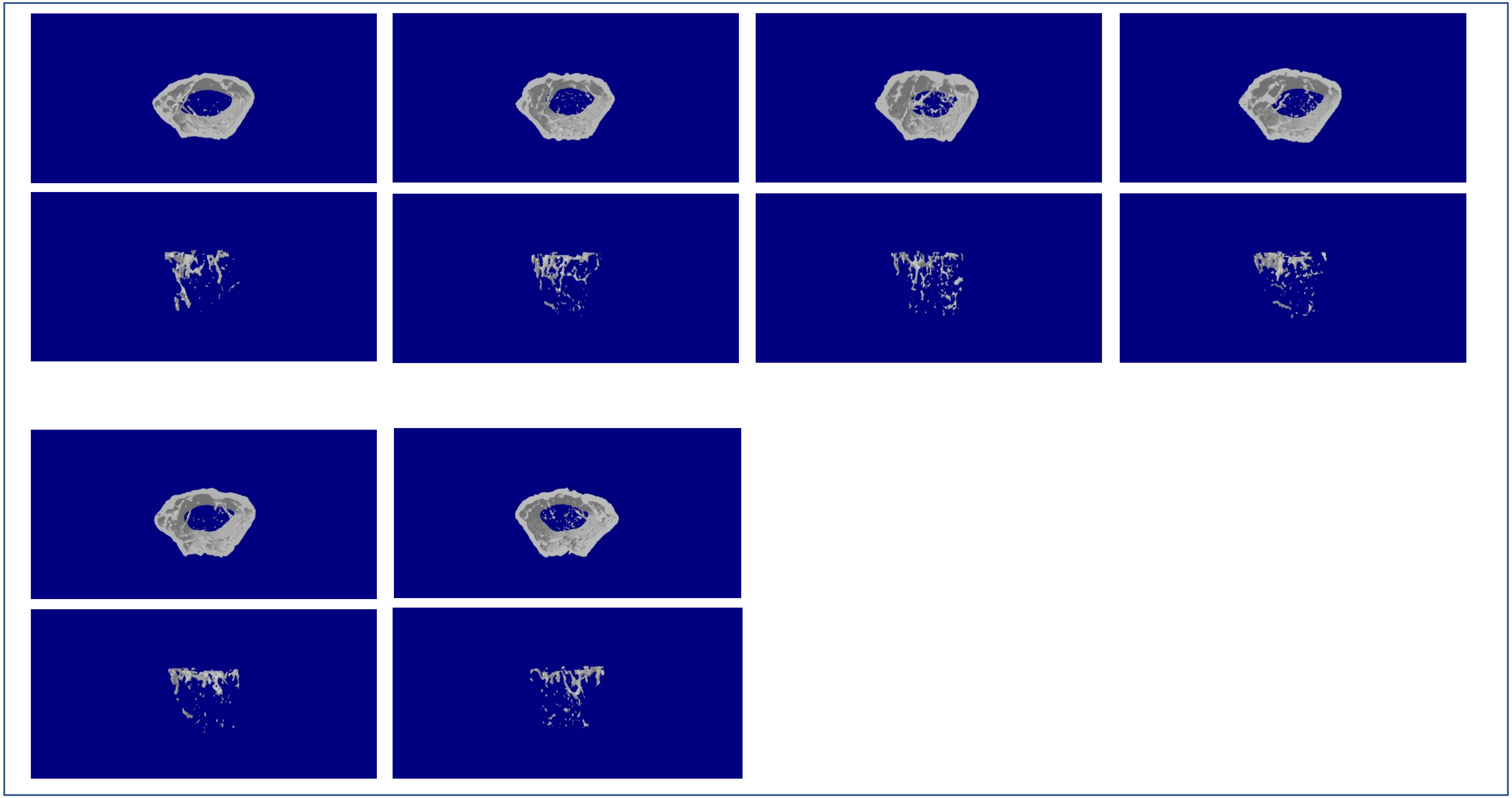

Figure.4h

OVX shSIRT2-shLRG1-sEVs

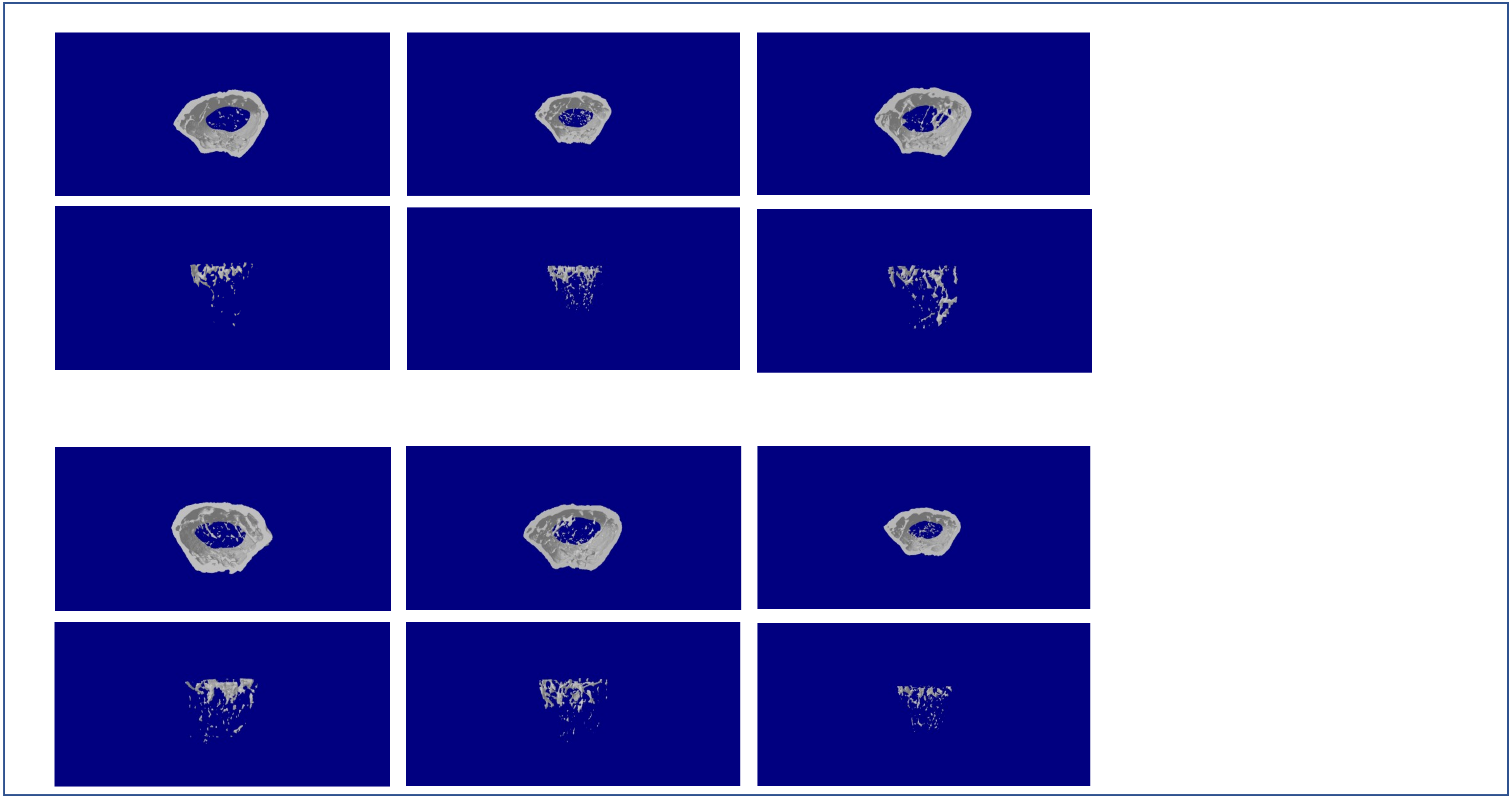

Figure.4h

OVX LRG1-sEVs

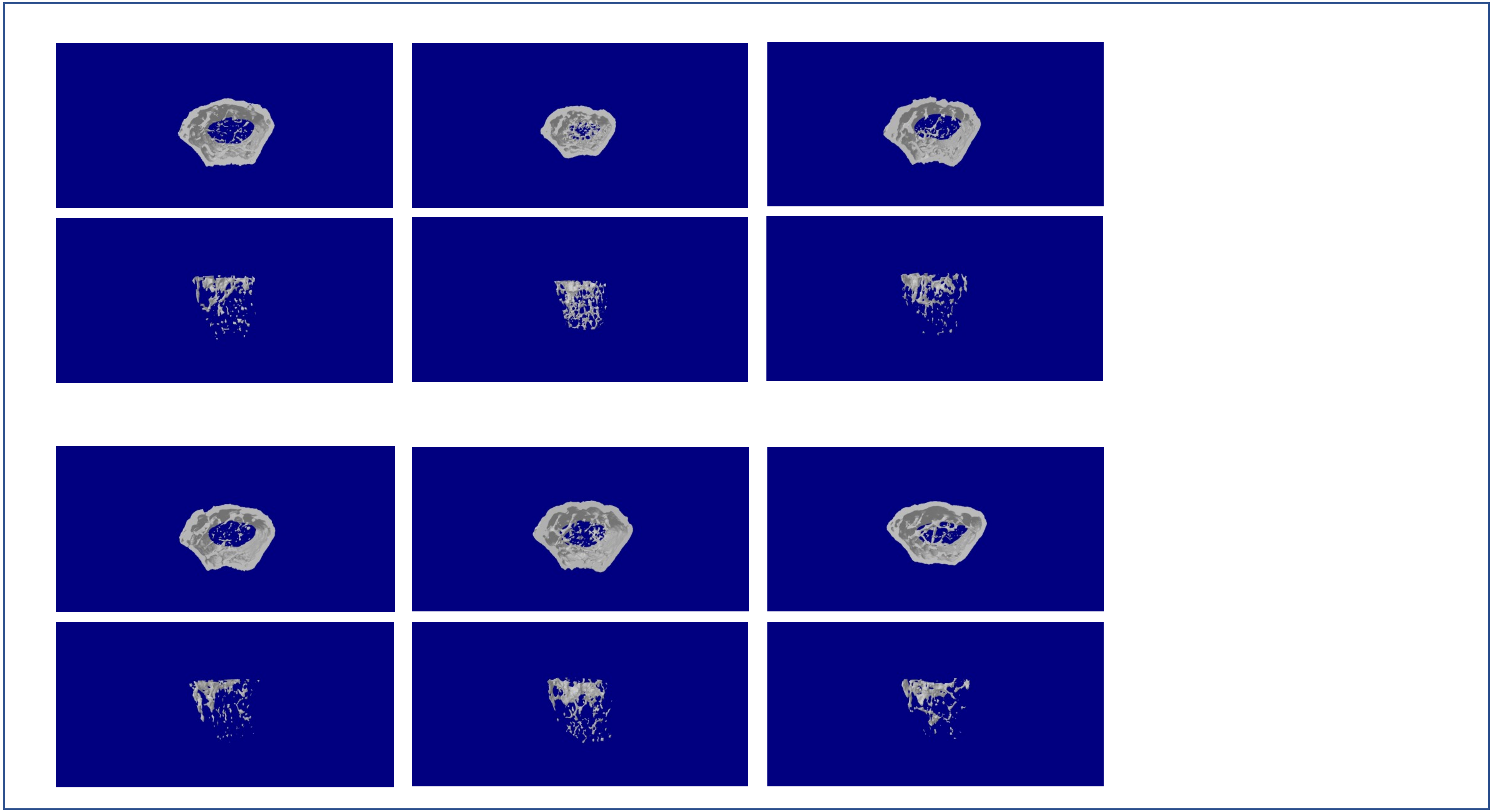

Figure.4k TRAP staining on paraffin-embedded femur sections in each group after corresponding sEVs treatment

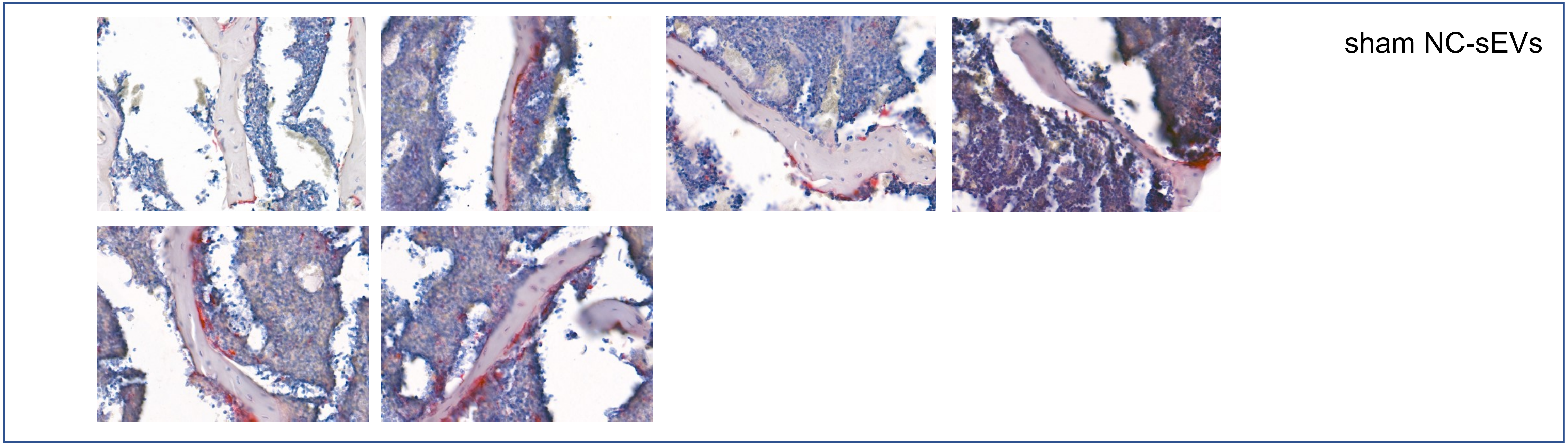

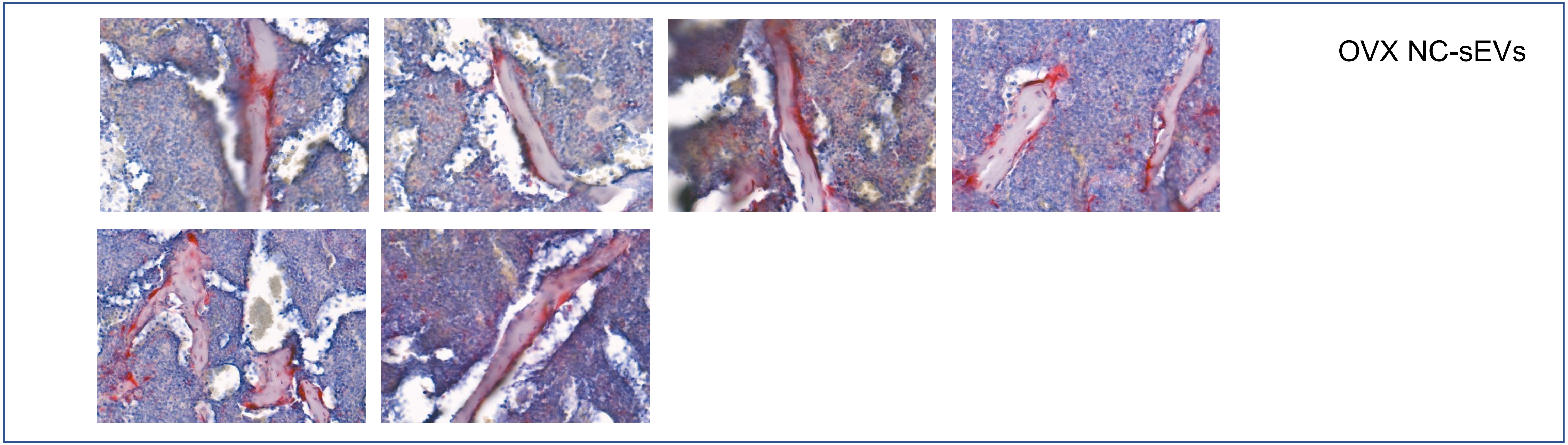

Figure.4k TRAP staining on paraffin-embedded femur sections in each group after corresponding sEVs treatment

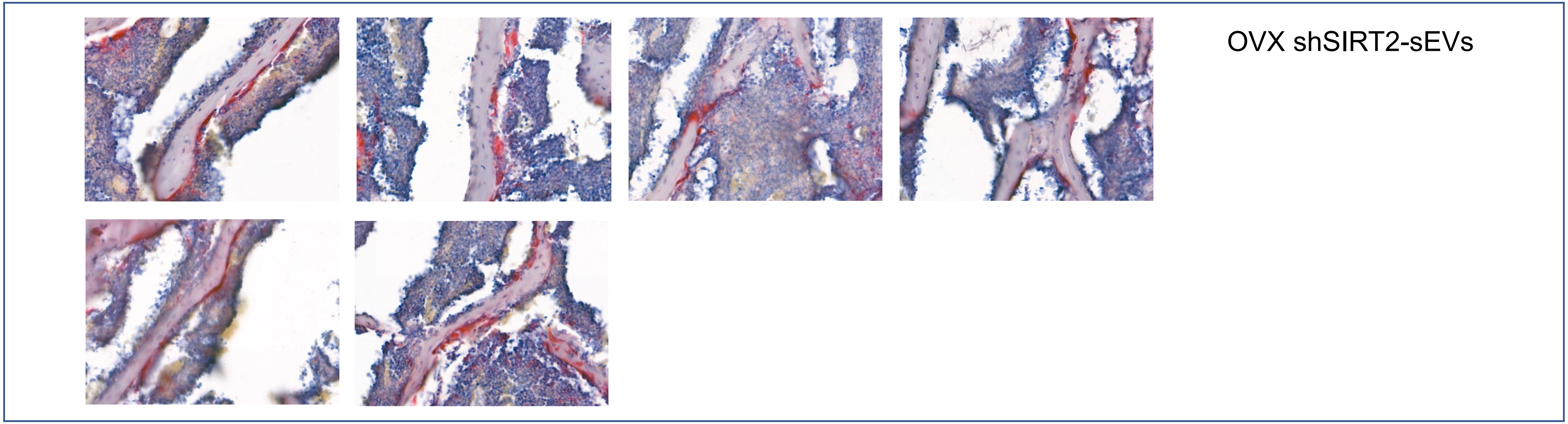

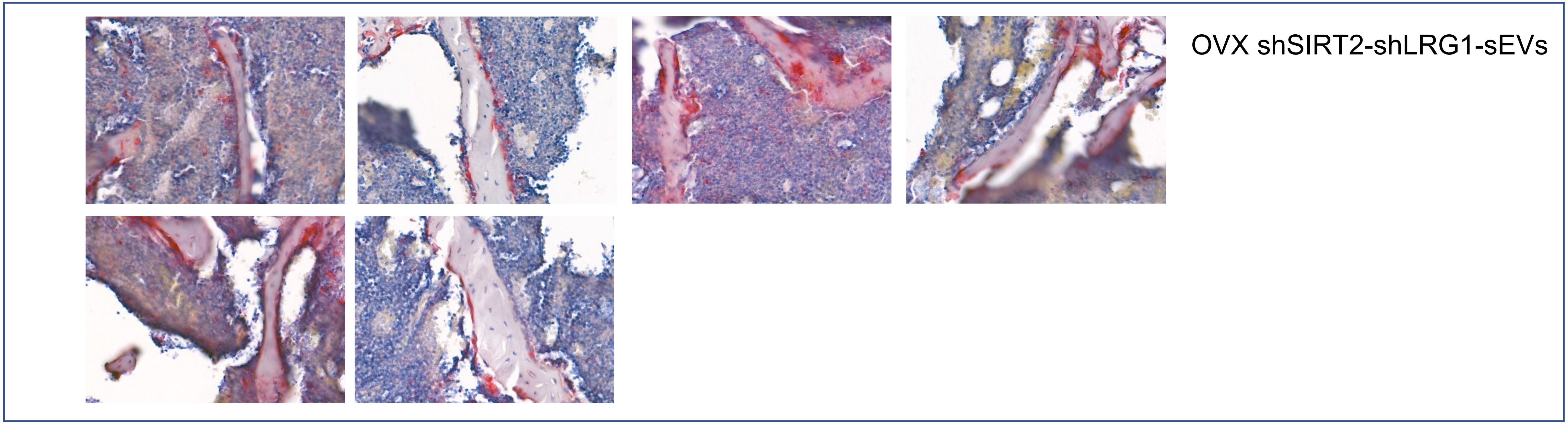

Figure.4k TRAP staining on paraffin-embedded femur sections in each group after corresponding sEVs treatment

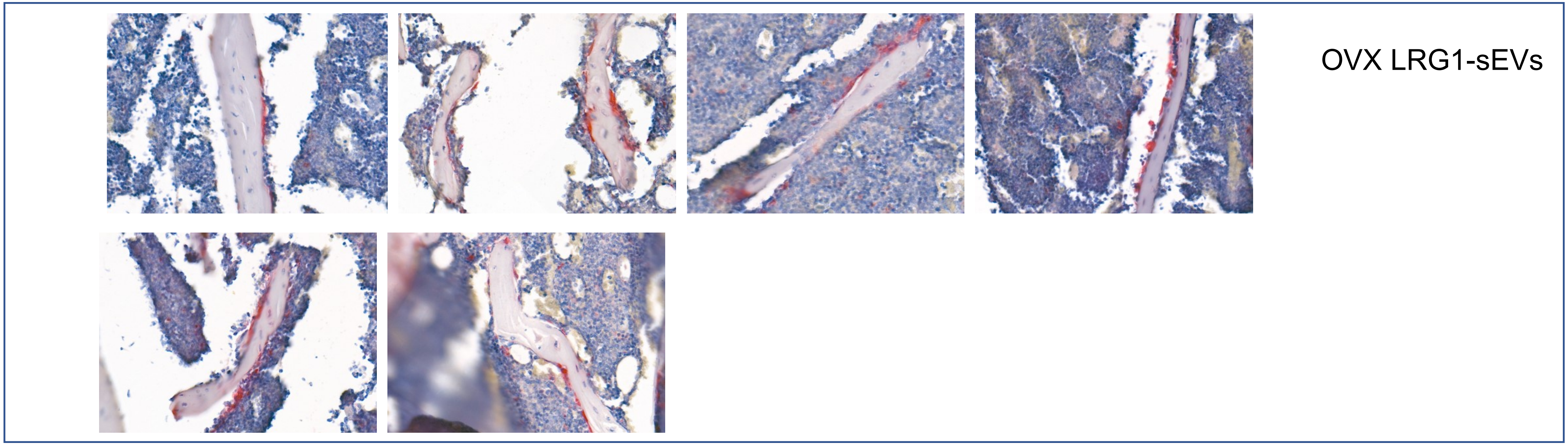

Figure. 5b

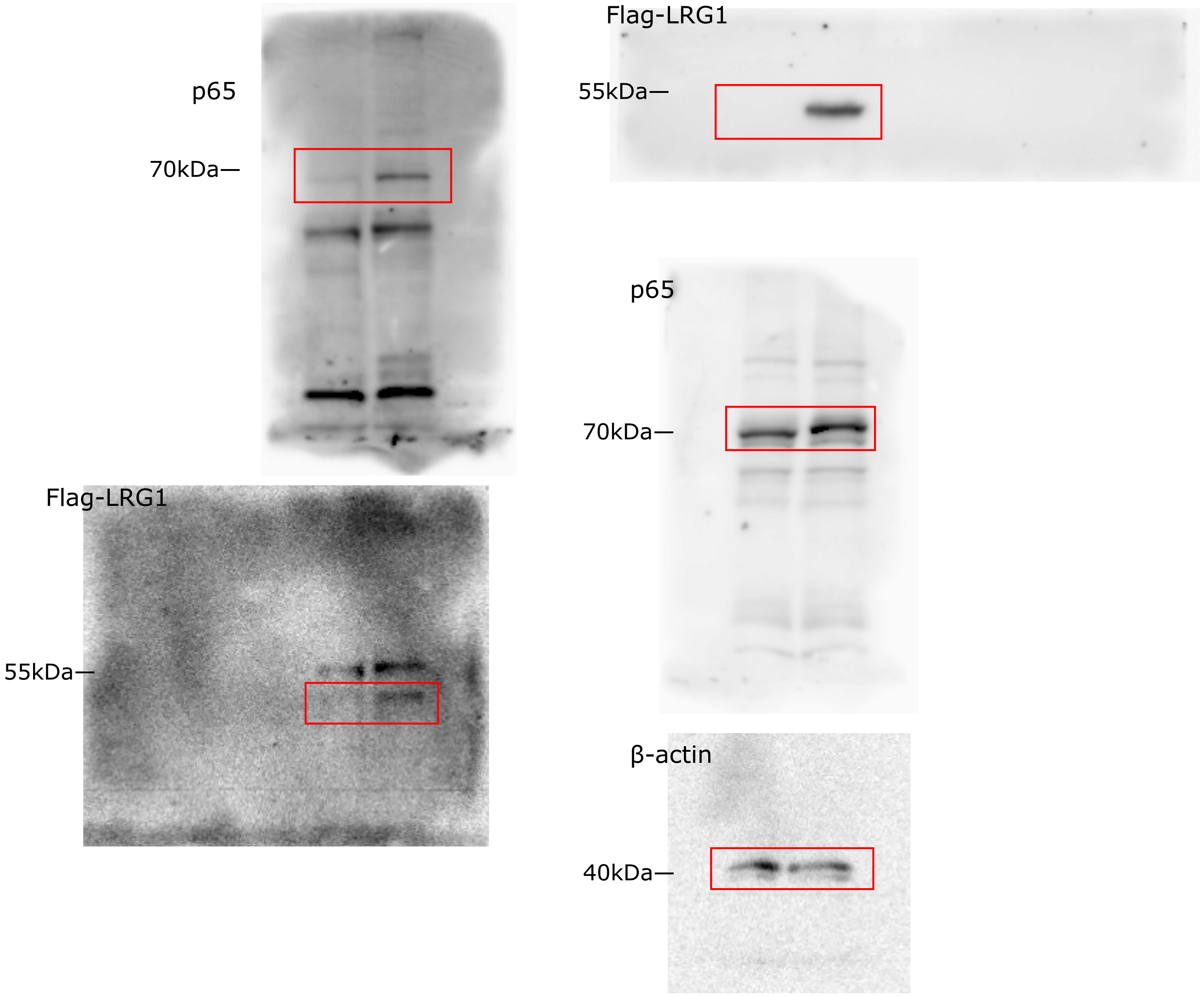
p65
70kDa—
Flag-LRG1
55kDa—
Flag-LRG1
55kDa—
p65
70kDa—
β-actin
40kDa—

Figure. 5c

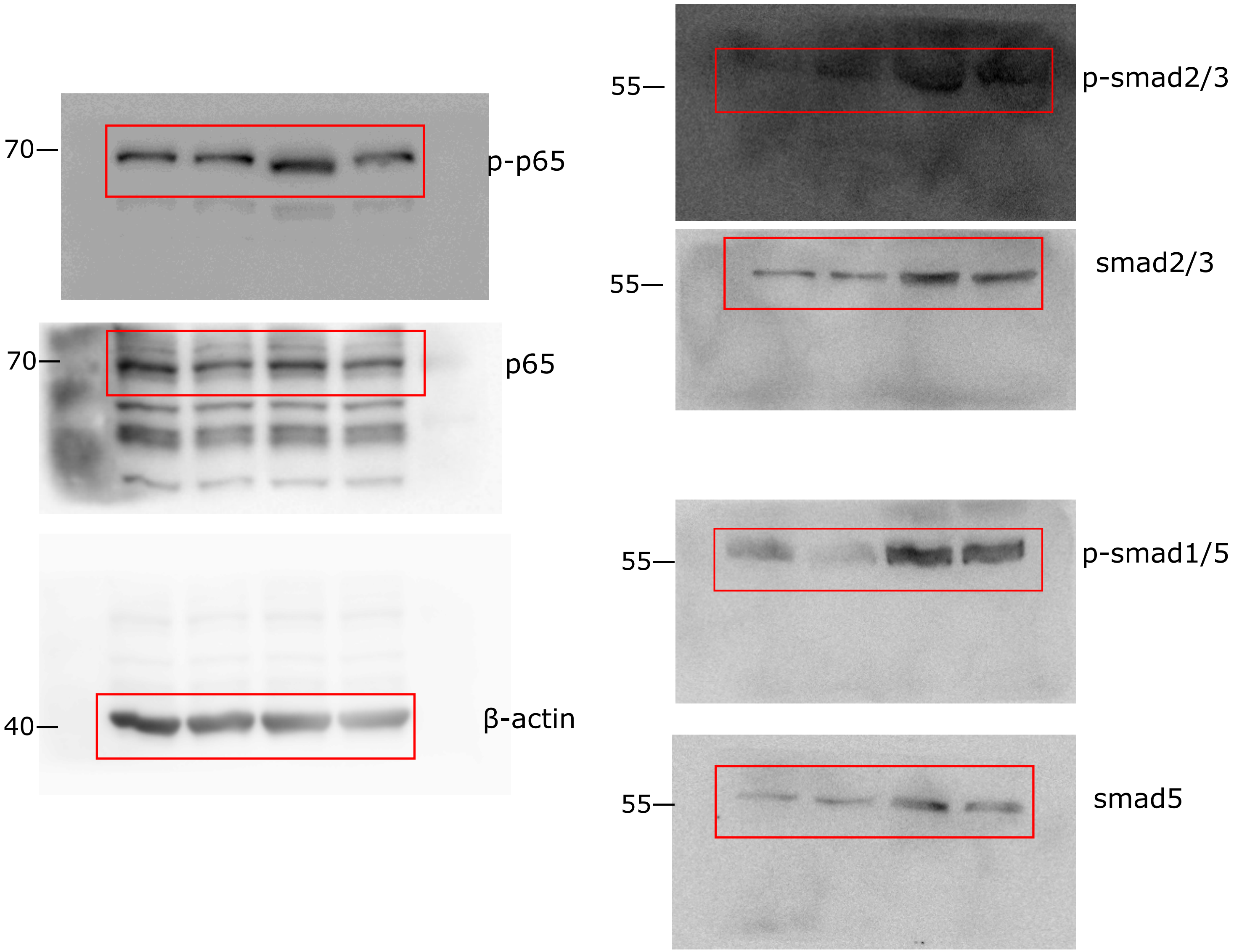
70—
p-p65
70—
p65
40—
β-actin
55—
p-smad2/3
55—
smad2/3
55—
p-smad1/5
55—
smad5

Figure. 5f

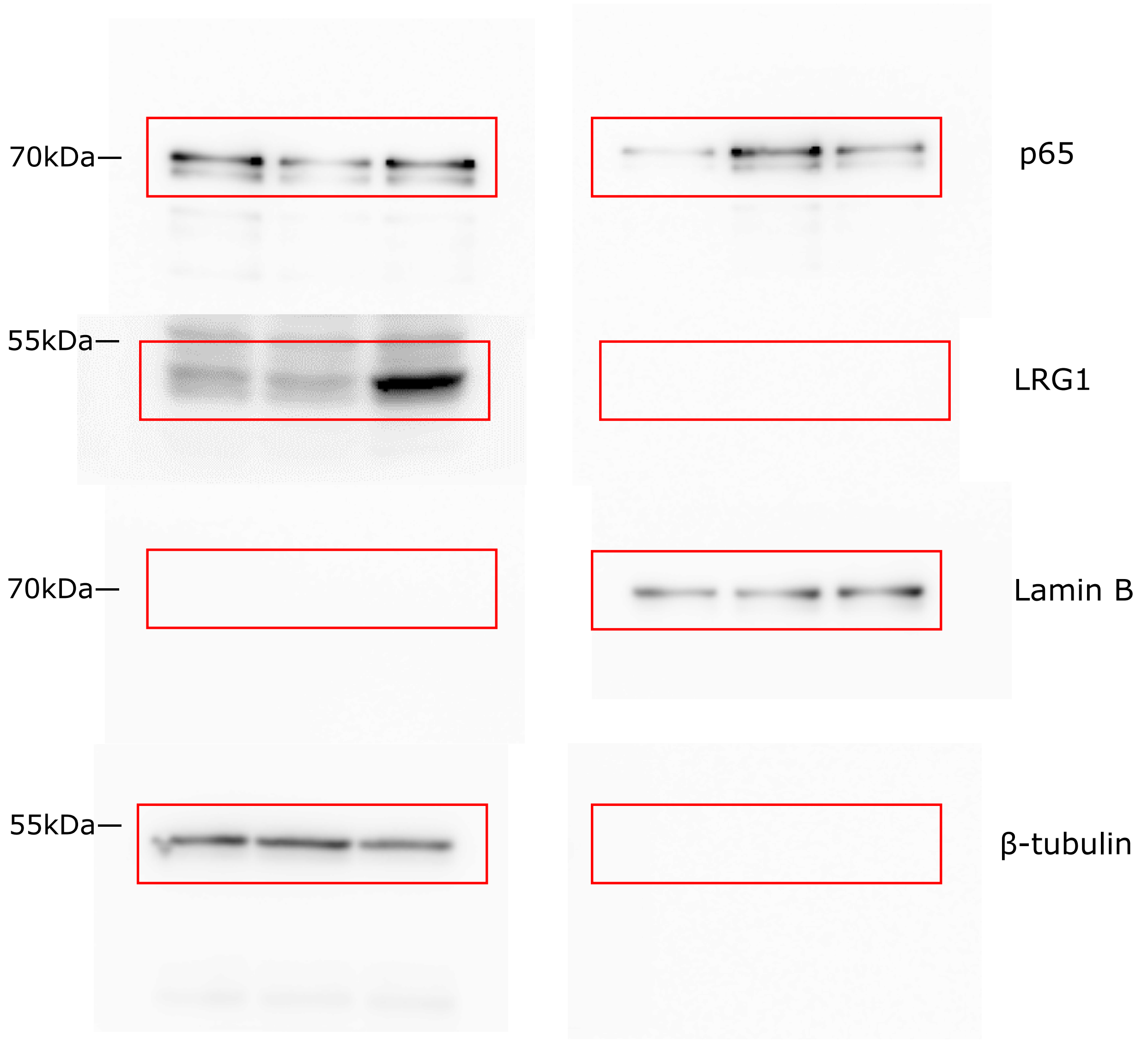

Figure.5d Immunofluorescence analysis of p65 (red) location in RANKL-induced BMDMs treated with LRG1-GFP-sEVs

CTRL

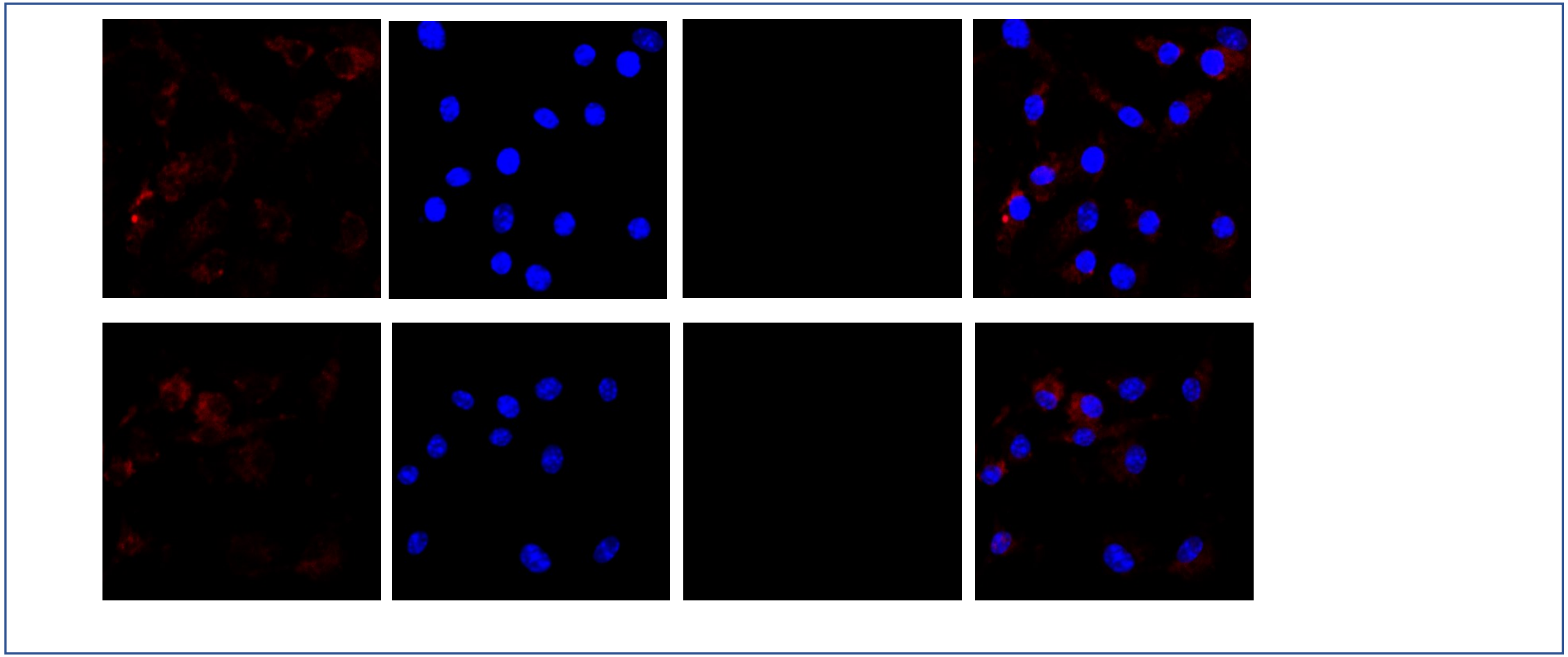

Figure.5d

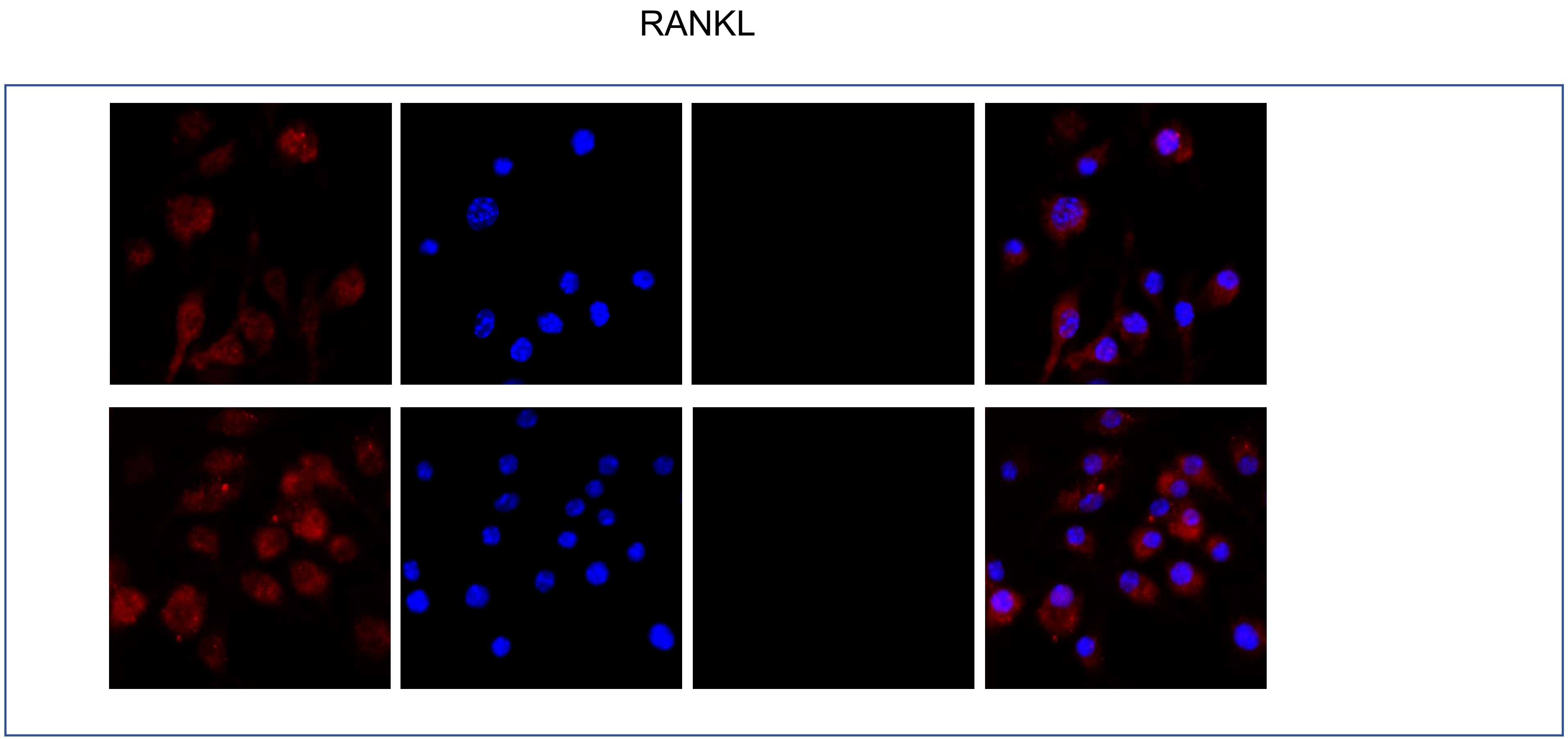

Figure.5d

RANKL+GPF-LRG1-sEVs

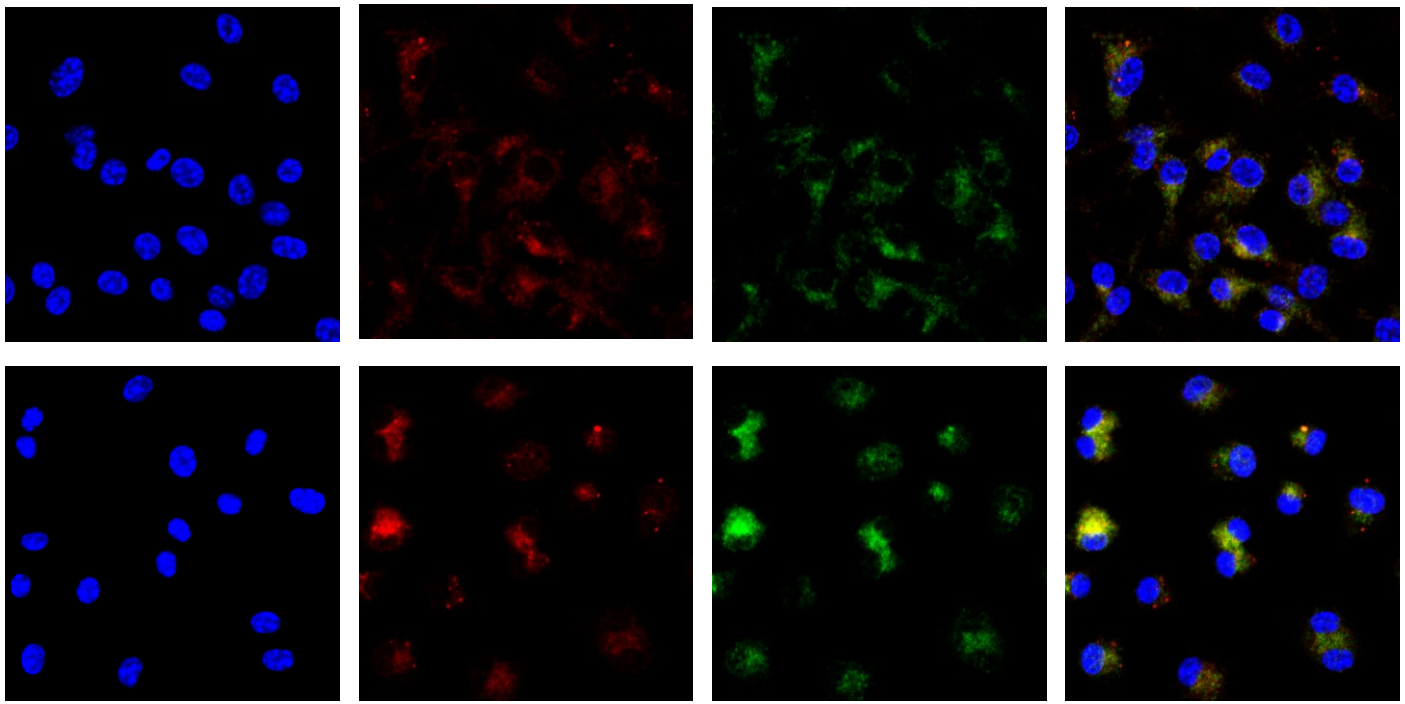

Figure.5g immunofluorescence images of primary BMDMs isolated from aged mice

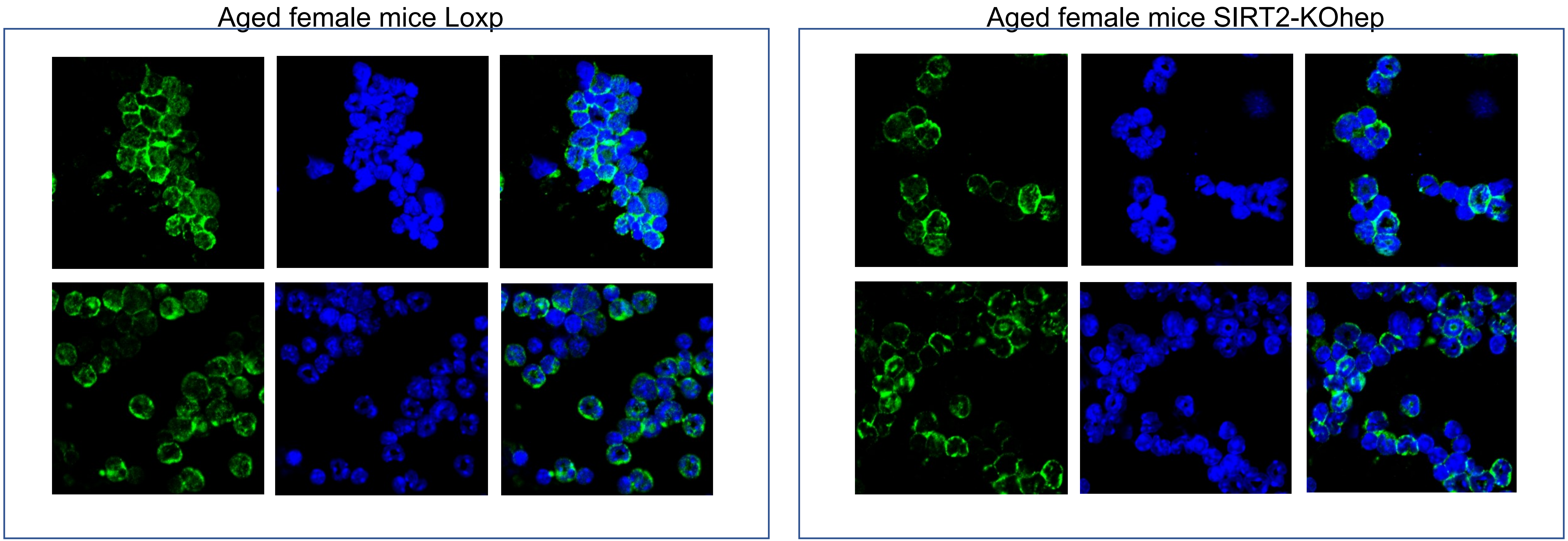

Figure.5i immunofluorescence images of primary BMDMs isolated from aged mice

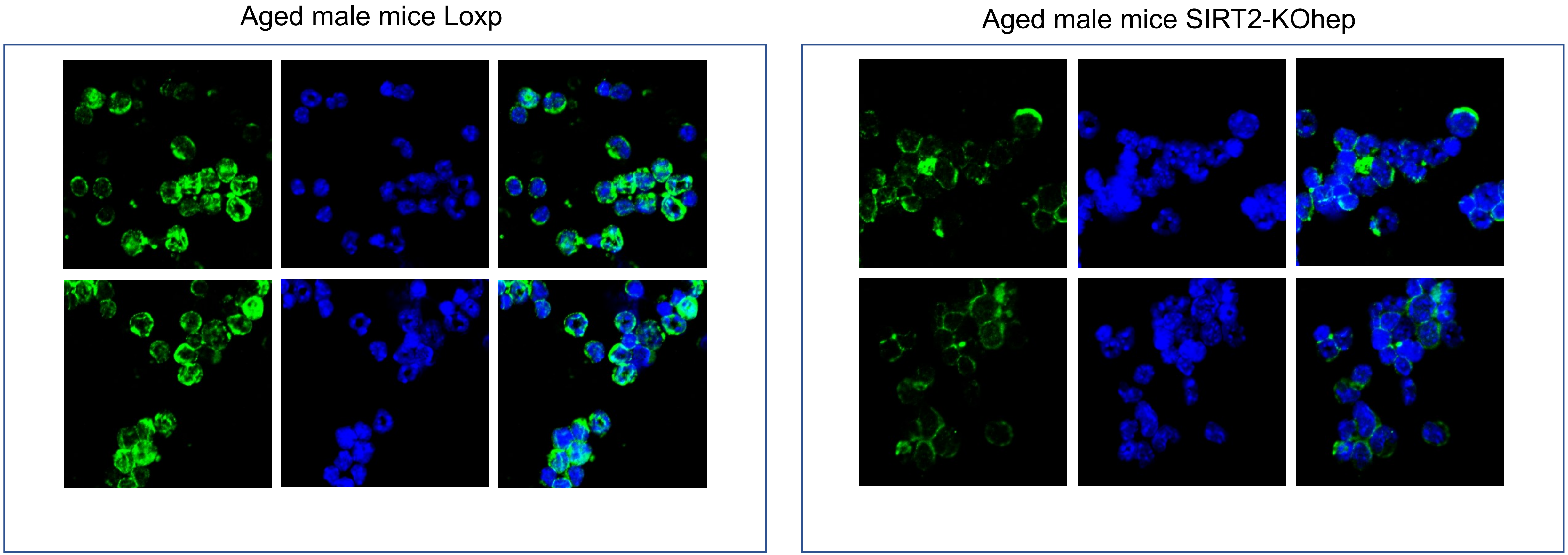

Figure.5k TRAP staining of osteoclasts treated with LRG1-sEVs and the inhibitors of p65 nuclear translocation

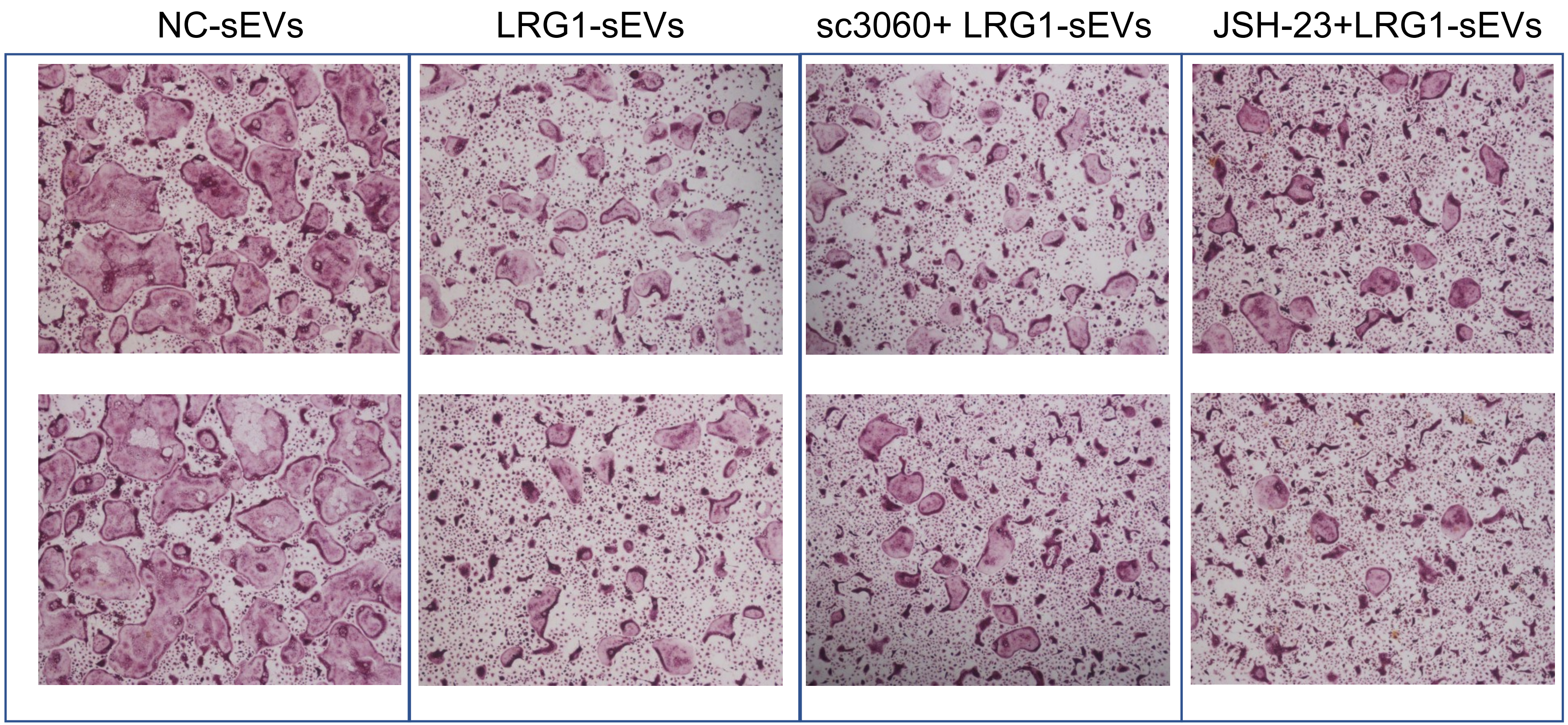

Figure.5n TRAP staining of RAW264.7 cells overexpressed p65 and treated with LRG1-sEVs

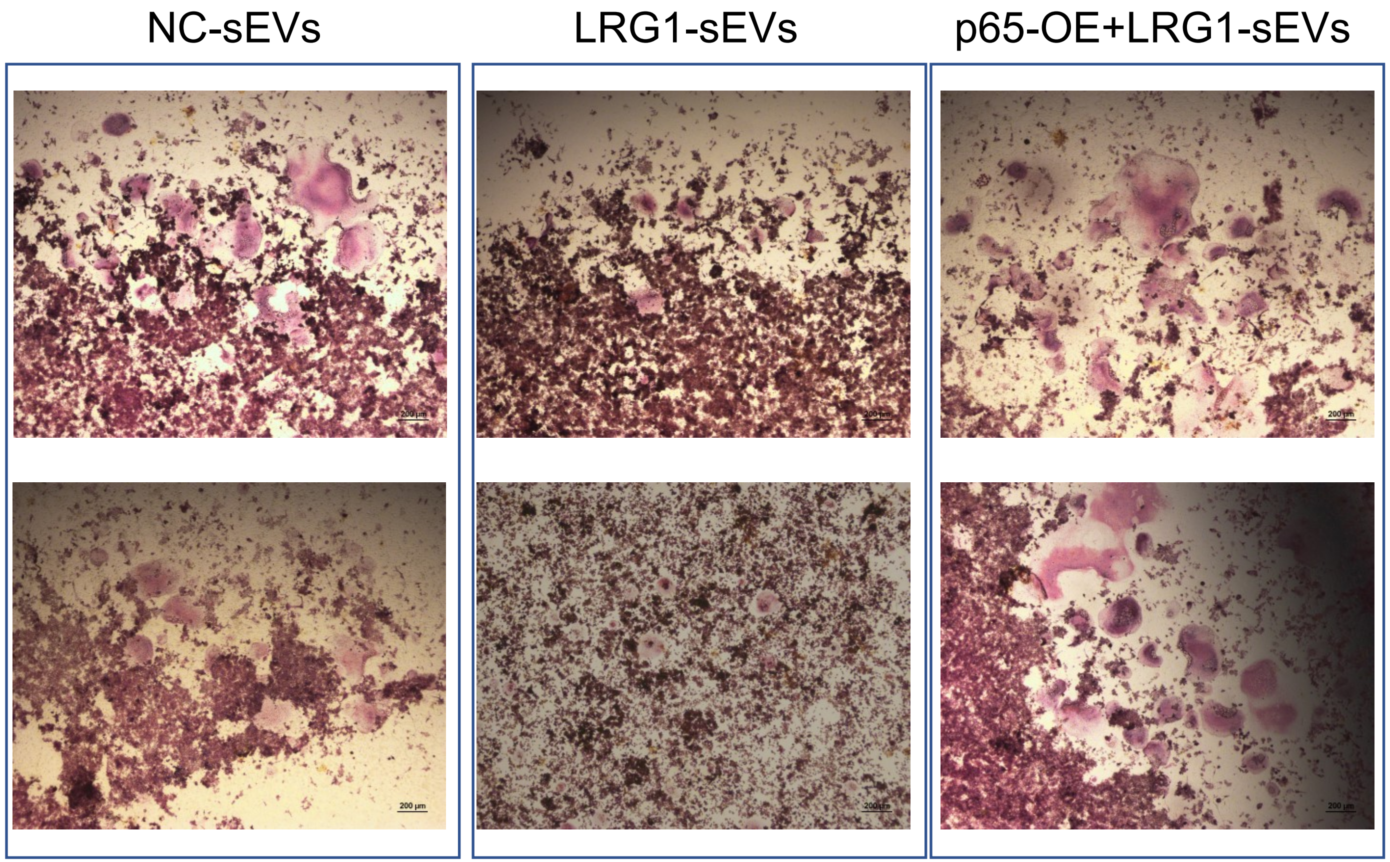

Figure.6b

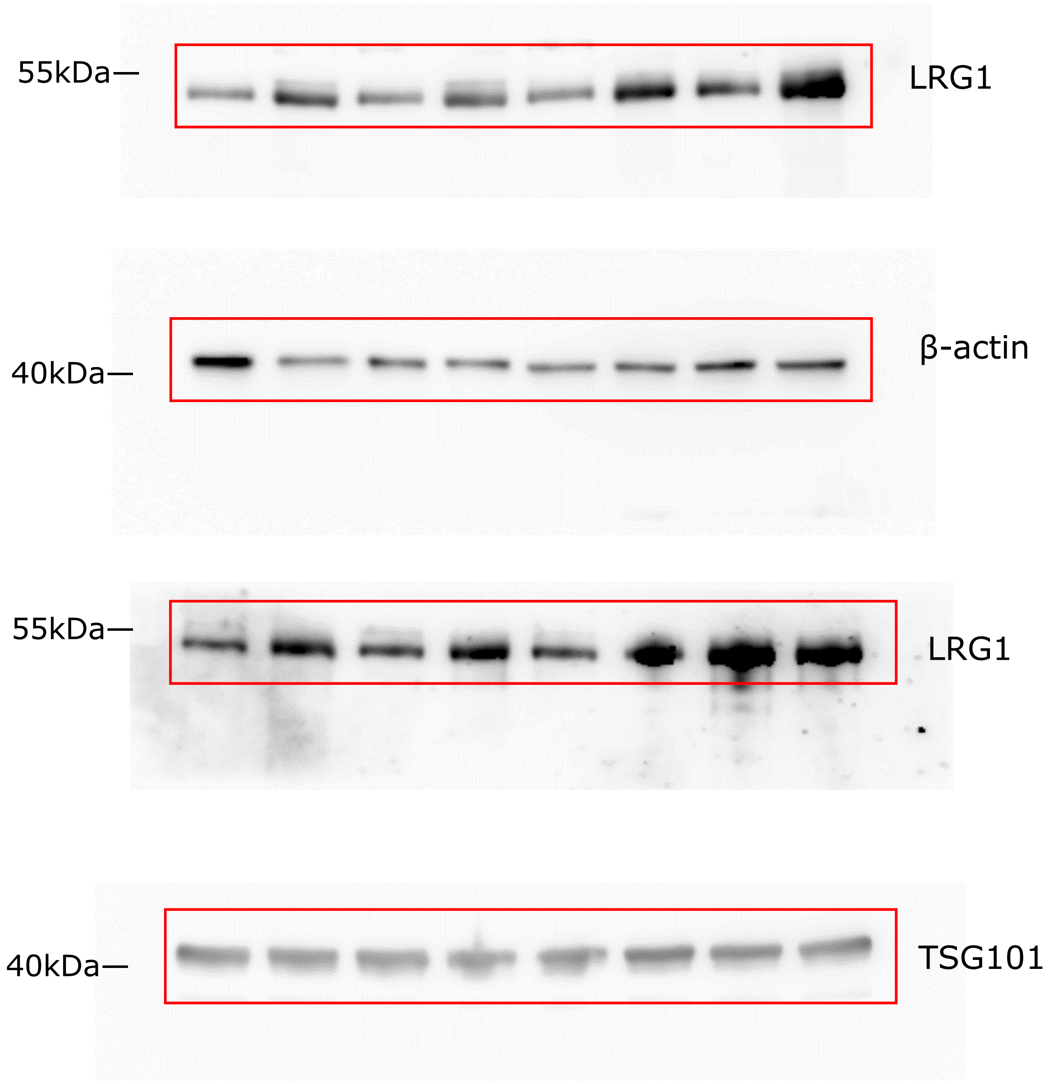

Figure.6c

sham

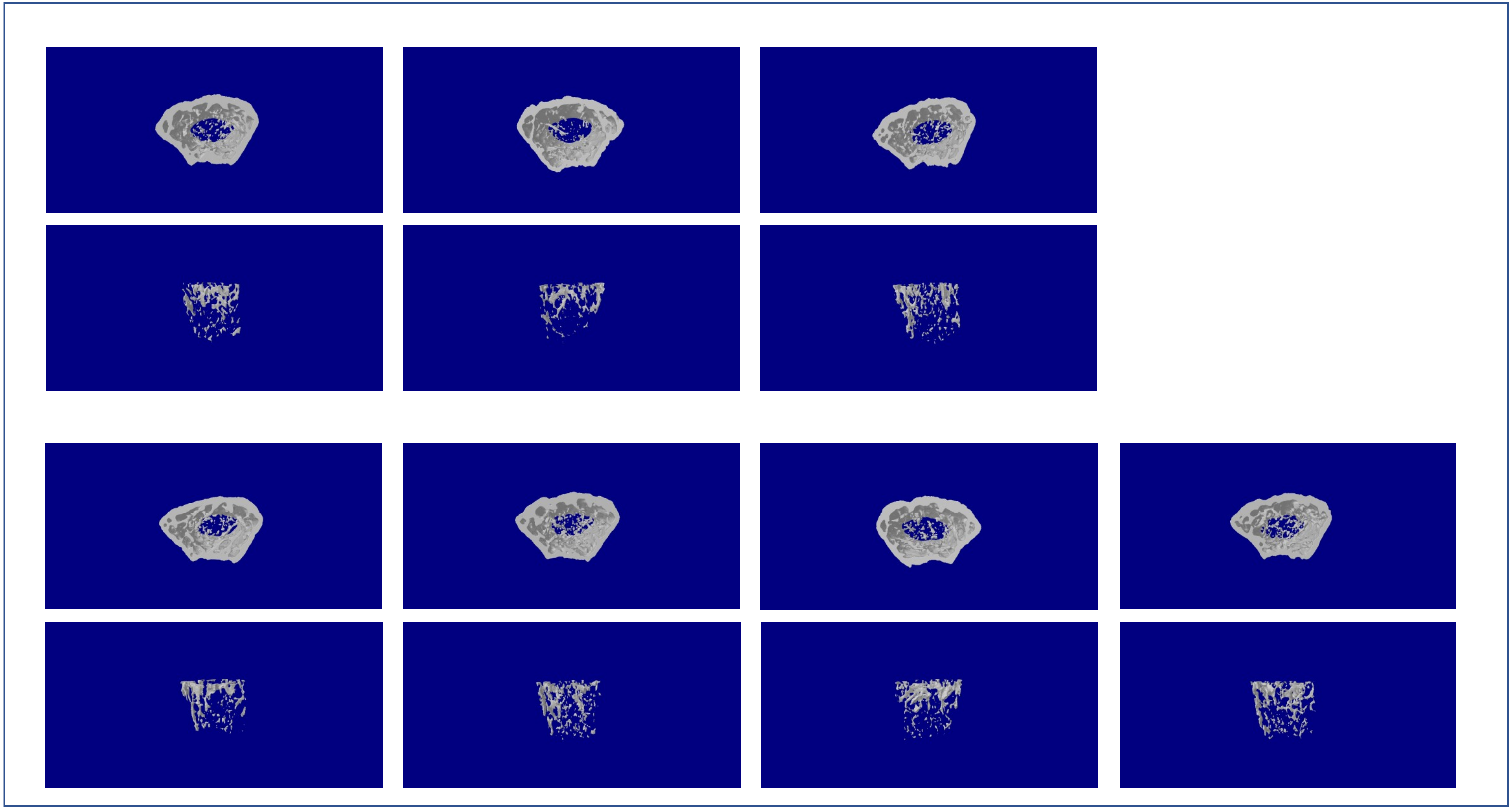

Figure.6c

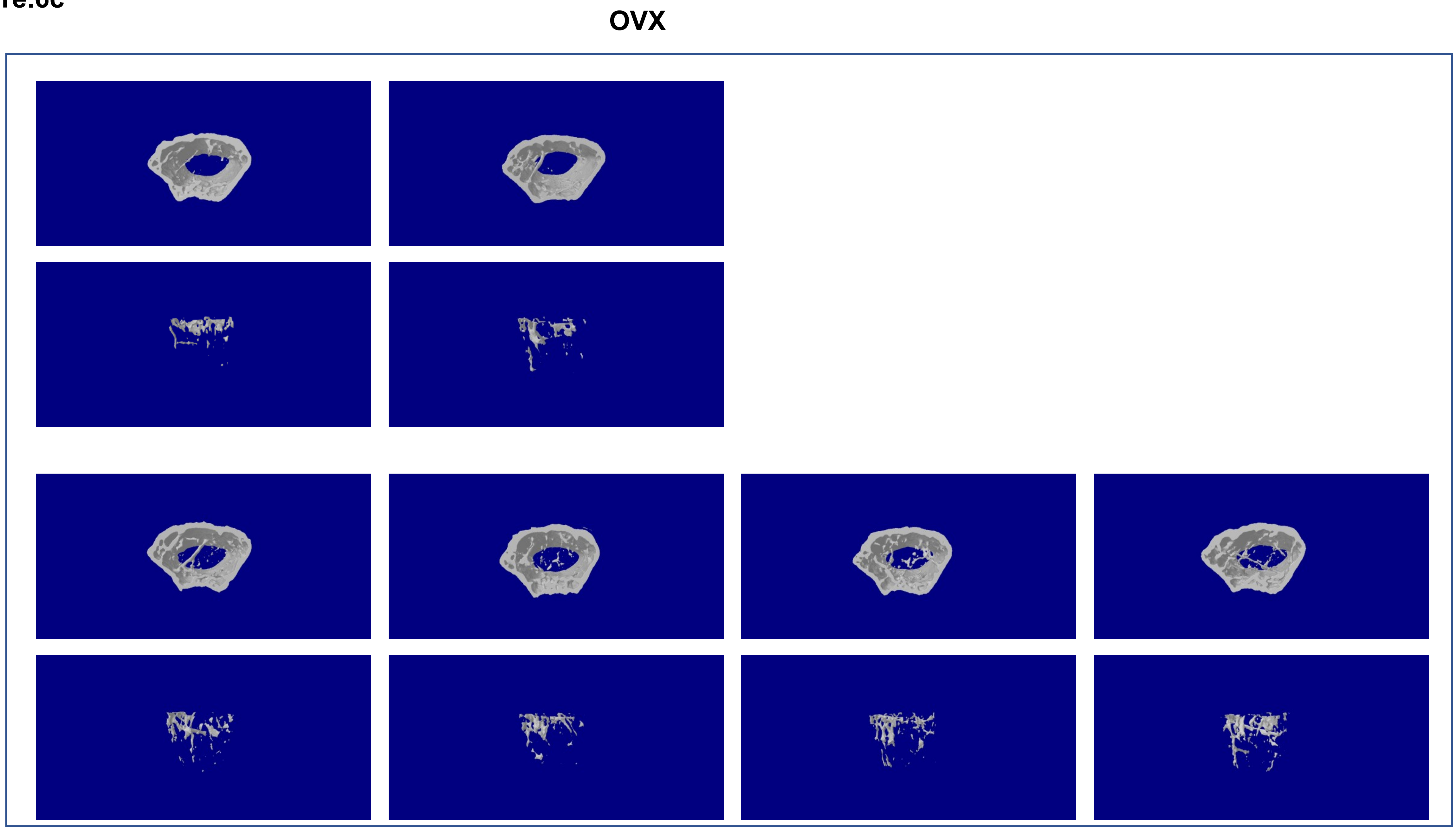

Figure.6c

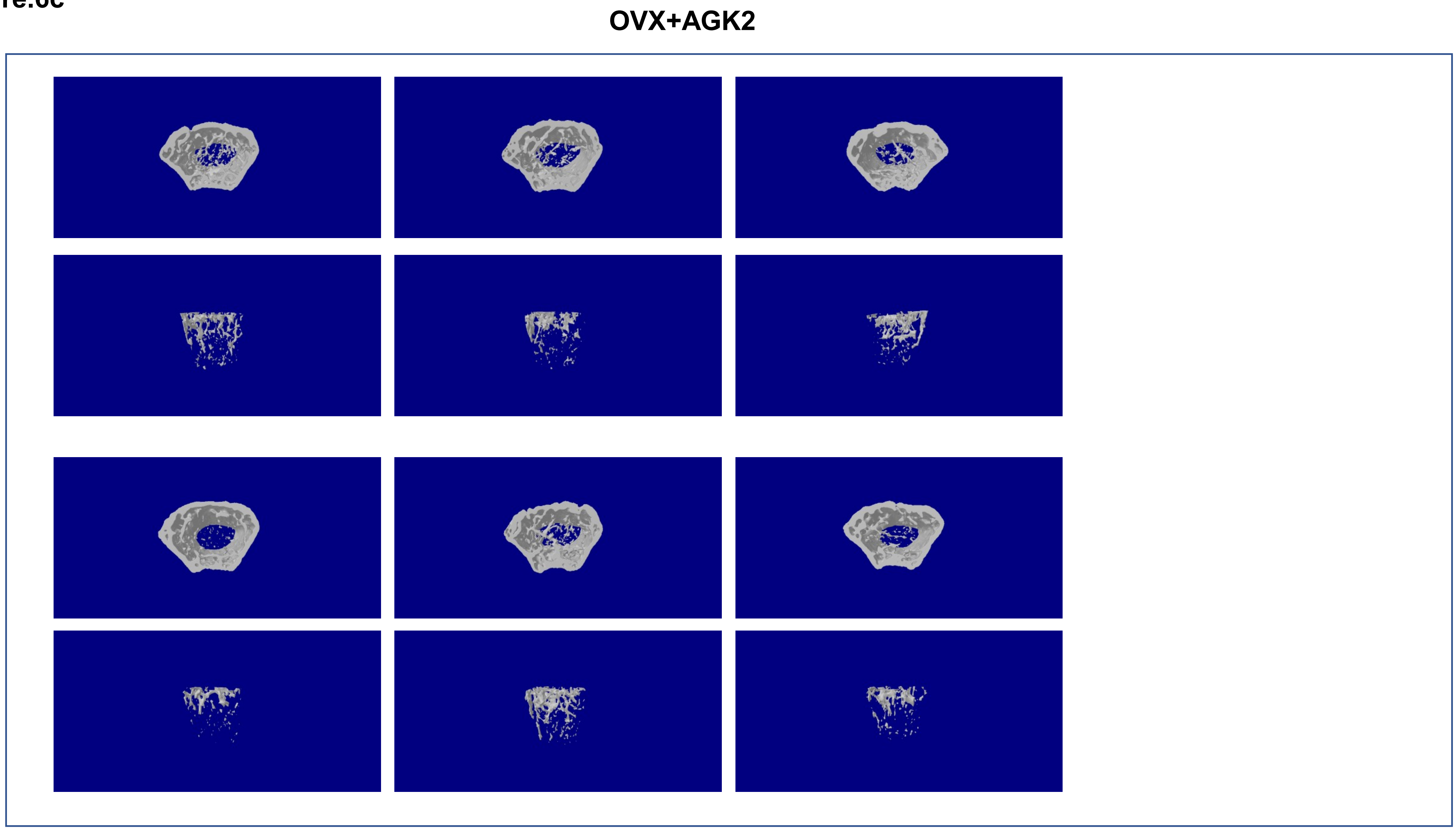

Figure.6e

OVX+Loxp

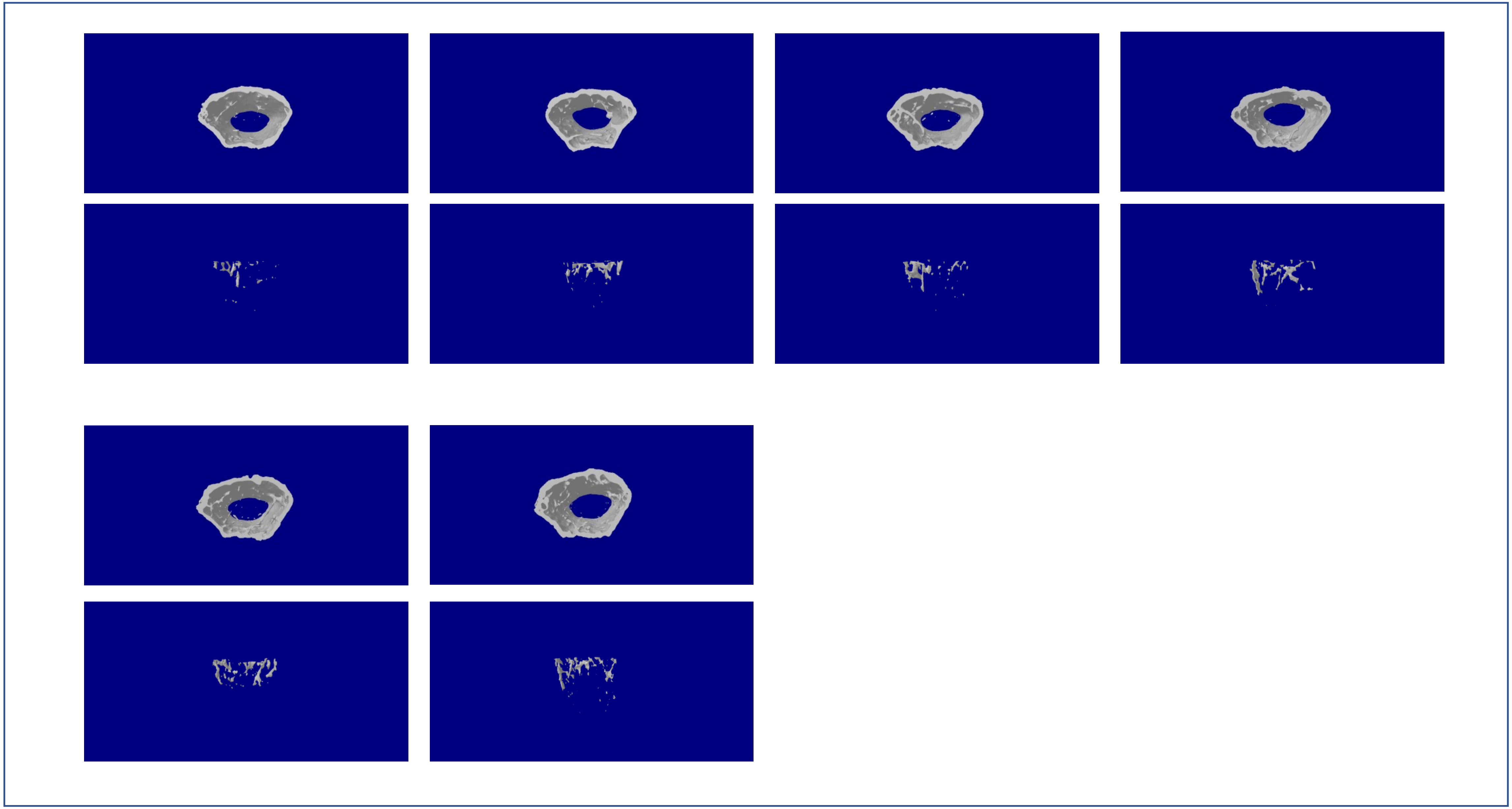

Figure.6e

OVX+SIRT2-KOhep

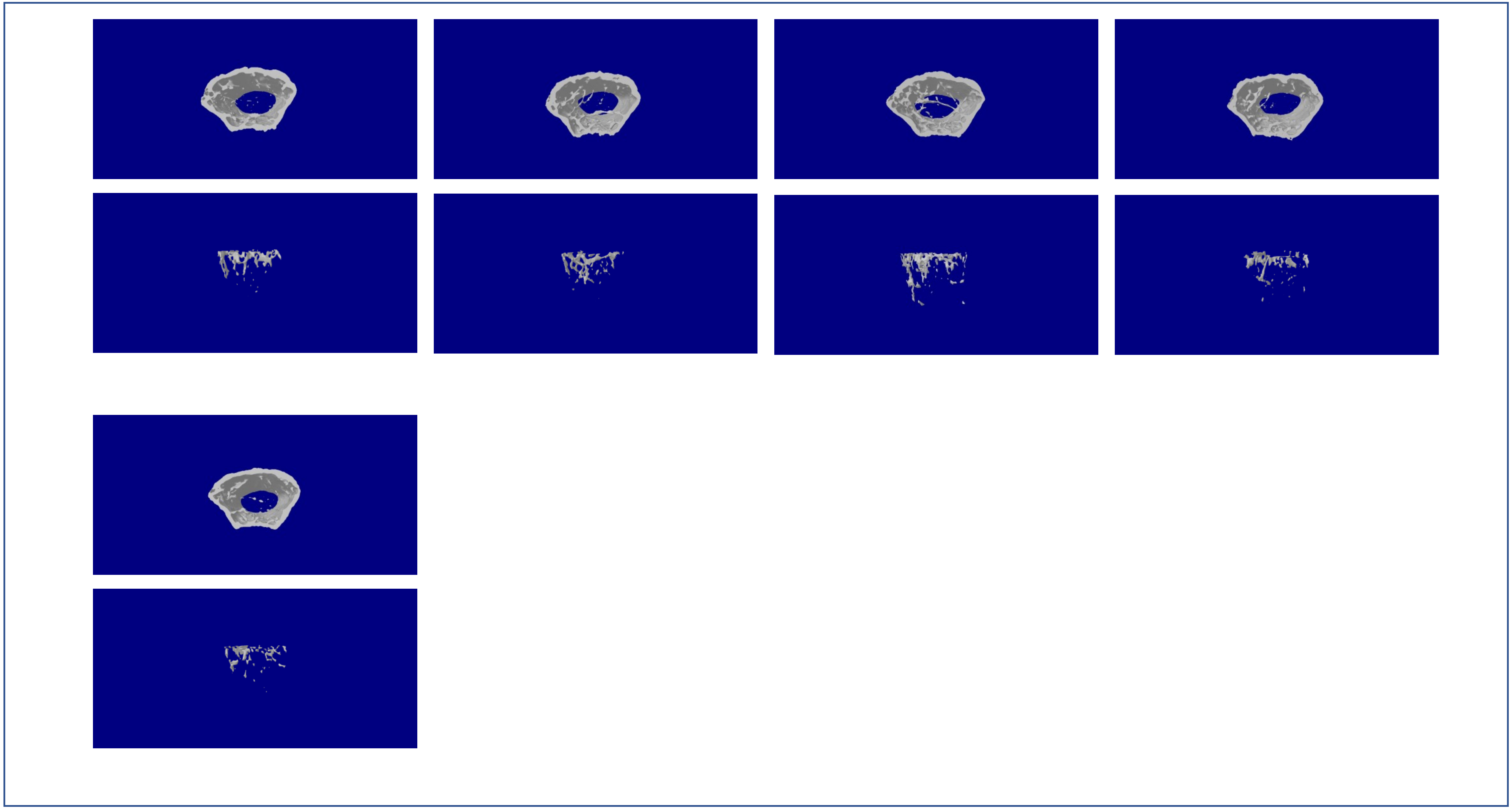

Figure.6e

OVX+SIRT2-Kohep+AGK2

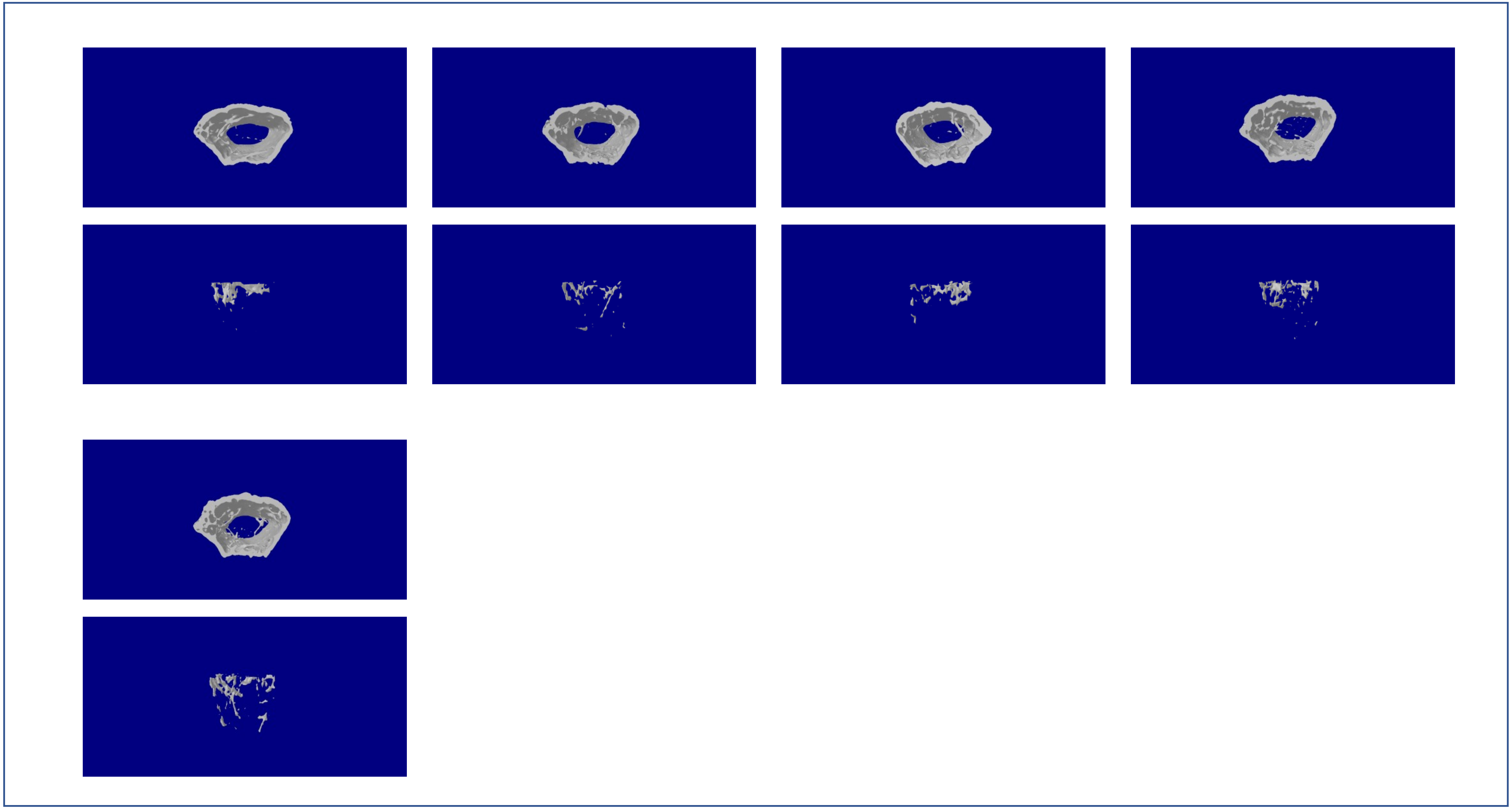

Figure.7b TRAP staining images of human PBMCs cultured with RANKL and sEVs

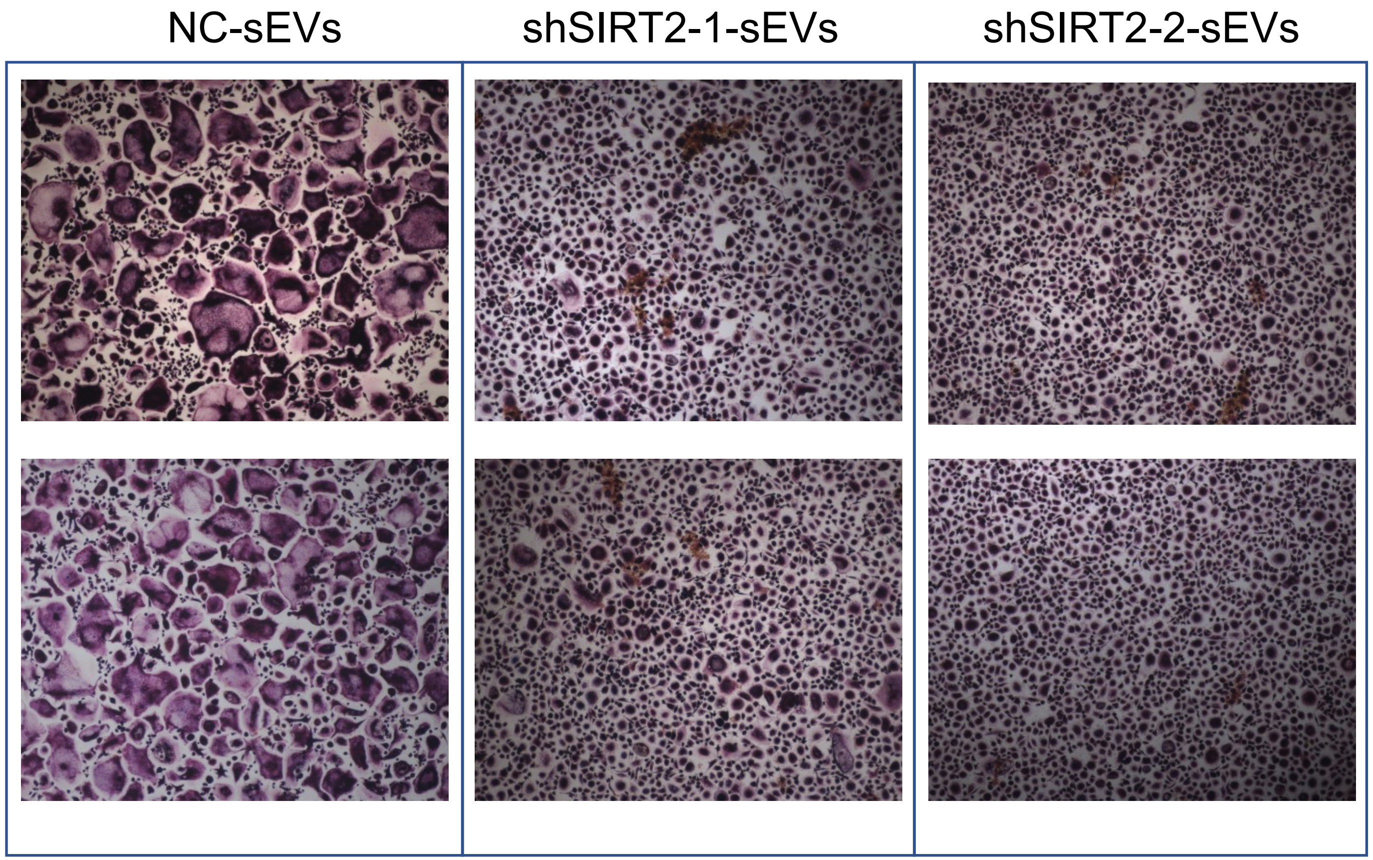

Figure.7f TRAP staining images of human PBMCs cultured with RANKL and sEVs

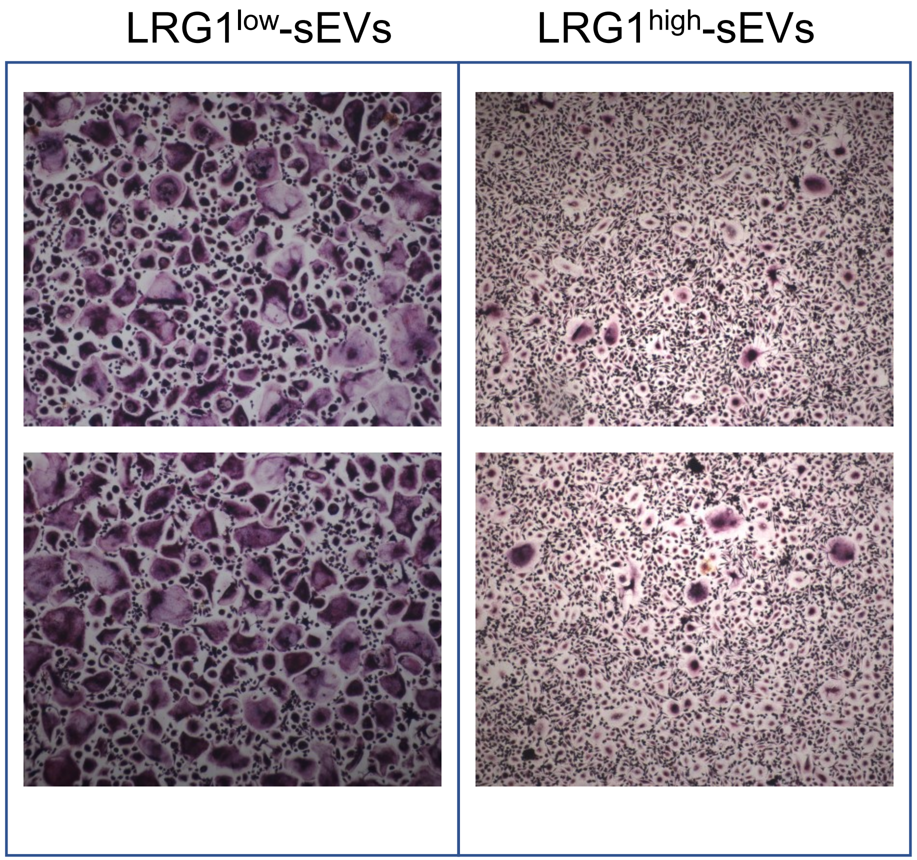

Figure.7j TRAP staining images of PBMCs cultured with RANKL and sEVs-LRG1 or denosumab

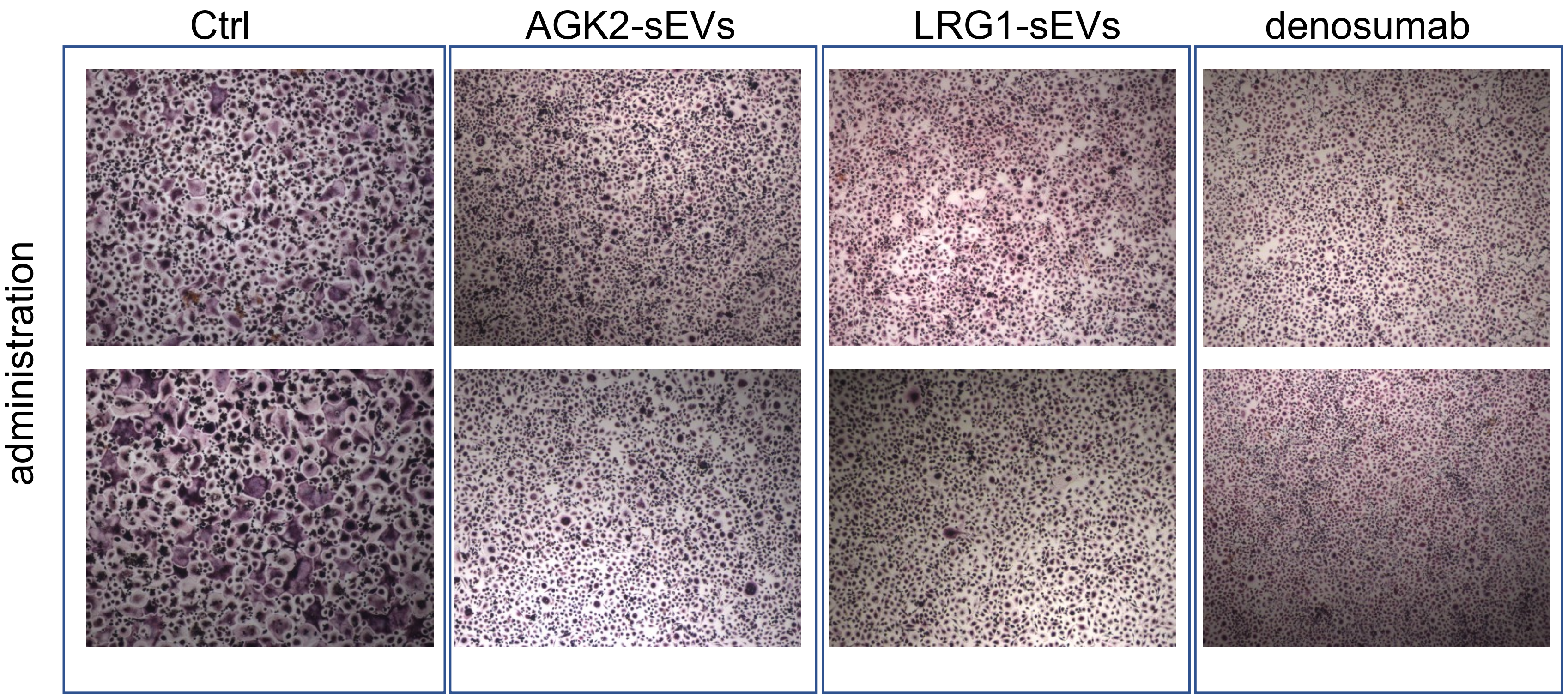

Figure.7j TRAP staining images of PBMCs cultured with RANKL and sEVs-LRG1 or denosumab

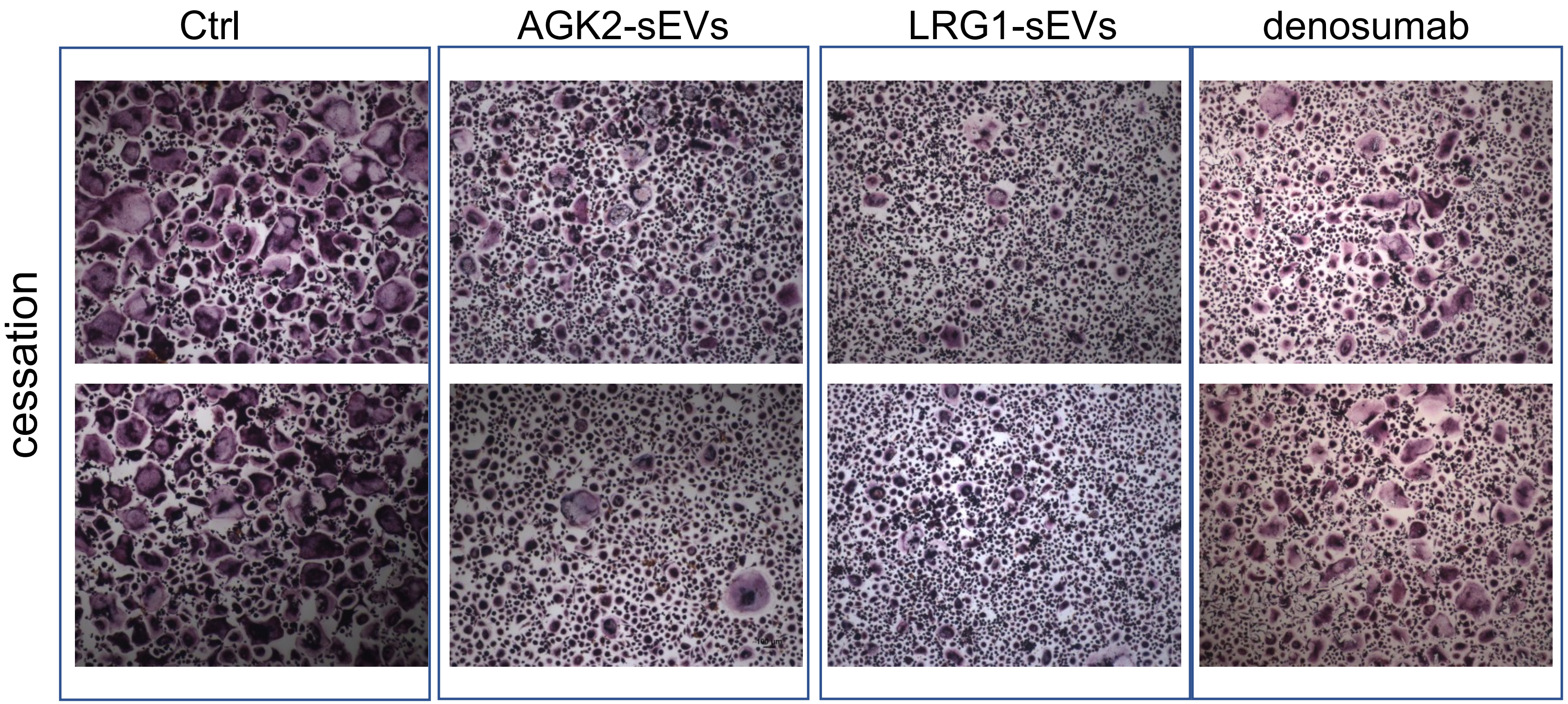

Figure7I IHC images of SIRT2 expression levels in human liver tissues

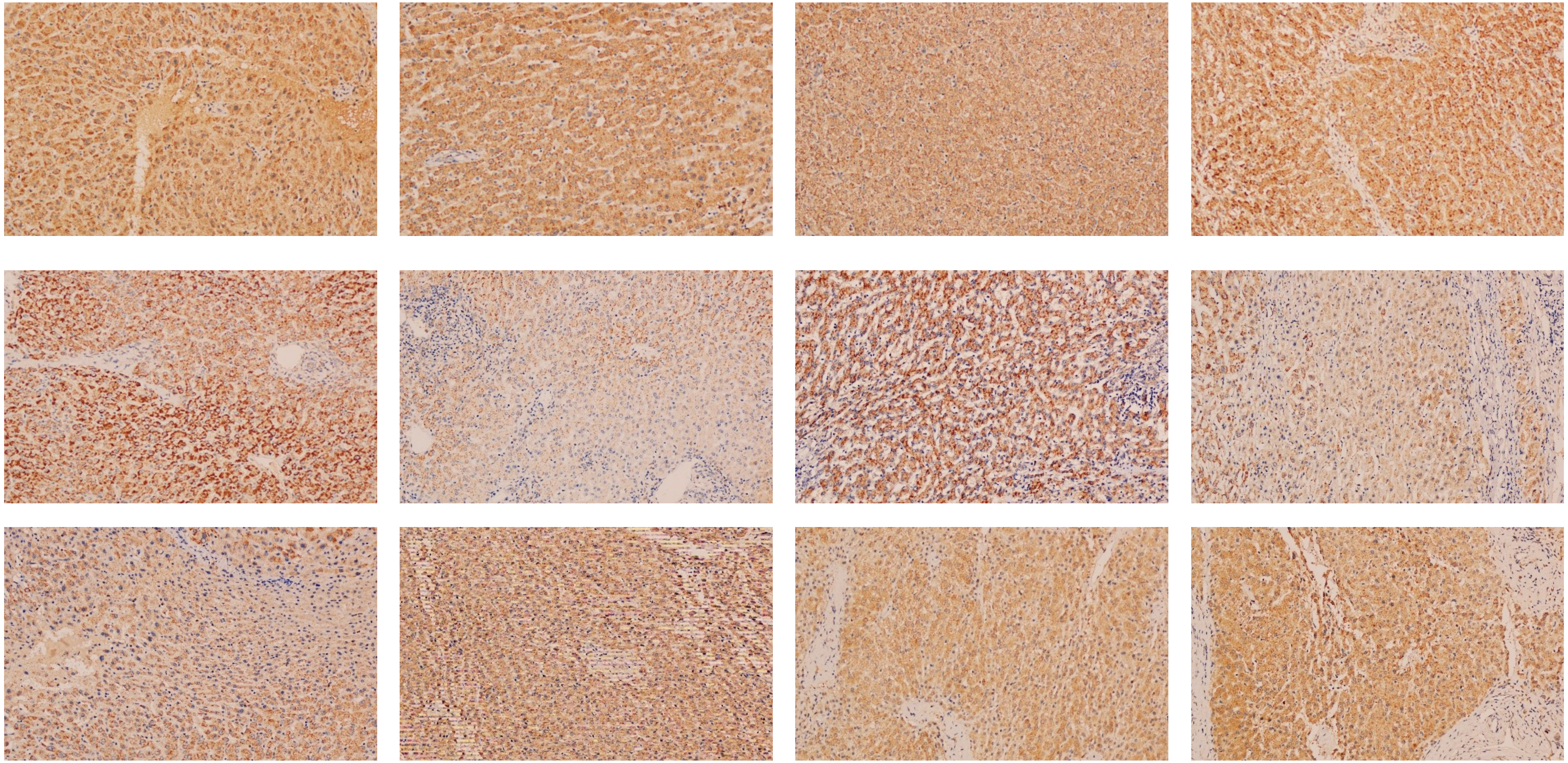

Figure7I IHC images of SIRT2 expression levels in human liver tissues

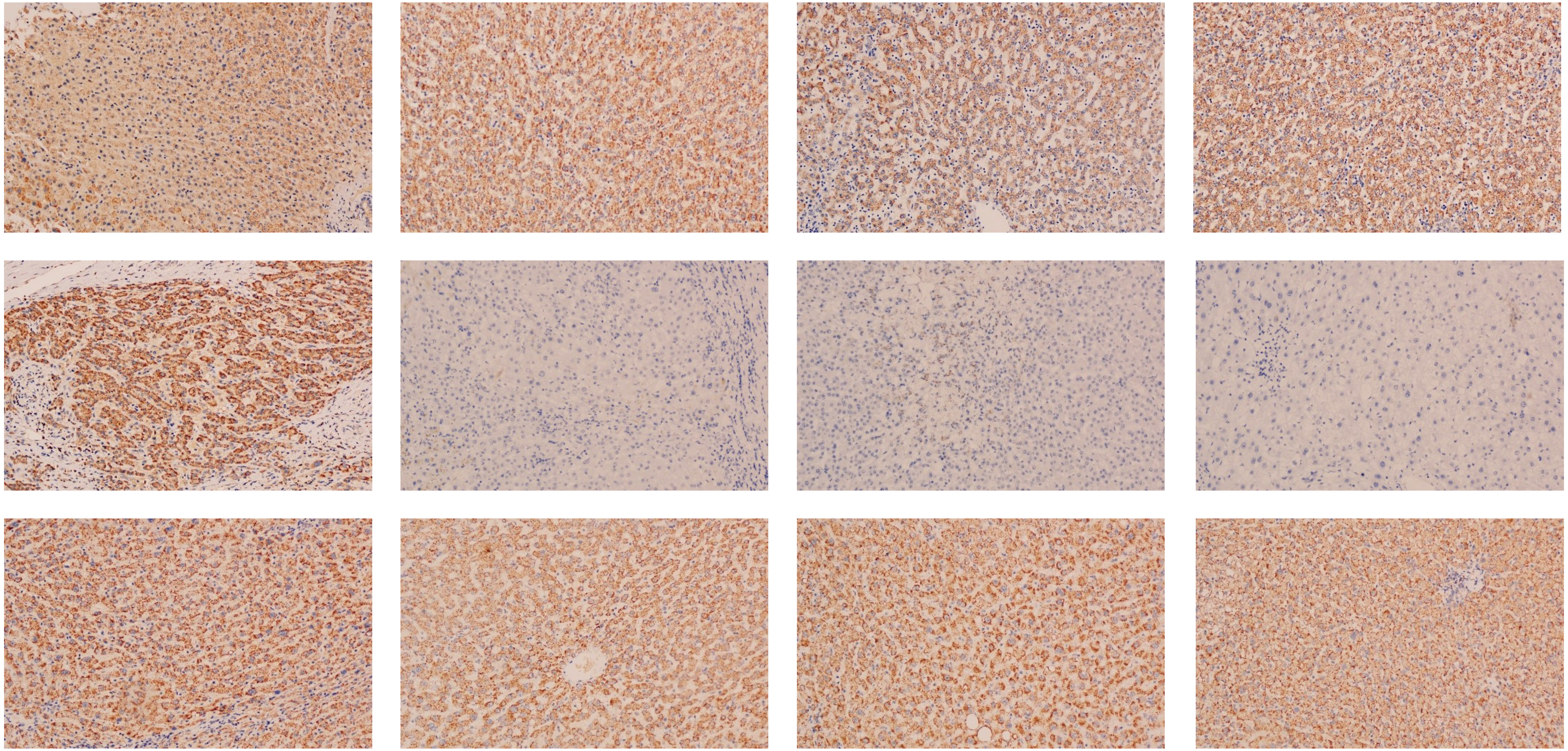

Figure7I IHC images of SIRT2 expression levels in human liver tissues

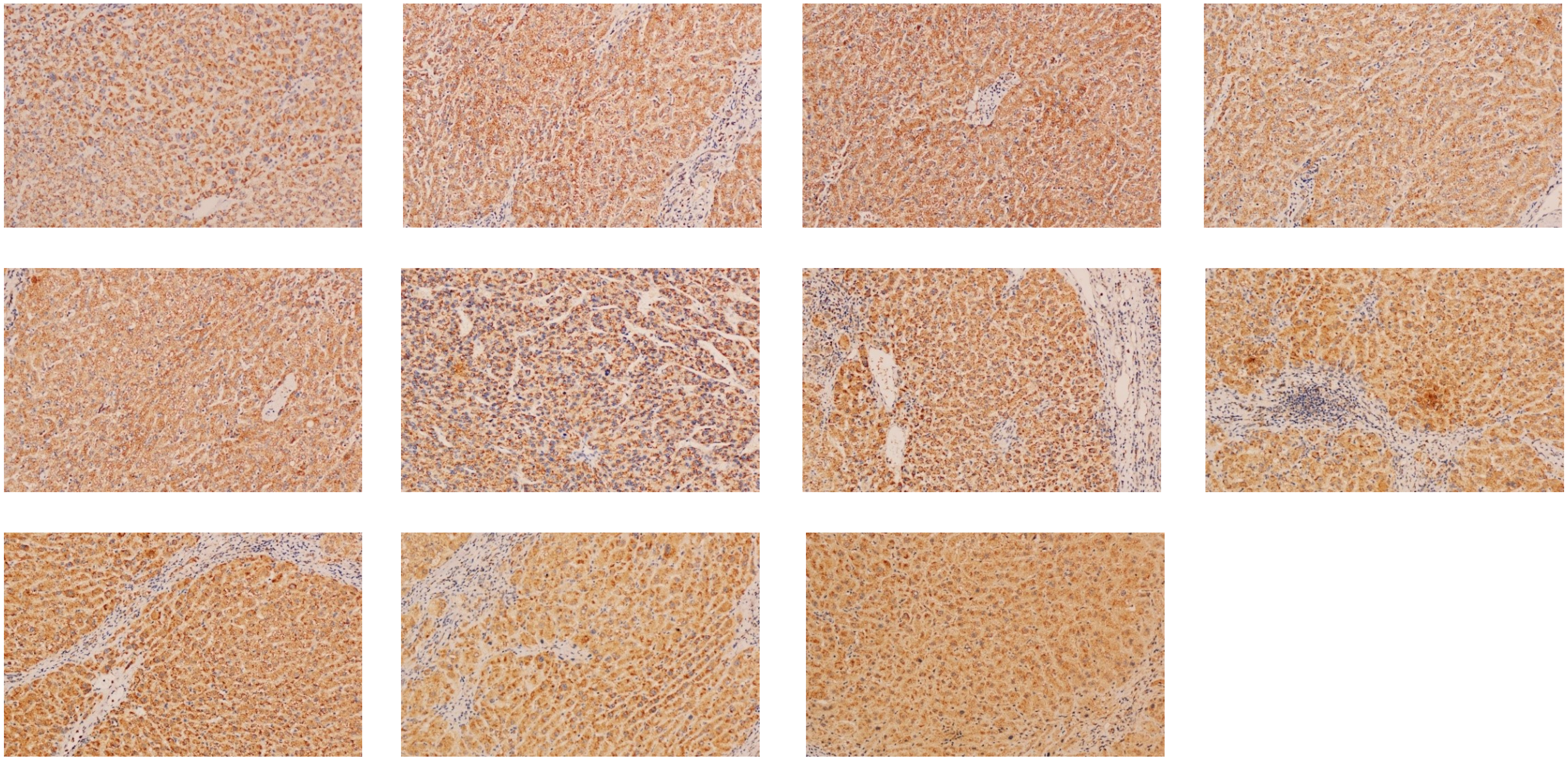

Figure.7n western blot analysis of the protein expression of plasma-sEVs-LRG1 from patients

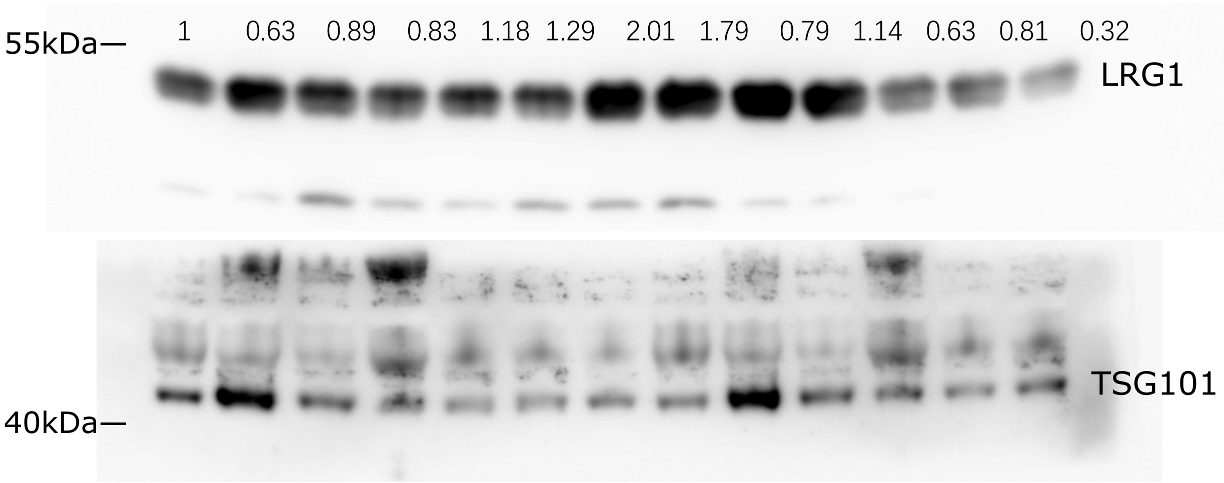

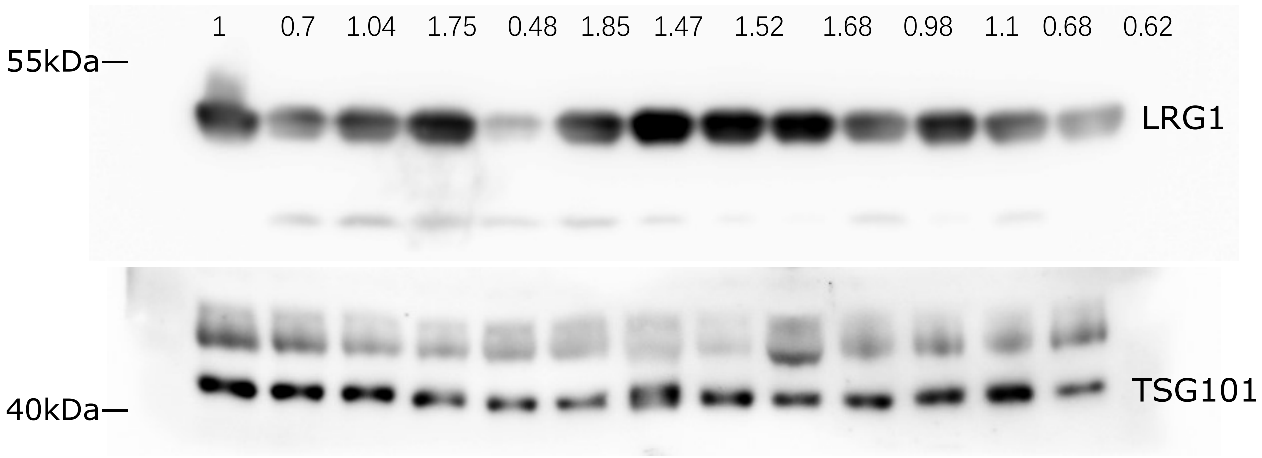
1 0.7 1.04 1.75 0.48 1.85 1.47 1.52 1.68 0.98 1.1 0.68 0.62
55kDa—
LRG1
TSG101
40kDa—

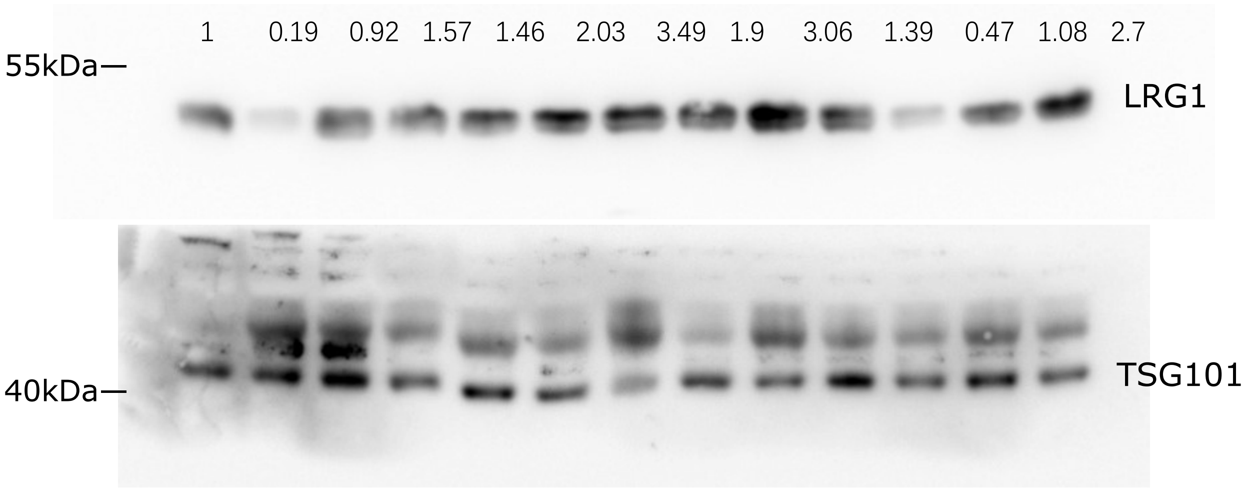
1 0.19 0.92 1.57 1.46 2.03 3.49 1.9 3.06 1.39 0.47 1.08 2.7
55kDa
LRG1
TSG101
40kDa

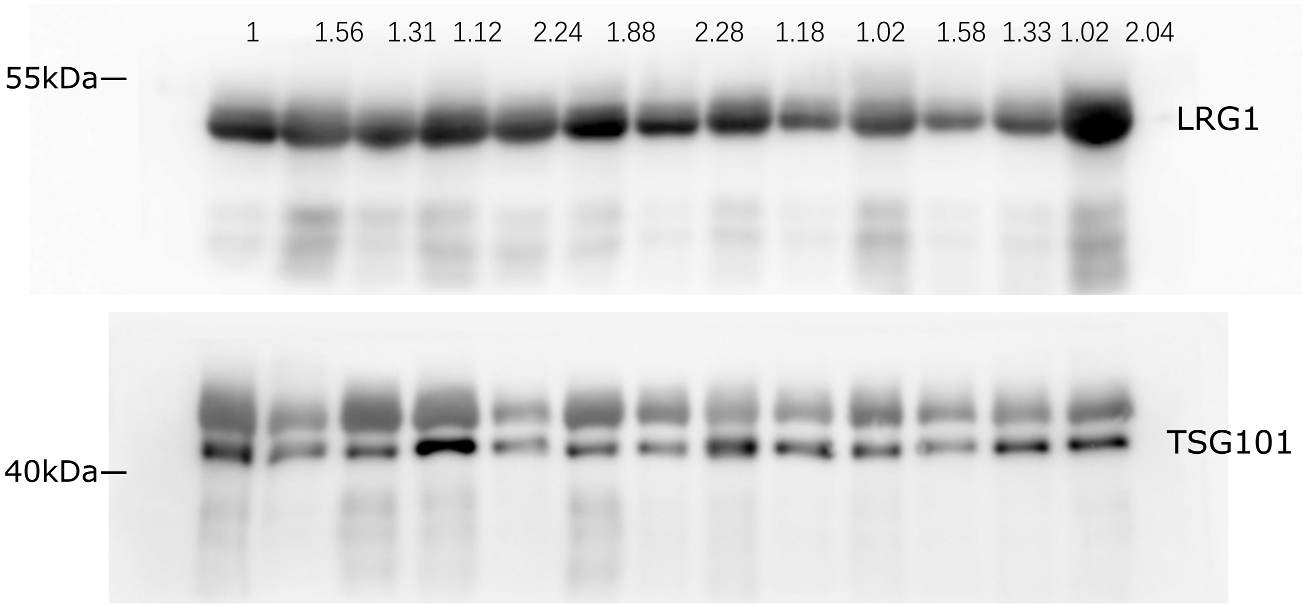
1 1.56 1.31 1.12 2.24 1.88 2.28 1.18 1.02 1.58 1.33 1.02 2.04
55kDa—
LRG1
TSG101
40kDa—

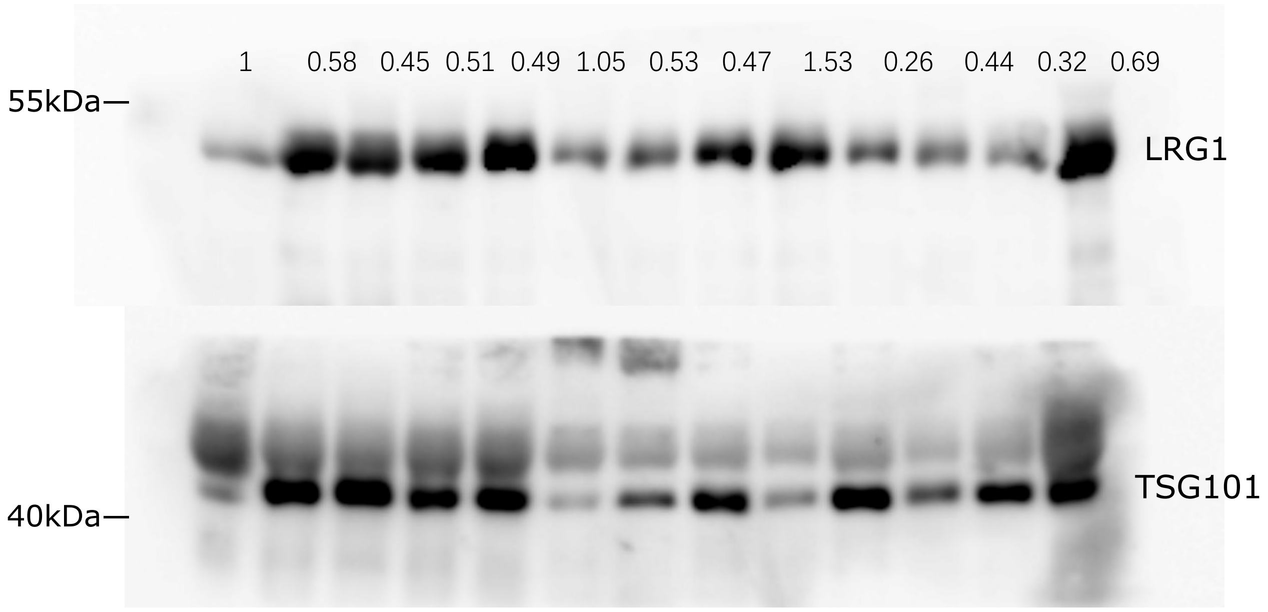
1 0.58 0.45 0.51 0.49 1.05 0.53 0.47 1.53 0.26 0.44 0.32 0.69
55kDa—
LRG1
TSG101
40kDa—

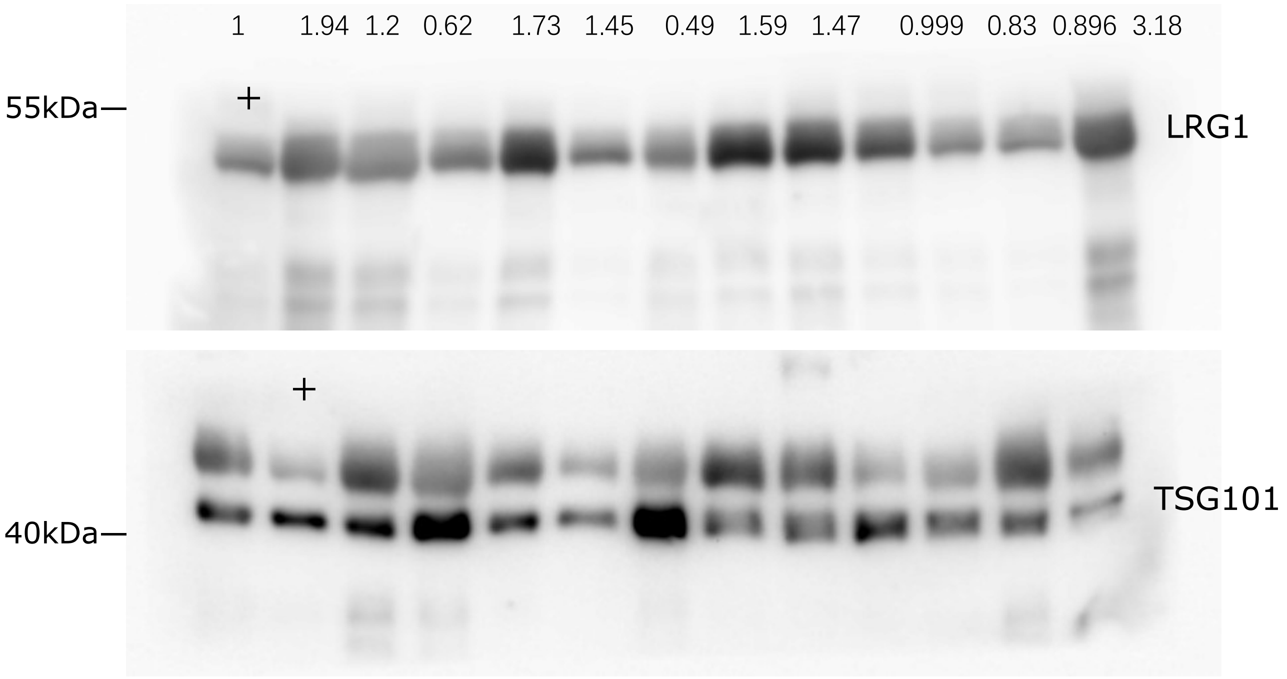
1 1.94 1.2 0.62 1.73 1.45 0.49 1.59 1.47 0.999 0.83 0.896 3.18
55kDa—
+
LRG1
+
TSG101
40kDa—

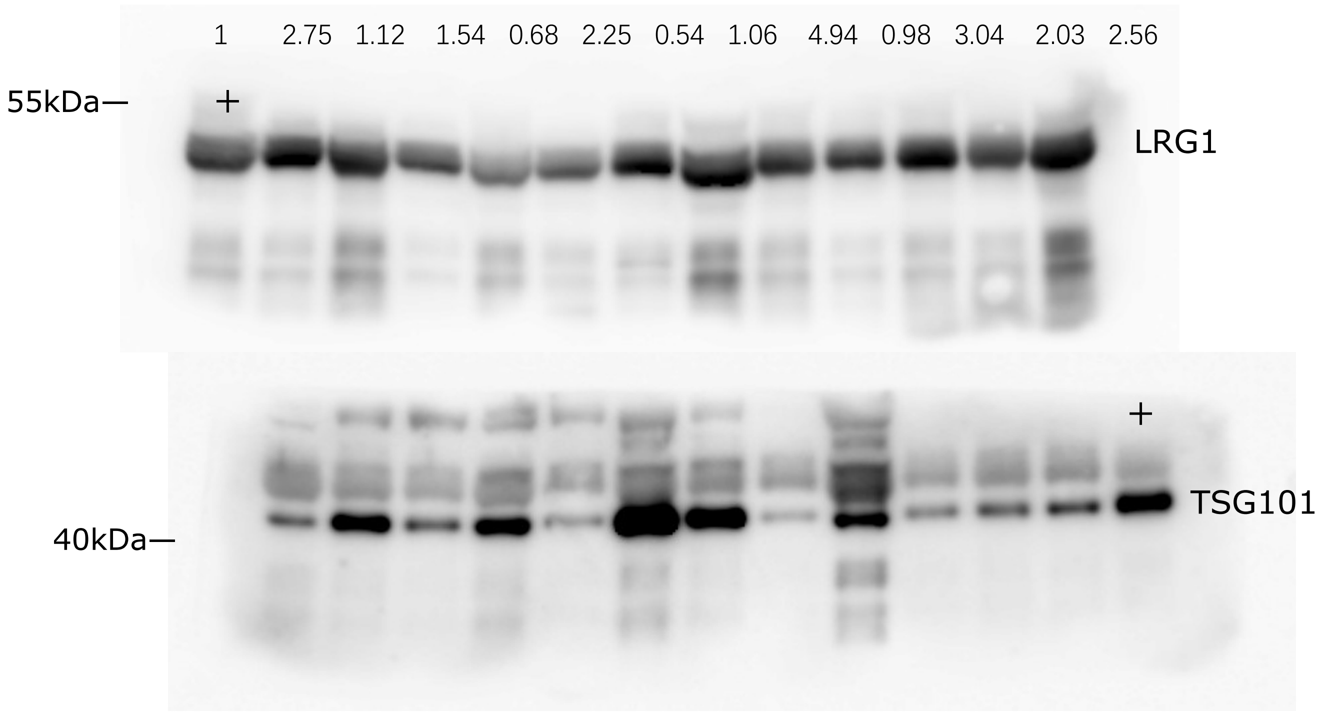
1 2.75 1.12 1.54 0.68 2.25 0.54 1.06 4.94 0.98 3.04 2.03 2.56
55kDa—
+
LRG1
+
TSG101
40kDa—

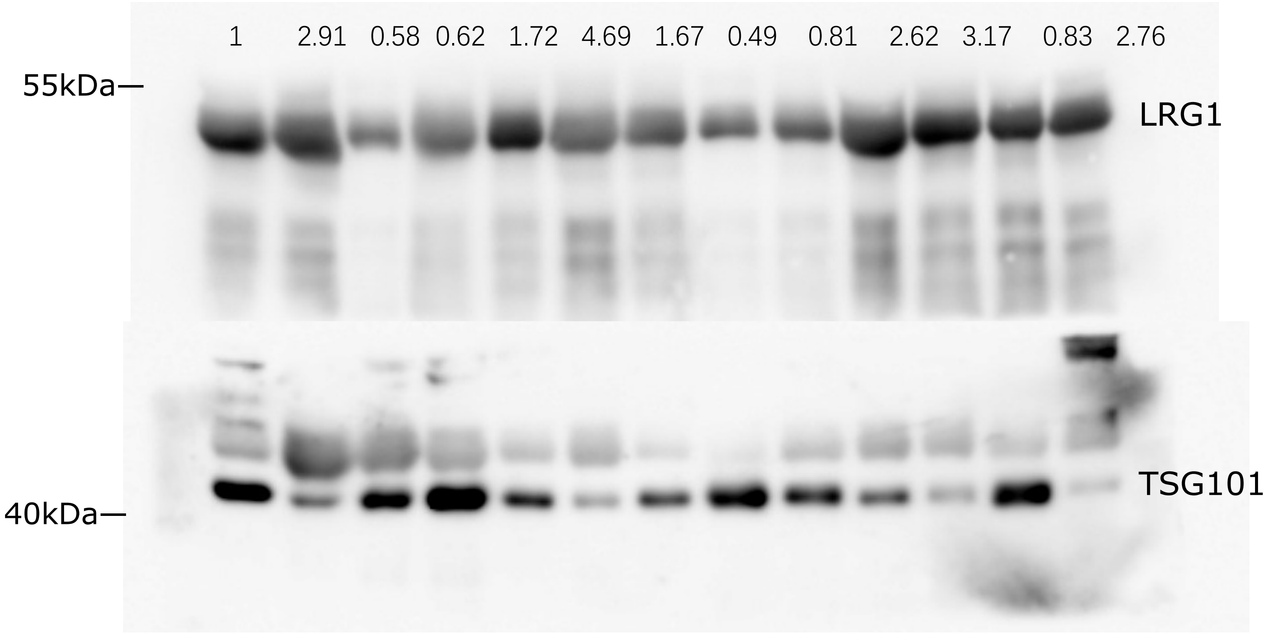
1 2.91 0.58 0.62 1.72 4.69 1.67 0.49 0.81 2.62 3.17 0.83 2.76
55kDa—
LRG1
TSG101
40kDa—

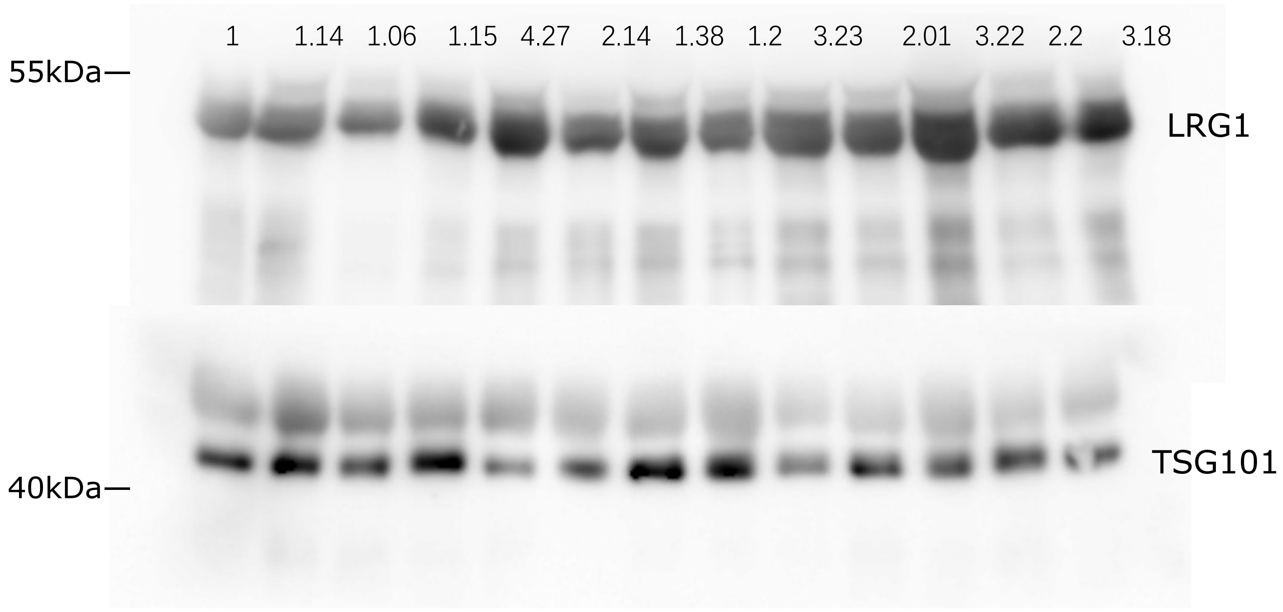
1 1.14 1.06 1.15 4.27 2.14 1.38 1.2 3.23 2.01 3.22 2.2 3.18
55kDa—
LRG1
TSG101
40kDa—

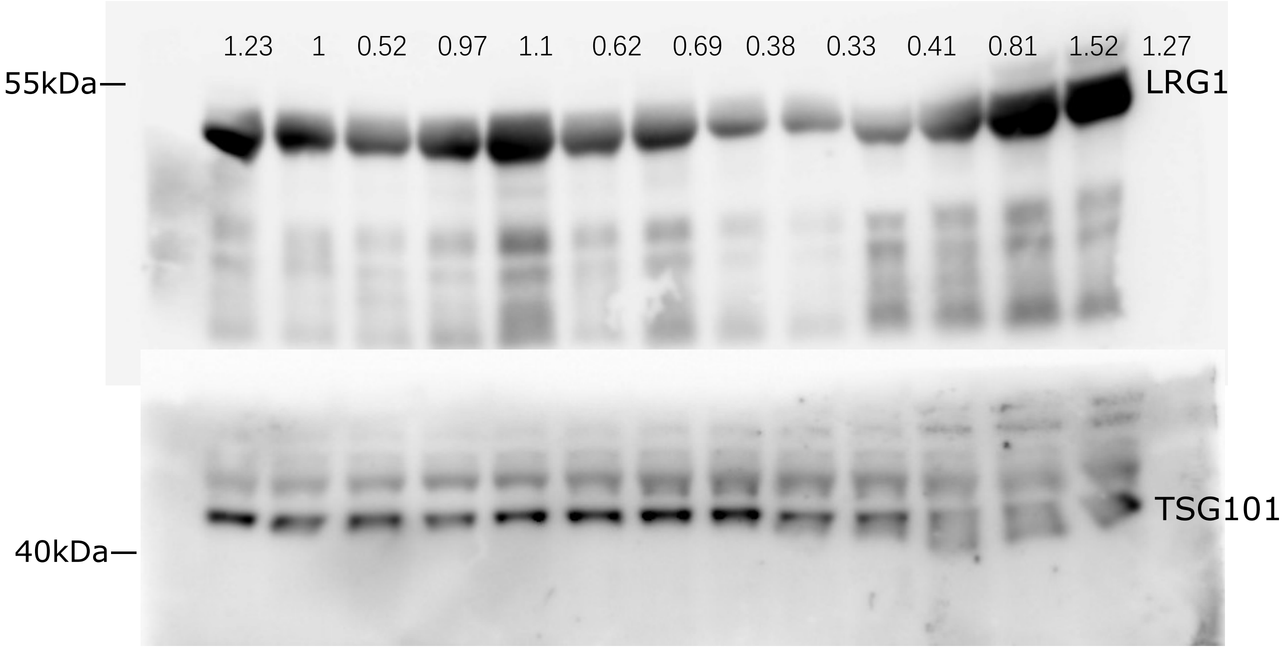
1.23 1 0.52 0.97 1.1 0.62 0.69 0.38 0.33 0.41 0.81 1.52 1.27
55kDa—
LRG1
TSG101
40kDa—

Extended Data Fig.1b

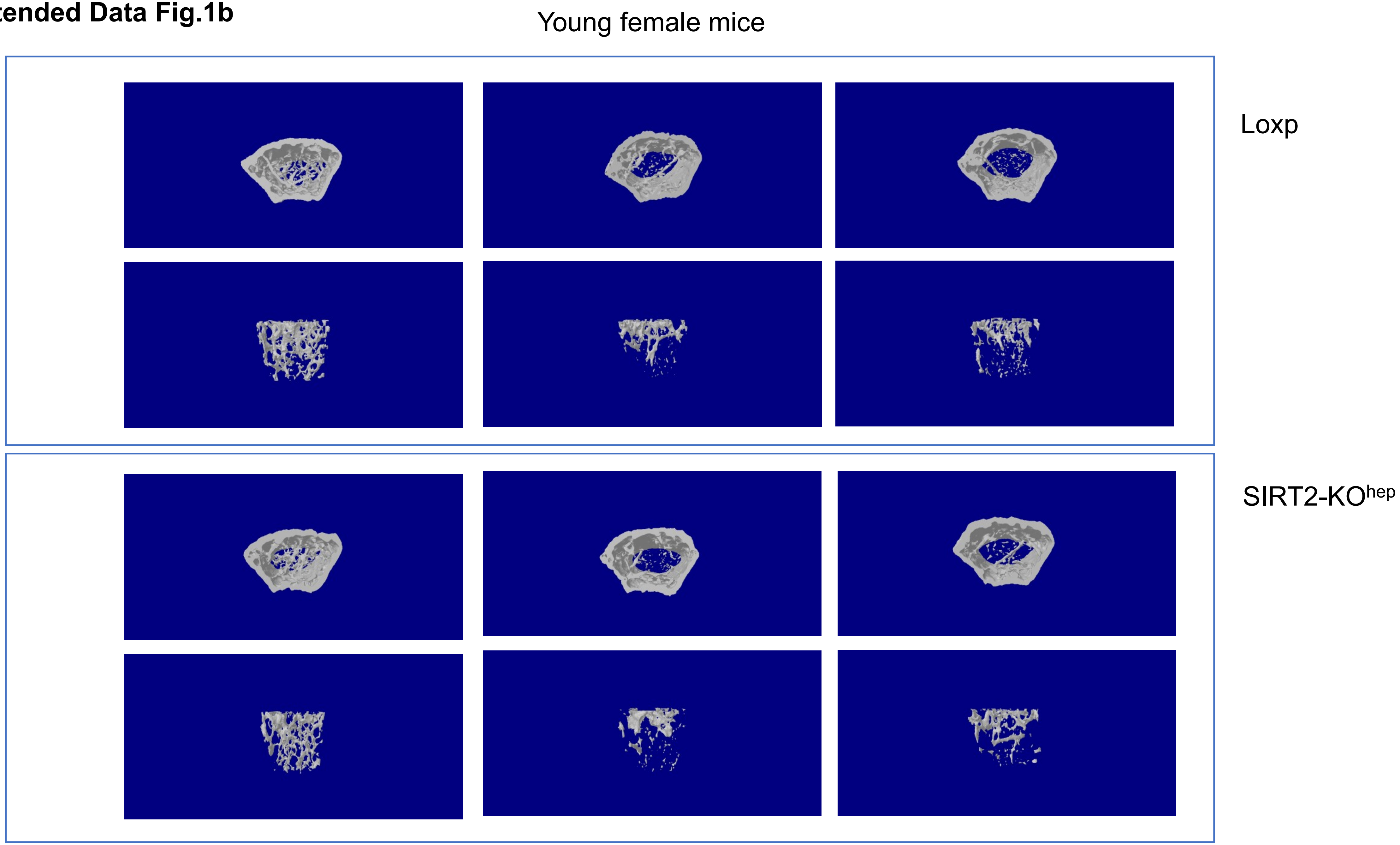

Extended Data Fig.1e

Young male mice-Loxp

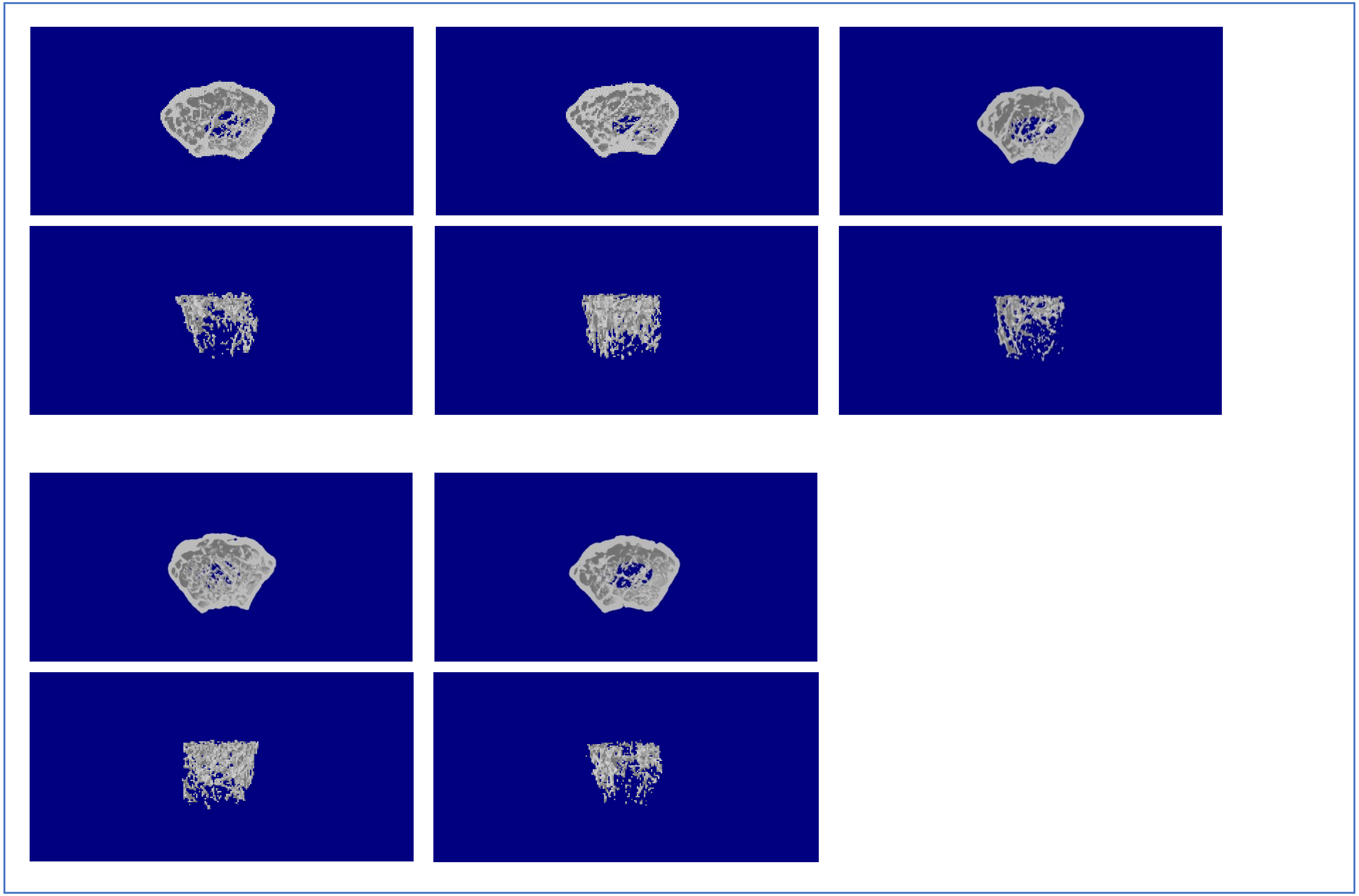

Extended Data Fig.1e

Young male mice-SIRT2-KO^{hep}

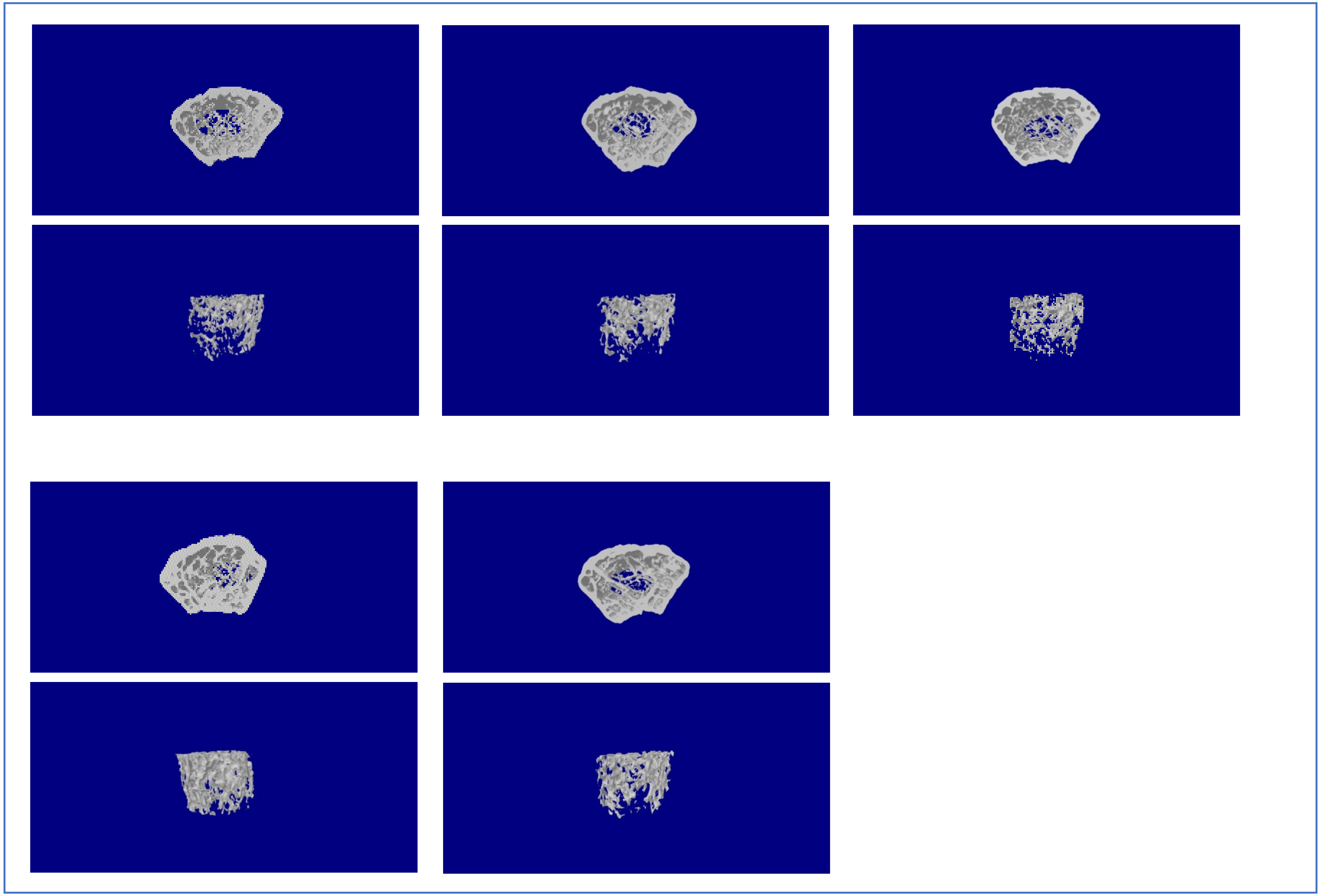

Extended Data Fig.1i H&E staining on paraffin-embedded femur sections in aged mice

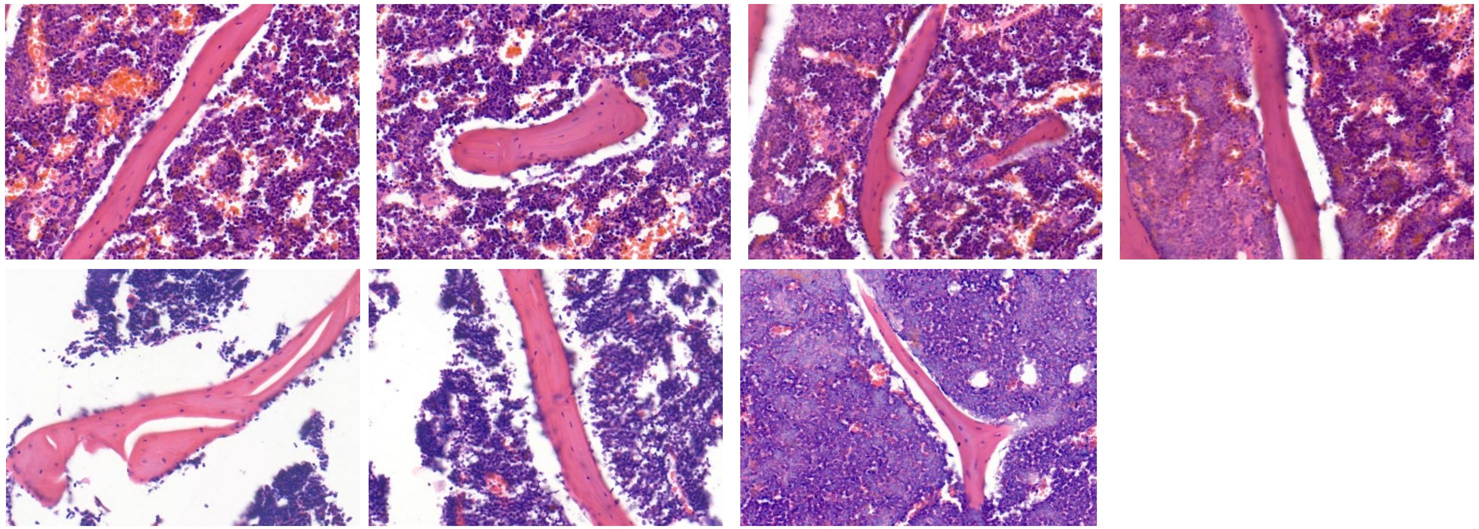

aged female mice
-Loxp

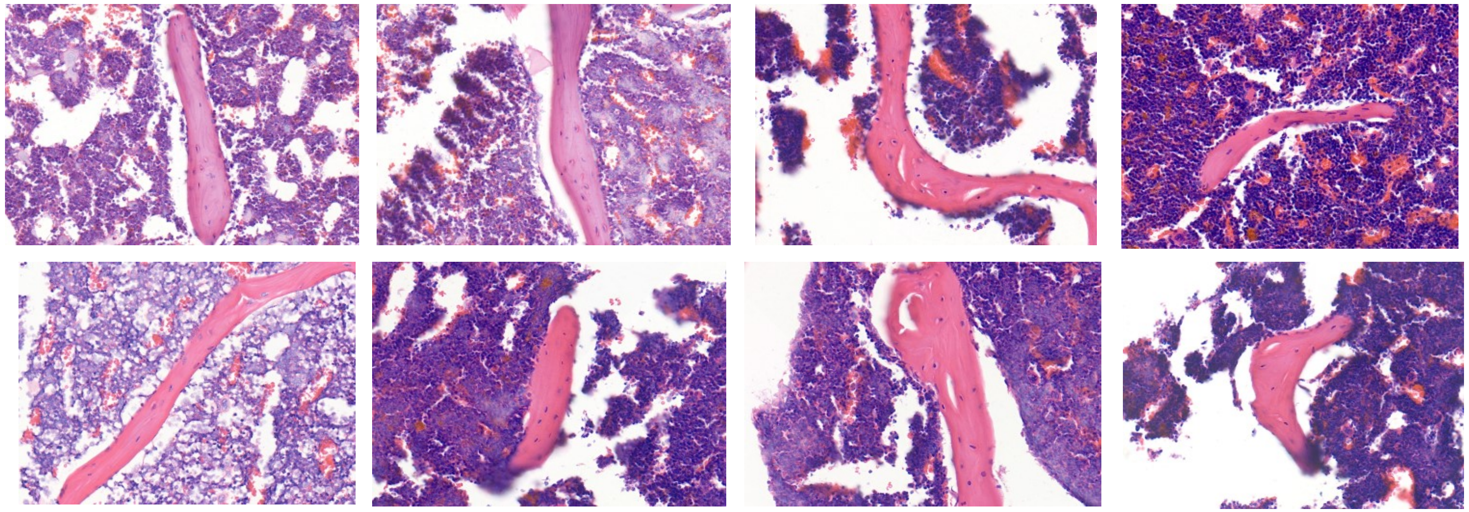

Aged female mice
-SIRT2-KO^{hep}

Extended Data Fig.1m H&E staining on paraffin-embedded femur sections in aged mice

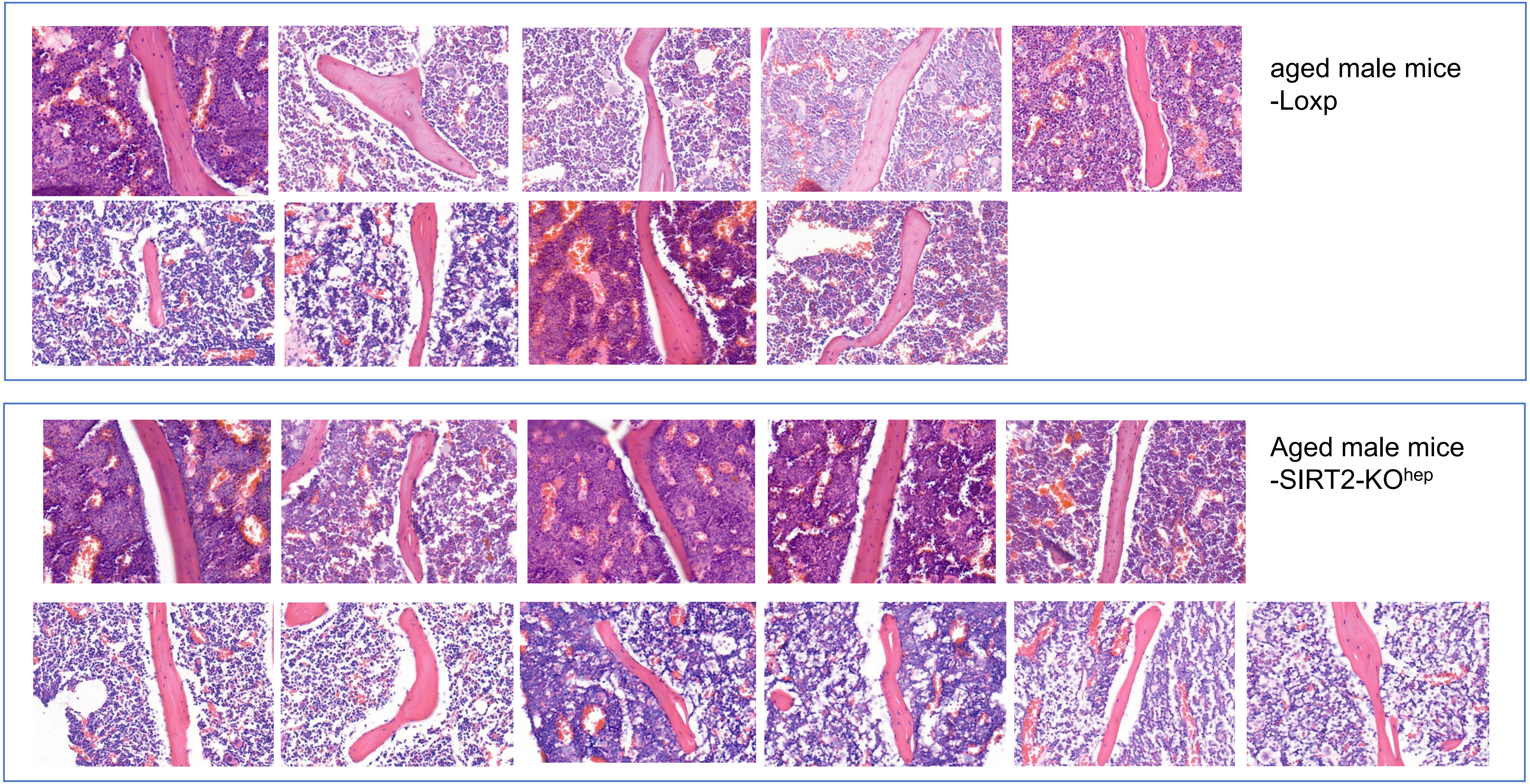

Extended data fig.2a ALP and ARS staining after osteogenesis from BM-MSCs treated with plasma

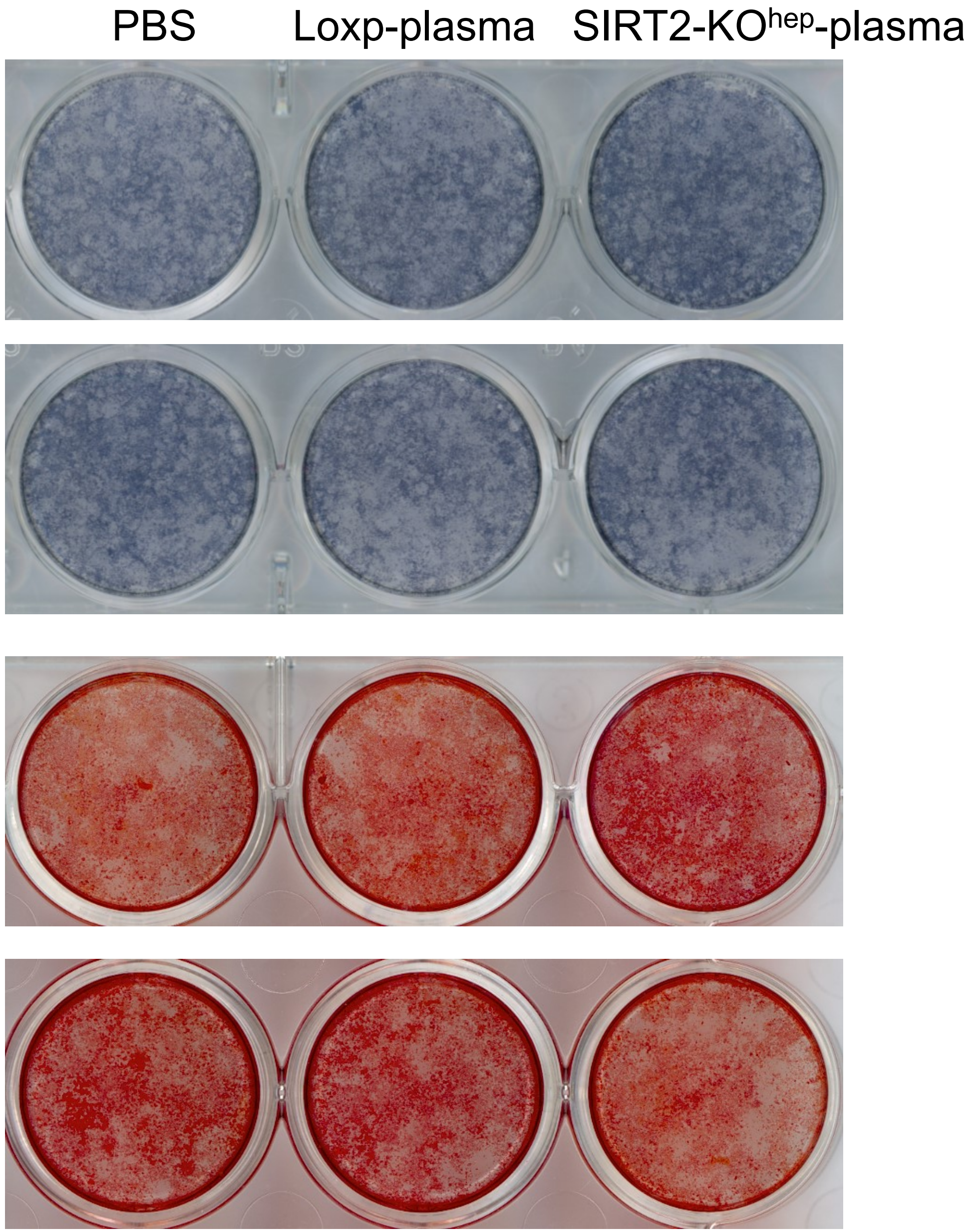

Extended data Fig.4a TRAP staining images of osteoclasts treated with the sEVs derived from AML12 hepatocytes

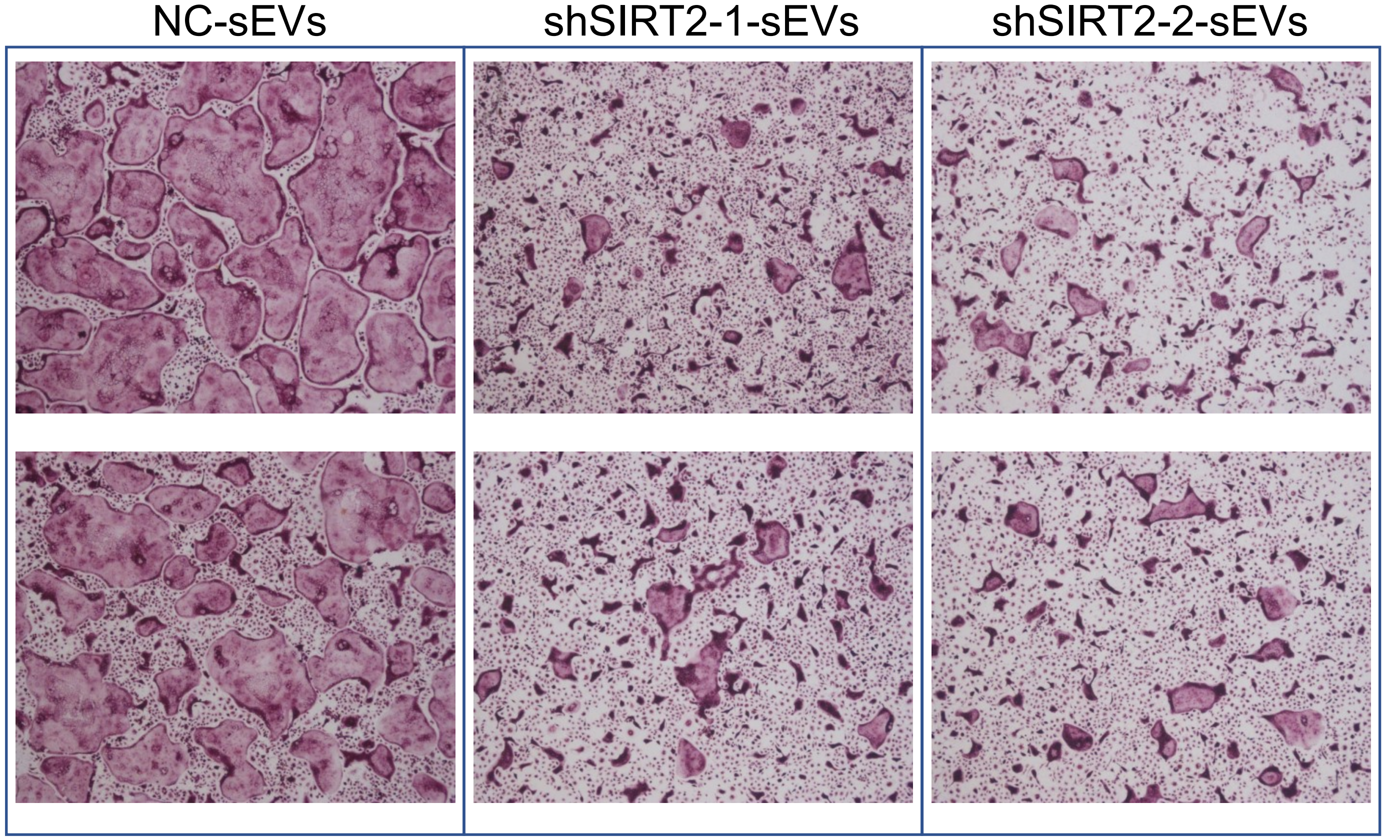

Extended Data Fig.6a TRAP staining on paraffin-embedded femur sections in young mice

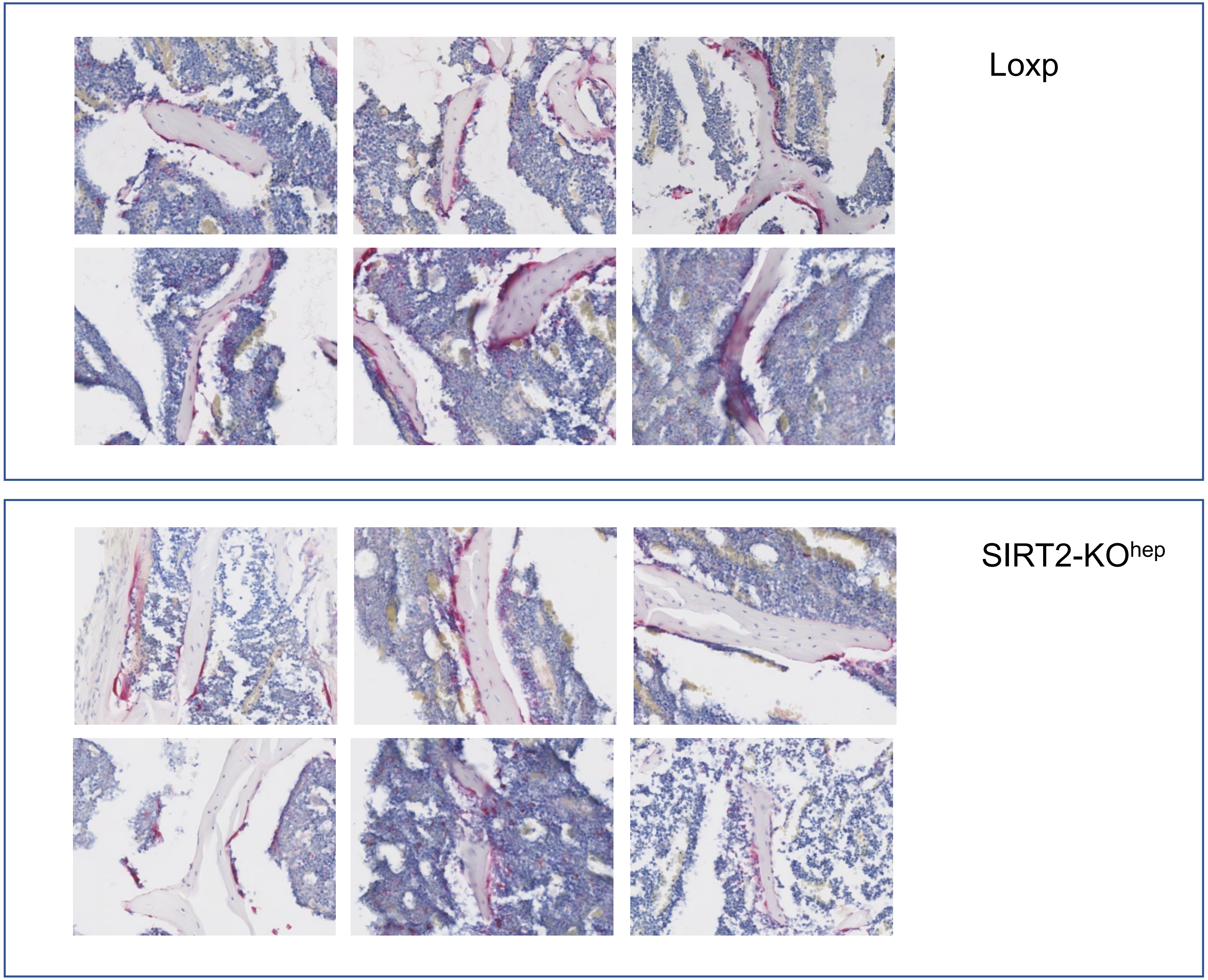

Extended Data Fig.6c immunofluorescence images of murine femurs in young mice

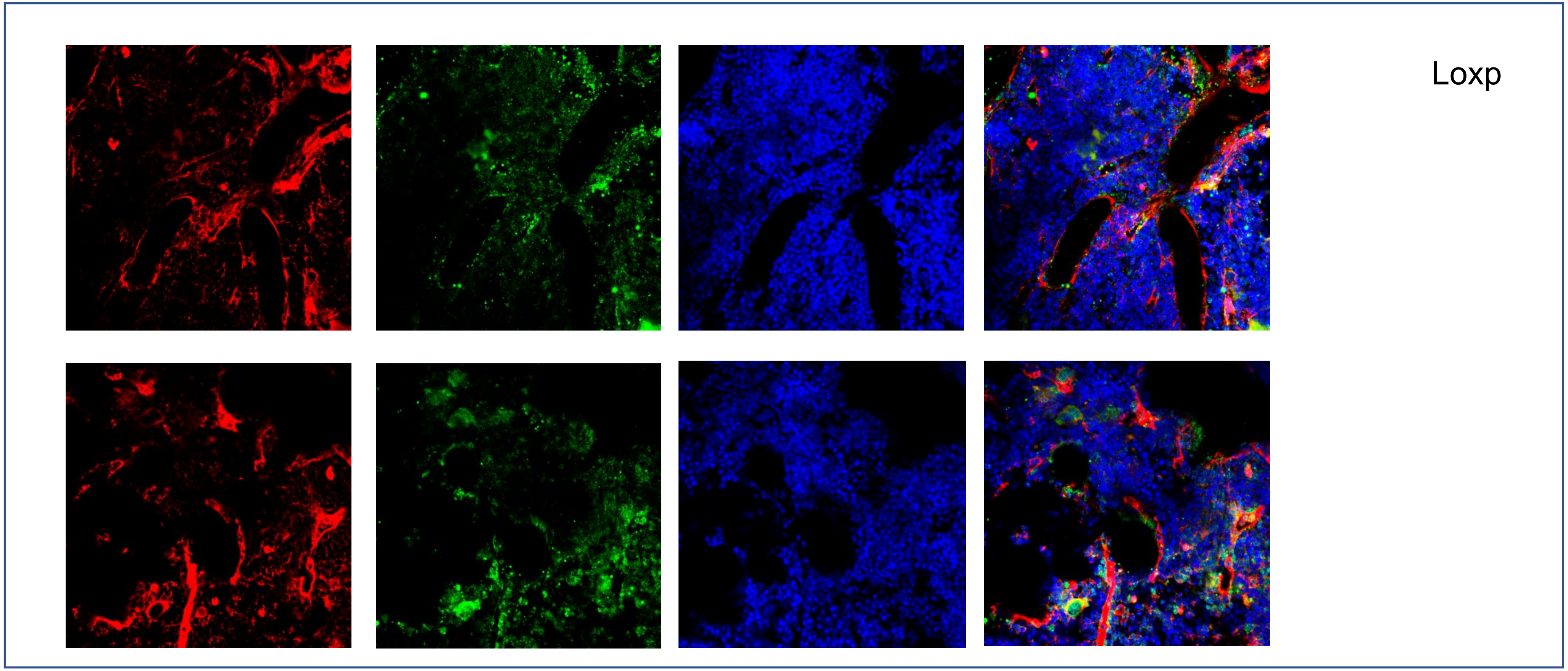

Extended Data Fig.6c immunofluorescence images of murine femurs in young mice

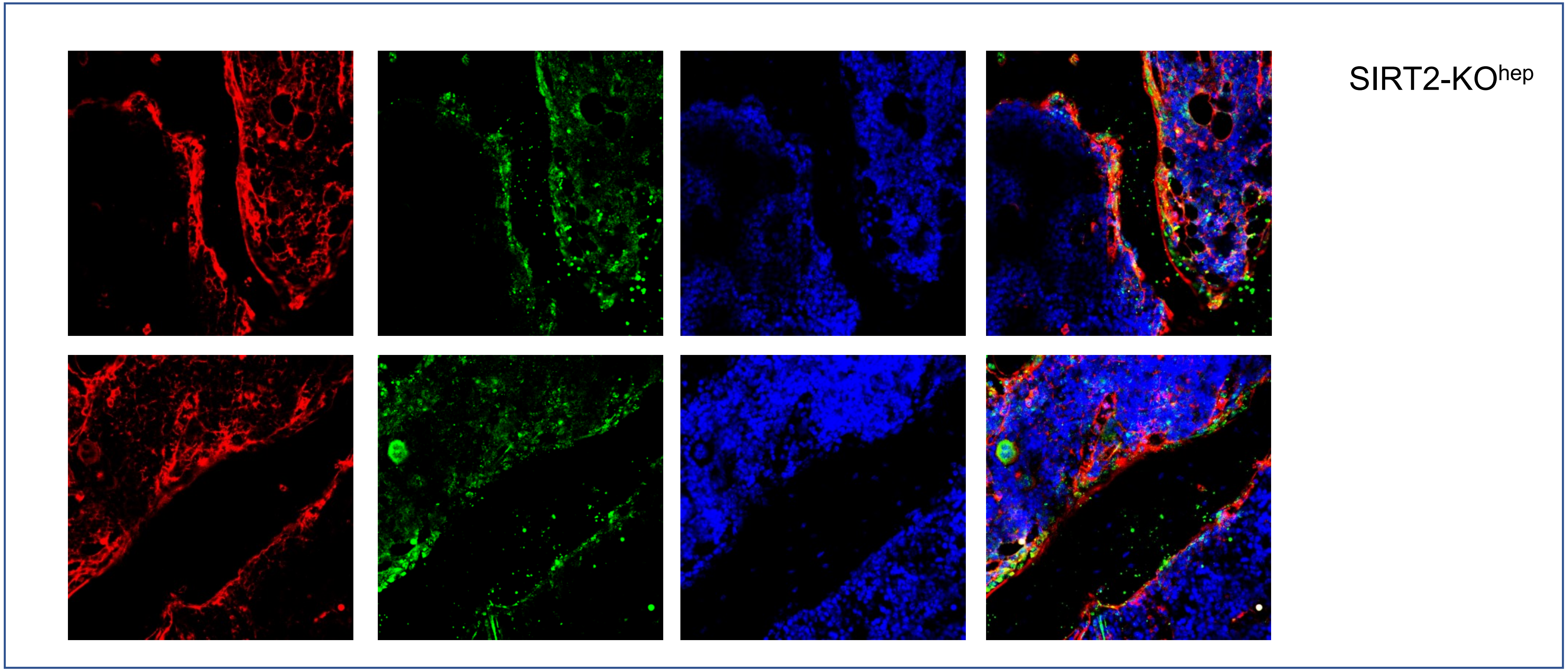

Extended Data Fig.7d IHC detection of CD31 in the paraffin-embedded bone section of distal femur of aged mice

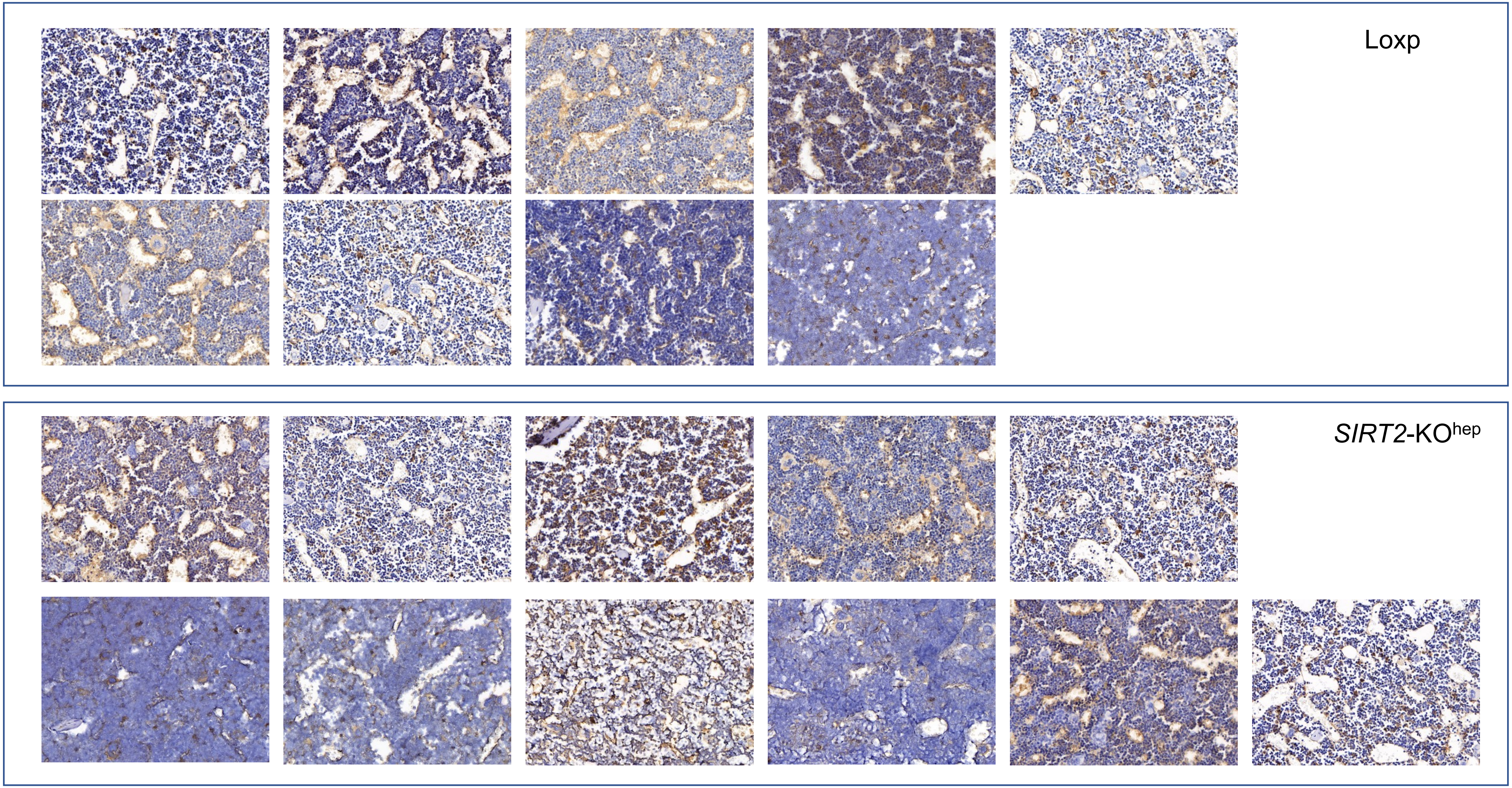

Extended data fig.8a TRAP staining images of BMDMs treated with RANKL and AGK2

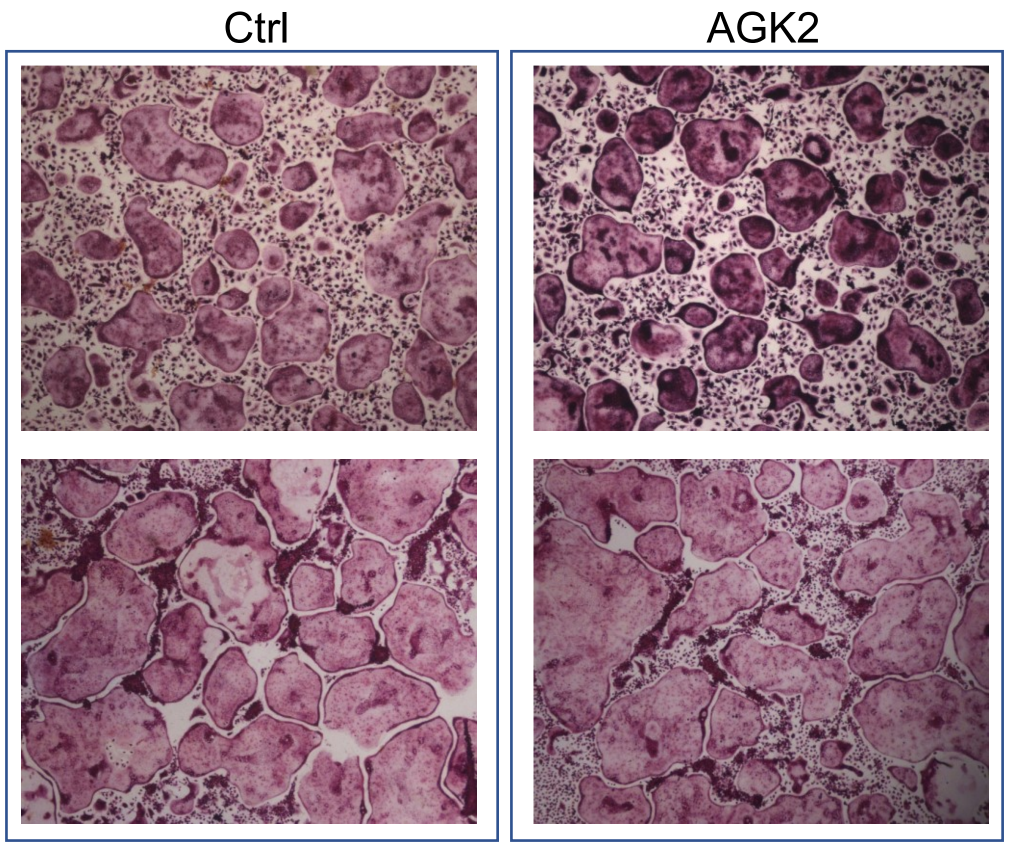

Extended data fig.9e

aged female mice loxp

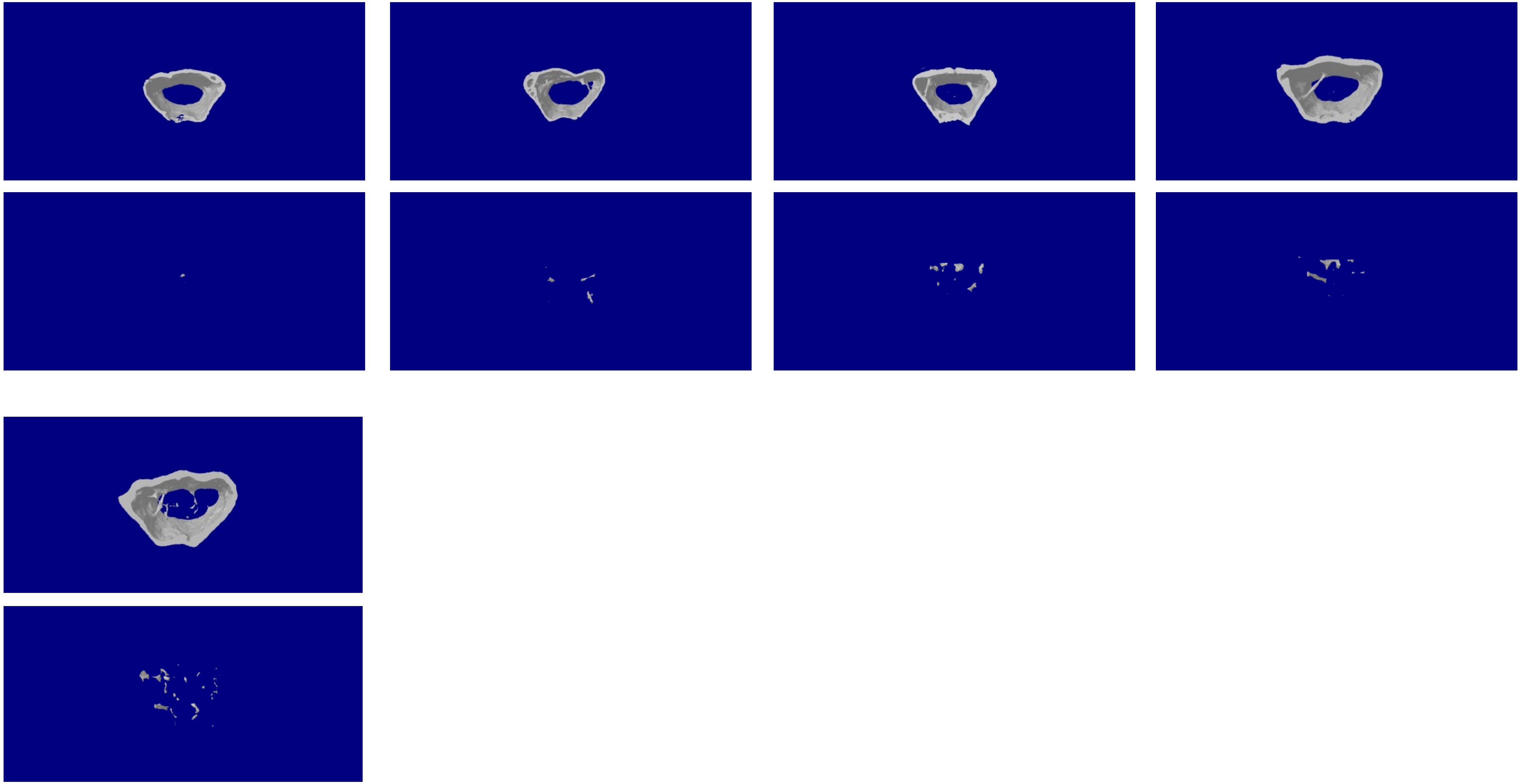

Extended data fig.9e

aged female mice SIRT2-KO^{lyz}

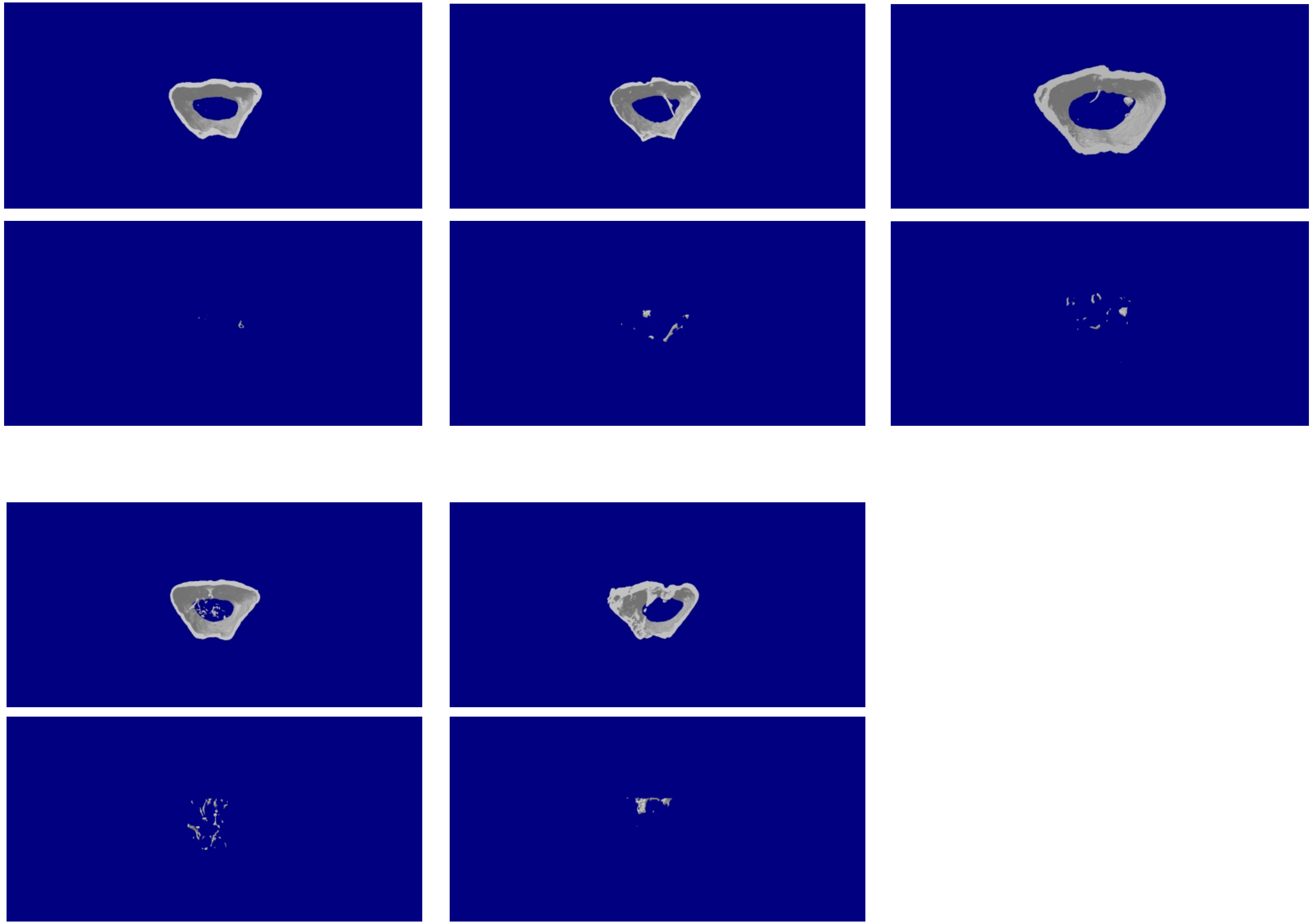

Extended data fig.9g

aged male mice loxp

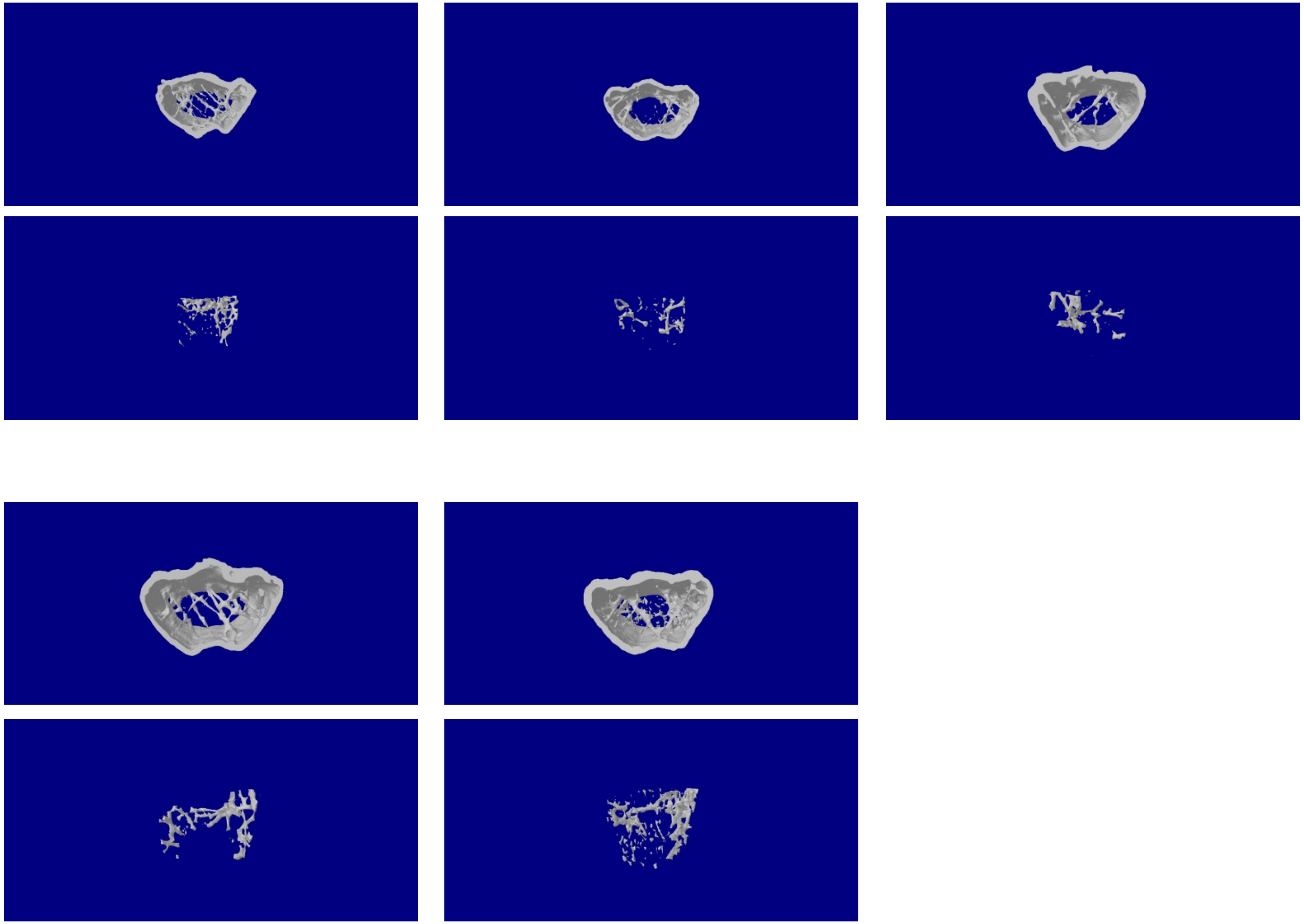

Extended data fig.9g

aged male mice SIRT2-KOlyz

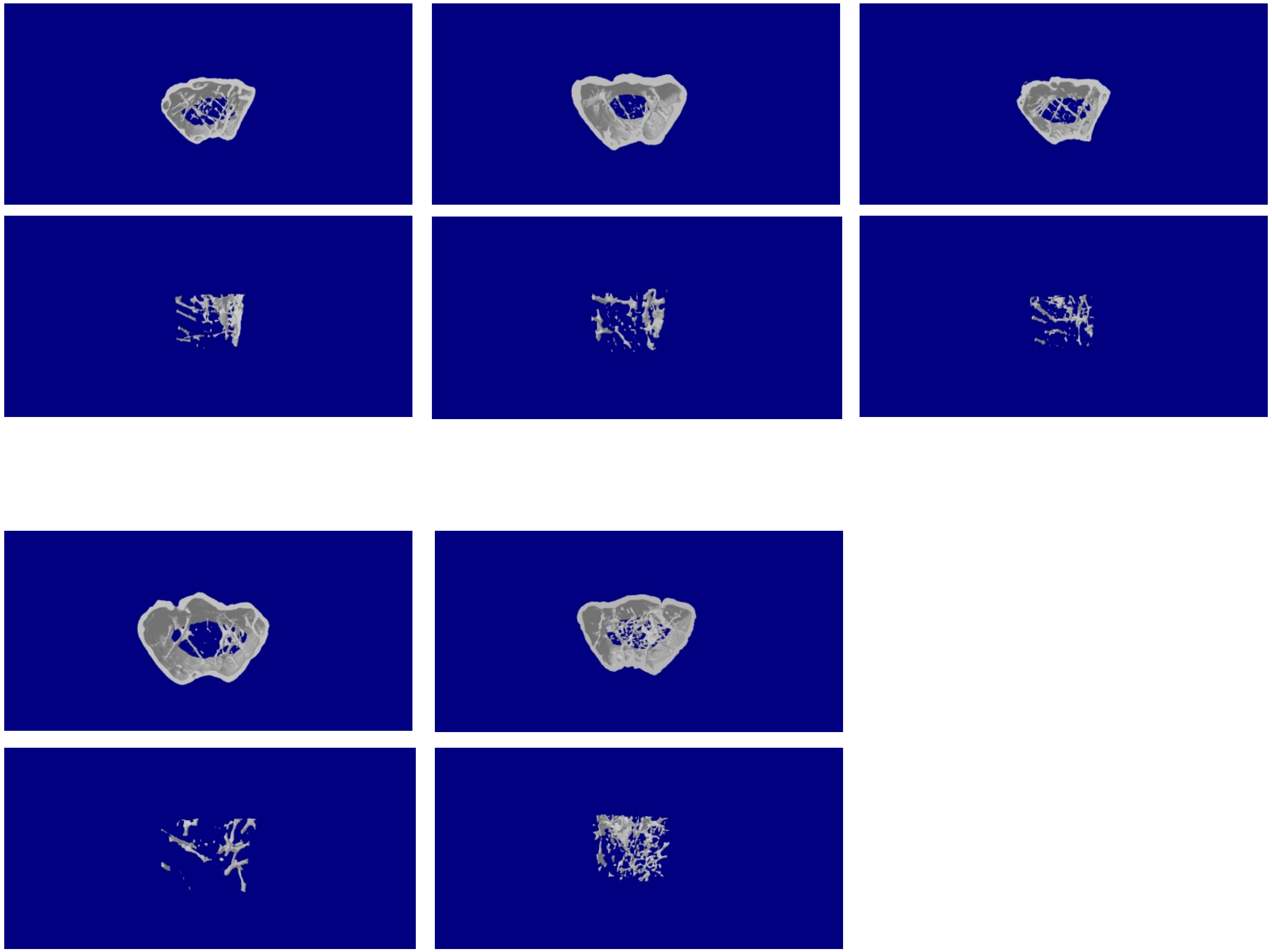

Extended data fig.9i

OVX loxp

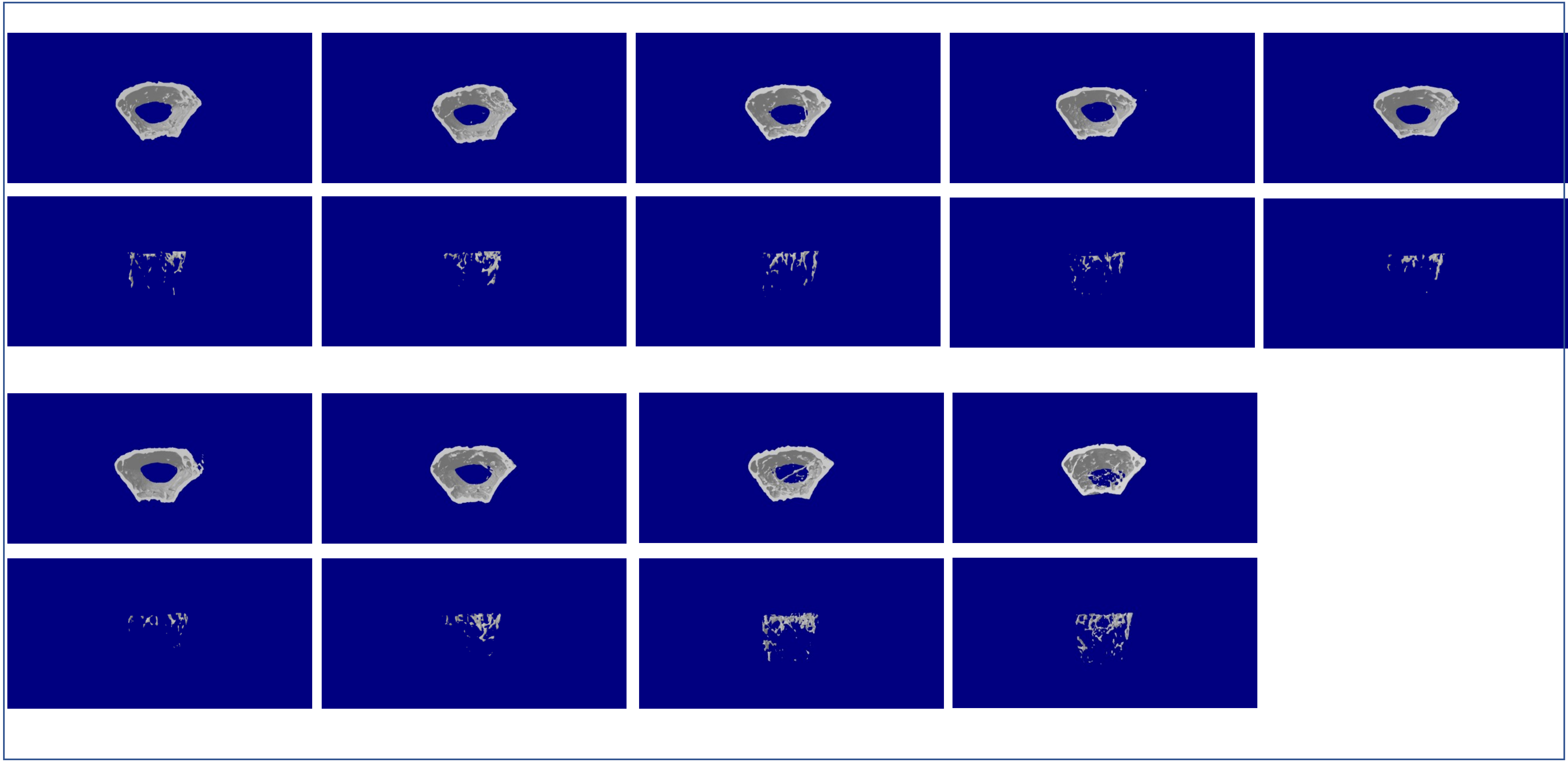

Extended data fig.9i

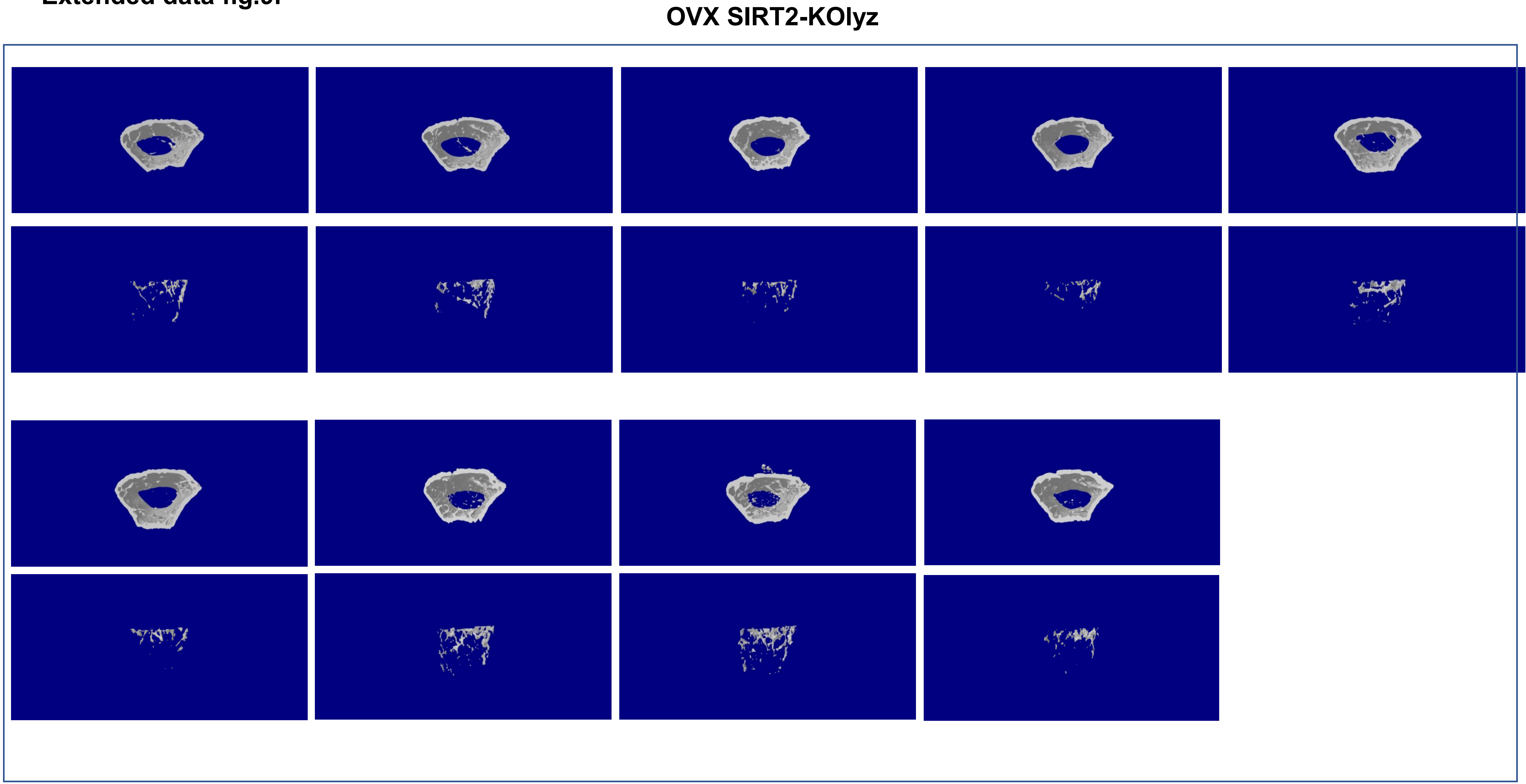

Extended data fig.9k BMDMs were isolated from LoxP and SIRT2-KOlyz mice and cultured with RANKL to generate osteoclasts.

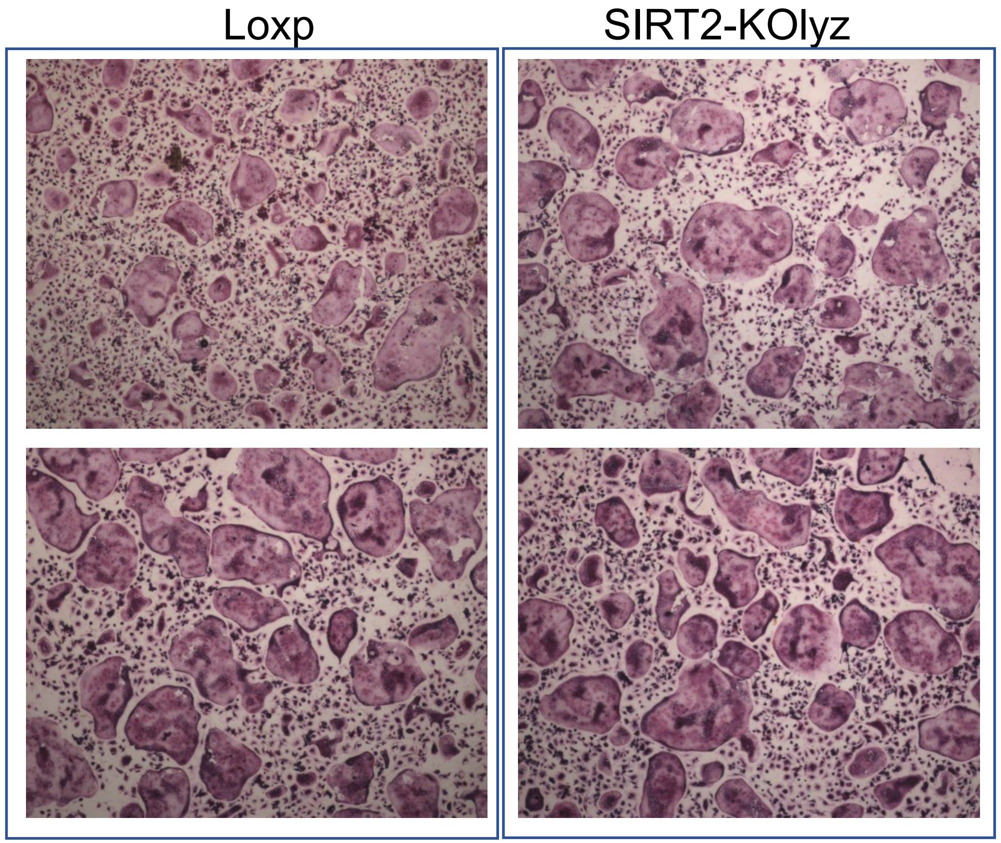

Extended data fig.9n BMDMs were isolated from LoxP and SIRT2-KOlyz mice and cultured with RANKL to generate osteoclasts.

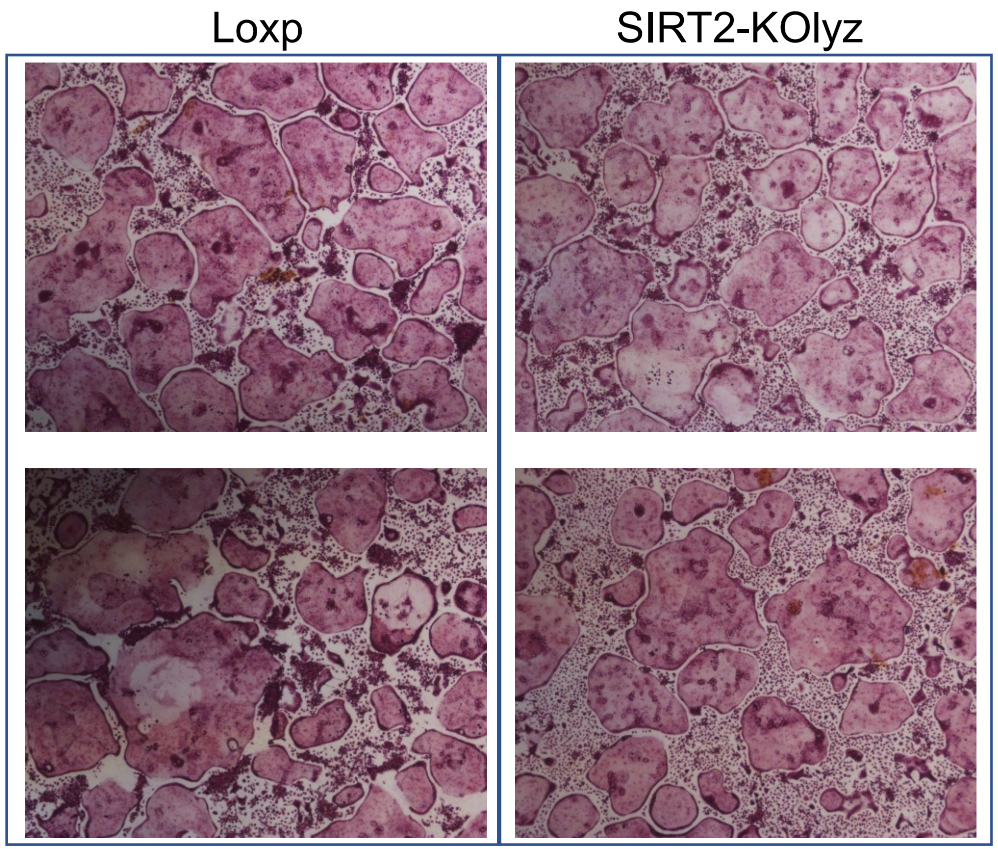

Extended data fig.10 TRAP staining images of human PBMCs cultured with RANKL and sEVs from HepG2 cells treated with control or AGK2

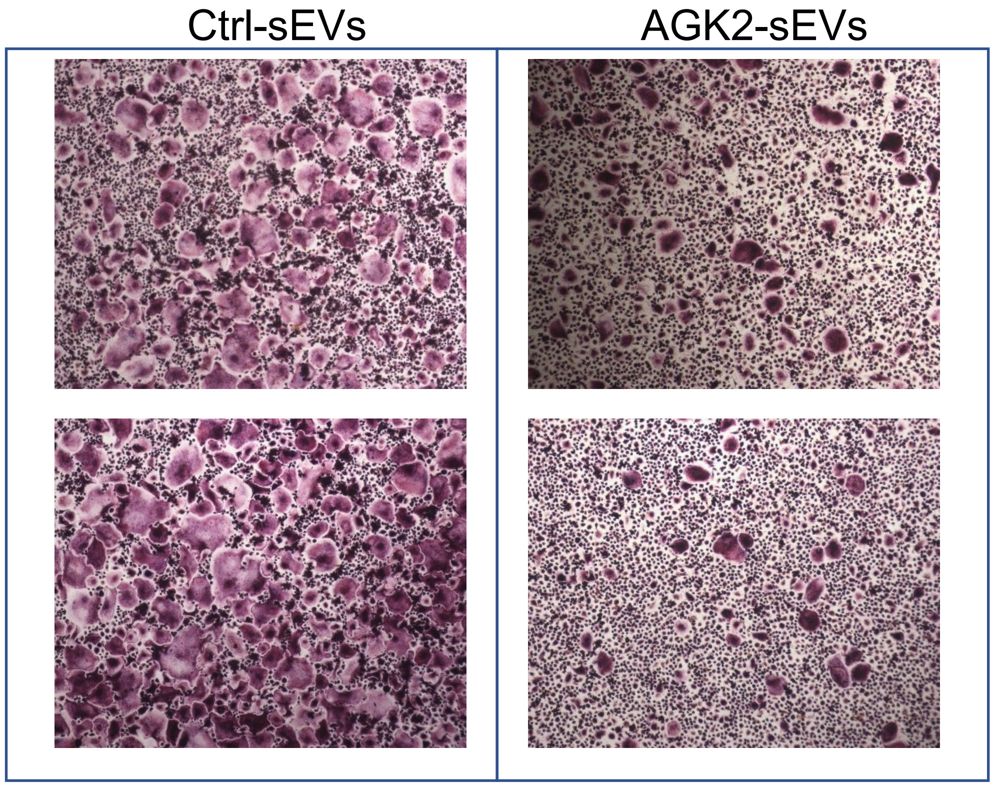

Supplement: Supplementary file 1 — Additional images of replicates. [file 42255_2023_803_MOESM1_ESM.pdf]
