# Supplementary material for: SIRT2 regulates extracellular vesicle-mediated liver–bone communication
Source: Nat Metab. 2023 May 15;5(5):821–41. doi: 10.1038/s42255-023-00803-0 (PMC10229428; doi:10.1038/s42255-023-00803-0)
Supplement: Supplementary file 2 — Reporting Summary [file 42255_2023_803_MOESM2_ESM.pdf]

nature portfolio

Corresponding author(s): Ming He

Last updated by author(s): Mar 30, 2023

Reporting Summary

Nature Portfolio wishes to improve the reproducibility of the work that we publish. This form provides structure for consistency and transparency in reporting. For further information on Nature Portfolio policies, see our Editorial Policies and the Editorial Policy Checklist.

Statistics

For all statistical analyses, confirm that the following items are present in the figure legend, table legend, main text, or Methods section.

n/a	Confirmed	
☐	☒	The exact sample size (*n*) for each experimental group/condition, given as a discrete number and unit of measurement
☐	☒	A statement on whether measurements were taken from distinct samples or whether the same sample was measured repeatedly
☐	☒	The statistical test(s) used AND whether they are one- or two-sided<br>*Only common tests should be described solely by name; describe more complex techniques in the Methods section.*
☐	☒	A description of all covariates tested
☐	☒	A description of any assumptions or corrections, such as tests of normality and adjustment for multiple comparisons
☐	☒	A full description of the statistical parameters including central tendency (e.g. means) or other basic estimates (e.g. regression coefficient) AND variation (e.g. standard deviation) or associated estimates of uncertainty (e.g. confidence intervals)
☐	☒	For null hypothesis testing, the test statistic (e.g. *F*, *t*, *r*) with confidence intervals, effect sizes, degrees of freedom and *P* value noted<br>*Give P values as exact values whenever suitable.*
☒	☐	For Bayesian analysis, information on the choice of priors and Markov chain Monte Carlo settings
☒	☐	For hierarchical and complex designs, identification of the appropriate level for tests and full reporting of outcomes
☒	☐	Estimates of effect sizes (e.g. Cohen's *d*, Pearson's *r*), indicating how they were calculated

Our web collection on statistics for biologists contains articles on many of the points above.

Software and code

Policy information about availability of computer code

Data collection	X-ray micro-tomography (Skyscan1076, Bruker micro-CT, Belgium), Image Quant LAS 4000 Mini (GE Healthcare Bio-Sciences AB, Uppsala, Sweden), ABI 7300 PCR system (Applied Biosystems), Nikon Laser Confocal Scanning Microscope (JAPAN), Bioluminescence imaging, Image J (Media Cybernetics), Origin(OriginLab), DNBSEQ500 platform.
Data analysis	GraphPad Prism 9.0, Origin 2021, Image J v.1.8.0, DAVID(online platform:https://david.ncifcrf.gov/tools.jsp), Cistrome Data Browser(online platform:http://cistrome.org/db/#/), SPSS 27.0.

For manuscripts utilizing custom algorithms or software that are central to the research but not yet described in published literature, software must be made available to editors and reviewers. We strongly encourage code deposition in a community repository (e.g. GitHub). See the Nature Portfolio guidelines for submitting code & software for further information.

Data

Policy information about availability of data

All manuscripts must include a data availability statement. This statement should provide the following information, where applicable:

- Accession codes, unique identifiers, or web links for publicly available datasets
- A description of any restrictions on data availability
- For clinical datasets or third party data, please ensure that the statement adheres to our policy

The GEO accession numbers for our RNA-seq data are GSE228204. The mass spectrometry proteomics data have been deposited to the ProteomeXchange

Consortium (http://proteomecentral.proteomexchange.org) via the iProX partner repository with the dataset identifier PXD041145. Materials, reagents or other experimental data are available upon request. Source data are provided with this paper.

Human research participants

Policy information about studies involving human research participants and Sex and Gender in Research.

Reporting on sex and gender	For patients, in the comparison of the protein level of plasma-sEVs-LRG1 between normal BMD group and osteoporosis patients, only female patients met the diagnosis were included because there are no male patients diagnosed as osteoporosis in our collected data. In the correlation analysis between the expression of plasma-sEVs-LRG1 and BMD, β-CTX, PINP or BALP, due to the small number of male patient sample cases, both female and male patients are included for statistical analysis
Population characteristics	Blood samples from patients with osteoporosis or normal bone mass were collected from patients in both sex aged 60-70 years. Human liver tissues were collected from patients in both sex aged 28-75 years.
Recruitment	All participants voluntarily participated in the study without any self-selection bias or other biasesand provided informed consent.
Ethics oversight	The study was approved by the Medical Ethic Committee of Shanghai JiaoTong University School of Medicine and Institutional Review Board of The Affiliated Hospitals of Youjiang Medical University for Nationalities.

Note that full information on the approval of the study protocol must also be provided in the manuscript.

Field-specific reporting

Please select the one below that is the best fit for your research. If you are not sure, read the appropriate sections before making your selection.

☒ Life sciences ☐ Behavioural & social sciences ☐ Ecological, evolutionary & environmental sciences

For a reference copy of the document with all sections, see nature.com/documents/nr-reporting-summary-flat.pdf

Life sciences study design

All studies must disclose on these points even when the disclosure is negative.

Sample size	Sample sizes were chosen to ensure the possibility of statistical analysis and to simultaneously minimize the use of animals in accordance with animal care guidelines. No statistical methods were used to predetermine sample size.
Data exclusions	No data were excluded from the study.
Replication	All animal experimental findings were reproduced as biological replicates at the value stated in figure legends and in vitro experiments were performed with at least two biological replicates. All results are reproducible.
Randomization	All samples/participants and research animals were randomly assigned to groups
Blinding	Investigators were blinded to group allocation during data collection. All measurements and analyzes were objective.

Reporting for specific materials, systems and methods

We require information from authors about some types of materials, experimental systems and methods used in many studies. Here, indicate whether each material, system or method listed is relevant to your study. If you are not sure if a list item applies to your research, read the appropriate section before selecting a response.

Materials & experimental systems

n/a	Involved in the study	
☐	☒	Antibodies
☐	☒	Eukaryotic cell lines
☒	☐	Palaeontology and archaeology
☐	☒	Animals and other organisms
☒	☐	Clinical data
☒	☐	Dual use research of concern

Methods

n/a	Involved in the study	
☒	☐	ChIP-seq
☒	☐	Flow cytometry
☒	☐	MRI-based neuroimaging

Antibodies

Antibodies used	The following primary antibodies used for western blot experiments:<br>TSG101 rabbit polyclonal antibody (1:1000; Proteintech,28283-1-AP),<br>HSP70 rabbit polyclonal antibody (1:1000; Proteintech, 10995-1-AP),<br>Alix rabbit polyclonal antibody (1:1000; Proteintech, 12422-1-AP),<br>HRP-conjugated β-actin (1:5000; MBL, PM053-7)<br>SIRT2 rabbit polyclonal antibody (1:500; Sigma, S8447),<br>LRG1 Rabbit polyclonal antibody (1:250; Abcam, ab231188),<br>HRP-conjugated β-tubulin mouse monoclonal antibody (1:2000; Proteintech, HRP-66240),<br>Anti-rabbit IgG, HRP-linked Antibody (1:5000; Cell Signaling Technology, #7074),<br>Monoclonal anti FLAG M2 mouse Antibody (1:2500; Sigma, F1804),<br>Anti-mouse IgG, HRP-linked Antibody (1:5000; Cell Signaling Technology, #7076),<br>Acetyl-Histone H4(lys16) rabbit mAb (1:1000; Cell Signaling Technology, #13534),<br>Histone H4 rabbit ployclonal antibody (1:500; Proteintech, 16047-1-AP),<br>p-p65 rabbit mAb (1:1000; Cell Signaling Technology, #3033),<br>p65 rabbit mAb (1:1000; Cell Signaling Technology, #8242),<br>p-Smad2/3 rabbit mAb (1:500; Cell Signaling Technology, #8828),<br>T-Smad2/3 rabbit mAb (1:1000; Cell Signaling Technology, #8685),<br>p-Smad1/5 rabbit mAb (1:500; Cell Signaling Technology, #9516),<br>T-Smad1 rabbit mAb (1:1000; Cell Signaling Technology, #6944),<br>T-Smad5 rabbit mAb (1:1000; Cell Signaling Technology, #12534),<br>Saa1/Saa2 rabbit mAb (1:1000; Abcam, ab199030)<br>goat polyclonal anti-LaminB (1:500; Santa Cruz, sc6216),<br>anti-goat IgG (1:2000; Millipore AP106P)<br>The following antibodies used for immunohistochemistry:<br>CD31 Goat pAb (1:200, Servicebio Biotechnology, Inc, GB13063),<br>HRP conjugated Rabbit Anti-Goat IgG (H+L) (1:200, Servicebio Biotechnology, Inc, GB23204),<br>SIRT2 rabbit polyclonal antibody (1:250 for patients and 1:200 for mice, Sigma, S8447).<br>HRP conjugated Goat Anti-Rabbit IgG (H+L) (1:200, Servicebio Biotechnology, Inc, GB23303)<br>The following antibody used for ChIP analysis:<br>Acetyl-Histone H4(lys16) rabbit mAb (1:1000; Cell Signaling Technology, #13534).<br>The following antibodies used for immunofluorescence analysis:<br>mouse monoclonal anti-CTSK antibody (1:100, Santa, sc-48353)<br>rabbit polyclonal anti-LRG1 antibody (1:50, ABclonal, A7850)<br>Texas Red Goat anti-mouse IgG H&L antibody (1:1000, Abcam, ab6787),<br>Alexa Fluor 488 donkey anti-rabbit IgG(H+L) Antibody (1:1000, Thermo Fisher Scientific, A-21206)<br>p65 rabbit mAb (1:100, Cell Signaling Technology, 8242)<br>rabbit polyclonal anti-LPHN2 antibody (1:100, Abcam, ab139498),<br>Alexa Fluor594 Donkey anti-Rabbit IgG (H+L) Antibody(1:200, Thermo Fisher Scientific, A-21207).
Validation	All antibodies are from commercial sources (Proteintech, Cell Signaling Technology, Sigma, Abcam, Santa cruz, Millipore, Servicebio, ABclonal and Thermo Fisher). They are all commercial antibodies with validations available either as proofs or publication references on the manufacturers website.

Eukaryotic cell lines

Policy information about cell lines and Sex and Gender in Research

Cell line source(s)	Mouse hepatic cell lines (AML12), HEK293T cells, Raw264.7 cells and human hepatic cell lines (HepG2) were obtained from American Type Culture Collection (ATCC, USA). Primary bone marrow mesenchymal stem cells (BM-MSCs), bone marrow derived monocytes (BMDMs) and Primary hepatocytes were obtained from the indicated mouse strains.
Authentication	AML12cells HEK293T cells, Raw264.7 cells and HepG2 cells were authenticated by STR profiling.
Mycoplasma contamination	All the cell lines were tested for mycoplasma contamination and tested negative.
Commonly misidentified lines<br>(See ICLAC register)	No commonly misidentified cell lines were used.

Animals and other research organisms

Policy information about studies involving animals; ARRIVE guidelines recommended for reporting animal research, and Sex and Gender in Research

Laboratory animals	The hepatocyte-specific SIRT2 knockout mice were obtained from Johan Auwerx Laboratory (Switzerland) and Alb-Cre mice purchased from Jackson Laboratory (U.S) in a C57BL/6 background. The BMDM-specific SIRT2 knockout mice were generated by using Cre driven by Lyz2 (Lysosome C-2) promoters. C57BL/6 mice were purchased from the Shanghai SLAC Laboratory Animal Co. Ltd. 6-12 weeks old and 18 months old male and female mice were used as noted in the manuscript. All mice were maintained in the animal facility of Shanghai Jiao Tong University School of Medicine under specific pathogen-free conditions on a normal chow diet. Mice were housed in an animal facility on a 12-h light cycle at the room temperature of 21–22 °C and humidity of 40–60%, with free access to food and water.

Wild animals	None.
Reporting on sex	The data for key experiments are in both sexes, including (1) SIRT2 was up-regulated in primary hepatocytes of aged mice ; (2) Hepatocyte-specific SIRT2 knockout prevents aging-associated bone loss by suppressing osteoclastogenesis in mice; (3) Medium-derived sEVs of the primary hepatocytes of aged SIRT2-KOhep obviously suppressed osteoclastogenesis; (4) Hepatocyte-specific SIRT2 deficiency obviously enhanced LRG1 protein expression in the cytoplasm and medium-sEVs of aged primary hepatocytes, as well as in the aged osteoclast progenitors in vivo; (5) The nuclear colocalization of p65 was significantly decreased in the primary BMDMs isolated from aged SIRT2-KOhep mice; (6) Young and aged SIRT2-KOhep mice have normal body weight and bone mass; (7) Liver-specific SIRT2 knockout has no effects on Vitamin D synthesis; (8) Similar SIRT2 expression was in the primary BMDMs from young and aged mice; (9) BMDM-specific SIRT2 knockout has no effect on inhibiting osteoclastogenesis and slowing down bone loss in aged mice. Meanwhile, OVX-induced murine model is widely used for postmenopausal osteoporosis study and is only applicable to female mice, so the experiments only used female mice.
Field-collected samples	No field-collected samples were used in the study.
Ethics oversight	The animal experiments were performed in accordance with the approved guidelines by the Institutional Animal Care and Use Committee (IACUC) at Shanghai Jiaotong University School of Medicine(SJTU-SM).

Note that full information on the approval of the study protocol must also be provided in the manuscript.
