# Supplementary material for: SIRT2 regulates extracellular vesicle-mediated liver–bone communication
Source: Nat Metab. 2023 May 15;5(5):821–41. doi: 10.1038/s42255-023-00803-0 (PMC10229428; doi:10.1038/s42255-023-00803-0)
Supplement: Supplementary file 3 — Primers and antibodies. [file 42255_2023_803_MOESM3_ESM.pdf]

Supplementary Tables

Supplementary Table S1.

The cDNA target sequences of shRNAs, and primer sequences used for cloning.

Name	Species	Sequence (5’->3’)
The cDNA target sequences of shRNAs		
shSIRT2-1	Mouse	CCAACCATCTGCCACTACT
shSIRT2-2	Mouse	AGCTGTTGGTGGATGAGCA
shLRG1	Mouse	GGCCTACAGCACCTGGATA
shSIRT2-1	Human	TGGACGAGCTGACCTTGGA
shSIRT2-2	Human	AGCGCGTTTCTTCTCCTGT
Primers sequences used for cloning		
LRG1	Mouse	Forward: ATGGTCTCTTGGCAGCATCA<br>Reverse: TTACTTATCGTCGTCATCCT

Supplementary Tables S2.

The primary antibodies used in Western blot analysis.

Antibody	Company name	Dilution	Catalogue number
TSG101	Proteintech	1:1000	28283-1-AP
HSP70	Proteintech	1:1000	10995-1-AP
Alix	Proteintech	1:1000	12422-1-AP
SIRT2	Sigma	1:500	S8447
LRG1	Abcam	1:250	ab231188
β-tubulin	Proteintech	1:2000	HRP-66240
Anti-rabbit IgG	Cell Signaling Technology	1:5000	#7074
FLAG	Sigma	1:2500	F1804
Anti-mouse IgG	Cell Signaling Technology	1:5000	#7076
β-actin	MBL	1:5000	PM053-7
Acetyl-H4K16	Cell Signaling Technology	1:1000	#13534
Histone H4	Proteintech	1:500	16047-1-AP
p-p65	Cell Signaling Technology	1:1000	#3033
p65	Cell Signaling Technology	1:1000	#8242
p-Smad2/3	Cell Signaling Technology	1:500	#8828
Smad2/3	Cell Signaling Technology	1:1000	#8685
p-Smad1/5	Cell Signaling Technology	1:500	#9516
Smad1	Cell Signaling Technology	1:1000	#6944
Smad5	Cell Signaling Technology	1:1000	#12534
Saa1/Saa2	Abcam	1:1000	ab199030
LaminB	Santa Cruz	1:500	sc6216
anti-goat IgG	Millipore	1:2000	AP106P

Supplementary Table S3.

Primers sequences used for real-time PCR.

Name	Species	Sequence (5’->3’)
RPL13A	Mouse	Forward: GAGGTCGGGTGGAAGTACCA
		Reverse: TGCATCTTGGCCTTTTCCTT
LRG1	Mouse	Forward: CCATGTCAGTGTGCAGATTC
		Reverse: AAGAGTGAGAGGTGGAAGAG
Runx2	Mouse	Forward: GCATGGTGGAGGTACTAGCTG
		Reverse: GCCGTCCACTGTGATTTTG
Sp7	Mouse	Forward: ATGGCGTCCTCTCTGCTTG
		Reverse: TGAAAGGTCAGCGTATGGCTT
ALP	Mouse	Forward: ATCTTTGGTCTGGCTCCCATG
		Reverse: TTTCCCGTTCACCGTCCAC
Osteocalcin	Mouse	Forward: GCAATAAGGTAGTGAACAGACTCC
		Reverse: GTTTGTAGGCGGTCTTCAAGC
GAPDH	Mouse	Forward: ACTGAGGACCAGGTTGTC
		Reverse: TGCTGTAGCCGTATTCATTG
NFATc1	Mouse	Forward: GGAGAGTCCGAGAATCGAGAT
		Reverse: TTGCAGCTAGGAAGTACGTCT
CathespinK	Mouse	Forward: GGAGTTGACTTCCGCAATCCT
		Reverse: ACTTGAACACCCACATCCTGC
Acp5	Mouse	Forward: AGCAGCTCCCTAGAAGATGGA
		Reverse: AGCCACAAATCTCAGGGTGG
DC-STAMP	Mouse	Forward: CCGTGAAGGTAGGAACGCTT
		Reverse: AGATTCAGCGGAGTGGCAAG
CYP2R1	Mouse	Forward: TCAACTGTCGTTCTAAATGGCT
		Reverse: TCTGGAATTGAGTAAGCCTCCC
CYP27A1	Mouse	Forward: TCCAGGCACAGGAGAGTACG

		Reverse: TACTTGGTCTTGTTCAGCACCTGG
NFATc1	Human	Forward: CCAGTCCCTTCCAAGTTTCCA
		Reverse: GCATAGCCATAGTGTTCTTCCT
CTSK	Human	Forward: TTCCCGCAGTAATGACACCC
		Reverse: GGAACCACACTGACCCTGAT
ITGB3	Human	Forward: TGGTAGAAGAGCCAGAGTGTC
		Reverse: TACAGTGGGTTGTTGGCTGT
CALCR	Human	Forward: CCCCAGGATGCAATTTTCTGG
		Reverse: AGAATTGGGGTTGGGTGATTTAG
GAPDH	Human	Forward: TCGGAGTCAACGGATTTGGT
		Reverse: TTCCCGTTCTCAGCCTTGAC

Supplementary Table S4.

Primers sequences used for CHIP.

Name	Species	Sequence (5’->3’)
LRG1-chip-primer 1	Mouse	Forward: CAGACCTGGCACCAAGCTAA<br>Reverse: GCAGGCCTGAATCTGTTCCT
LRG1-chip-primer 2	Mouse	Forward: ACACTGTCCATCTGTCGGTG<br>Reverse: GAGAGCATTGCGGGTCAGAT
LRG1-chip-primer 3	Mouse	Forward: TAACTCTCTGTCCAGCACGC<br>Reverse: TTGTGGGAGATGTCGAAGCC
LRG1-chip-primer 4	Mouse	Forward: CGCCAACCGAAACAAGATGT<br>Reverse: TGACATGGGACCACATTGGC
LRG1-chip-primer 5	Mouse	Forward: GGGCCTACAGCACCTGGATA<br>Reverse: GAGATGTCGAAGCCGTCCTG

Supplementary Table S5.

Characteristics of the female osteoporotic patients **(related to Fig. 7n,o)**.

**Characteristics**	**Women**		
	**Normal Control (n=28)**	**Osteoporotic patients (n=25)**	**p**
**Age (years)**	64.3 (60.0-70.0)	65.2 (61.0-70.0)	0.25
**β-CTX (pg/ml)**	400.99(263.0-564.0)	437.55(203.0-693.0)	0.23
**PINP (ng/ml)**	20.03(10.21-30.22)	22.57(13.2-32.87)	0.08
**BALP (ug/l)**	12.55(4.92-21.51)	13.05(4.68-19.35)	0.67

Characteristics of the study population **(related to Fig. 7p-s).**

**Characteristics**	**Sex**		
	**Men (n=36)**	**Women (n=84)**	**p**
**Age (years)**	65.3 (60.0-70.0)	64.8 (60.0-70.0)	0.44
**β-CTX (pg/ml)**	397.61(167.0-856.7)	457.52(203.0-793.0)	0.04
**PINP (ng/ml)**	22.29(12.40-33.0)	22.02(10.21-39.26)	0.81
**BALP (ug/l)**	10.08(4.34-19.3)	12.93(4.68-26.25)	<0.01

β-CTX, type 1 collagen carboxyl terminal peptide; PINP, type 1 procollagen amino terminal peptide; BALP, bone-specific alkaline phosphatase.
